# Supplementary material for: A Novel Approach for Predicting the Survival of Colorectal Cancer Patients Using Machine Learning Techniques and Advanced Parameter Optimization Methods
Source: Cancers (Basel). 2024 Sep 20;16(18):3205. doi: 10.3390/cancers16183205 (PMC11430446; doi:10.3390/cancers16183205)
Supplement: Supplementary file 1 [file cancers-16-03205-s001.zip › cancers-3205324-supplementary.pdf]

Supplementary materials for “A novel approach for predicting the survival of colorectal cancer patients using machine learning techniques and advanced parameter optimization methods”

Dataset

Complete dataset (before preprocessing)

ID	Field	Description	Type (DBF)	Size/Text	Domain/Data Format
6	ESCOLARI	Patient's education level code	INT	1	Domain:<br>1 - ILLITERATE,<br>2 - INCOMPLETE ELEMENTARY,<br>3 - COMPLETE ELEMENTARY,<br>4 - HIGH SCHOOL,<br>5 - COLLEGE,<br>9 - UNKNOWN
10	IDADE	Patient's age	INT	3	
11	SEXO	Patient's gender	INT	1	Domain:<br>1 - MALE,<br>2 - FEMALE
12	UFNASC	State of birth	CHAR	2	Other options:<br>SI - NO INFORMATION,<br>OP - OTHER COUNTRY

17	UFRESID	State of residence	CHAR	2	Other options:<br>OP - OTHER COUNTRY
18	IBGE	Code of patient's city of residence according to IBGE with verification digit	CHAR	7	
19	CIDADE	City of residence	CHAR	200	
21	CATEATEN D	Category of care f diagnosis	INT	1	Domain:<br>1 - PRIVATE INSURANCE,<br>2 - SUS,<br>3 - PRIVATE,<br>9 - UNKNOWN
22	DTCONSUL T	Date of 1st consultation	DATE	10	Format: DD/MM/YYYY
23	CLINICA	Clinic code	INT	2	Domínio:<br><br>1 – ALLERGY/IMMUNOLOGY<br>2 – CARDIAC SURGERY<br>3 – HEAD AND NECK SURGERY<br>4 – GENERAL SURGERY<br>5 – PEDIATRIC SURGERY<br>6 – PLASTIC SURGERY<br>7 – THORACIC SURGERY<br>8 – VASCULAR SURGERY<br>9 – MEDICAL CLINIC<br>10 – DERMATOLOGY

					11 – ENDOCRINOLOGY<br>12 – GASTRIC SURGERY<br>13 – GASTROENTEROLOGY<br>14 – GERIATRICS<br>15 – GYNECOLOGY<br>16 – GYNECOLOGY/OBSTETRICS<br>17 – HEMATOLOGY<br>18 – INFECTIOUS DISEASES<br>19 – NEPHROLOGY<br>20 – NEUROSURGERY<br>21 – NEUROLOGY<br>22 – OPHTHALMOLOGY<br>23 – SURGICAL ONCOLOGY<br>24 – CLINICAL ONCOLOGY<br>25 – PEDIATRIC ONCOLOGY<br>26 – ORTHOPEDICS<br>27 – OTORHINOLARYNGOLOGY<br>28 – PEDIATRICS<br>29 – PNEUMOLOGY<br>30 – PROCTOLOGY<br>31 – RADIOTHERAPY<br>32 – UROLOGY<br>33 – MASTOLOGY<br>34 – CUTANEOUS ONCOLOGY<br>35 – PELVIC SURGERY<br>36 – ABDOMINAL SURGERY<br>37 – DENTISTRY<br>38 – LIVER TRANSPLANT<br>99 – IGNORED
24	DIAGPREV	Previous diagnosis and treatment	INT	1	Domain:<br>1 - NO DIAGNOSIS/NO TRE[illegible]<br>2 - DIAGNOSIS/NO TREATM[illegible]<br>3 - DIAGNOSIS/TREATMEN[illegible]<br>4 - OTHERS
25	DTDIAG	Date of diagnosis	DATE	10	Format: DD/MM/YYYY

26	BASEDIAG	Diagnostic base code	INT	1	Domain:<br>1 - CLINICAL EXAM,<br>2 - NON-MICROSCOPIC AUXILIARY TESTS,<br>3 - MICROSCOPIC CONFIRMATION,<br>4 - UNKNOWN
27	TOPO	Topography code	CHAR	4	Format: C999,<br>Diagnosis date: Until 2005 - CID-O 2nd edition,<br>From 2006 - CID-O 3rd edition
28	TOPOGRUP	Topography group	CHAR	3	
29	DESCTOPO	Topography description	CHAR	80	
30	MORFO	Morphology code	CHAR	5	Format: 99999, Diagnosis date: Until 2005 - CID-O 2nd edition, From 2006 - CID-O 3rd edition
31	DESCMORFO	Morphology descr	CHAR	80	
32	EC	Clinical stage	CHAR	5	

					Diagnosis date: Until 2005 - TNM 5th edition, 2006 to 2013 - TNM 6th edition, From 2014 - TNM 7th edition
33	ECGRUP	Clinical staging gr[illegible]	CHAR	3	
34	T	TNM Classificatior	CHAR	5	Diagnosis date: Until 2005 - TNM 5th edition, 2006 to 2013 - TNM 6th edition, From 2014 - TNM 7th edition
35	N	TNM Classificatior	CHAR	5	Diagnosis date: Until 2005 - TNM 5th edition, 2006 to 2013 - TNM 6th edition, From 2014 - TNM 7th edition
36	M	TNM Classificatior	CHAR	3	Diagnosis date: Until 2005 - TNM 5th edition, 2006 to 2013 - TNM 6th edition, From 2014 - TNM 7th edition
37	PT	Post-surgical staging	CHAR	5	
38	PN	Post-surgical staging	CHAR	5	
39	PM	Post-surgical staging	CHAR	3	
40	S	TNM Classificatior	INT	1	Diagnosis date: Until 2005 - TNM 5th edition, 2006 to 2013 - TNM 6th edition, From 2014 - TNM 7th edition;

					Domain:<br>0,<br>1,<br>2,<br>3,<br>8 - NOT APPLICABLE,<br>9 - X
41	G	TNM Classificatior (Grade)	CHAR	5	Diagnosis date: Until 2005 - TNM 5th edition,<br>2006 to 2013 - TNM 6th edition, From 2014 –<br>TNM 7th edition;<br><br>Domain (except C40, C41, C381, C382, C383, C47, C48, C49):<br>0,<br>1,<br>2,<br>3,<br>4,<br>8 - NOT APPLICABLE,<br>9 - X;<br><br>Domain (only for C40, C41, C381, C382, C383, C47, C48, C49):<br>HIGH,<br>LOW,<br>8 - NOT APPLICABLE,<br>9 - X
42	LOCALTNM	TNM Classificatior Location	INT	1	Diagnosis date: Until 2005 - TNM 5th edition,<br>2006 to 2013 - TNM 6th edition,<br>From 2014 - TNM 7th edition;<br>Domain:<br>1 - UPPER,<br>2 - MIDDLE,<br>3 - LOWER,<br>8 - NOT APPLICABLE,<br>9 - X
43	IDMITOTIC	TNM Classificatior Mitotic Index	INT	1	

					Diagnosis date:<br>Until 2005 - TNM<br>5th edition,<br>2006 to 2013 -<br>TNM 6th edition,<br>From 2014 - TNM<br>7th edition;<br>Domain:<br>1 - HIGH,<br>2 - LOW,<br>8 - NOT<br>APPLICABLE,<br>9 - X
44	PSA	TNM Classificatior	INT	1	Diagnosis date: Until 2005<br>- TNM 5th edition,<br>2006 to 2013 - TNM 6th<br>edition,<br>From 2014 - TNM 7th<br>edition;<br>Domain:<br>1 - LESS THAN 10,<br>2 - GREATER OR EQUAL<br>TO 10 AND LESS THAN<br>20,<br>3 - GREATER OR EQUAL<br>TO 20,<br>8 - NOT APPLICABLE,<br>9 - X
45	GLEASON	TNM Classificatior<br>Gleason	INT	1	Diagnosis date: Until 2005 -<br>TNM 5th edition,<br>2006 to 2013 - TNM 6th<br>edition,<br>From 2014 - TNM 7th<br>edition;<br>Domain:<br>1 - LESS OR EQUAL TO 6,<br>2 - EQUAL TO 7,<br>3 - GREATER OR EQUAL<br>TO 8,<br>8 - NOT APPLICABLE,<br>9 – X

46	OUTRACLA	Other staging classification	CHAR	20	
47	META01	Metastasis	CHAR	3	Format: C99, CID-O 3rd edition (topography)
48	META02	Metastasis	CHAR	3	Format: C99, CID-O 3rd edition (topography)
49	META03	Metastasis	CHAR	3	Format: C950, CID-O 3rd edition (topography)
50	META04	Metastasis	CHAR	3	Format: C99, CID-O 3rd edition (topography)
51	DTTRAT	Start date of treatment	DATE	10	Format: DD/MM/YYYY
52	NAOTRAT	Code for reason treatment was not carried out	INT	1	Domain:<br>1 – TREATMENT REFUSED<br>2 – ADVANCED DISEASE, LACK OF CLINICAL CONDITIONS<br>3 – OTHER ASSOCIATED DISEASES<br>4 – TREATMENT ABANDONED<br>5 – DEATH DUE TO CANCER<br>6 – DEATH DUE TO OTHER CAUSES, UNSPECIFIED<br>7 – OTHER<br>8 – NOT APPLICABLE (IF TREATMENT OCCURRED)<br>9 – NO INFORMATION
53	TRATAMENTO	Code of combination of treatments performed	CHAR	1	Domain:<br>A – Surgery<br>B – Radiotherapy<br>C – Chemotherapy<br>D – Surgery + Radiotherapy<br>E – Surgery + Chemotherapy<br>F – Radiotherapy + Chemotherapy<br>G – Surgery + Radiotherapy + Chemotherapy<br>H – Surgery + Radiotherapy + Chemotherapy + Hormonal Therapy<br>I – Other treatment combinations<br>J – No treatment performed
54	TRATHOSP	Code of combination of treatments performed at hospital	CHAR	1	Domain:<br>A – Surgery<br>B – Radiotherapy<br>C – Chemotherapy<br>D – Surgery + Radiotherapy

					E – Surgery + Chemotherapy<br>F – Radiotherapy + Chemotherapy<br>G – Surgery + Radiotherapy + Chemotherapy<br>H – Surgery + Radiotherapy + Chemotherapy + Hormonal Therapy<br>I – Other treatment combinations<br>J – No treatment performed
55	TRATFANTES	Code of combination of treatments performed outside hospital (before/during admission)	CHAR	1	Domain:<br>A – Surgery<br>B – Radiotherapy<br>C – Chemotherapy<br>D – Surgery + Radiotherapy<br>E – Surgery + Chemotherapy<br>F – Radiotherapy + Chemotherapy<br>G – Surgery + Radiotherapy + Chemotherapy<br>H – Surgery + Radiotherapy + Chemotherapy + Hormonal Therapy<br>I – Other treatment combinations<br>J – No treatment performed
56	TRATFAPOS	Code of combination of treatments performed outside hospital (after admission)	CHAR	1	Domain:<br>A – Surgery<br>B – Radiotherapy<br>C – Chemotherapy<br>D – Surgery + Radiotherapy<br>E – Surgery + Chemotherapy<br>F – Radiotherapy + Chemotherapy<br>G – Surgery + Radiotherapy + Chemotherapy<br>H – Surgery + Radiotherapy + Chemotherapy + Hormonal Therapy<br>I – Other treatment combinations<br>J – No treatment performed
57	NENHUM	Treatment received at hospital = none	INT	1	0 – NO<br>1 – YES
58	CIRURGIA	Treatment received at hospital = surgery	INT	1	0 – NO<br>1 – YES
59	RADIO	Treatment received at	INT	1	0 – NO<br>1 – YES

		hospital = radiotherapy			
60	QUIMIO	Treatment received at hospital = chemotherapy	INT	1	0 – NO<br>1 – YES
61	HORMONIO	Treatment received at hospital = hormonal therapy	INT	1	0 – NO<br>1 – YES
62	TMO	Treatment received at hospital = bone marrow transplant	INT	1	0 – NO<br>1 – YES
63	IMUNO	Treatment received at hospital = immunotherapy	INT	1	0 – NO<br>1 – YES
64	OUTROS	Treatment received at hospital = others	INT	1	0 – NO<br>1 – YES
65	NENHUMANT	Treatment received outside hospital before admission = none	INT	1	0 – NO<br>1 – YES
66	CIRURANT	Treatment received outside hospital before admission = surgery	INT	1	0 – NO<br>1 – YES
67	RADIOANT	Treatment received outside hospital before admission = radiotherapy	INT	1	0 – NO<br>1 – YES
68	QUIMIOANT	Treatment received outside hospital before admission = chemotherapy	INT	1	0 – NO<br>1 – YES
69	HORMOANT	Treatment received outside hospital before admission = hormonal therapy	INT	1	0 – NO<br>1 – YES
70	TMOANT	Treatment received outside hospital before admission = bone marrow transplant	INT	1	0 – NO<br>1 – YES

71	IMUNOANT	Treatment received outside hospital before admission = immunotherapy	INT	1	0 – NO<br>1 – YES
72	OUTROSANT	Treatment received outside hospital before admission = others	INT	1	0 – NO<br>1 – YES
73	NENHUMAPOS	Treatment received outside hospital after admission = none	INT	1	0 – NO<br>1 – YES
74	CIRURAPOS	Treatment received outside hospital after admission = surgery	INT	1	0 – NO<br>1 – YES
75	RADIOAPOS	Treatment received outside hospital after admission = radiotherapy	INT	1	0 – NO<br>1 – YES
76	QUIMIOAPOS	Treatment received outside hospital after admission = chemotherapy	INT	1	0 – NO<br>1 – YES
77	HORMOAPOS	Treatment received outside hospital after admission = hormonal therapy	INT	1	0 – NO<br>1 – YES
78	TMOAPOS	Treatment received outside hospital after admission = bone marrow transplant	INT	1	0 – NO<br>1 – YES
79	IMUNOAPOS	Treatment received outside hospital after admission = immunotherapy	INT	1	0 – NO<br>1 – YES
80	OUTROSAPOS	Treatment received outside hospital after admission = others	INT	1	0 – NO<br>1 – YES
81	DTULTINFO	Date of last patient	DATE	10	Format: DD/MM/YYYY

		information			
82	ULTINFO	Last information about the patient	INT	1	Domain:<br>1 – ALIVE WITH CANCER<br>2 – ALIVE, UNSPECIFIED<br>3 – DEATH DUE TO CANCER<br>4 – DEATH DUE TO OTHER CAUSES, UNSPECIFIED
83	CONSDIAG	Difference in days between consultation and diagnosis	NUM	10,1	VALUE IN DAYS
84	TRATCONS	Difference in days between consultation and treatment	NUM	10,1	VALUE IN DAYS
85	DIAGTRAT	Difference in days between diagnosis and treatment	NUM	10,1	VALUE IN DAYS
86	ANODIAG	Year of diagnosis	INT	4	Format: 9999
87	CICI	Childhood tumor	CHAR	5	
88	CICIGRUP	Childhood tumor – Group	CHAR	80	
89	CICISUBGRU	Childhood tumor – Subgroup	CHAR	80	
90	FAIXAETAR	Patient age group	CHAR	5	
91	LATERALI	Laterality	INT	1	Domain:<br>1 – RIGHT<br>2 – LEFT<br>3 – BILATERAL<br>8 – NOT APPLICABLE
92	INSTORIG	Origin institution	CHAR	200	Required only if DIAGPREV = 03 – WITH DIAGNOSIS/WITH TREATMENT
93	DRS	DRS	CHAR	200	
94	RRAS	RRAS	CHAR	200	
95	PERDASEG	Loss of follow-up	INT	1	Domain:<br>0 – No<br>1 – Yes

					8 – Not applicable (excluded from indicator calculation)
96	ERRO	Admission with error	INT	1	Domain:<br>0 – No Error<br>1 – With Error
97	DTPREENCH	Date of completion	DATE	10	Format: DD/MM/YYYY
99	DTRECIDIVA	Date of last recurrence	DATE	10	Format: DD/MM/YYYY
100	RECNENHUM	No recurrence	INT	1	Domain: 0 – No<br>1 – Yes

Complete dataset used in article (after preprocessing)

Name	Description	Data type
SEXO	Patient’s gender	int
IDADE	Patient’s age	int
ESCOLARI	Education level	int
UFNASC	Birthplace	char
IBGE	Postal code	char
CIDADE	City of residence	char
CATEATEND	Health service provider	int
DTCONSULT	Date of first consultation	date
CLINICA	Diagnosis department	int
DIAGPREV	Diagnosis status	int
DTDIAG	Date of diagnosis	date
BASEDIAG	Basis of suspicion	int
TOPO	Cancer subgroup	char
TOPOGRUP	Cancer group	char
DESCTOPO	Cancer location	char
MORFO	Morphological code	char
DESCMORFO	Morphological subtype	char
EC	Clinical staging	char
ECGRUP	Staging group	char
T	Tumor growth	char
N	Spread to lymph nodes	char
M	Distant metastases	char
PT	PT scale	char
PN	PN scale	char
PM	PM scale	char
S	S scale	int
G	G scale	char
IDMITOTIC	Mitotic index	int
PSA	PSA classification	int
GLEASON	Gleason score	int

OUTRACLA	OUTRACLA code	char
META01	Metastasis 1	char
META02	Metastasis 2	char
META03	Metastasis 3	char
META04	Metastasis 4	char
DTTRAT	Treatment start date	date
NAOTRAT	Reason for no treatment	int
TRATAMENTO	Type of treatment	char
TRATHOSP	Treatment in hospital	char
TRATFANTES	Treatment before hospital	char
TRATFAPOS	Treatment after hospital	char
NENHUM	No treatment in hospital	boolean
CIRURGIA	Surgery in hospital	boolean
RADIOa	Radiotherapy in hospital	boolean
QUIMIO	Chemotherapy in hospital	boolean
HORMONIO	Hormone therapy in hospital	boolean
TMO	Stem cell transplant in hospital	boolean
IMUNO	Immunotherapy in hospital	boolean
OUTROS	Other methods in hospital	boolean
NENHUMANT	No treatment outside hospital	boolean
CIRURANT	Surgery outside hospital	boolean
RADIOANT	Radiotherapy outside hospital	boolean
QUIMIOANT	Chemotherapy outside hospital	boolean
HORMOANT	Hormone therapy outside hospital	boolean
TMOANT	Stem cell transplant outside hospital	boolean
IMUNOANT	Immunotherapy outside hospital	boolean
OUTROANT	Other methods outside hospital	boolean
NENHUMAPOS	No treatment anywhere	boolean
CIRURAPOS	Surgery anywhere	boolean
RADIOAPOS	Radiotherapy anywhere	boolean
QUIMIOAPOS	Chemotherapy anywhere	boolean
HORMOAPOS	Hormone therapy anywhere	boolean
TMOAPOS	Stem cell transplant anywhere	boolean
IMUNOAPOS	Immunotherapy anywhere	boolean
OUTROAPOS	Other methods anywhere	boolean
DTULTINFO	Last info date	date
ULTINFO	Last info status	int
CONSDIAG	Days from consultation to diagnosis	int
TRATCONS	Days from consultation to treatment	int

DIAGTRAT	Days from diagnosis to treatment	int
ANODIAG	Year of diagnosis	int
FAIXAETAR	Age group	char
LATERALI	Tumor laterality	char
INSTORIG	Treatment location if previously diagnosed	char
DRS	NFZ department	char
RRAS	Regional health network	char
DTPREENCH	End of treatment date	date
REGISTRADO	Registration status	char
DTRECIDIVA	Recurrence date	date
RECNENHUM	No recurrence	int
RECLOCAL	Local recurrence	int
RECREGIO	Regional recurrence	int
RECDIST	Distant metastasis	int
REC01	Metastasis type 1	char
REC02	Metastasis type 2	char
REC03	Metastasis type 3	char
REC04	Metastasis type 4	char
HABILIT2	Certification status	char

1. Results

1.1. Optuna

1.1.1. Alive 1 year

1.1.1.1. Random Forest

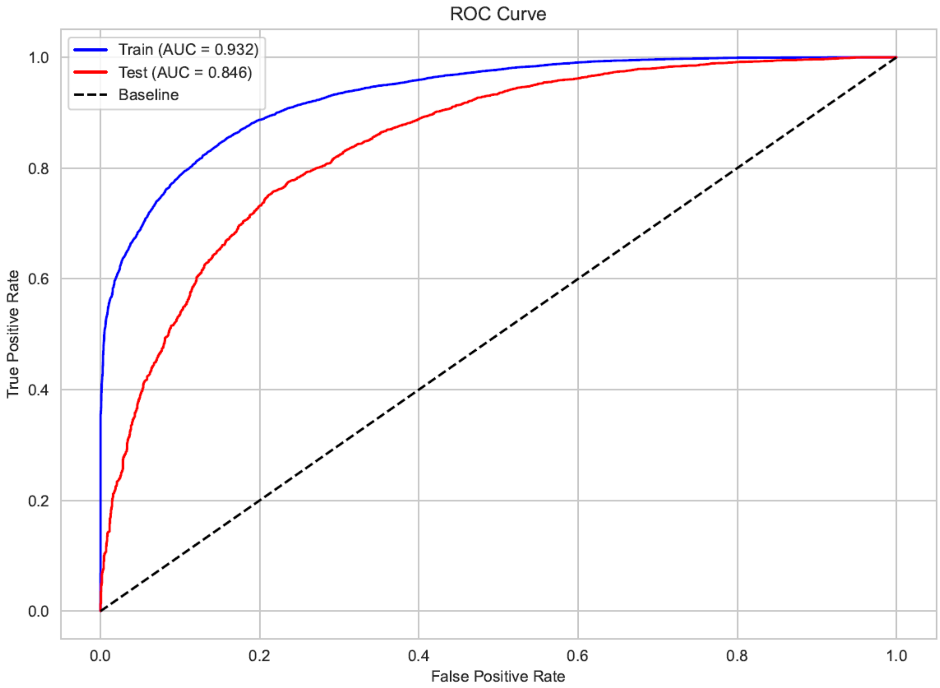

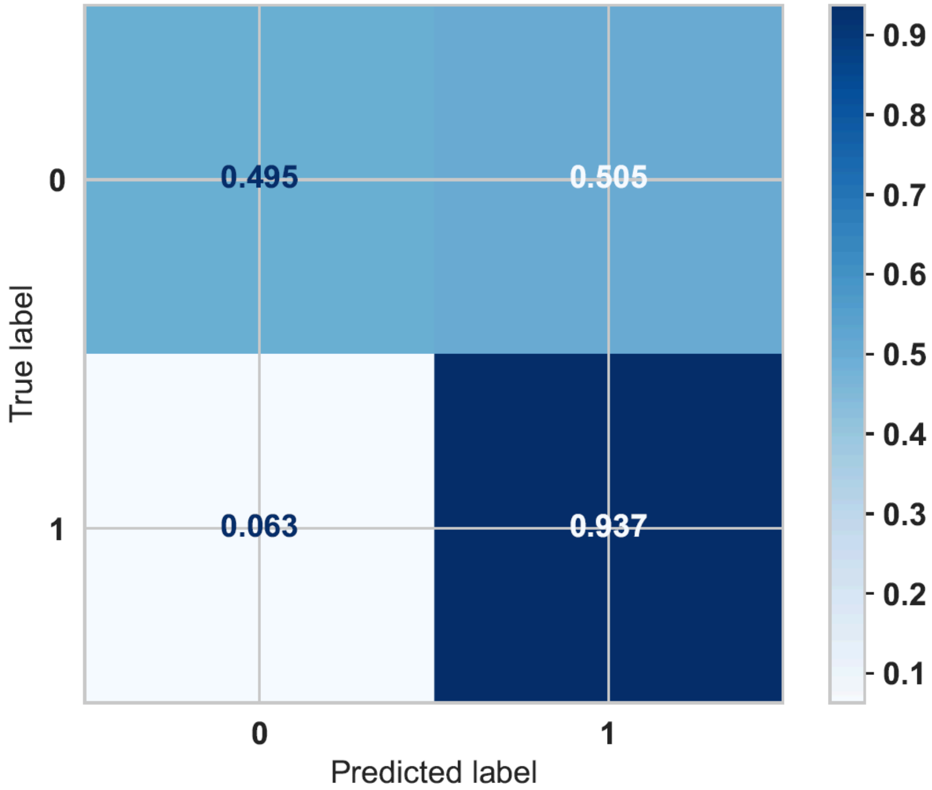

1.1.1.2. XGBoost

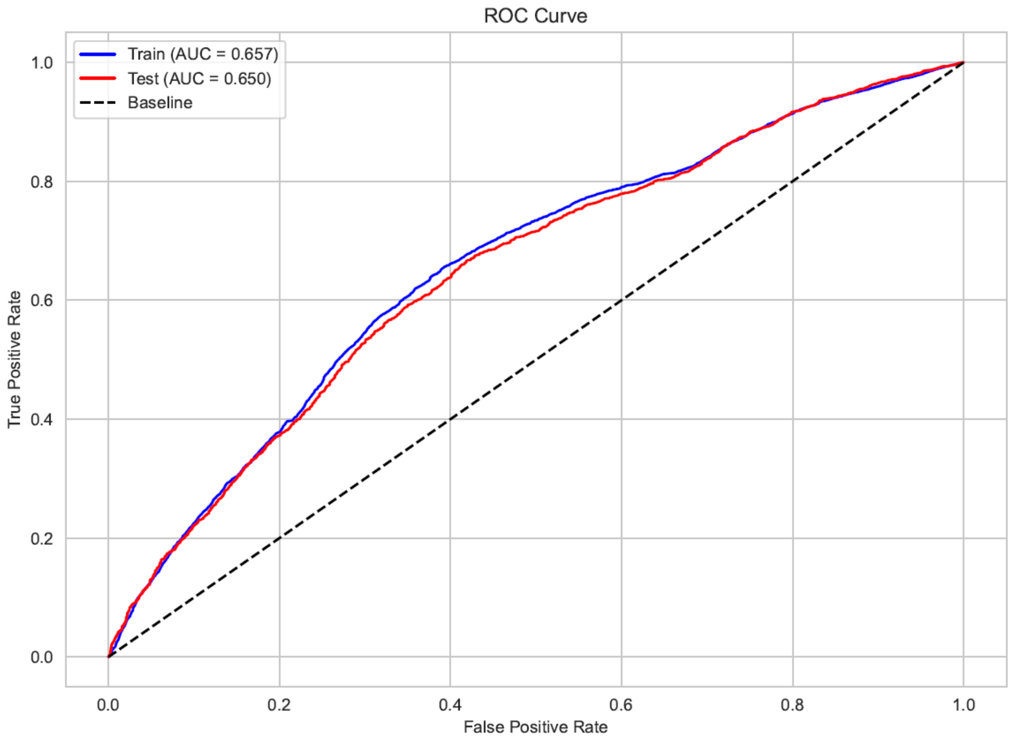

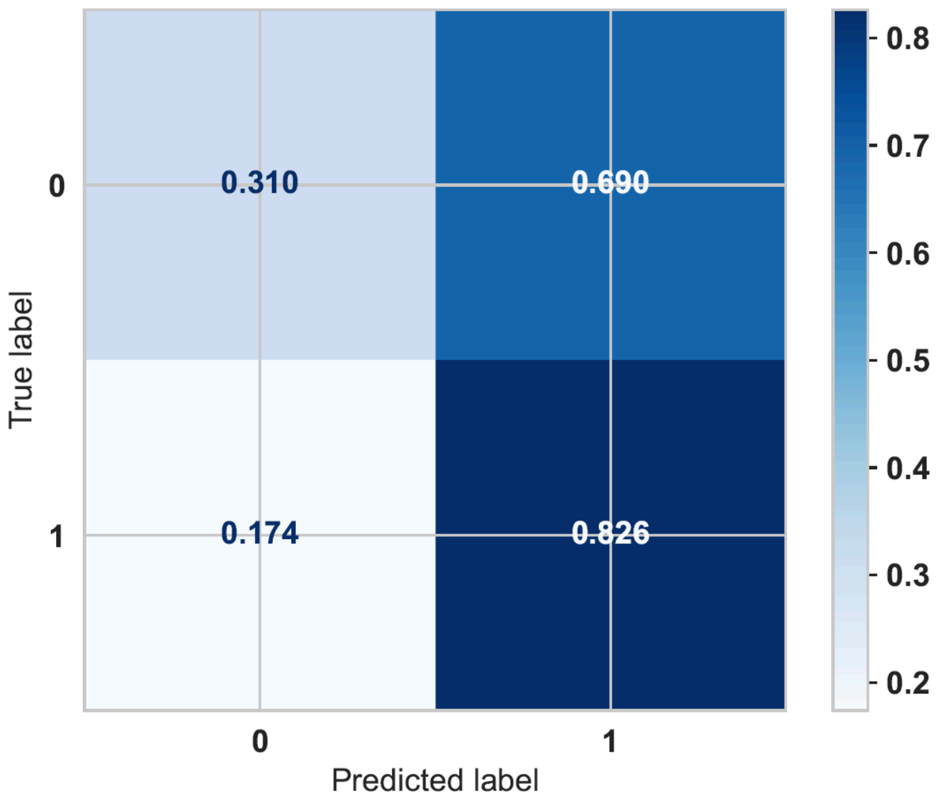

1.1.1.3. CatBoost

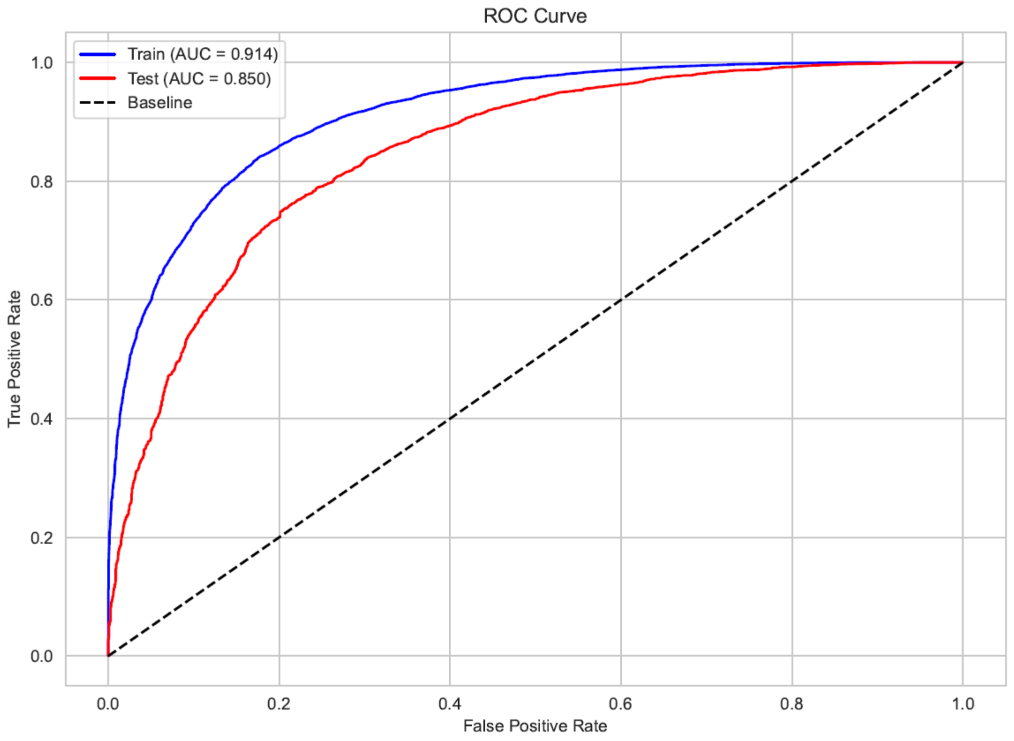

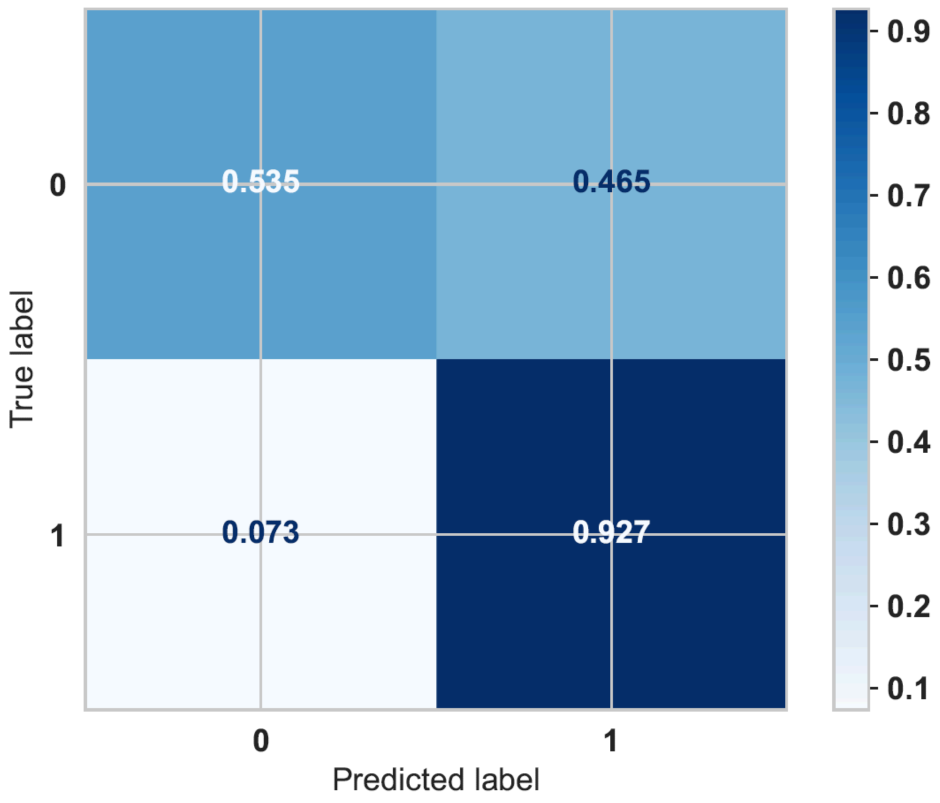

1.1.1.4. DecisionTreeClassifier

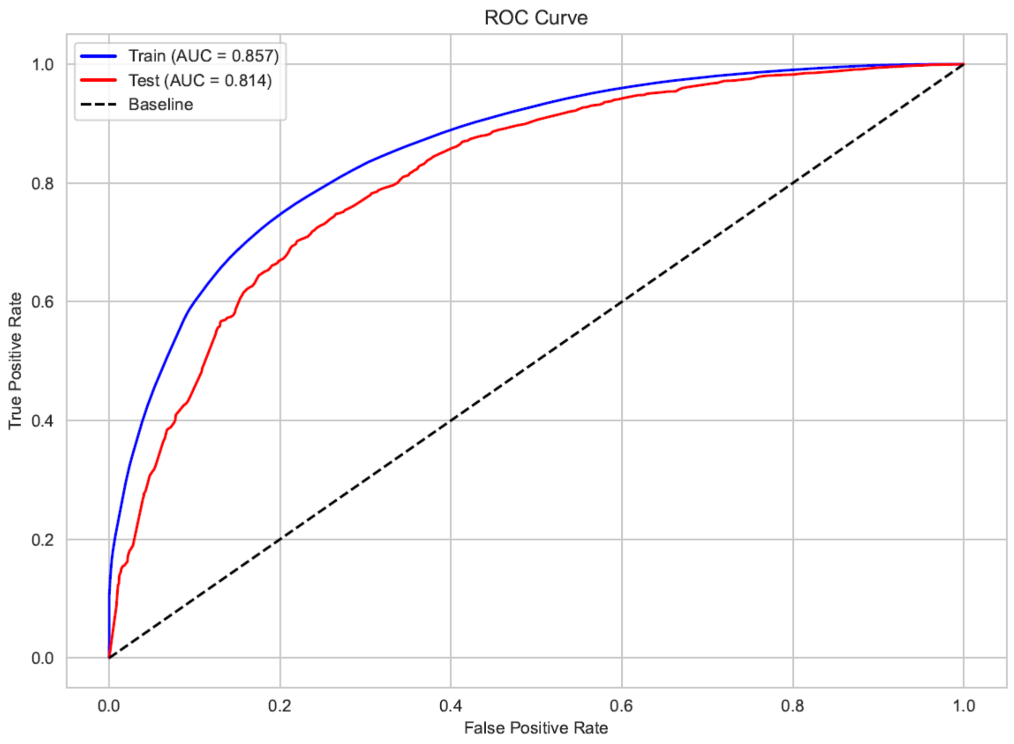

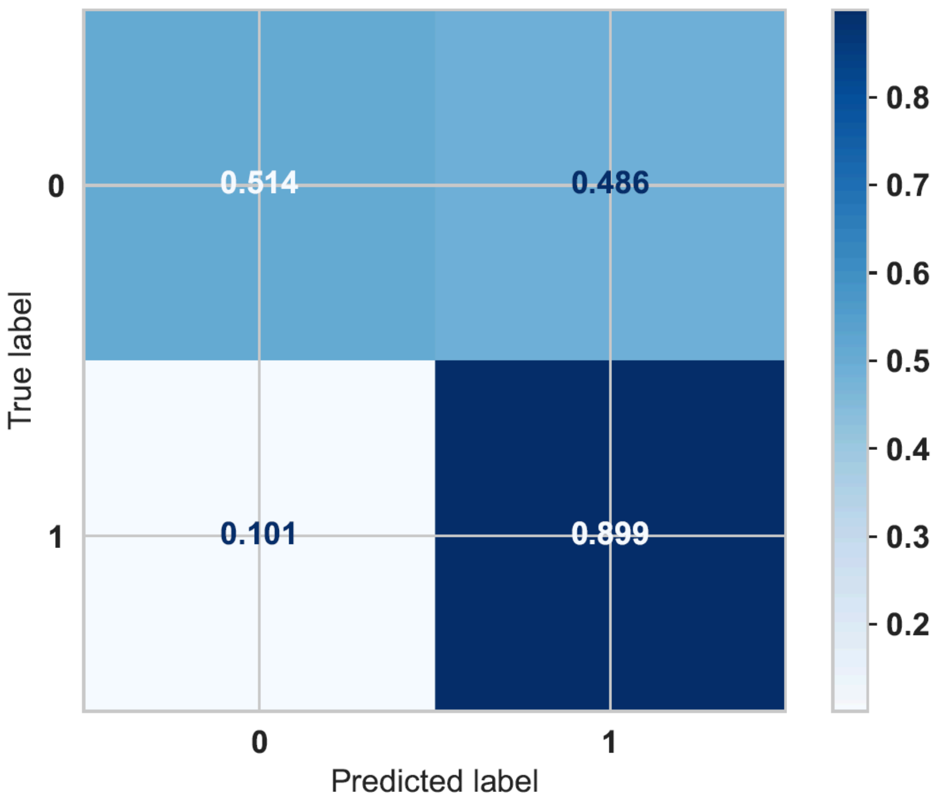

1.1.1.5. ExtraTreesClassifier

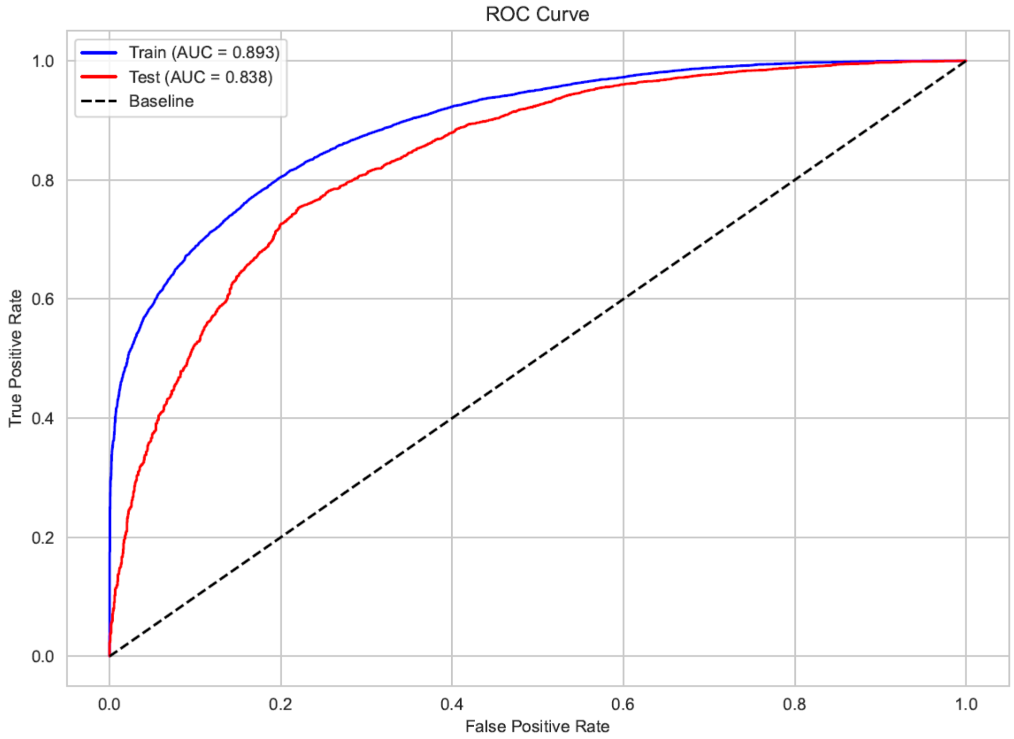

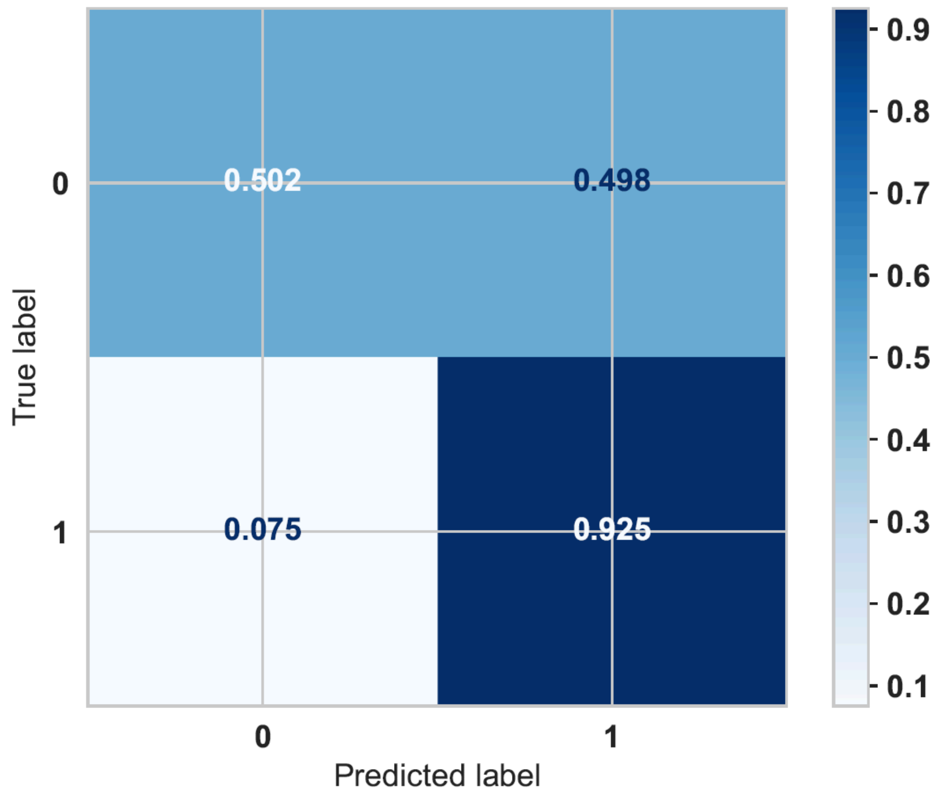

1.1.1.6. GradientBoosting

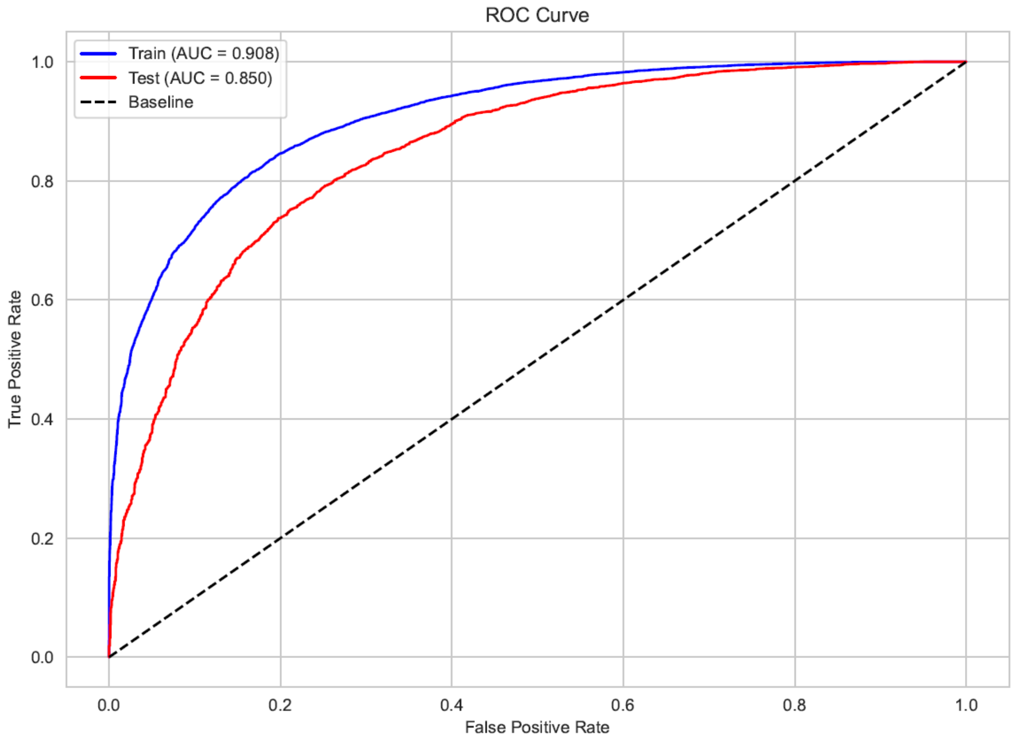

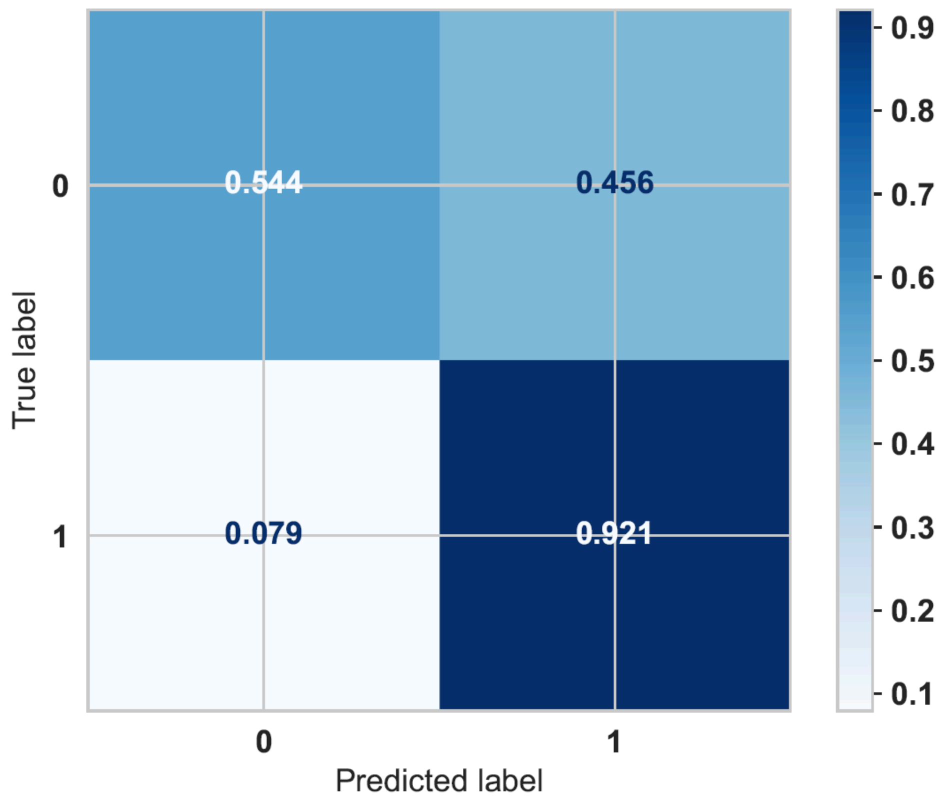
0.544
0.456
0.079
0.921
True label
Predicted label
0
1
0
1
0.9
0.8
0.7
0.6
0.5
0.4
0.3
0.2
0.1

1.1.1.7. KNN

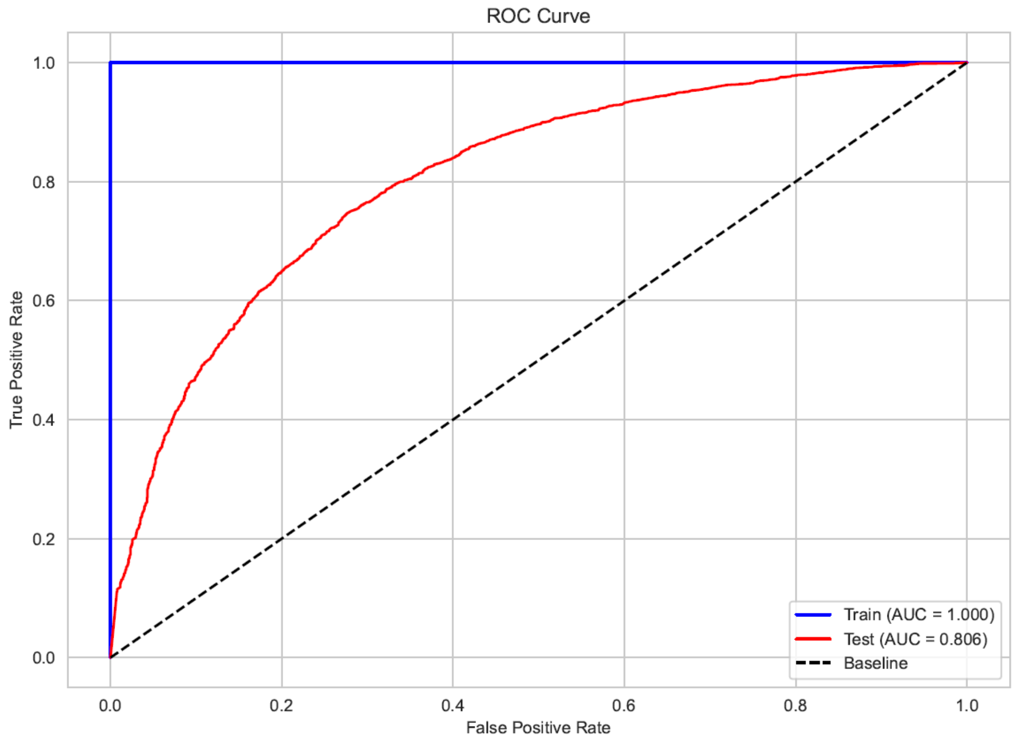
ROC Curve
True Positive Rate
False Positive Rate
Train (AUC = 1.000)
Test (AUC = 0.806)
Baseline
0.0
0.2
0.4
0.6
0.8
1.0

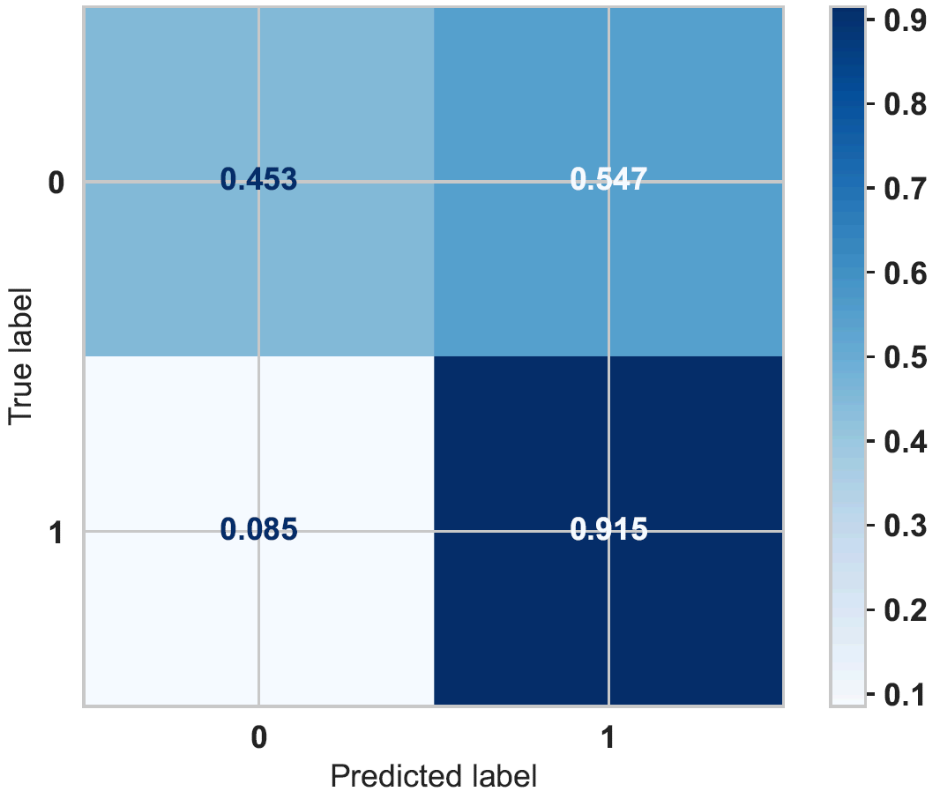

1.1.1.8. lightgbm

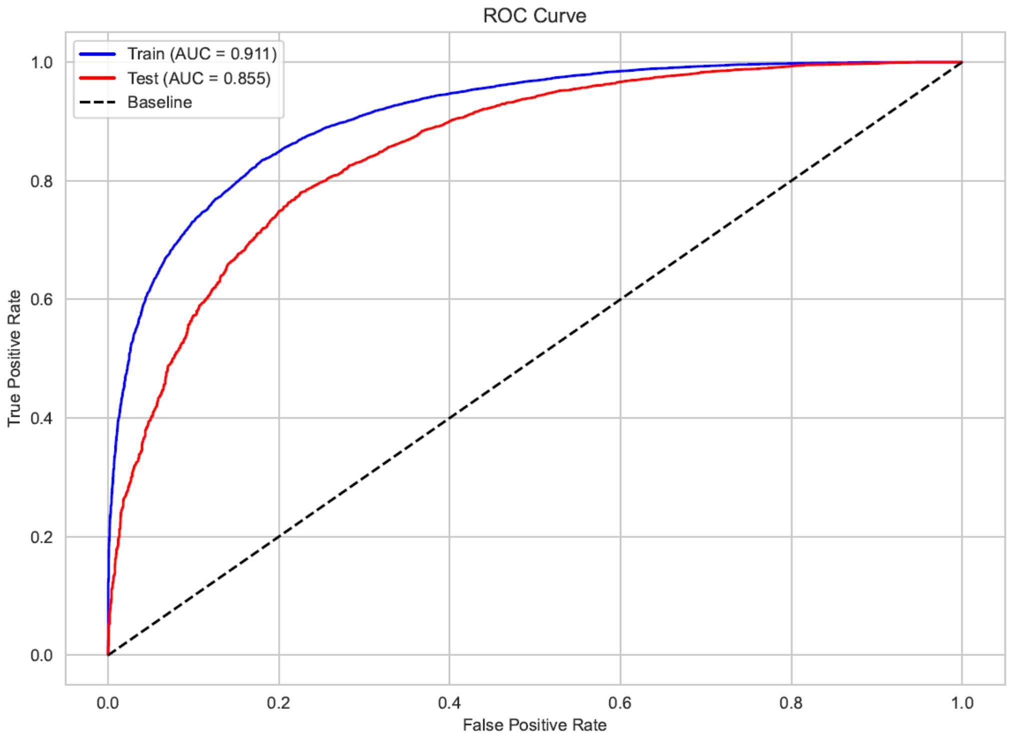

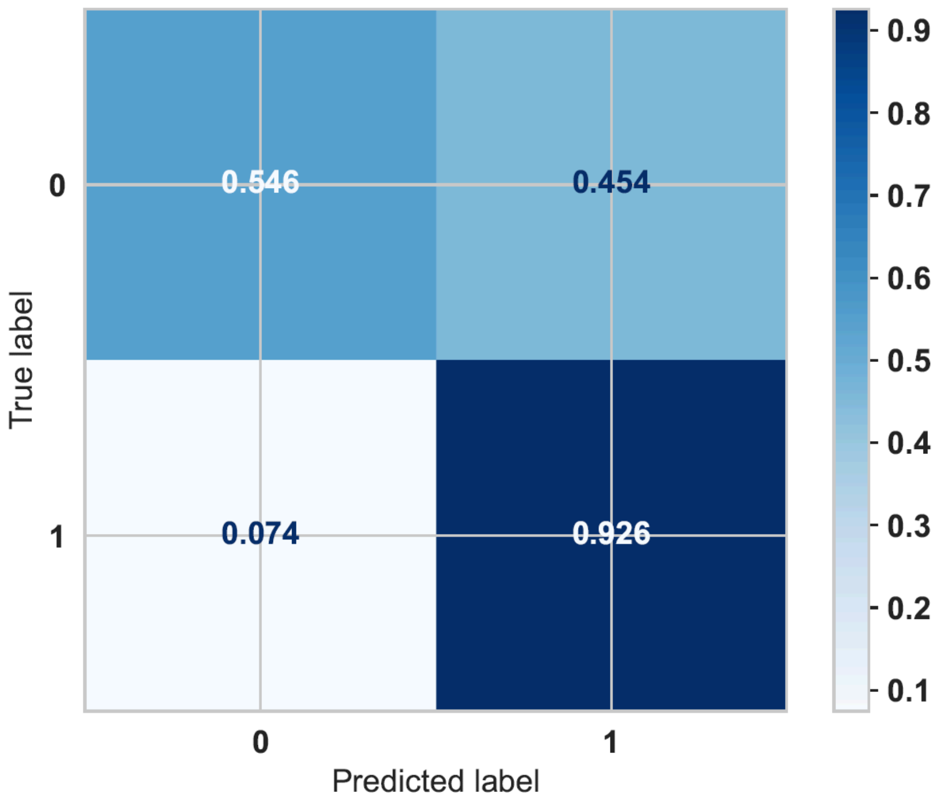

1.1.2. Alive 3 year

1.1.2.1. Random Forest

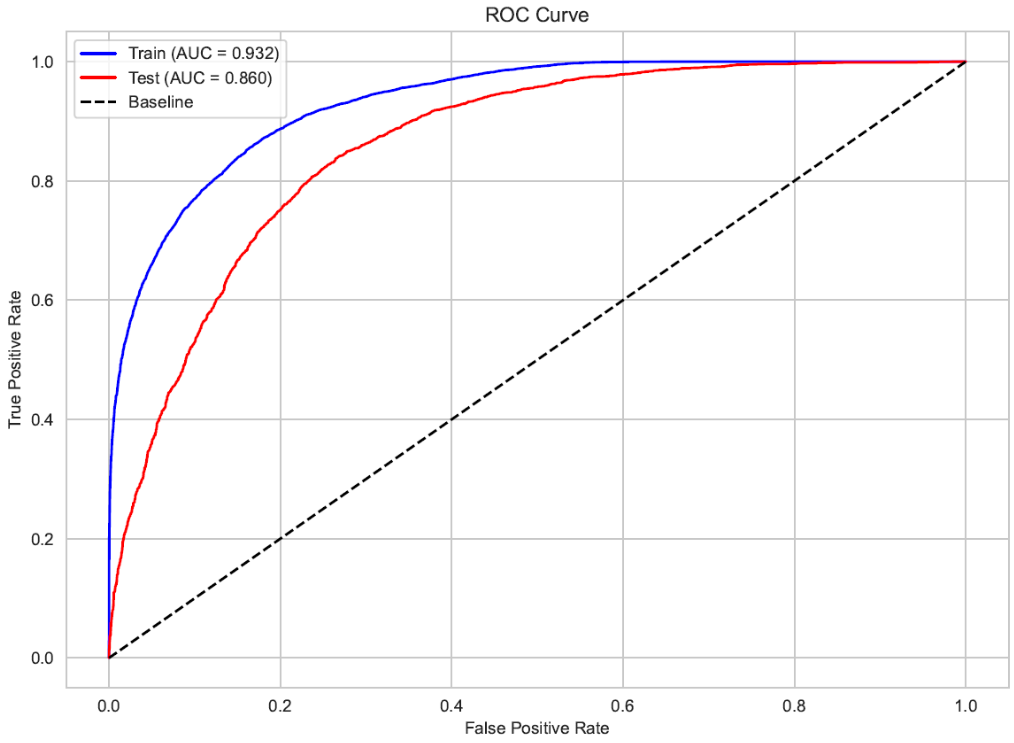

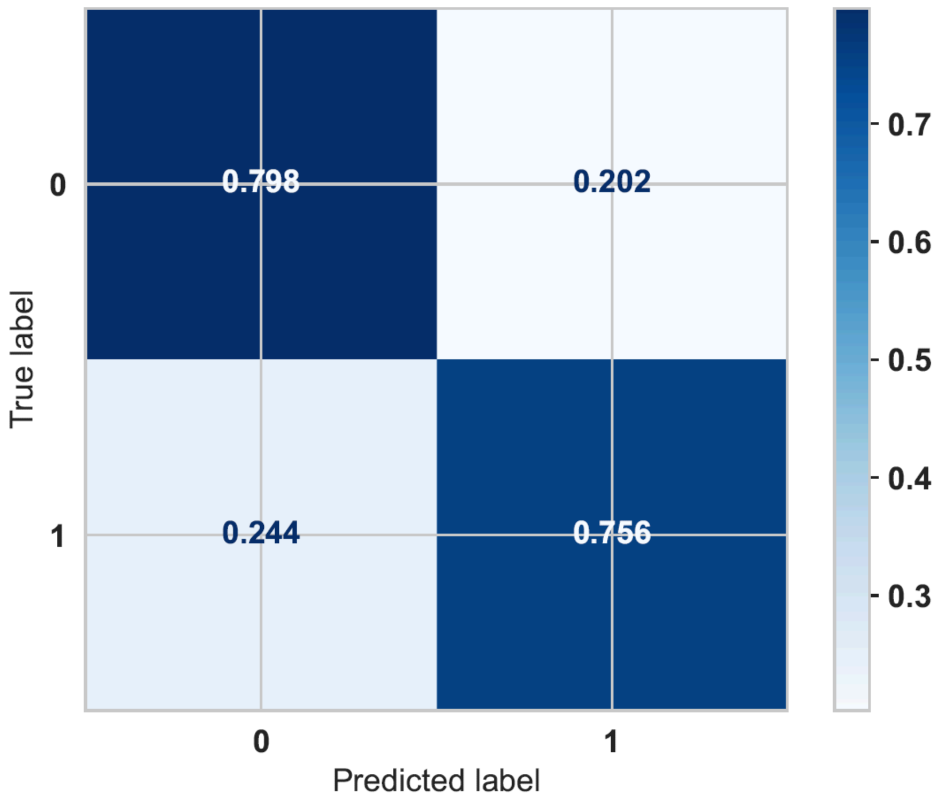

1.1.2.2. XGBoost

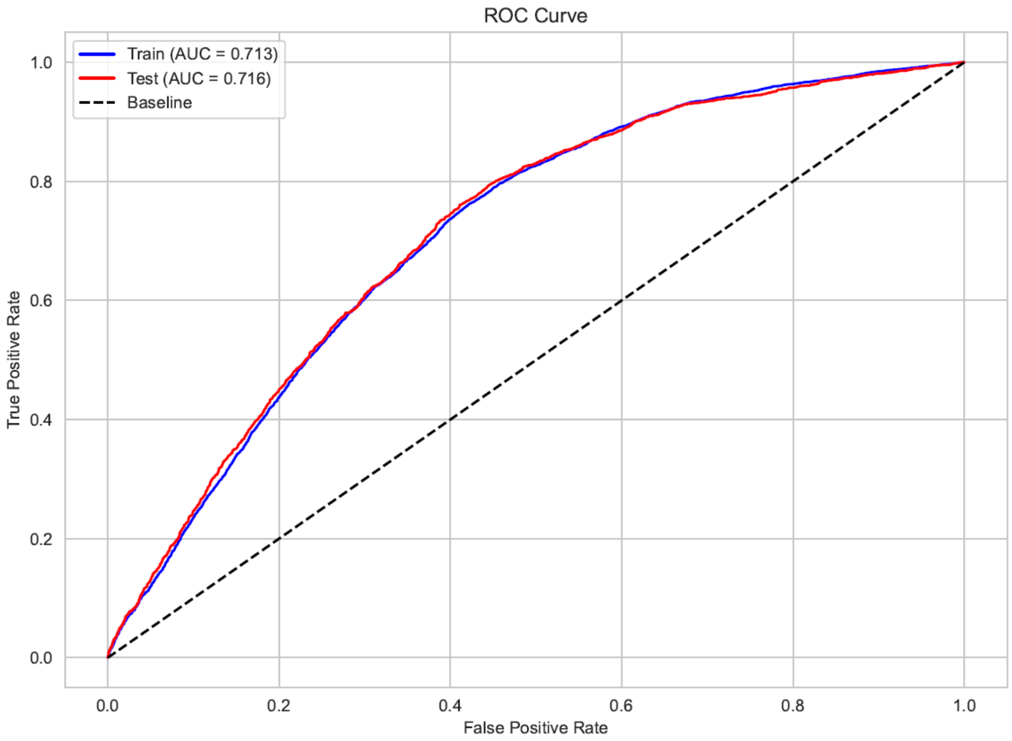

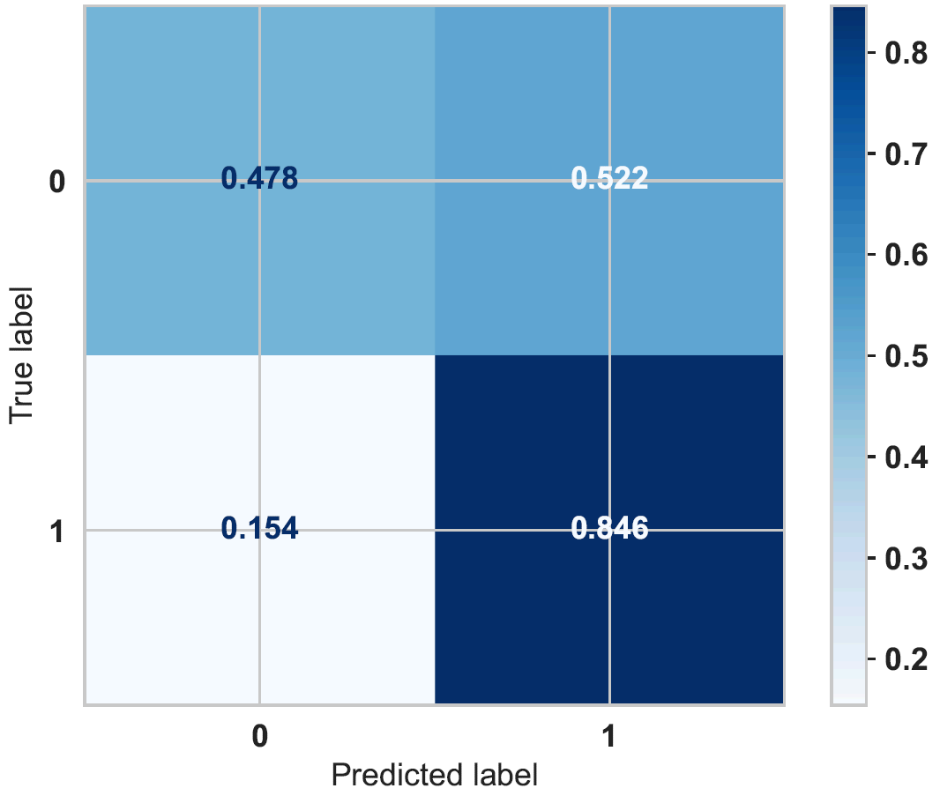

1.1.2.3. CatBoost

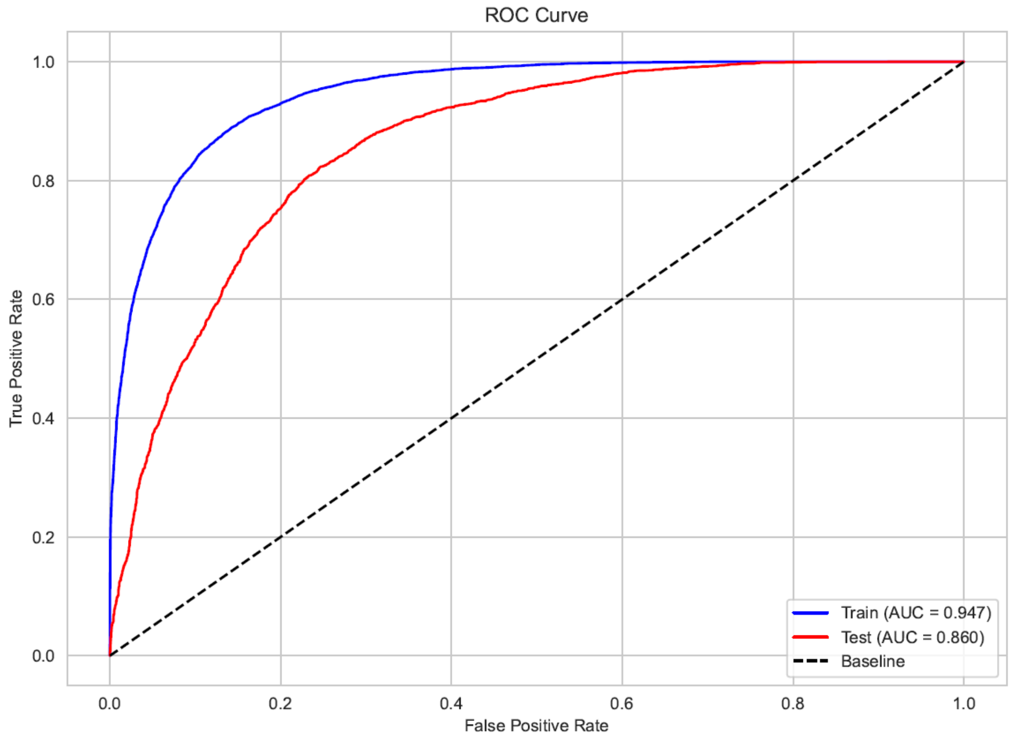

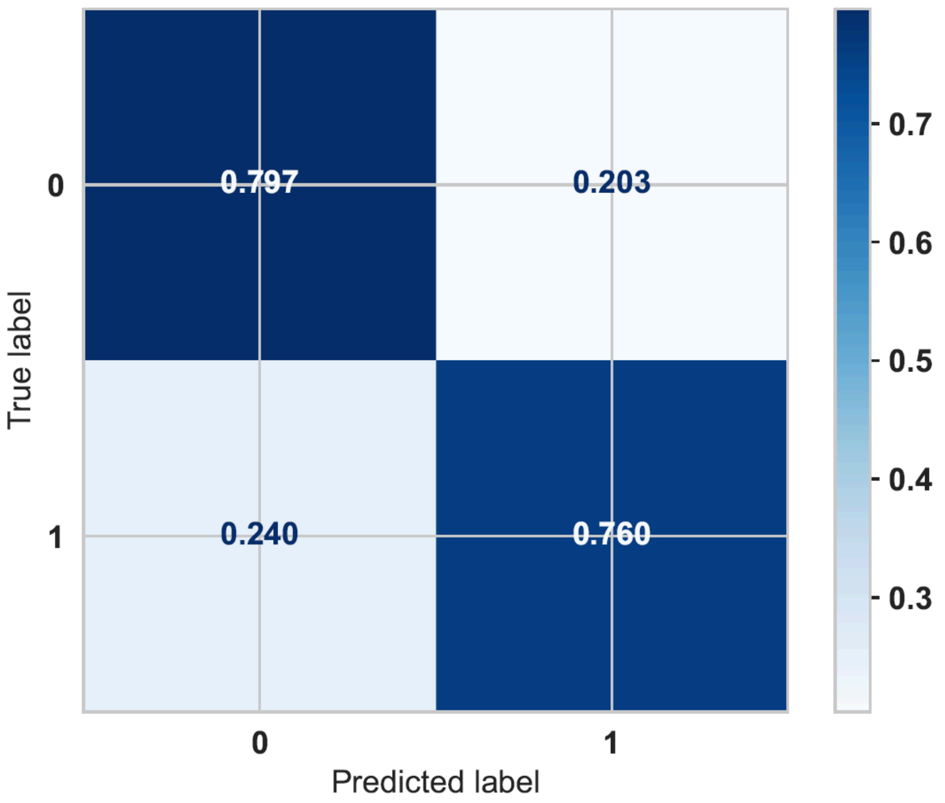

1.1.2.4. DecisionTreeClassifier

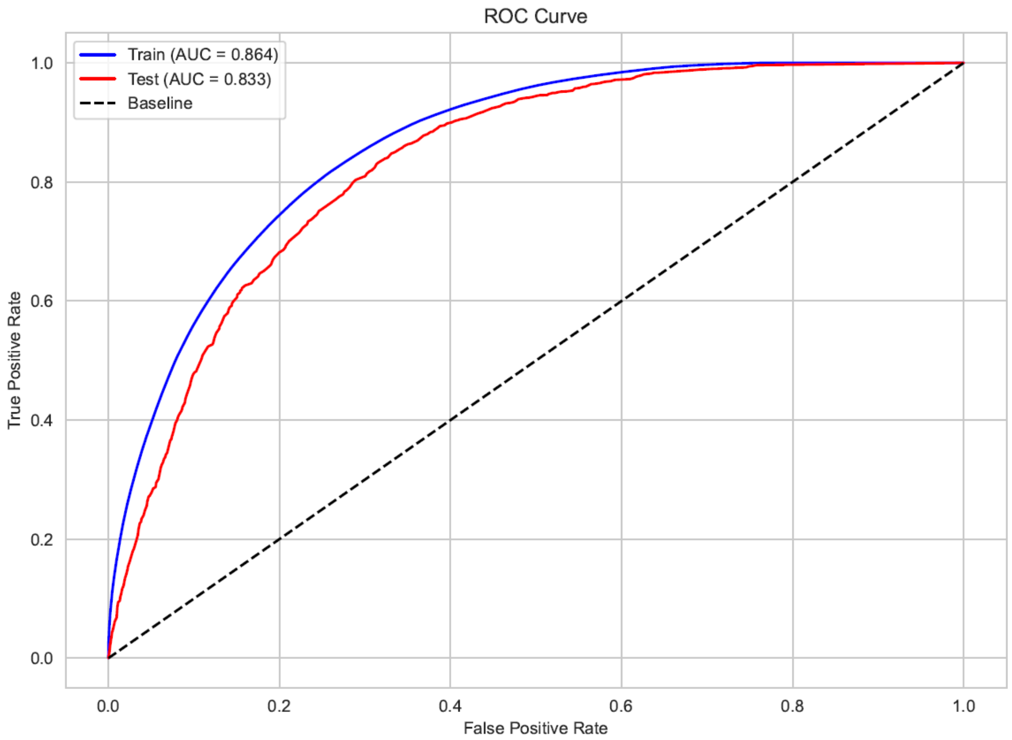

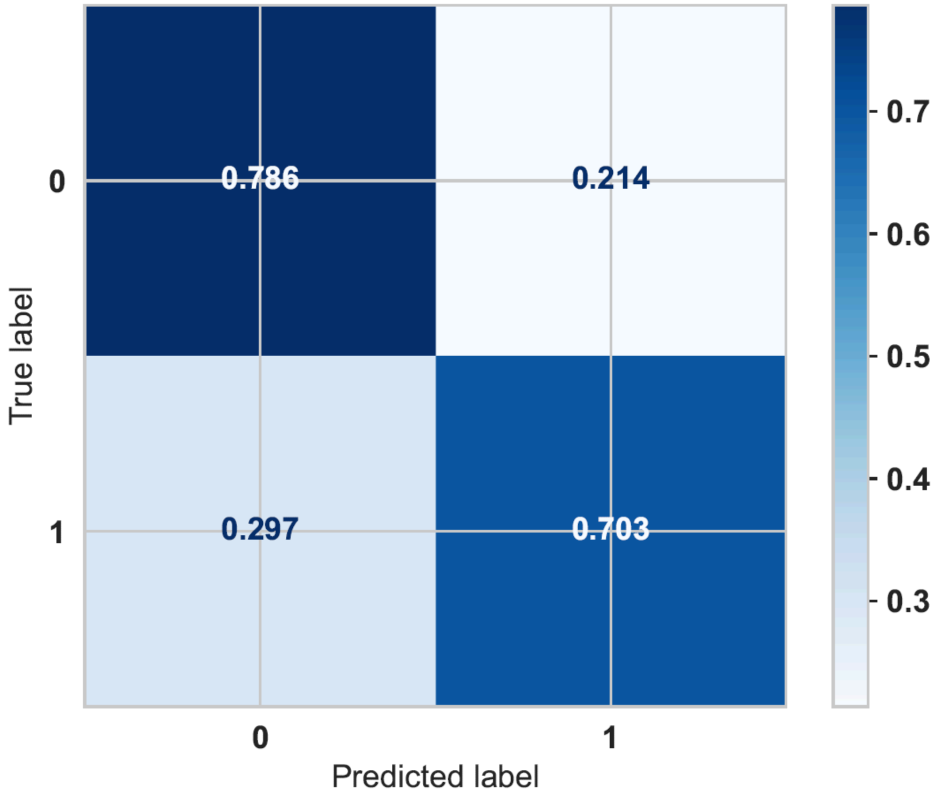

1.1.2.5. ExtraTreesClassifier

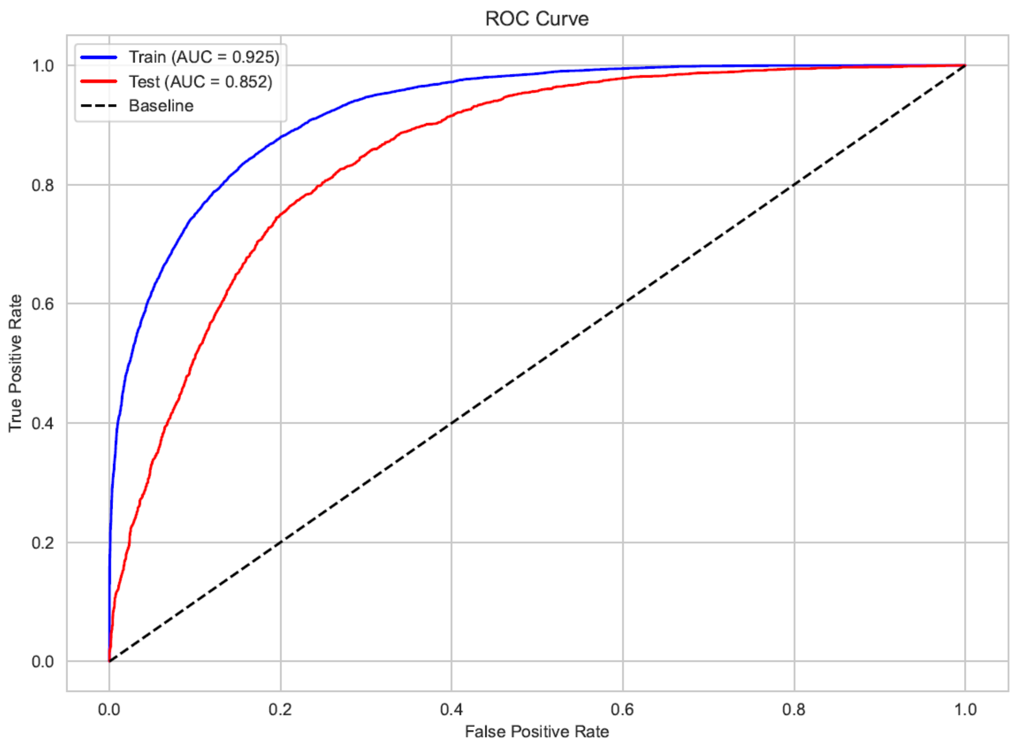

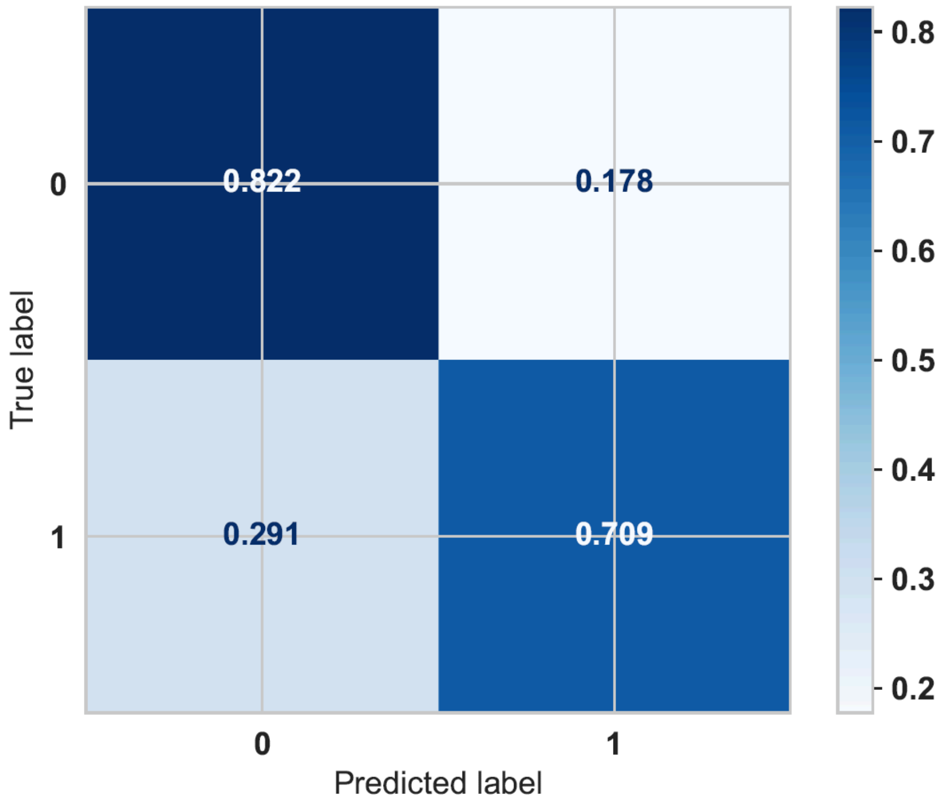

1.1.2.6. GradientBoosting

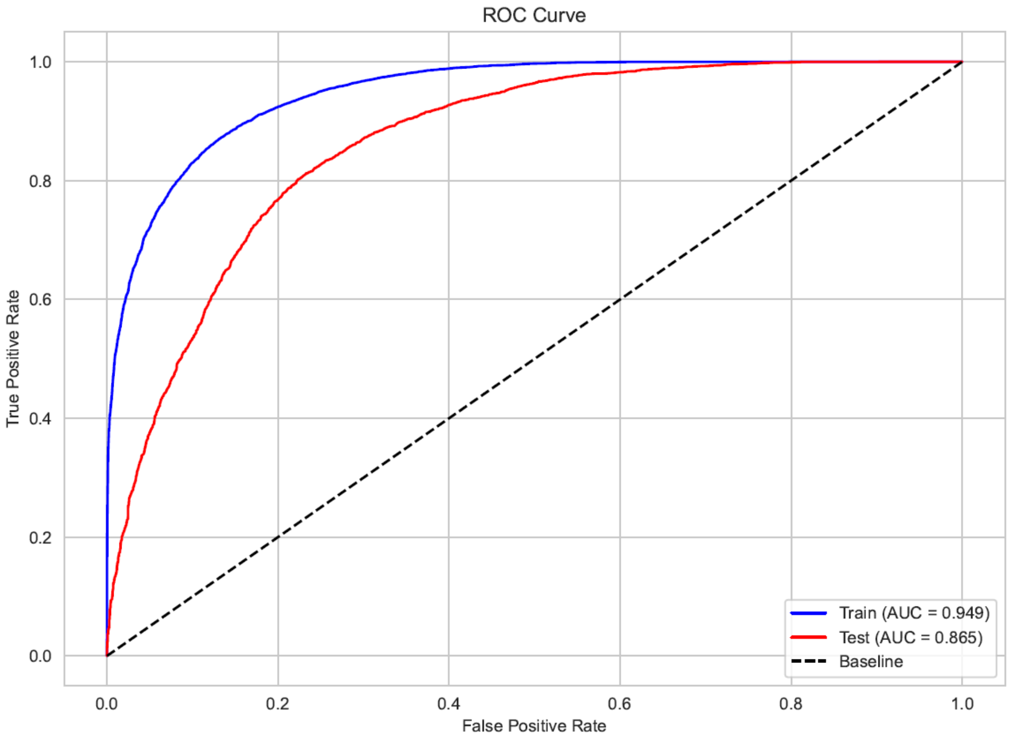
ROC Curve
True Positive Rate
False Positive Rate
0.0
0.2
0.4
0.6
0.8
1.0
Train (AUC = 0.949)
Test (AUC = 0.865)
Baseline

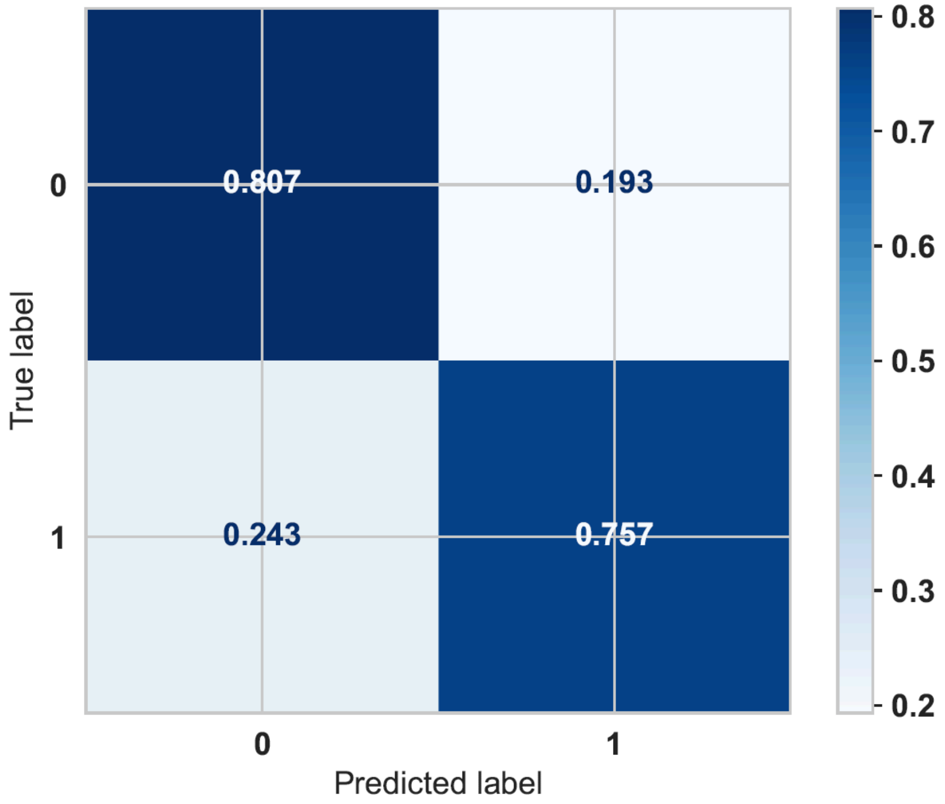
0.807
0.193
0.243
0.757
True label
Predicted label
0
1
0.8
0.7
0.6
0.5
0.4
0.3
0.2

1.1.2.7. KNN

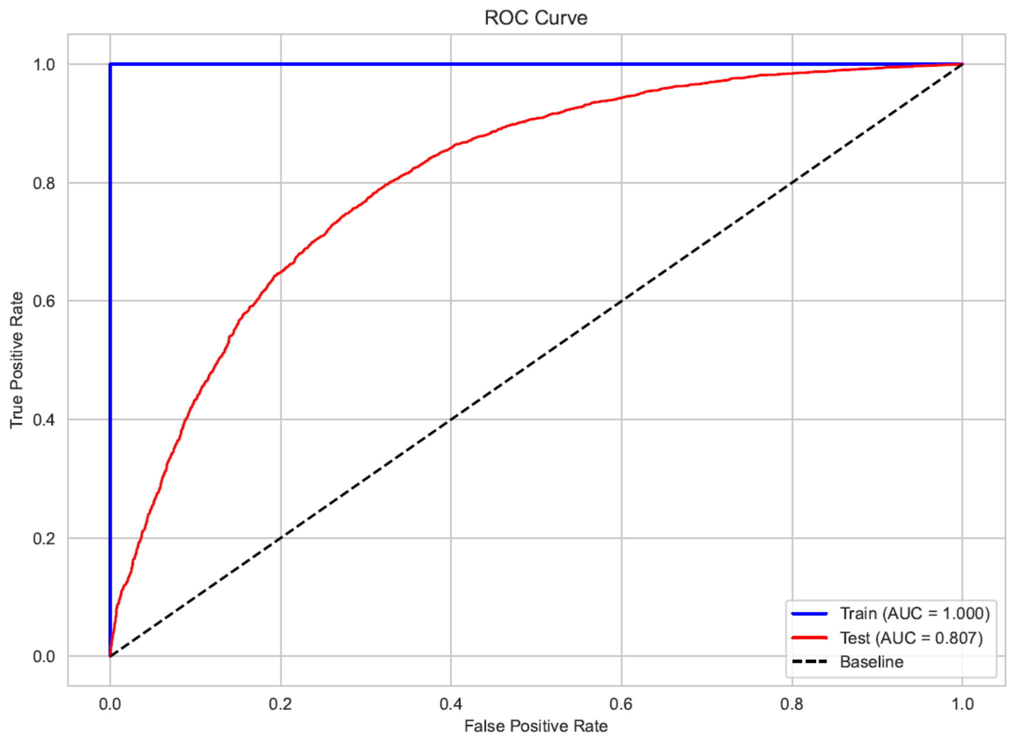

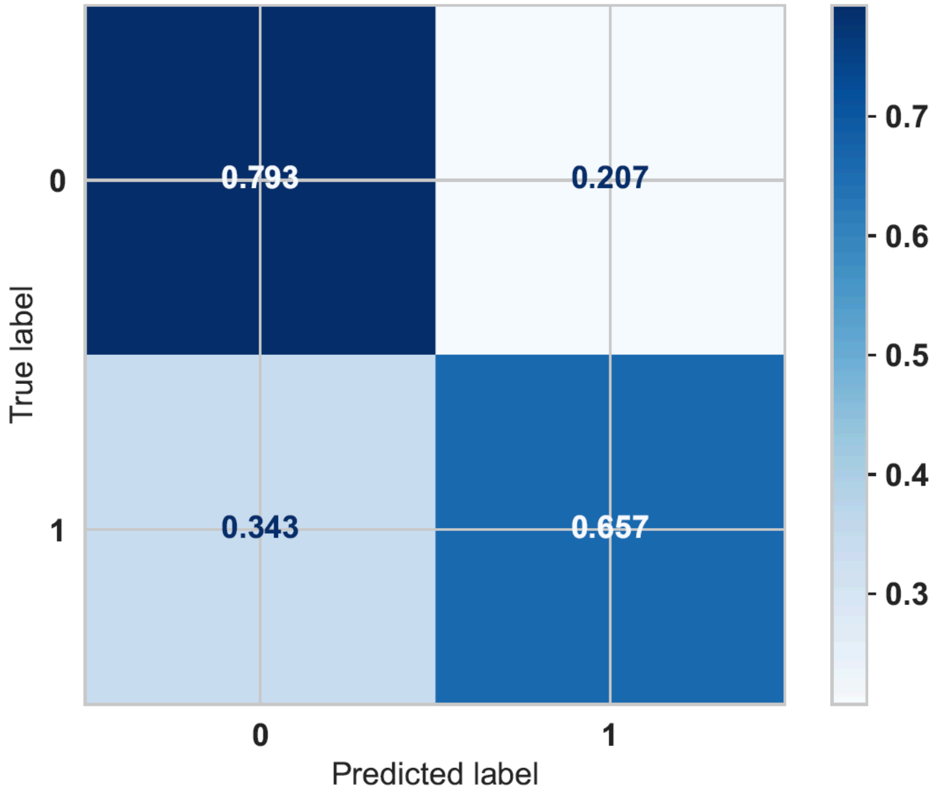

1.1.2.8. lightgbm

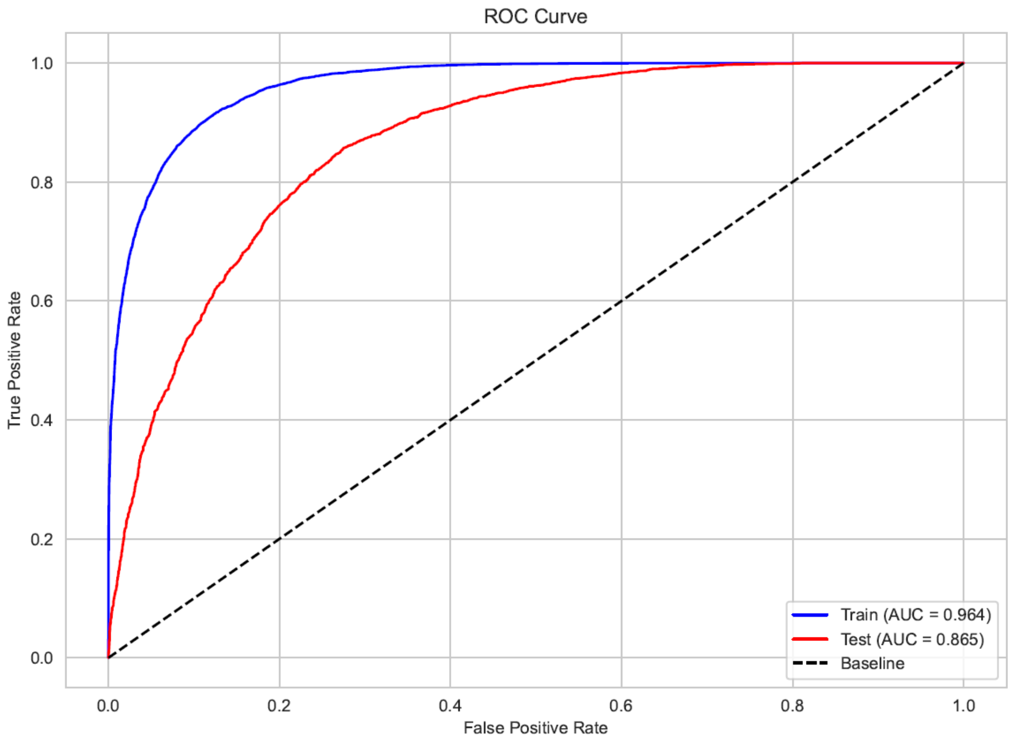

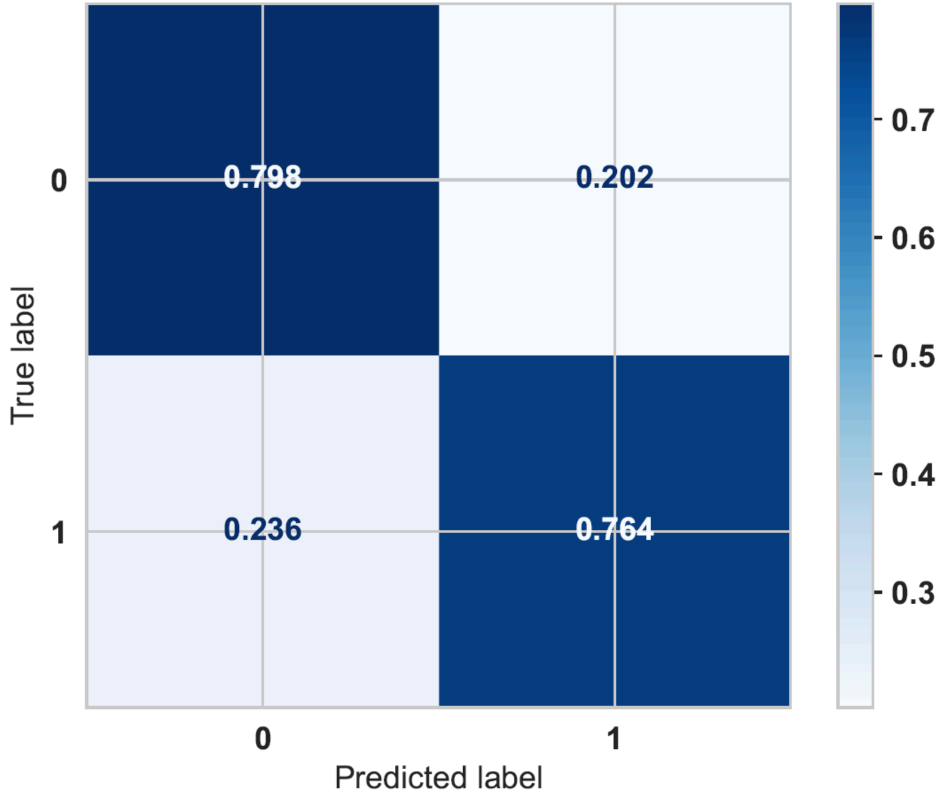

1.1.3. Alive 5 year

1.1.3.1. Random Forest

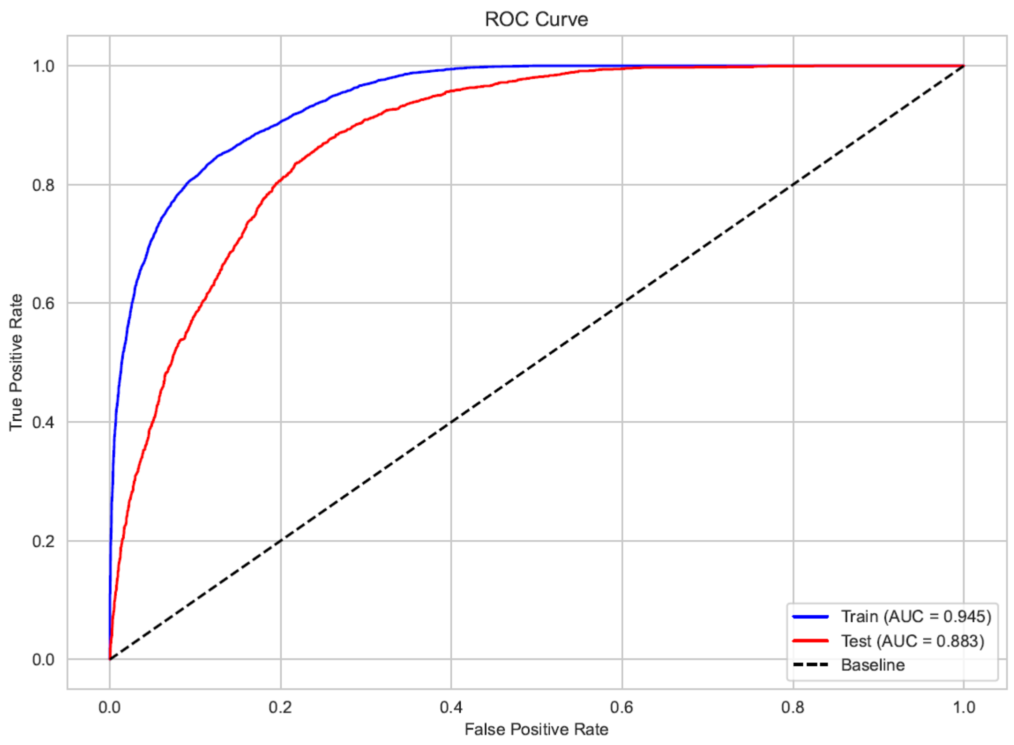

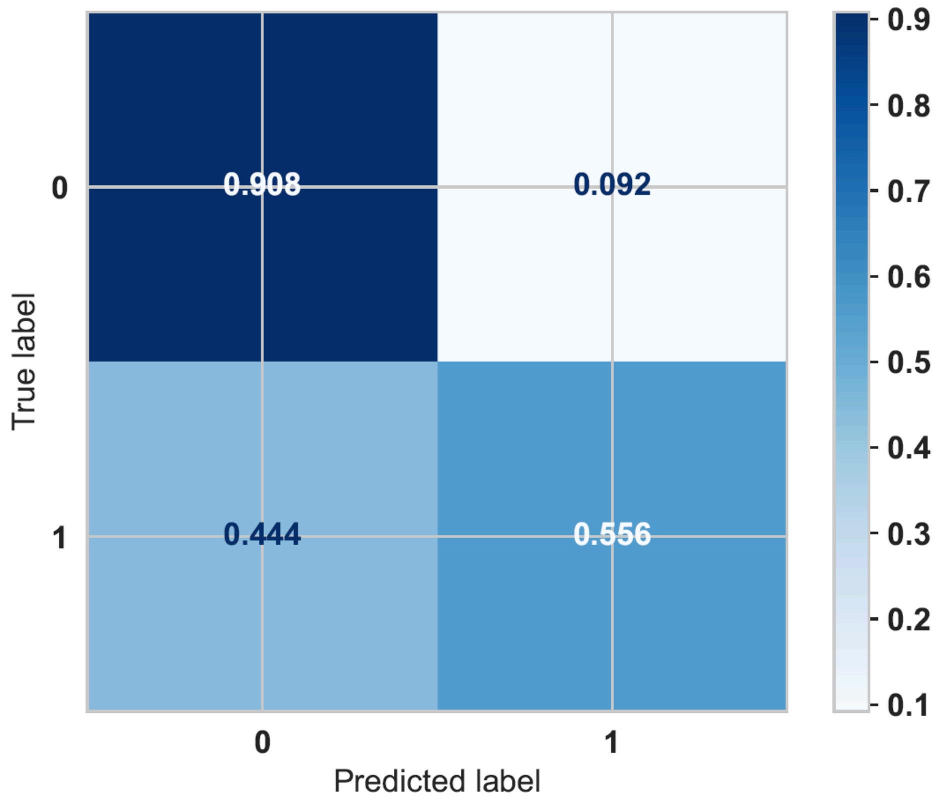

1.1.3.2. XGBoost

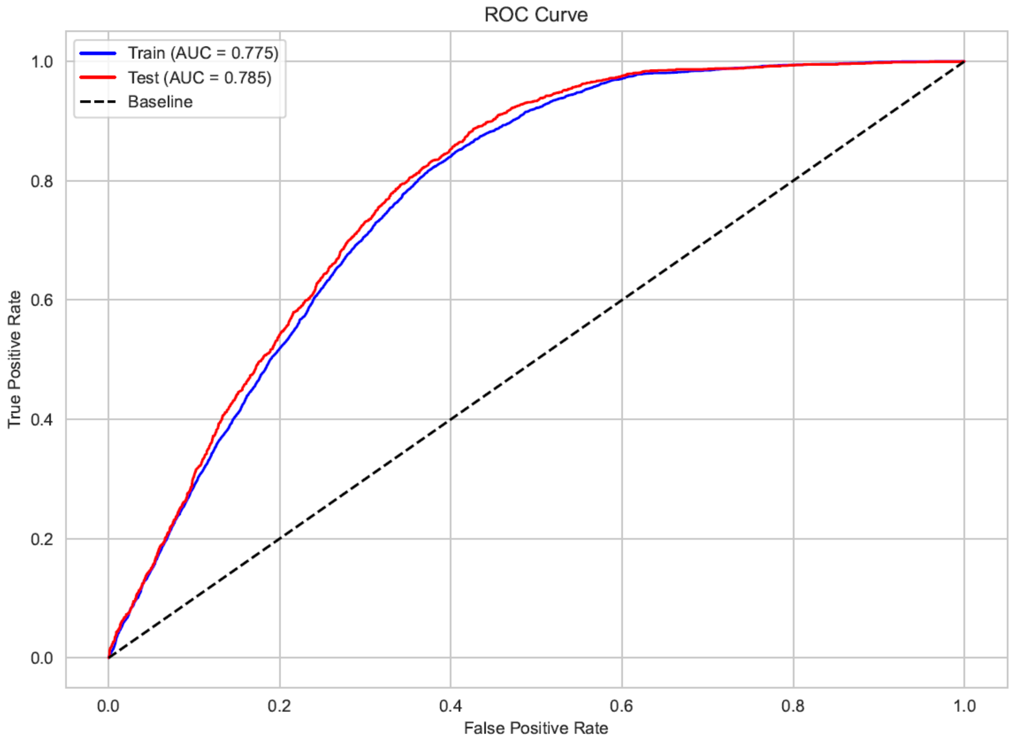

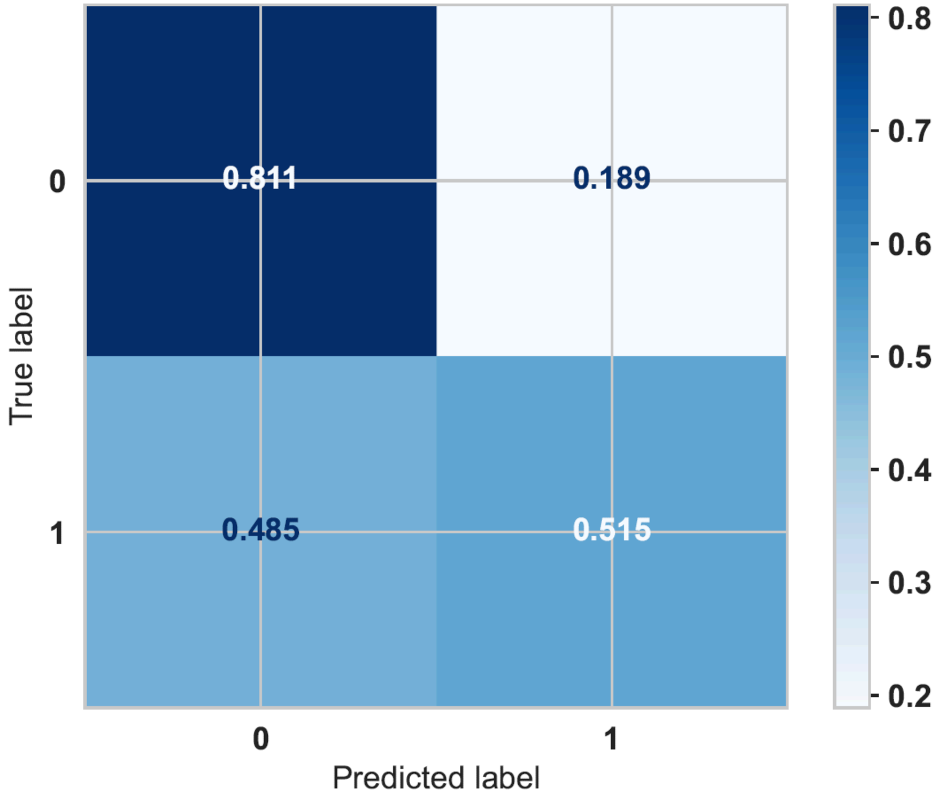

1.1.3.3. CatBoost

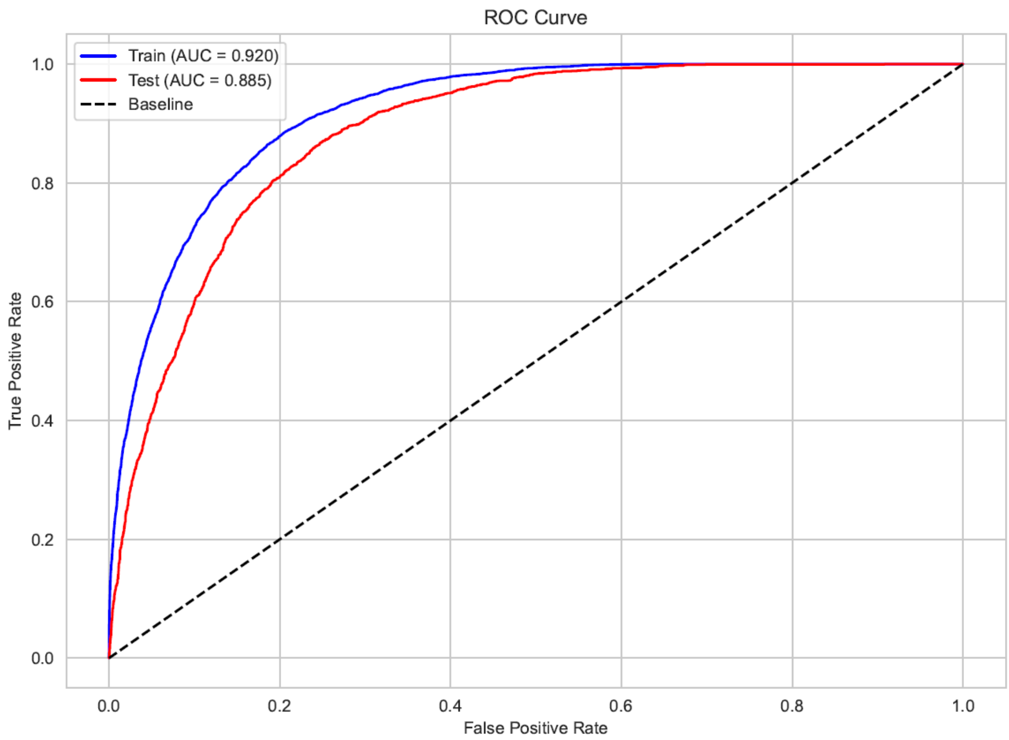

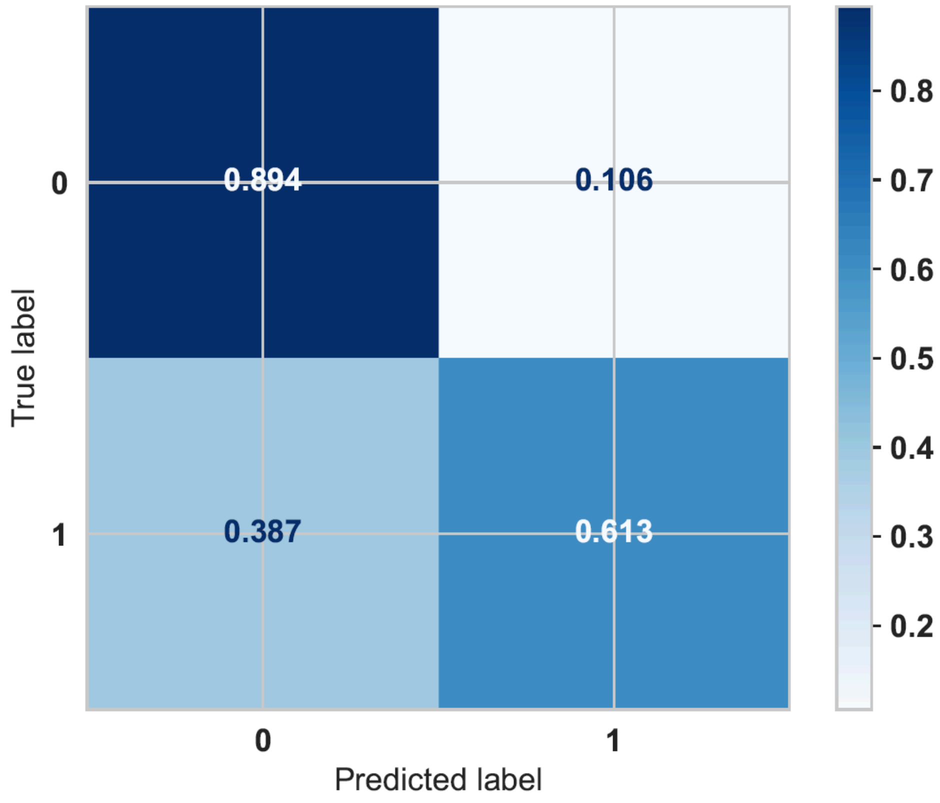

1.1.3.4. DecisionTreeClassifier

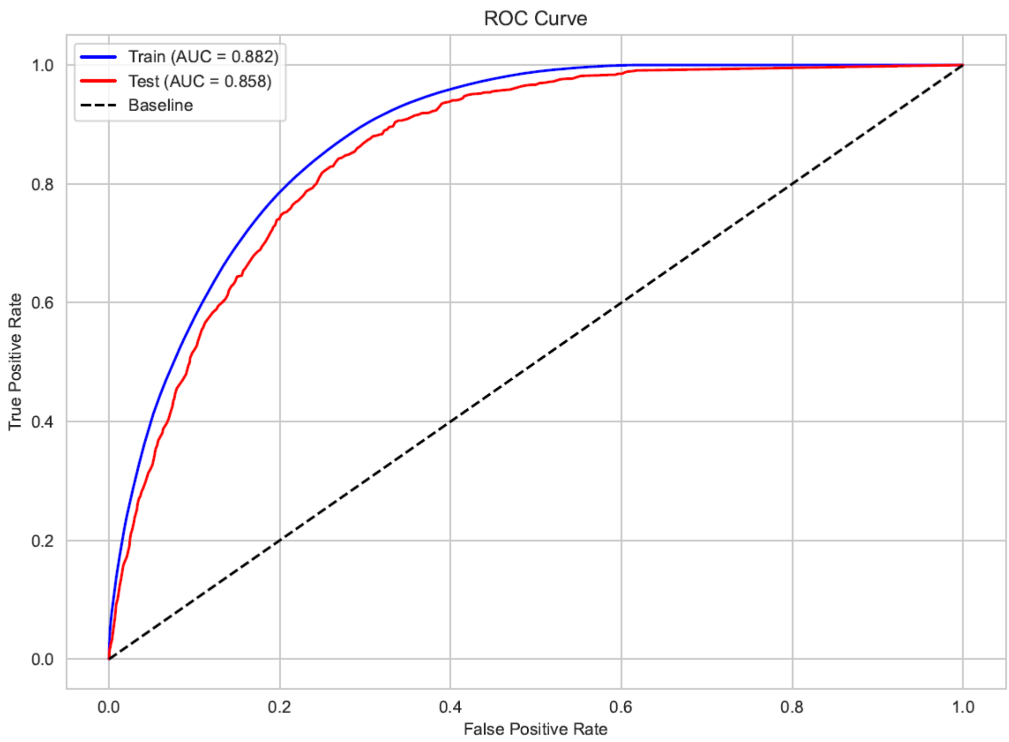

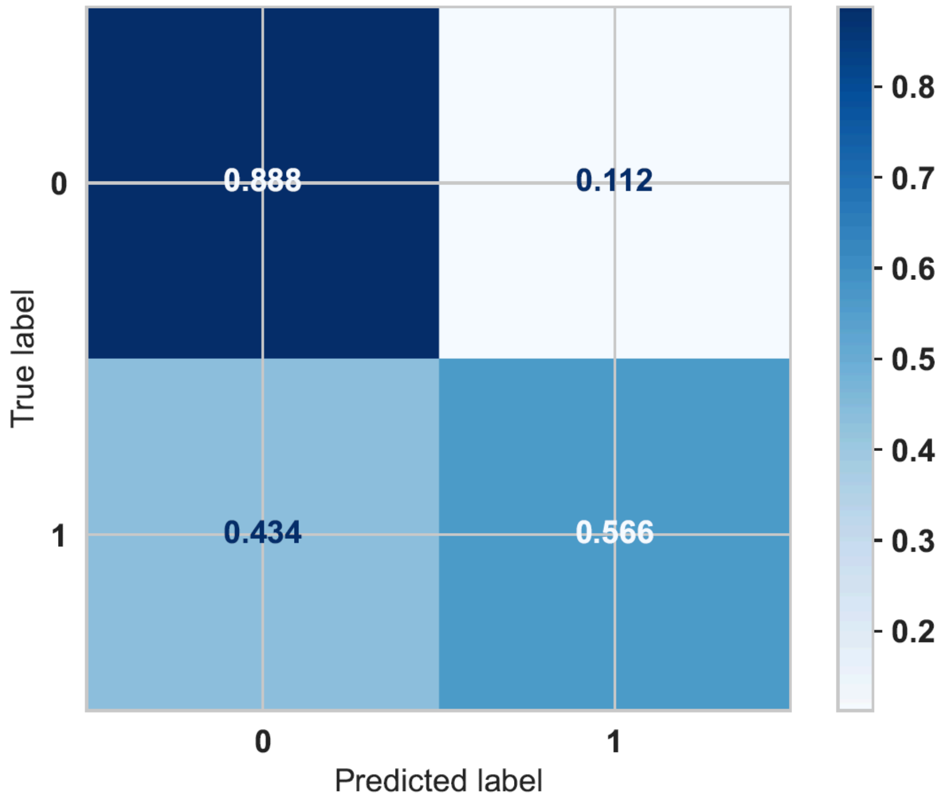

1.1.3.5. ExtraTreesClassifier

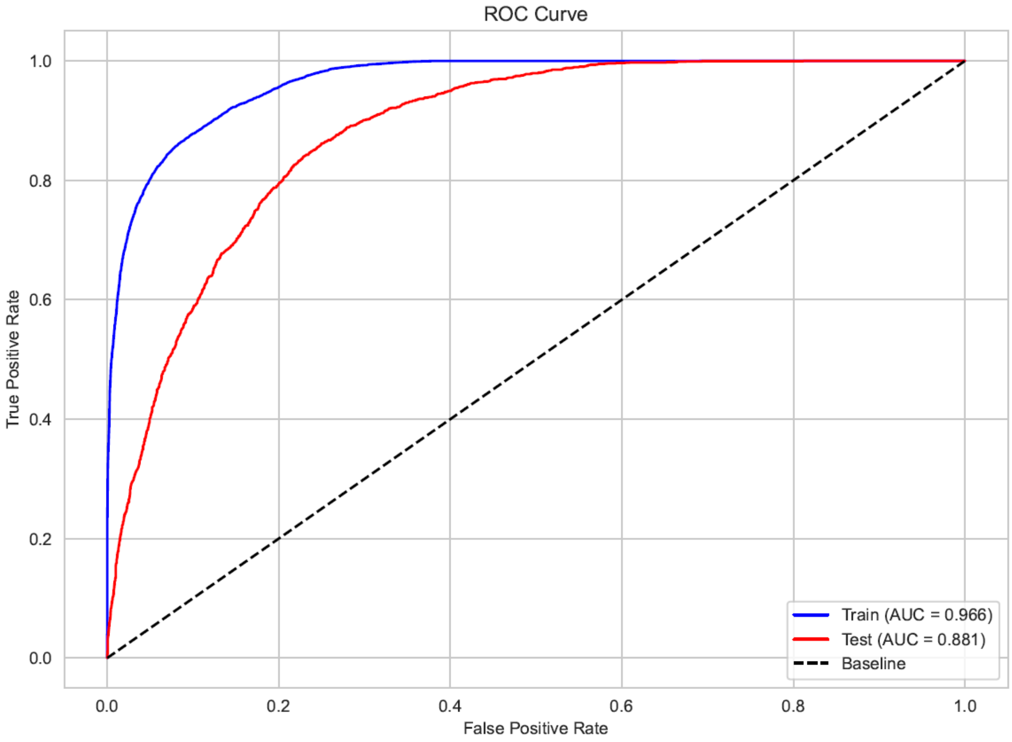

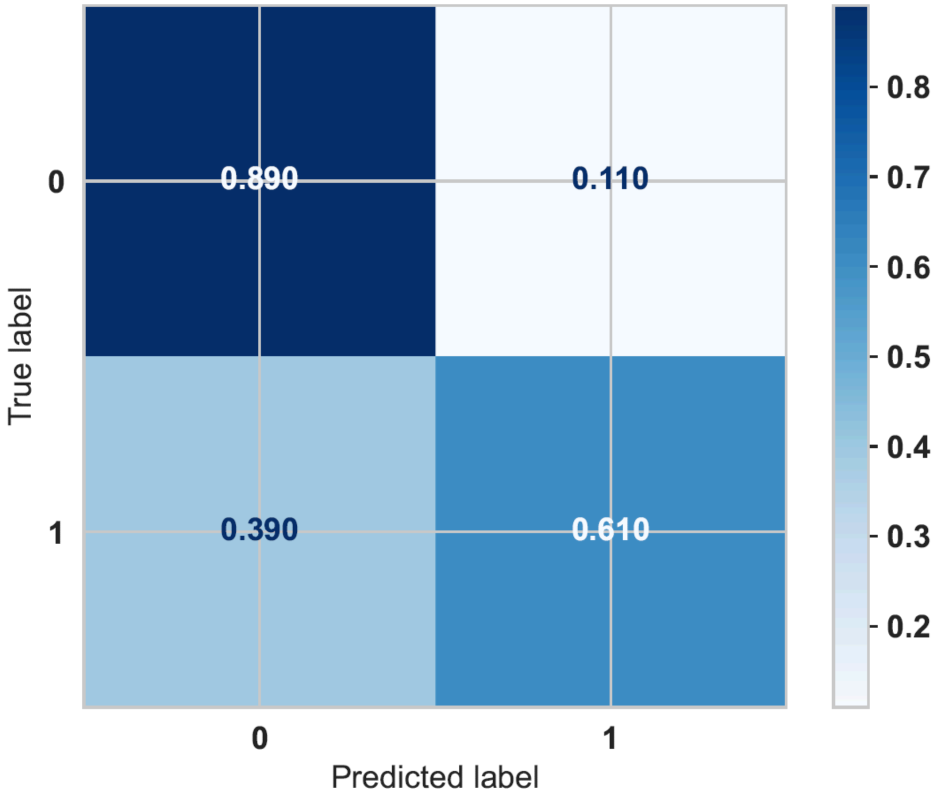

1.1.3.6. GradientBoosting

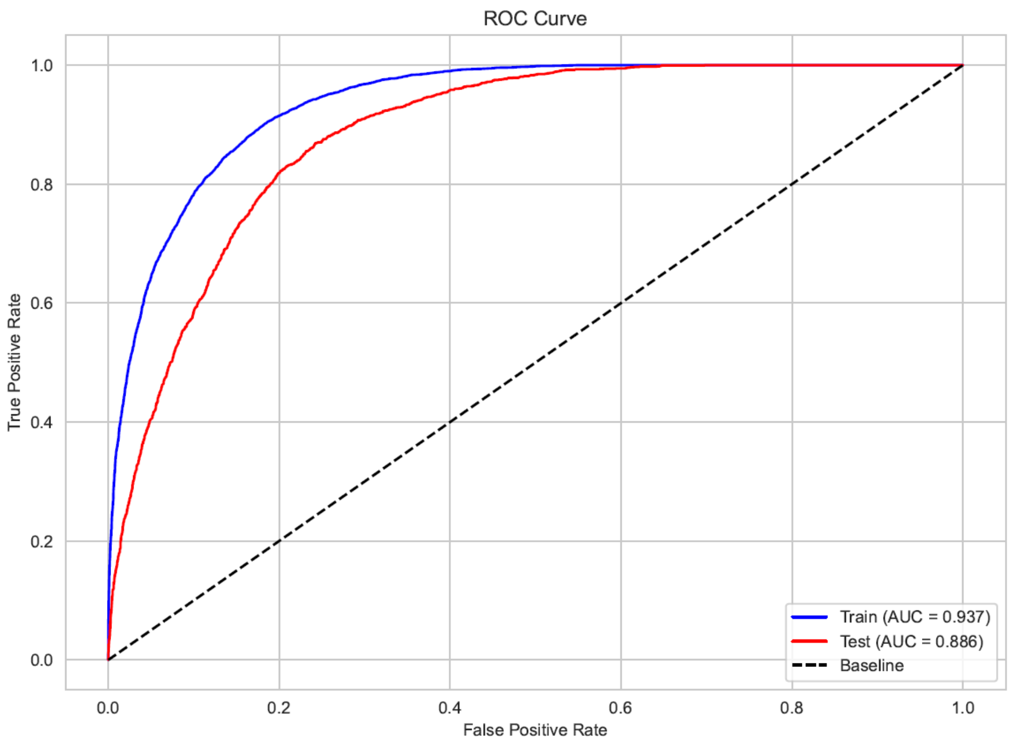
ROC Curve
True Positive Rate
False Positive Rate
0.0
0.2
0.4
0.6
0.8
1.0
Train (AUC = 0.937)
Test (AUC = 0.886)
Baseline

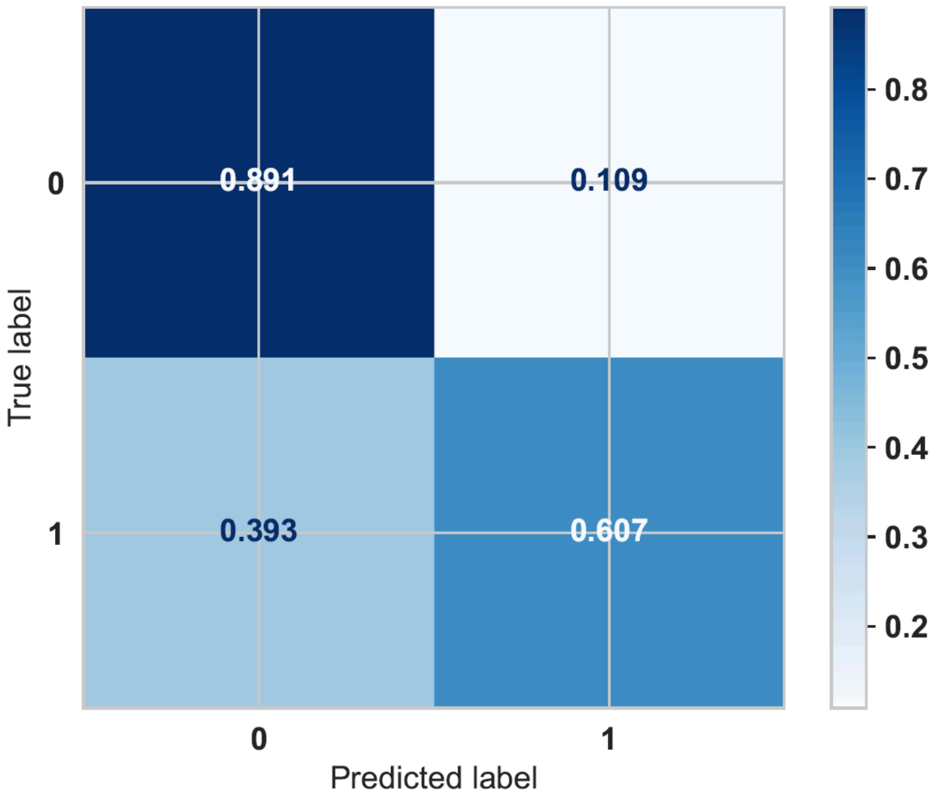
0.891
0.109
0.393
0.607
True label
Predicted label
0
1
0.8
0.7
0.6
0.5
0.4
0.3
0.2

1.1.3.7. KNN

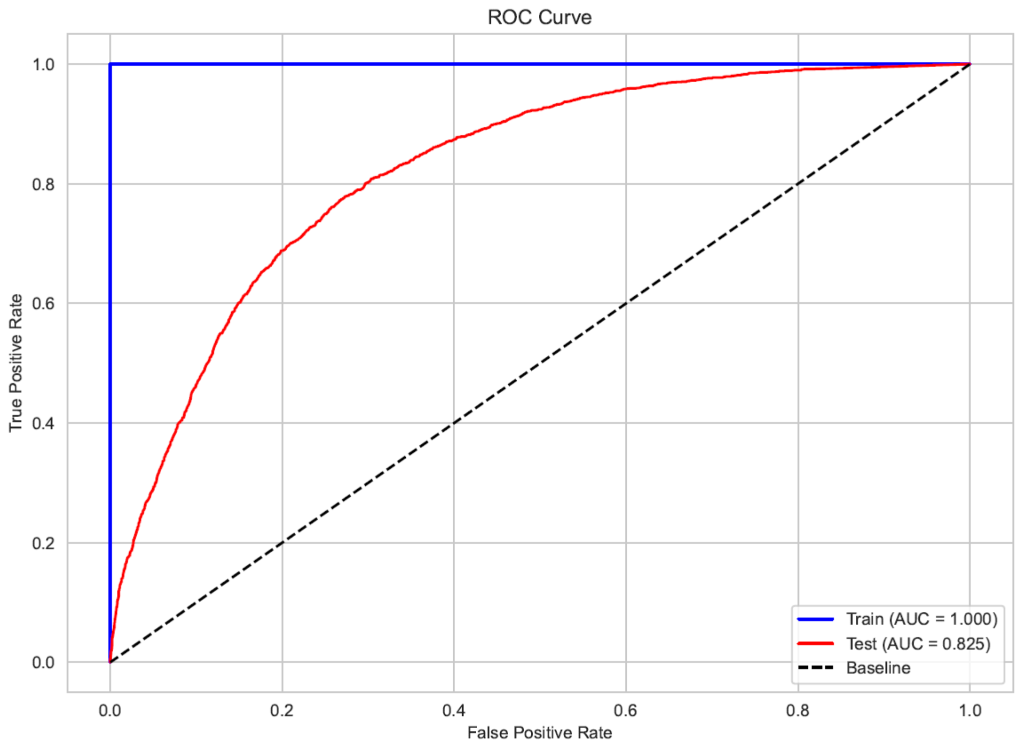

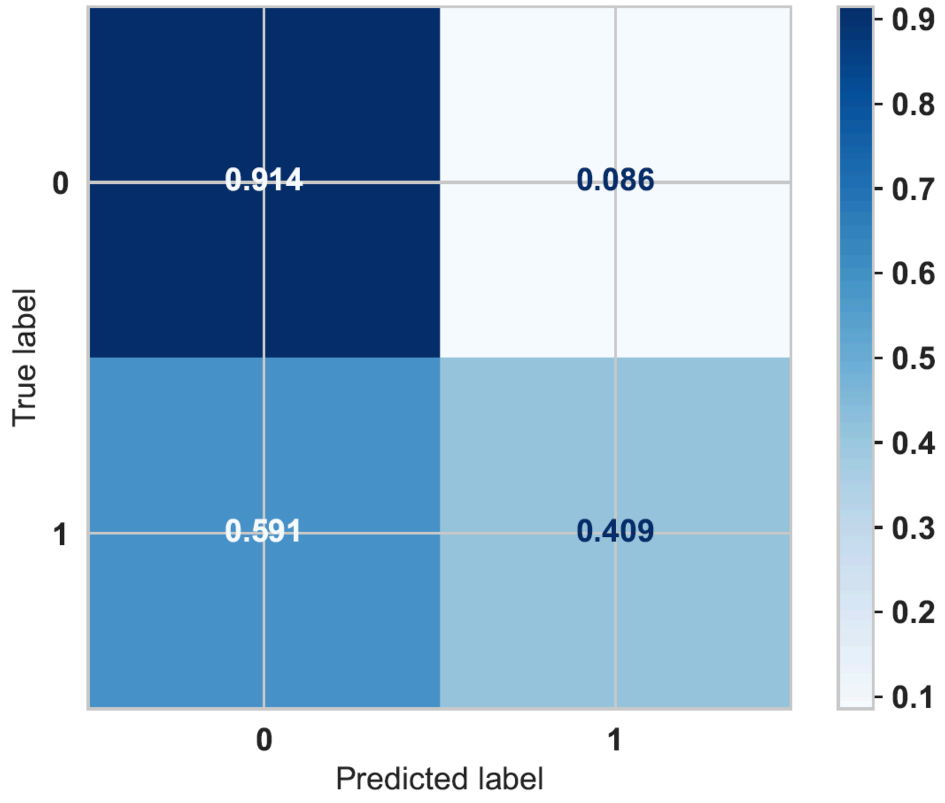

1.1.3.8. lightgbm

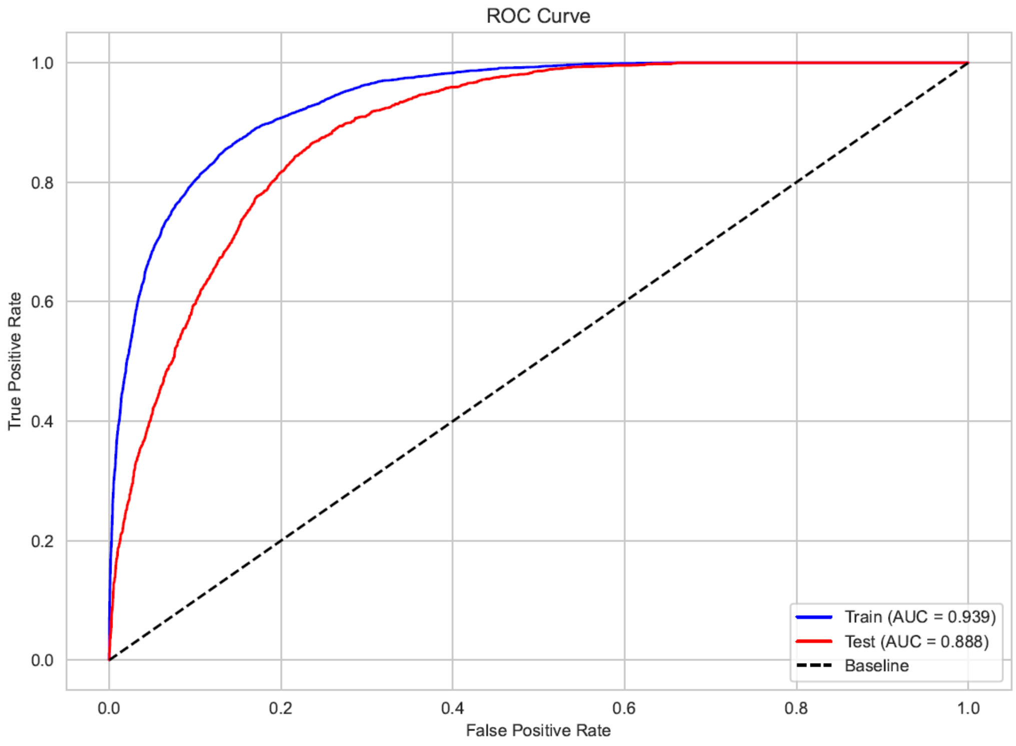

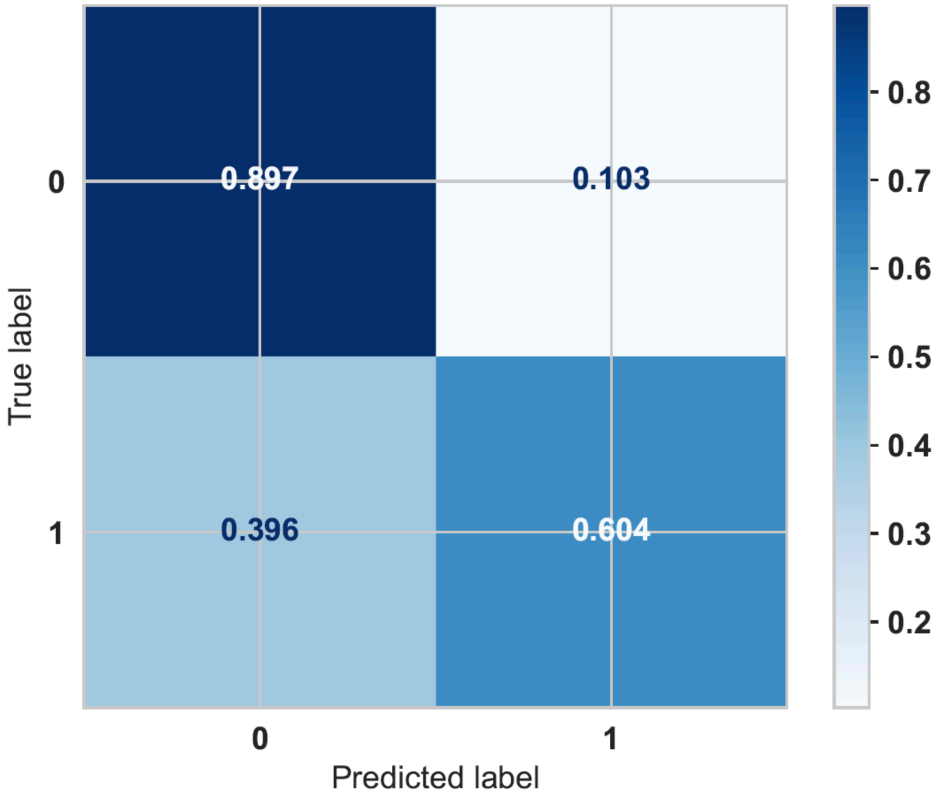

1.1.4. Cancer death

1.1.4.1. Random Forest

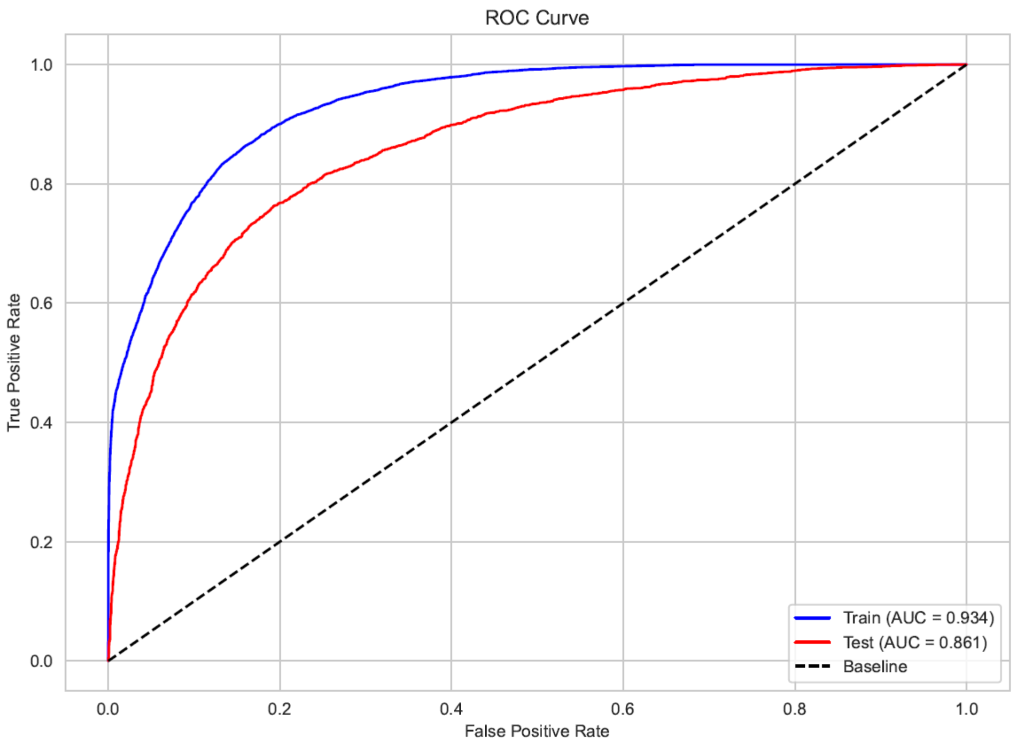

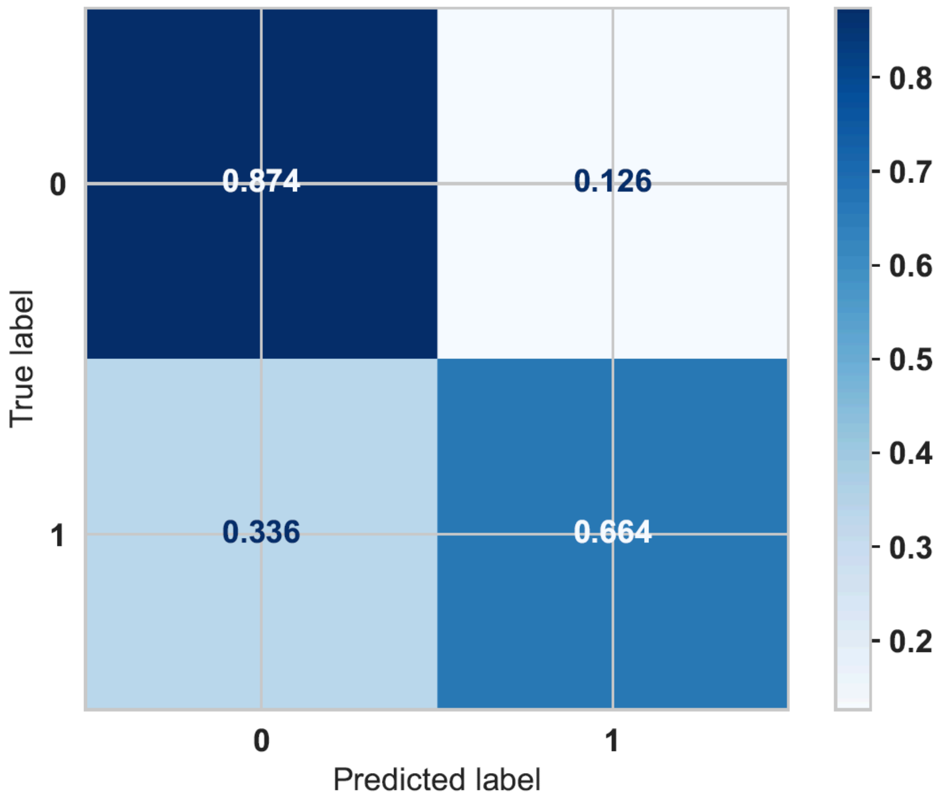

1.1.4.2. XGBoost

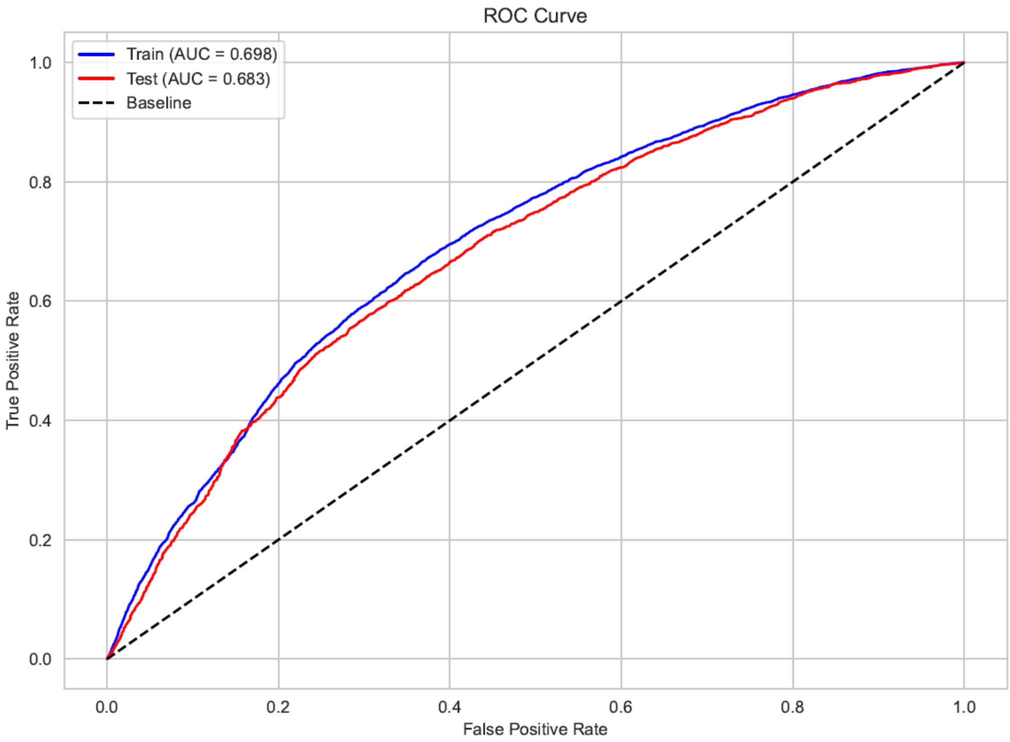

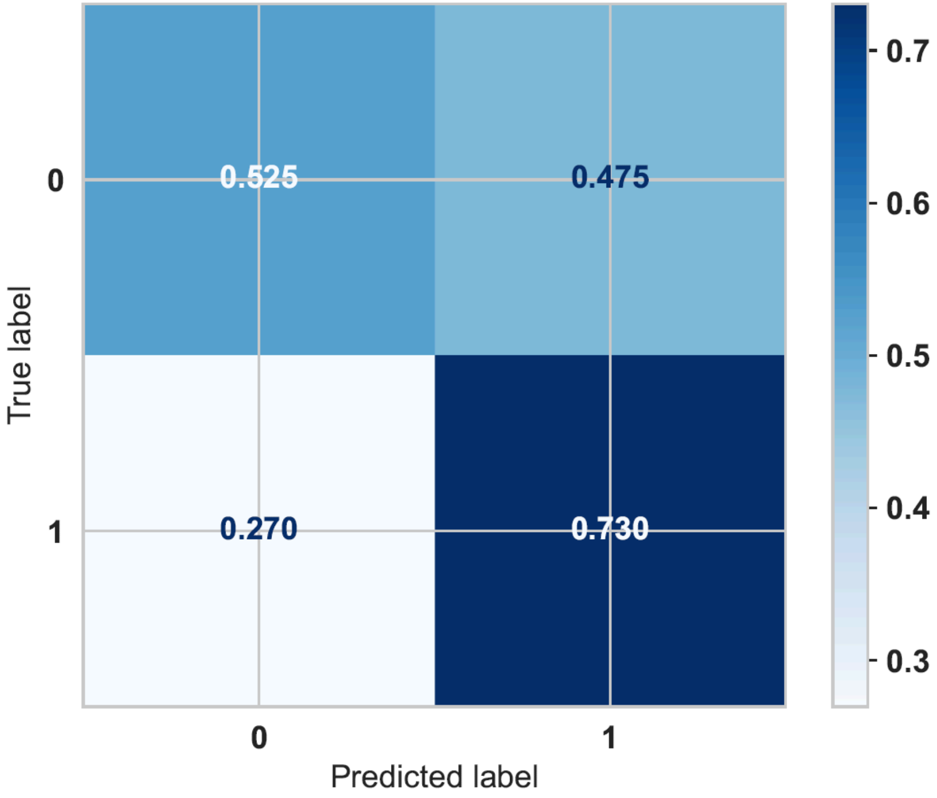

1.1.4.3. CatBoost

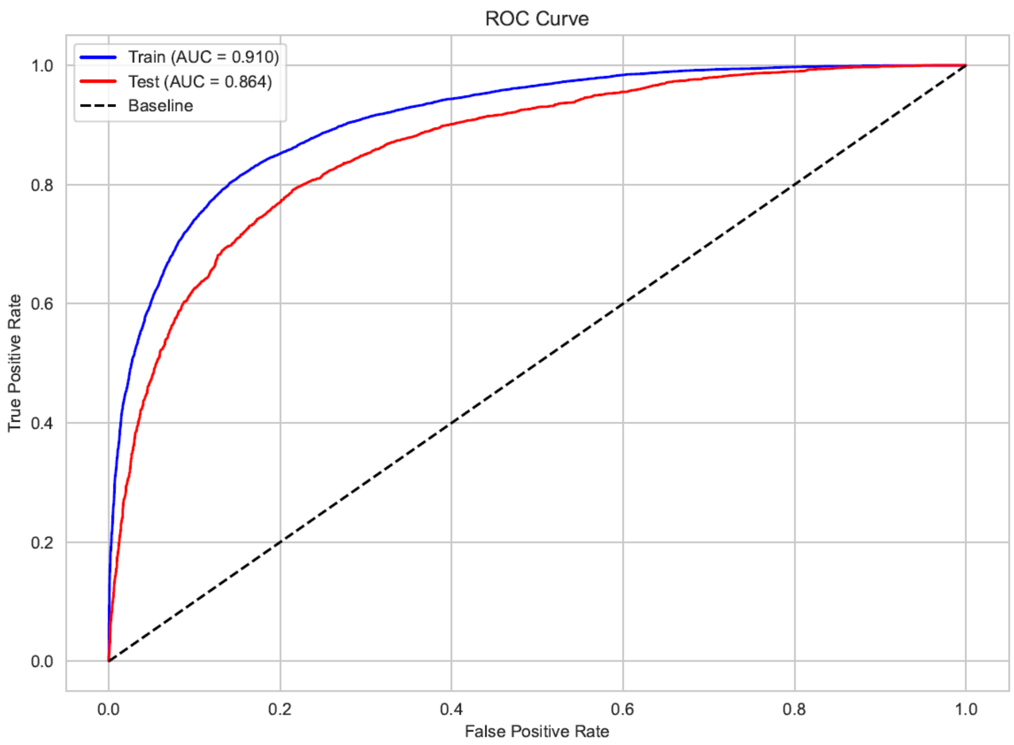

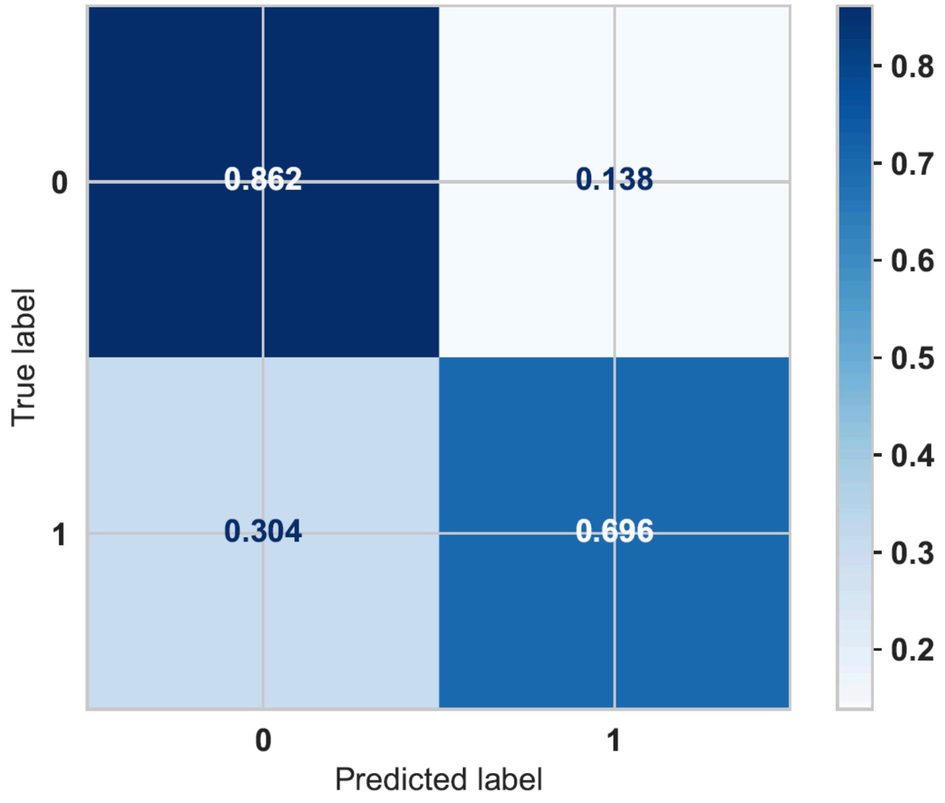

1.1.4.4. DecisionTreeClassifier

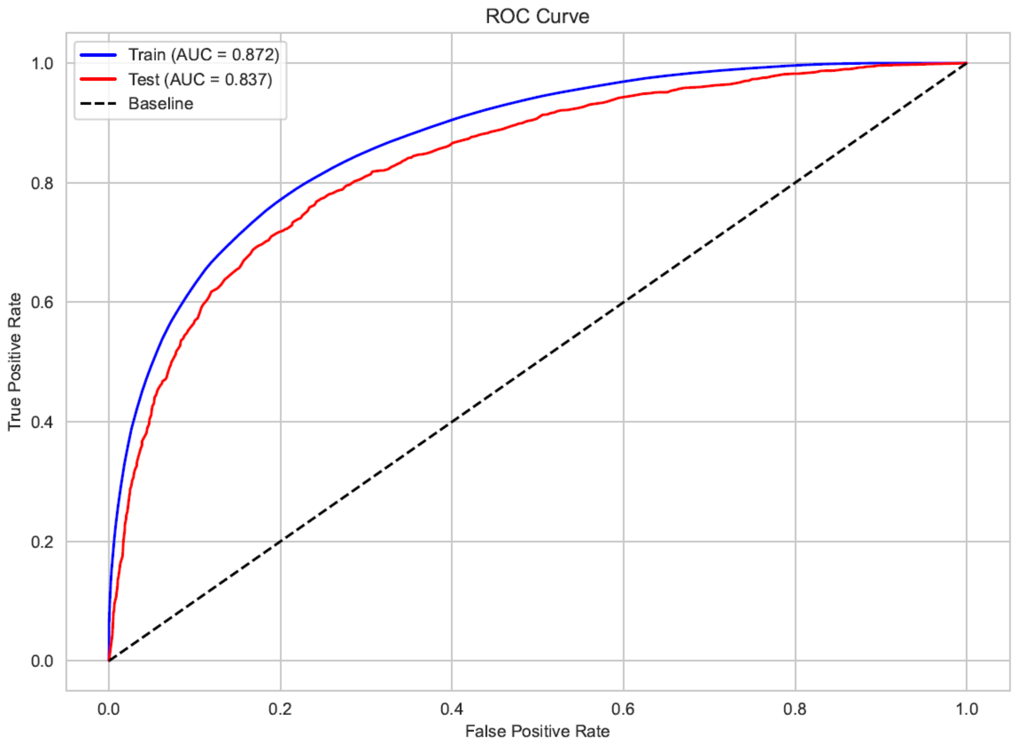

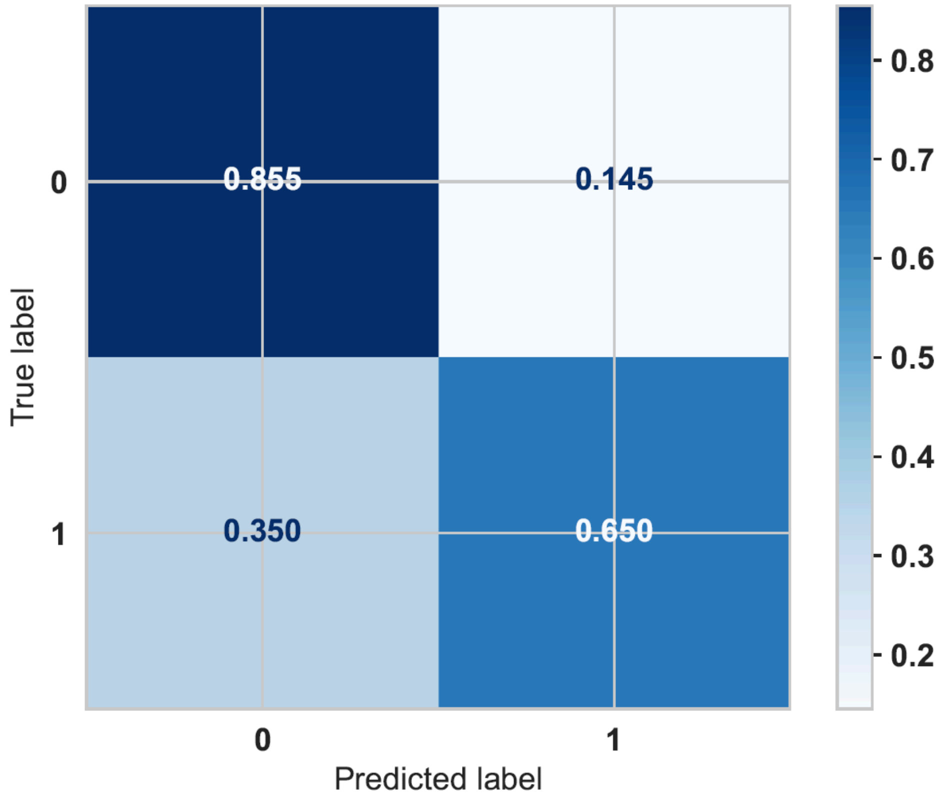

1.1.4.5. ExtraTreesClassifier

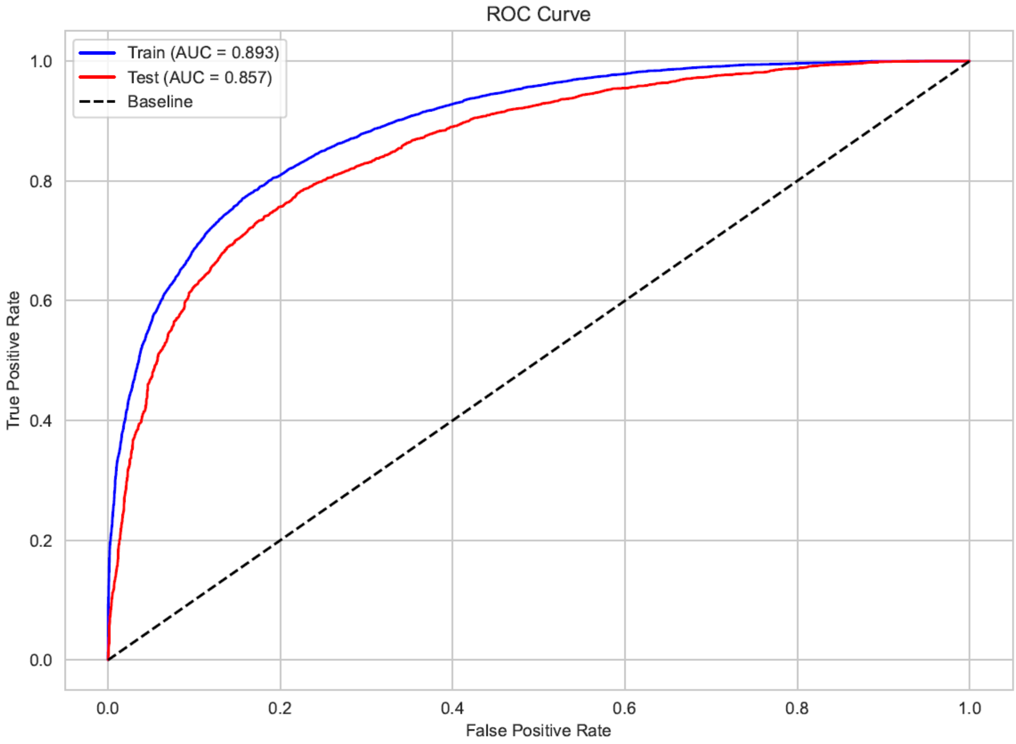

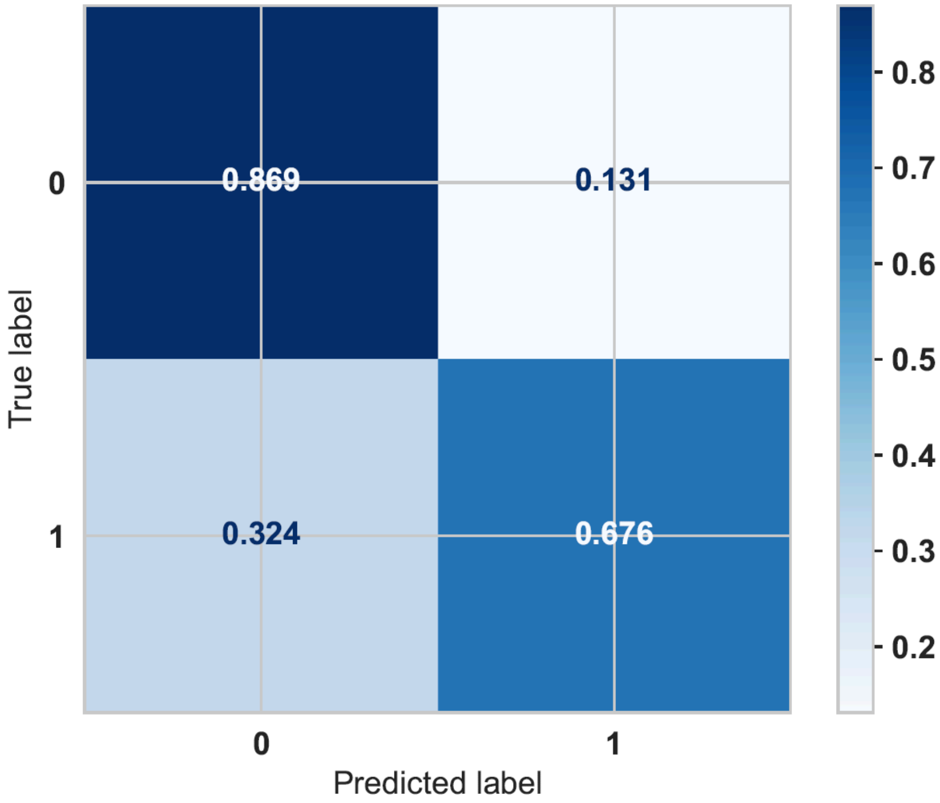

1.1.4.6. GradientBoosting

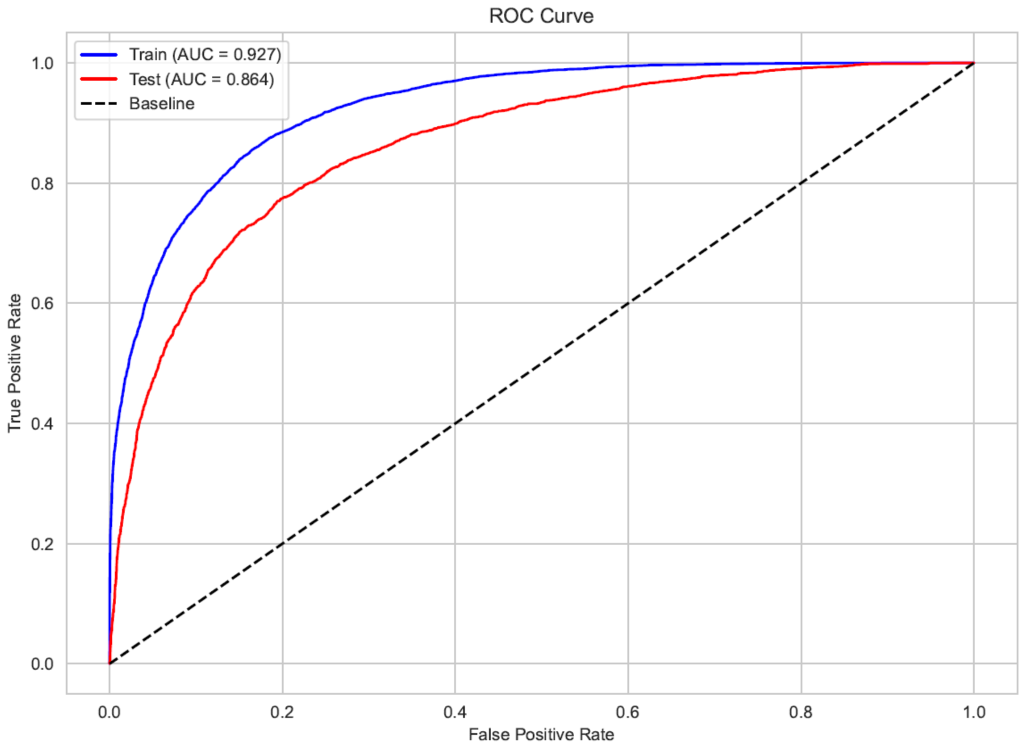
ROC Curve
Train (AUC = 0.927)
Test (AUC = 0.864)
Baseline
True Positive Rate
False Positive Rate
0.0
0.2
0.4
0.6
0.8
1.0

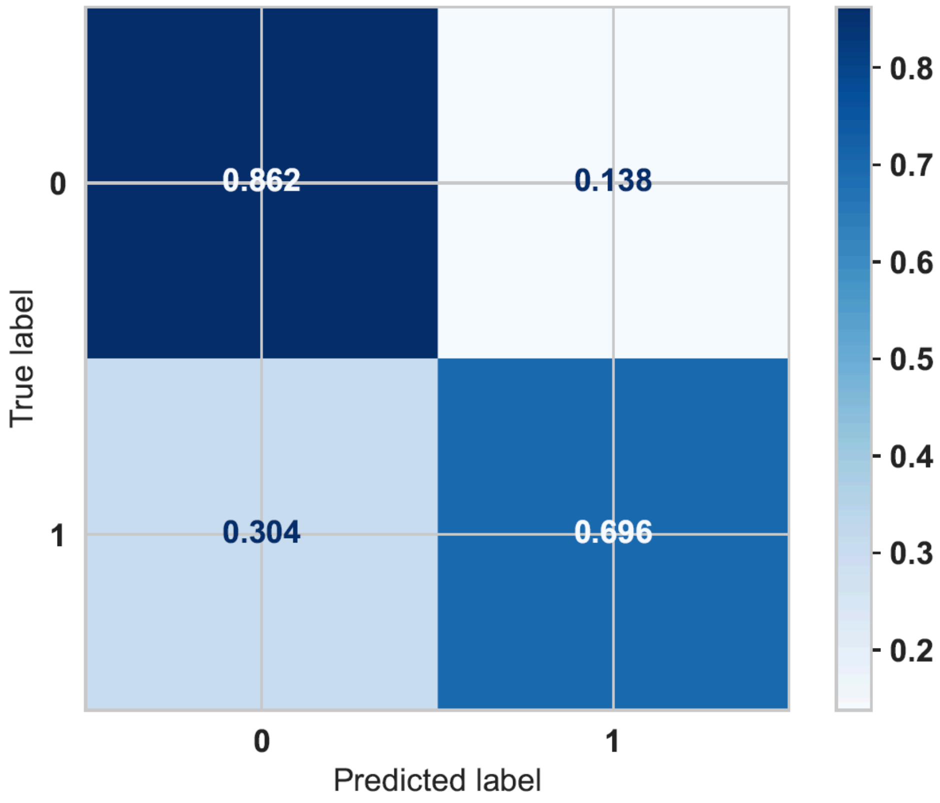
0.862
0.138
0.304
0.696
True label
Predicted label
0
1
0.8
0.7
0.6
0.5
0.4
0.3
0.2

1.1.4.7. KNN

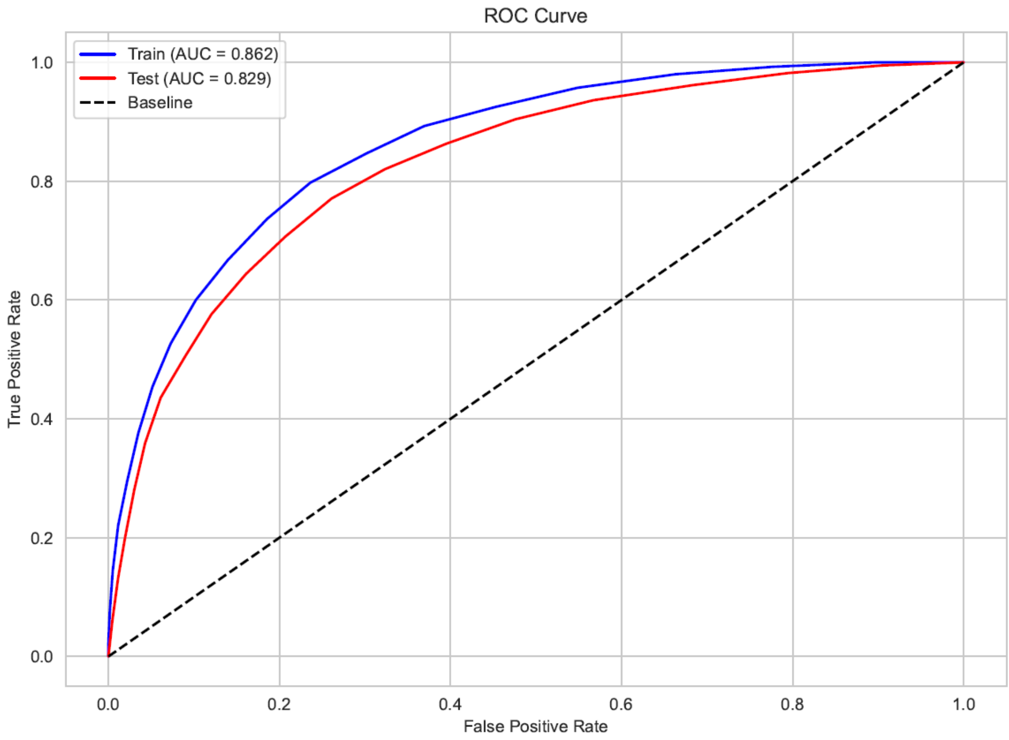

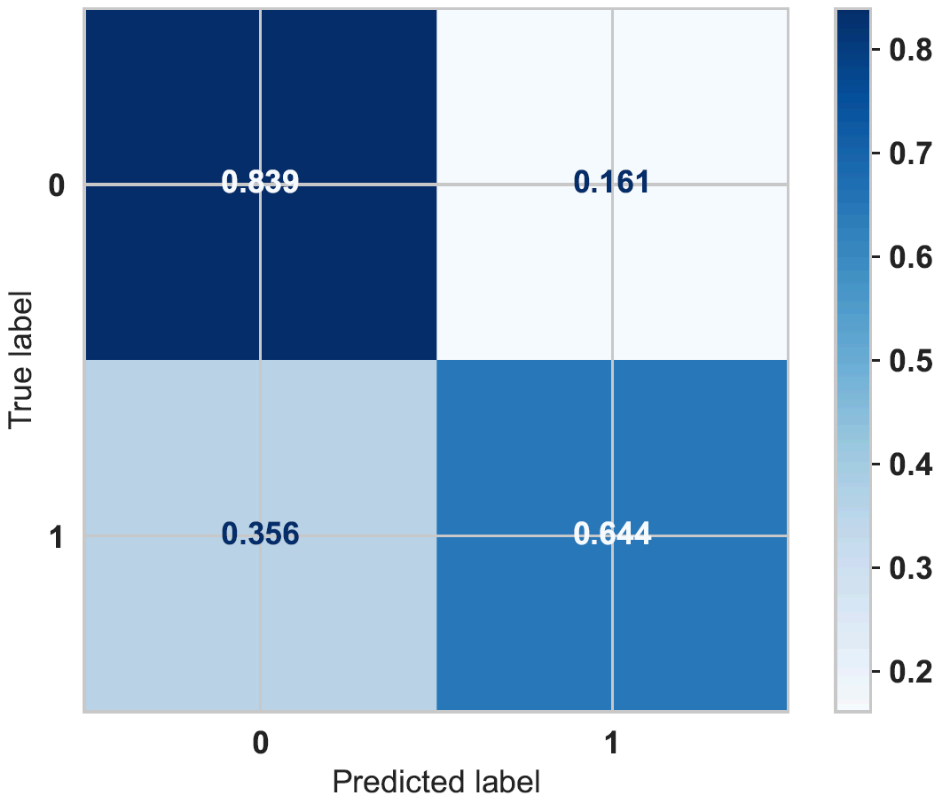

1.1.4.8. lightgbm

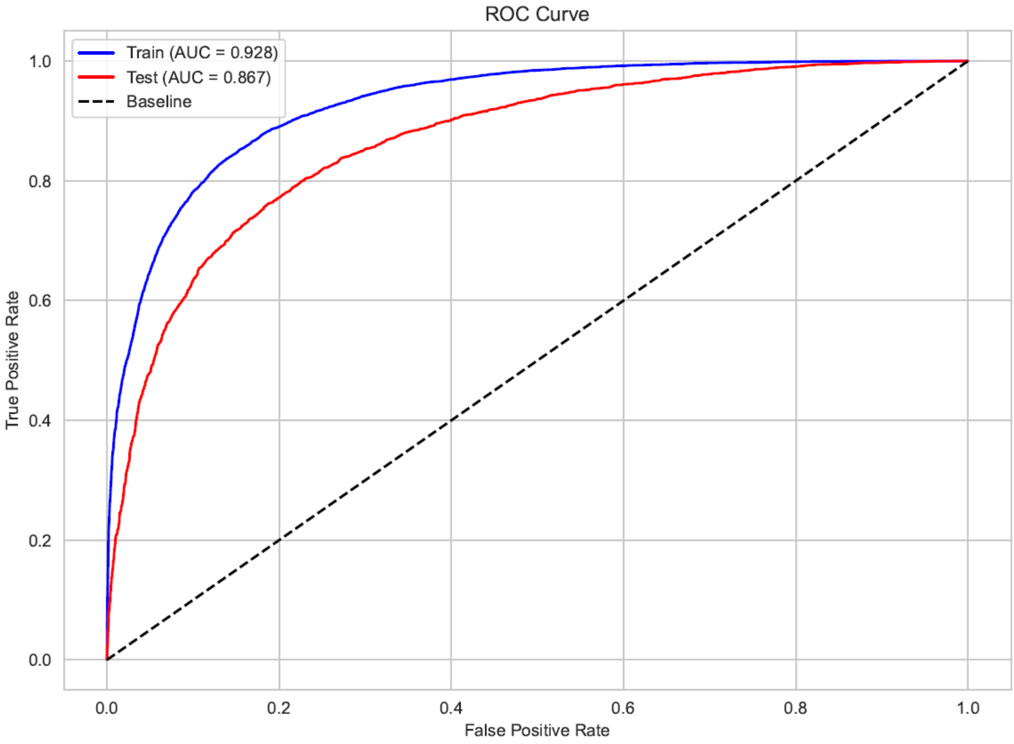

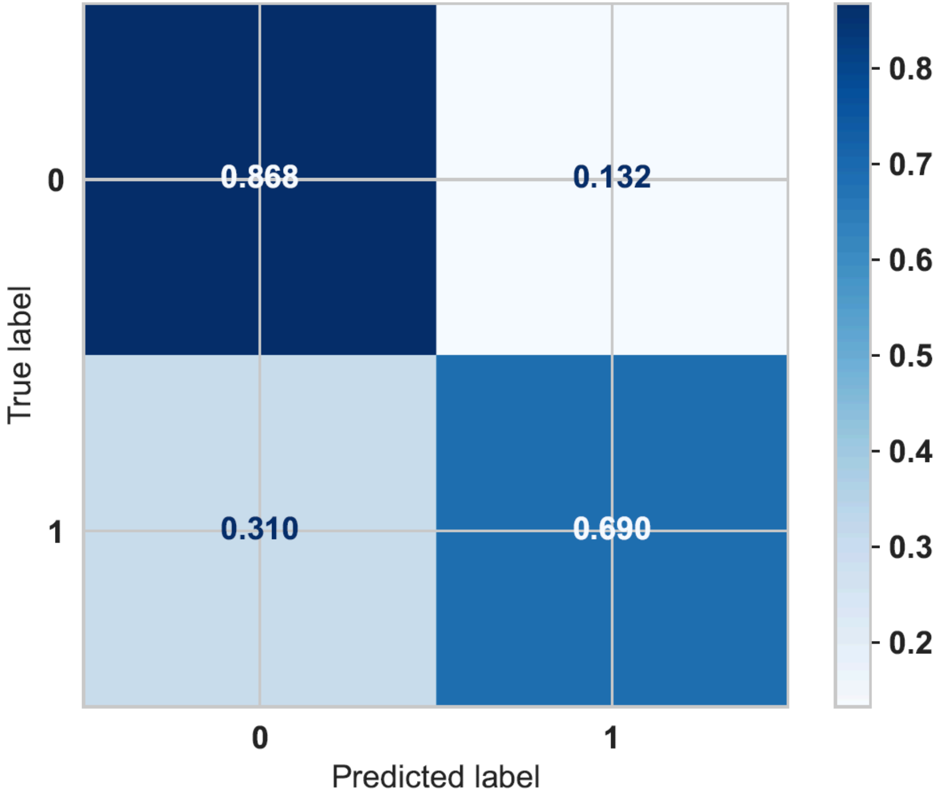

1.1.5. Overall death

1.1.5.1. Random Forest

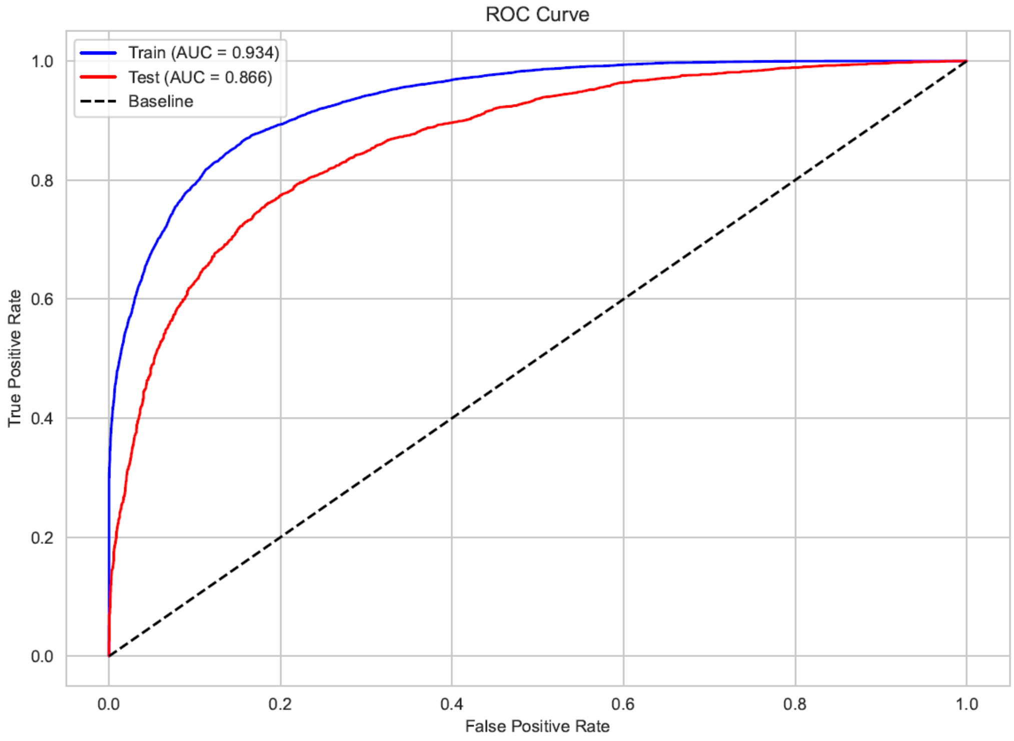

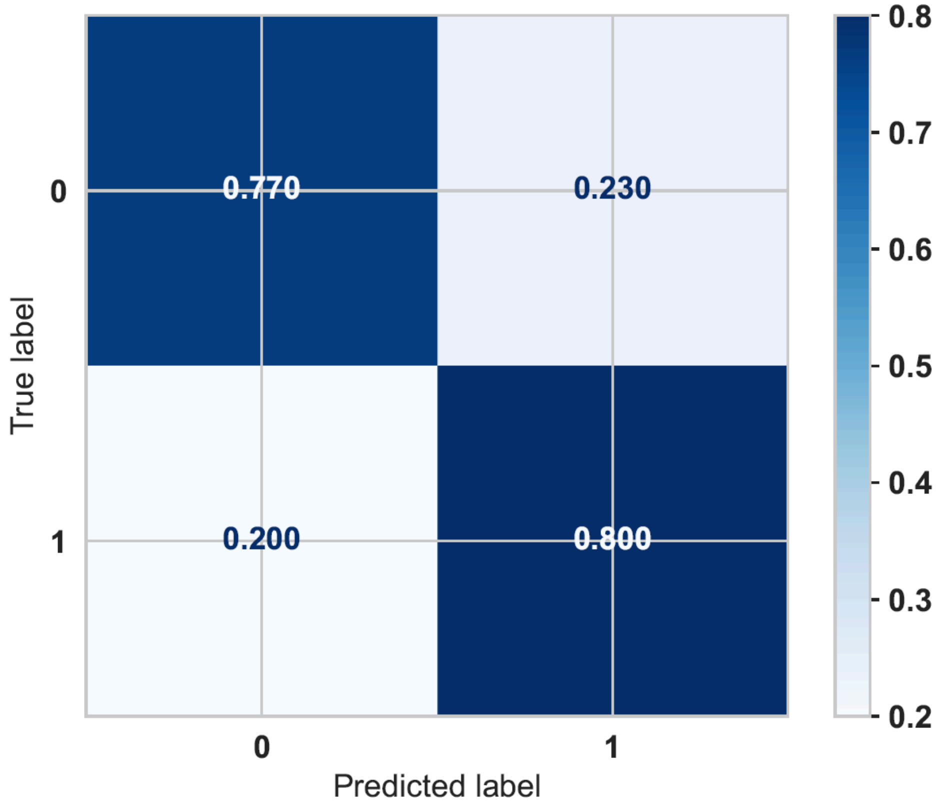

1.1.5.2. XGBoost

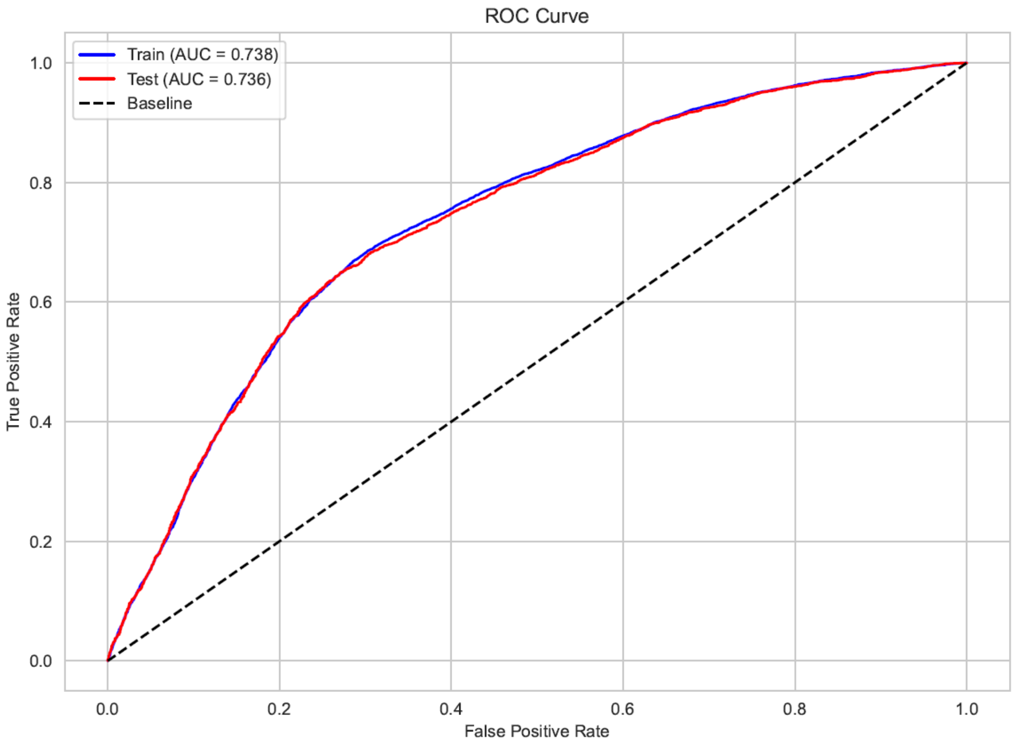

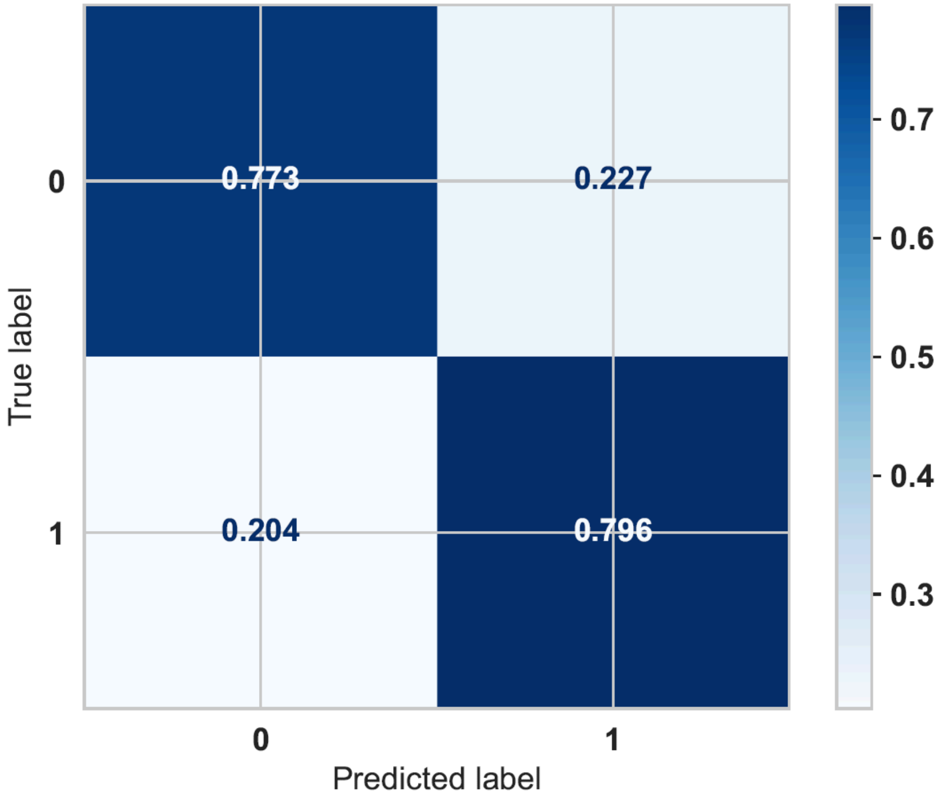

1.1.5.3. CatBoost

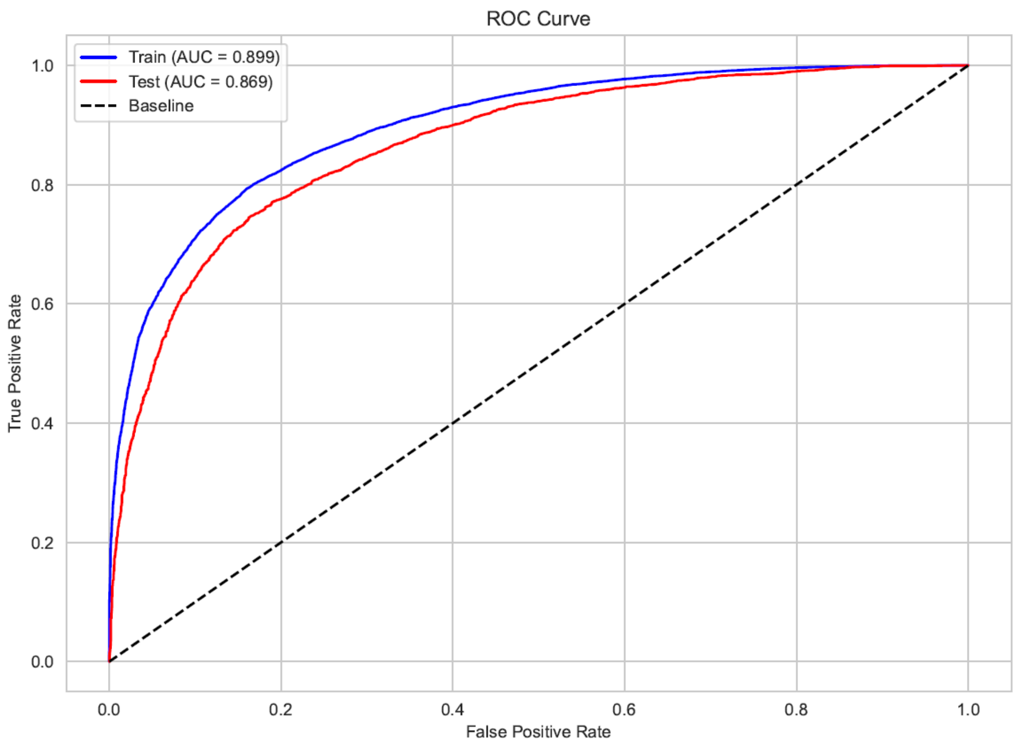

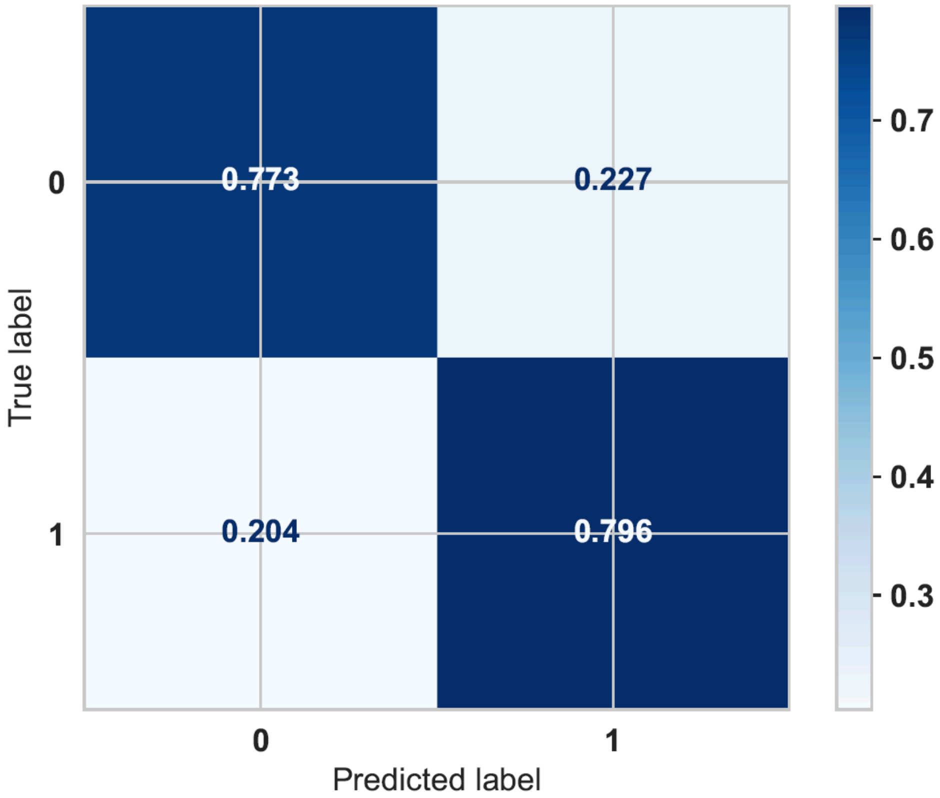

1.1.5.4. DecisionTreeClassifier

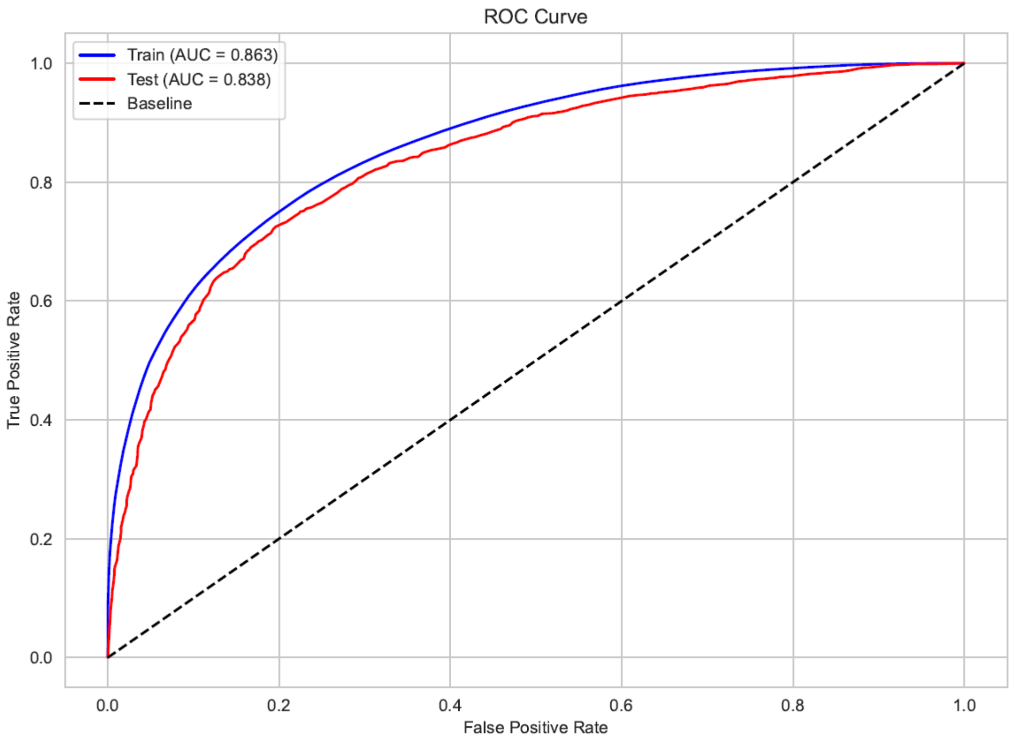

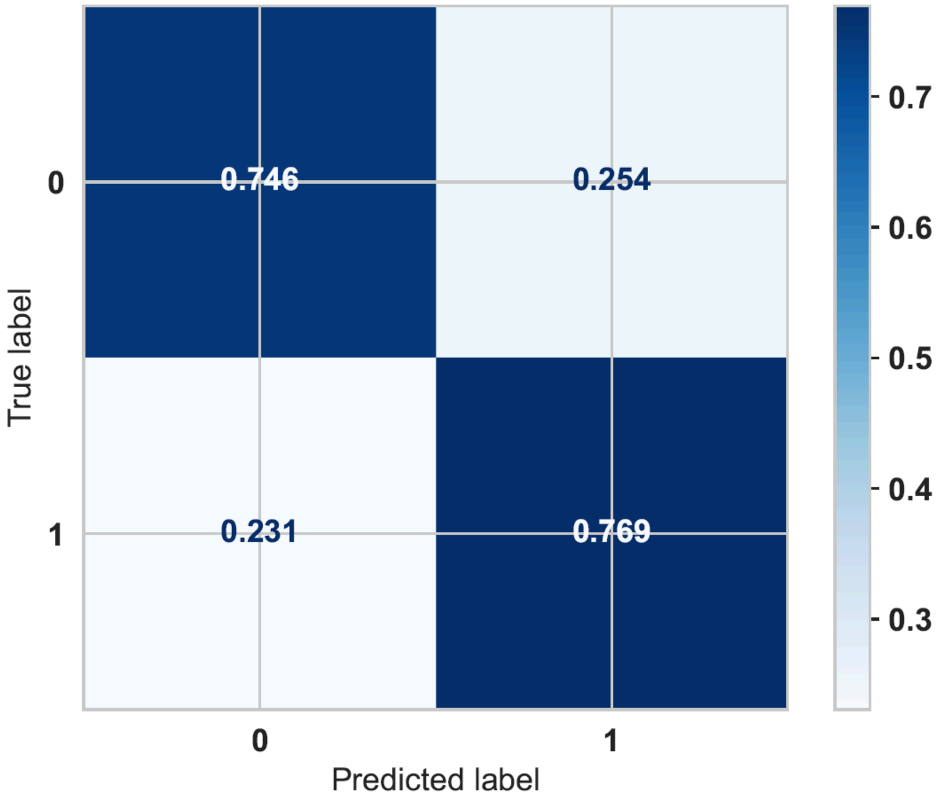

1.1.5.5. ExtraTreesClassifier

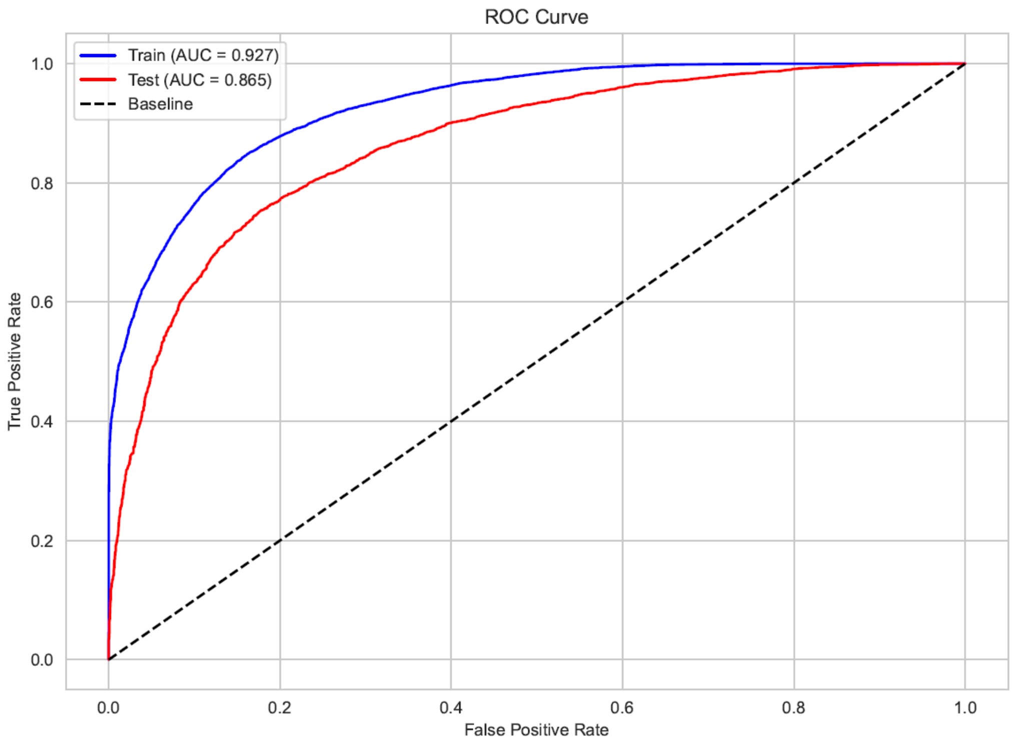

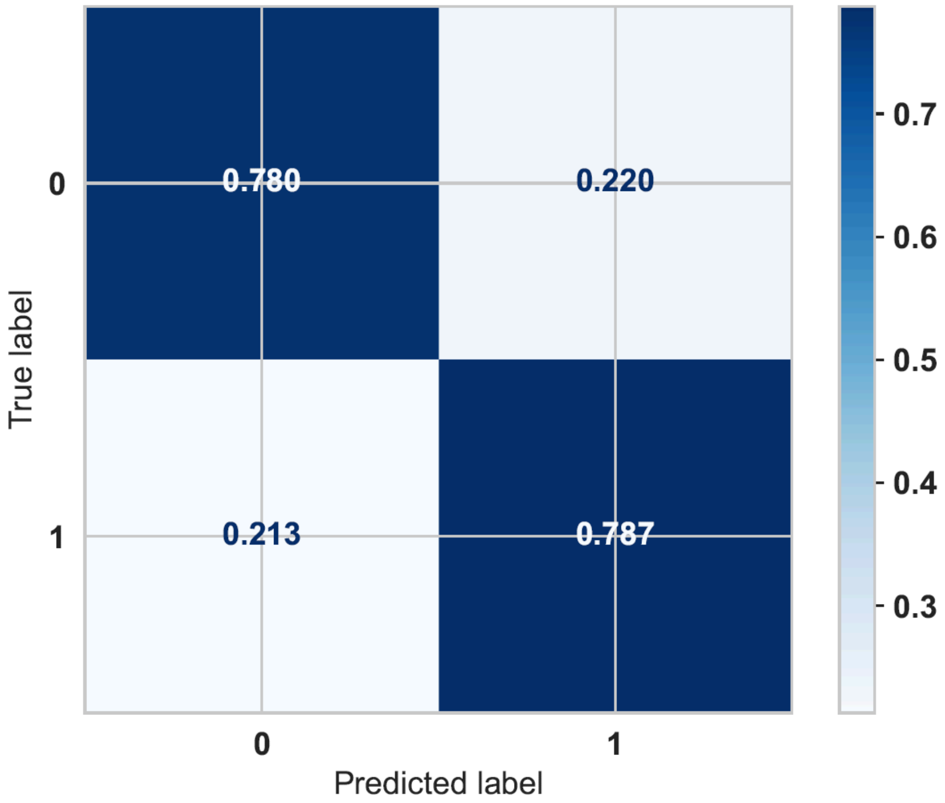

1.1.5.6. GradientBoosting

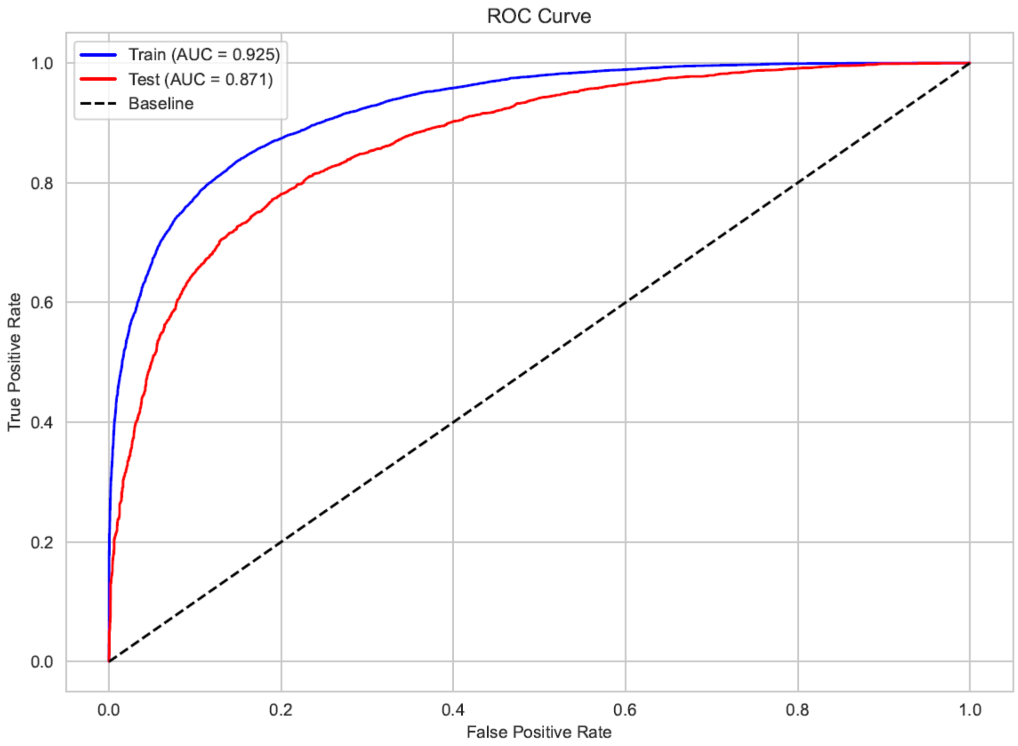
ROC Curve
Train (AUC = 0.925)
Test (AUC = 0.871)
Baseline
True Positive Rate
False Positive Rate
0.0
0.2
0.4
0.6
0.8
1.0

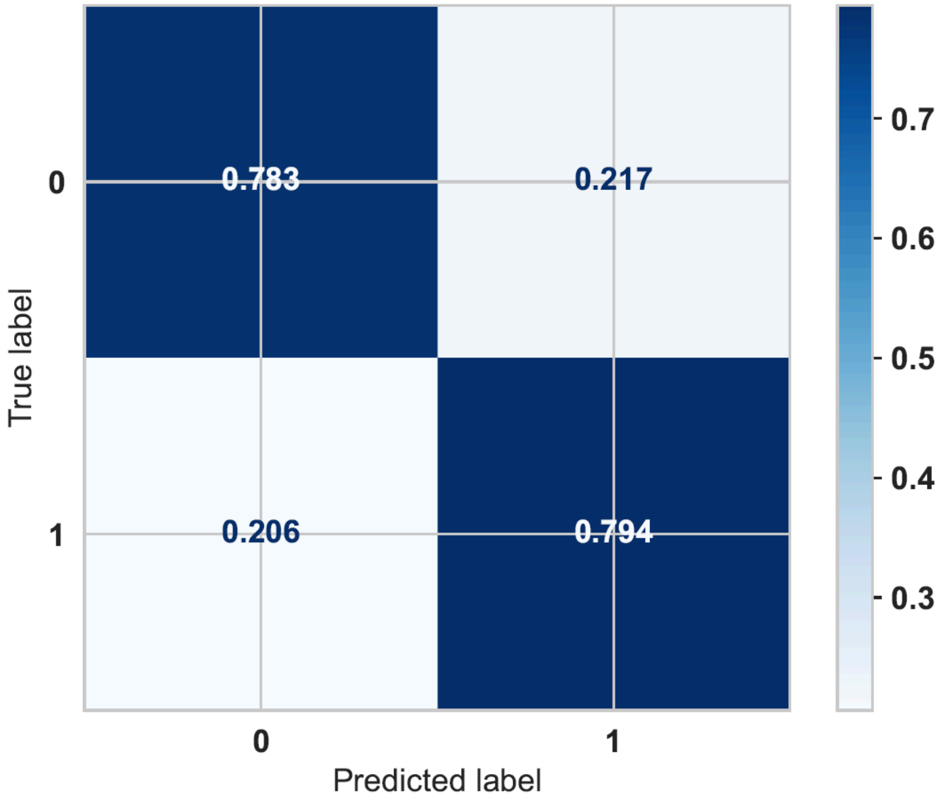
0.783
0.217
0.206
0.794
True label
Predicted label
0
1
0.7
0.6
0.5
0.4
0.3

1.1.5.7. KNN

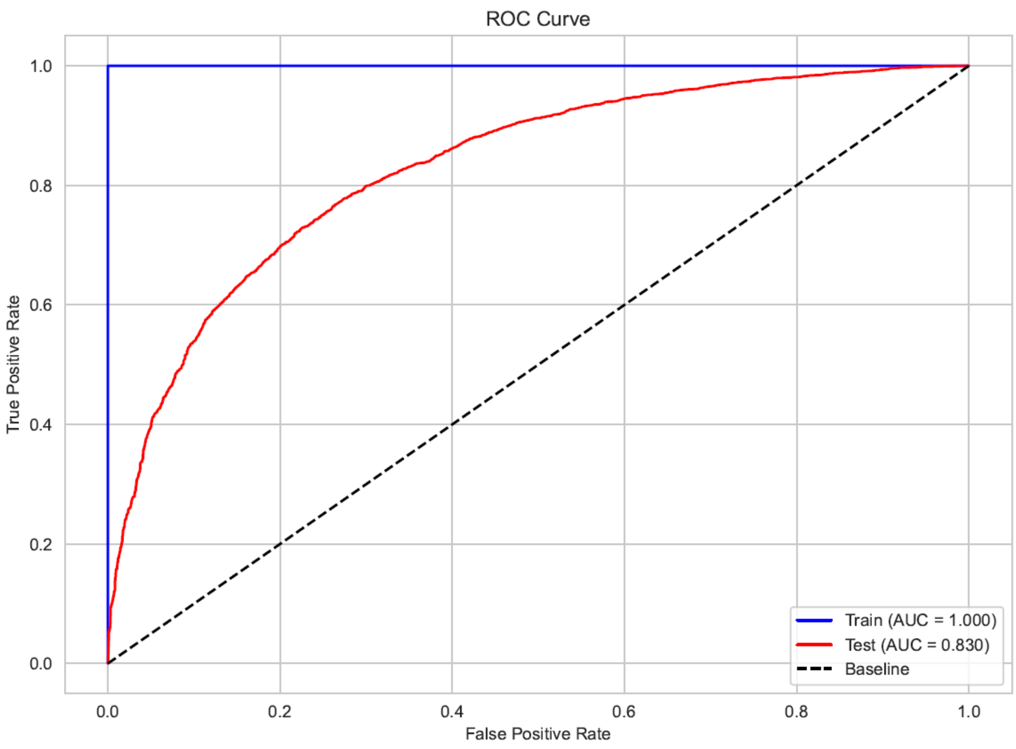

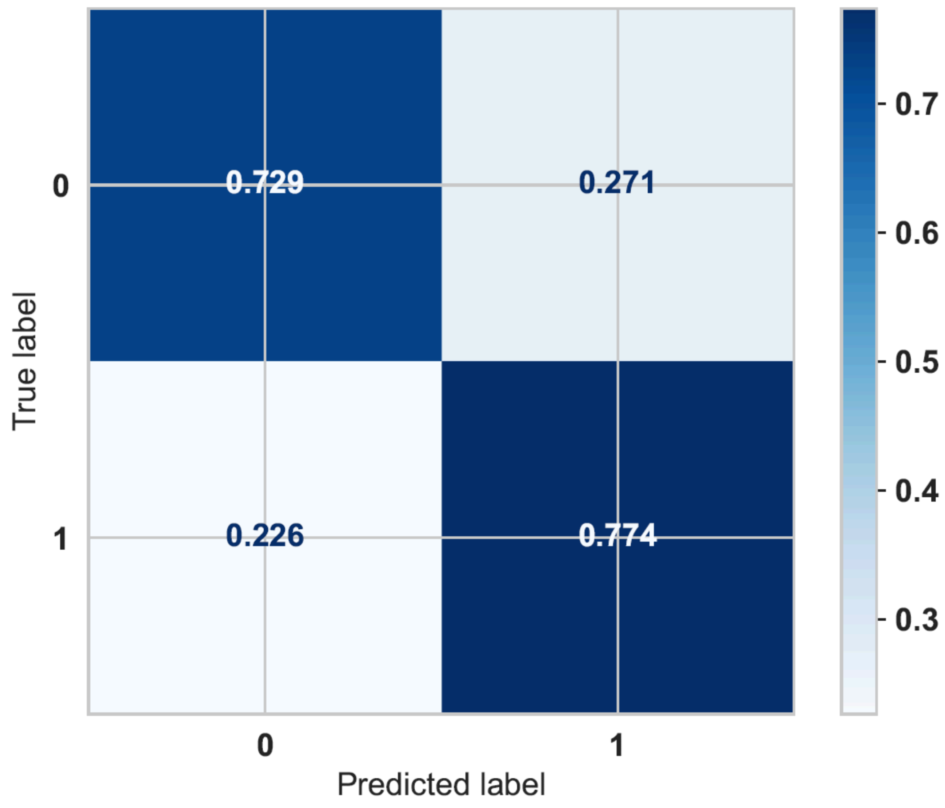

1.1.5.8. lightgbm

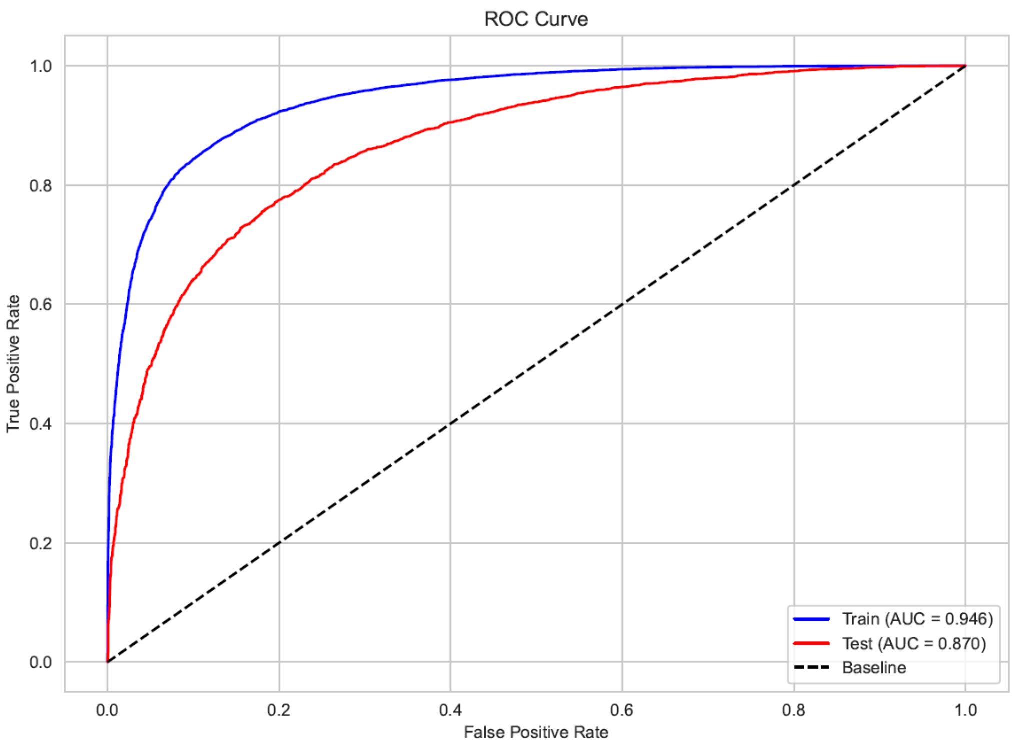

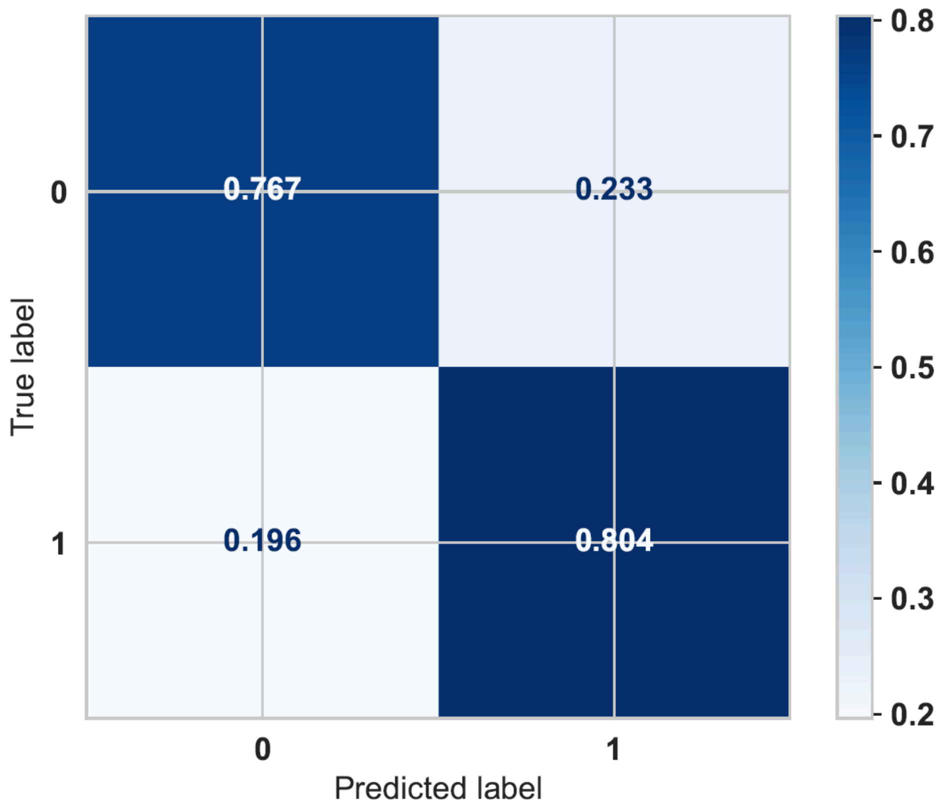

1.2. HyperOpt

1.2.1. Alive 1 year

1.2.1.1. Random Forest

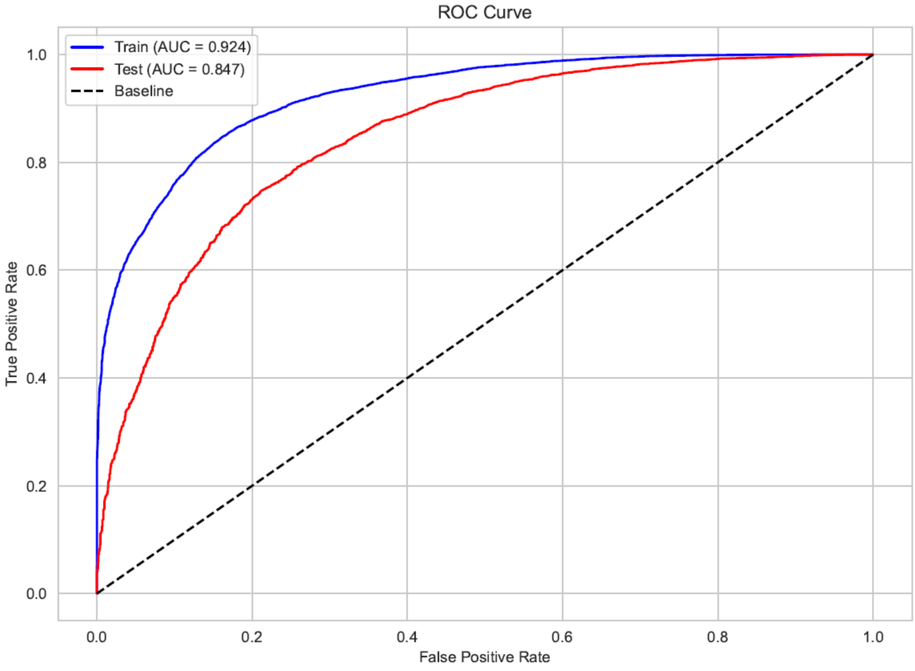

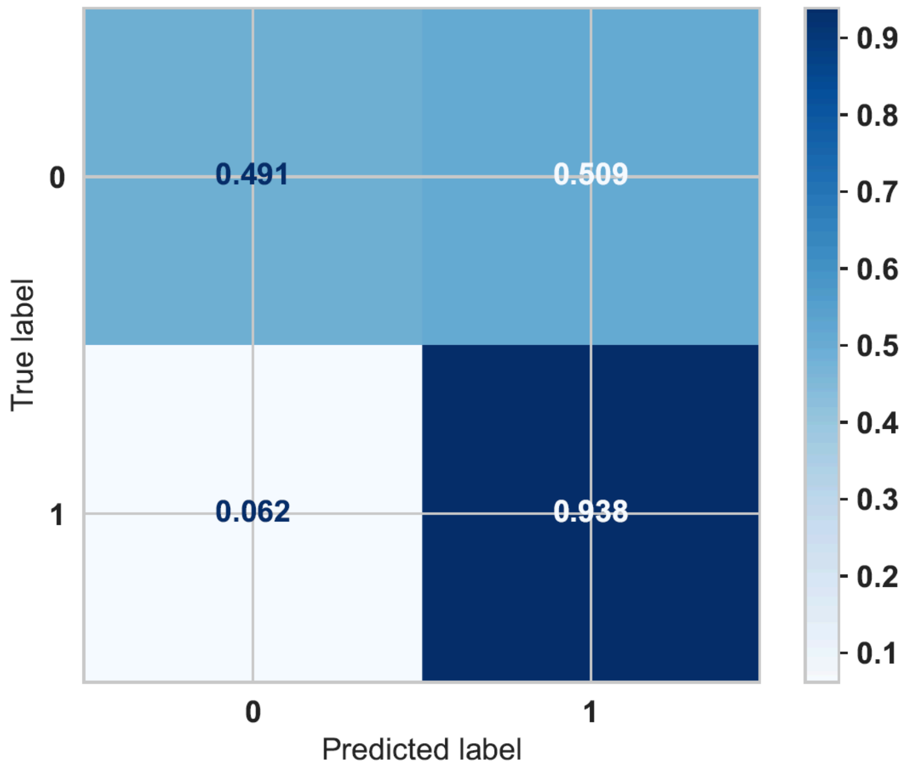

1.2.1.2. XGBoost

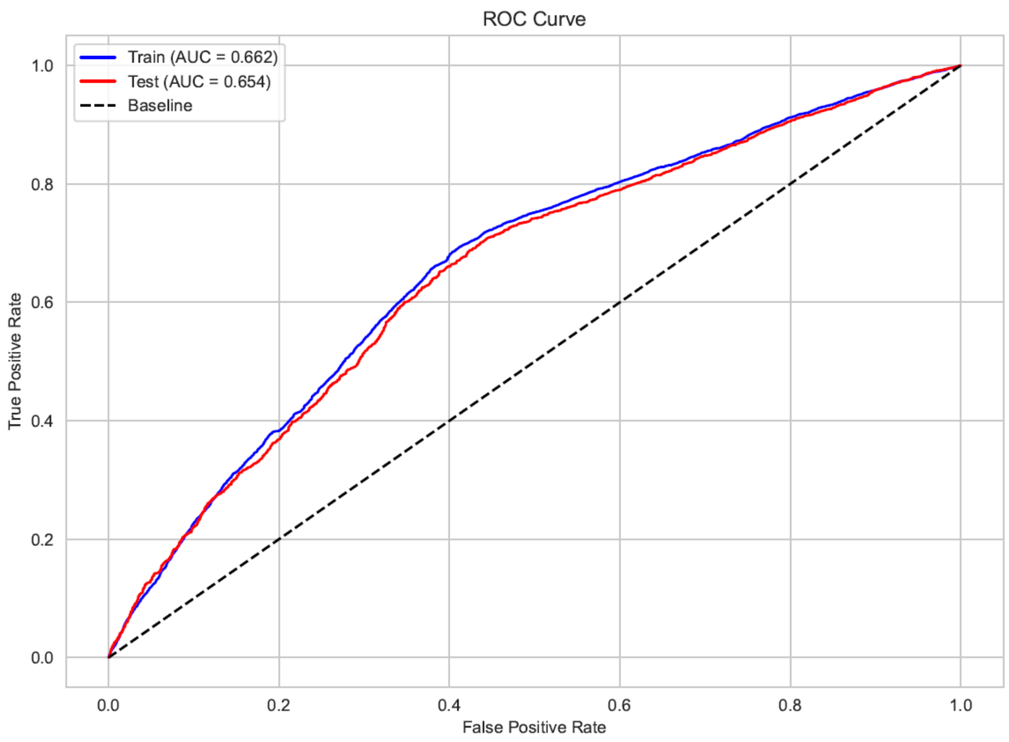

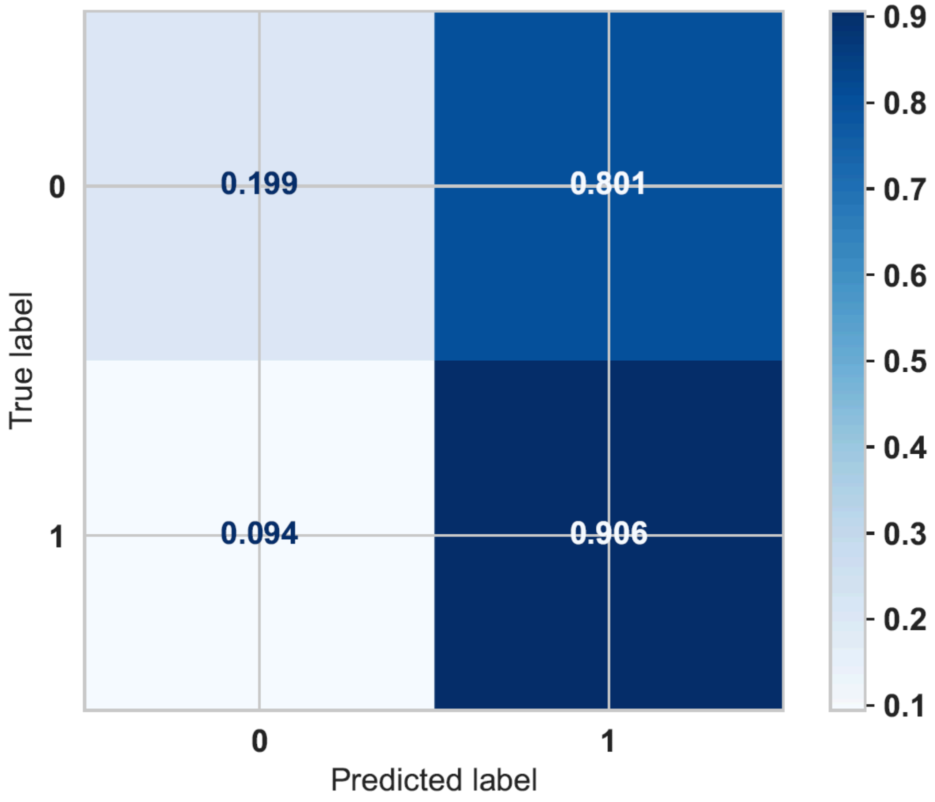

1.2.1.3. CatBoost

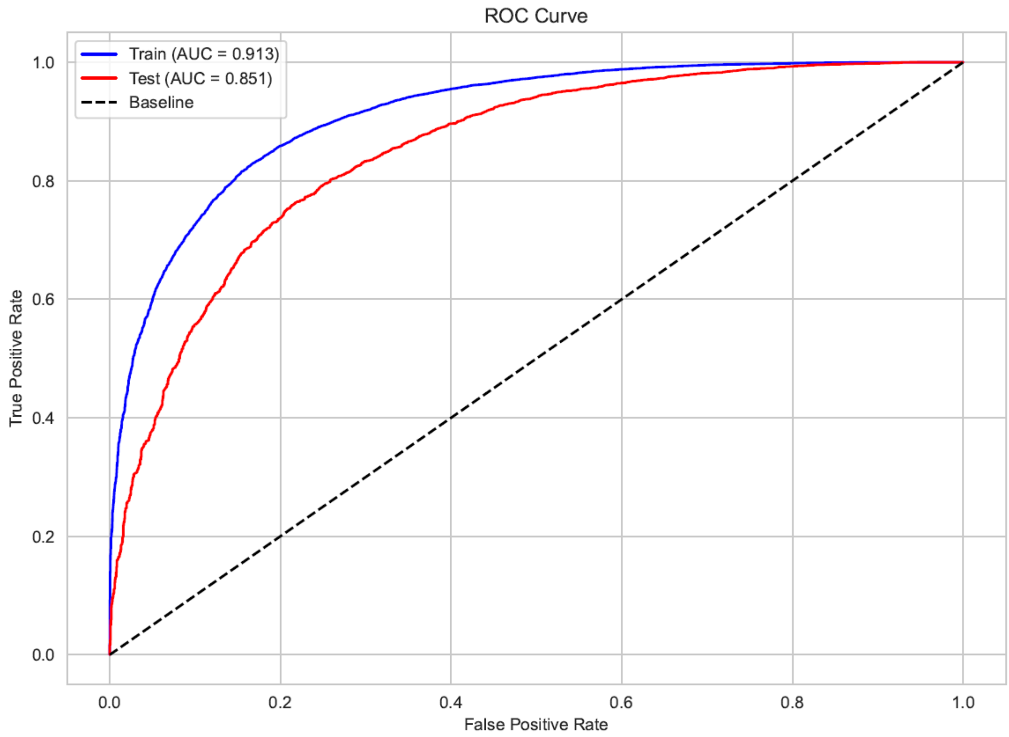

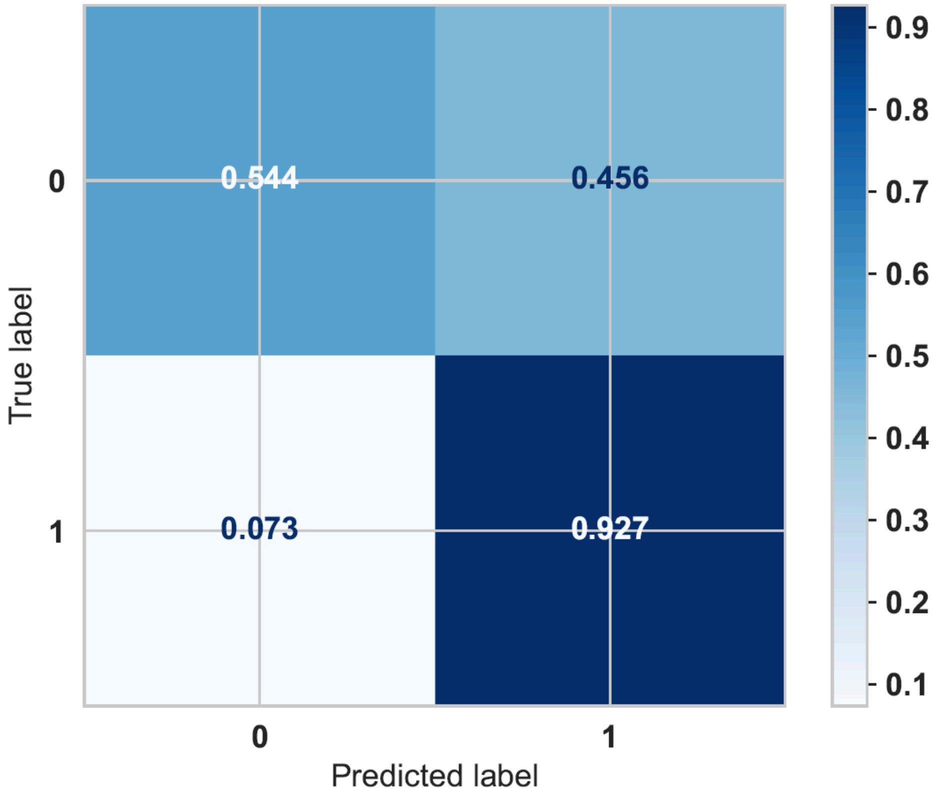

1.2.1.4. DecisionTreeClassifier

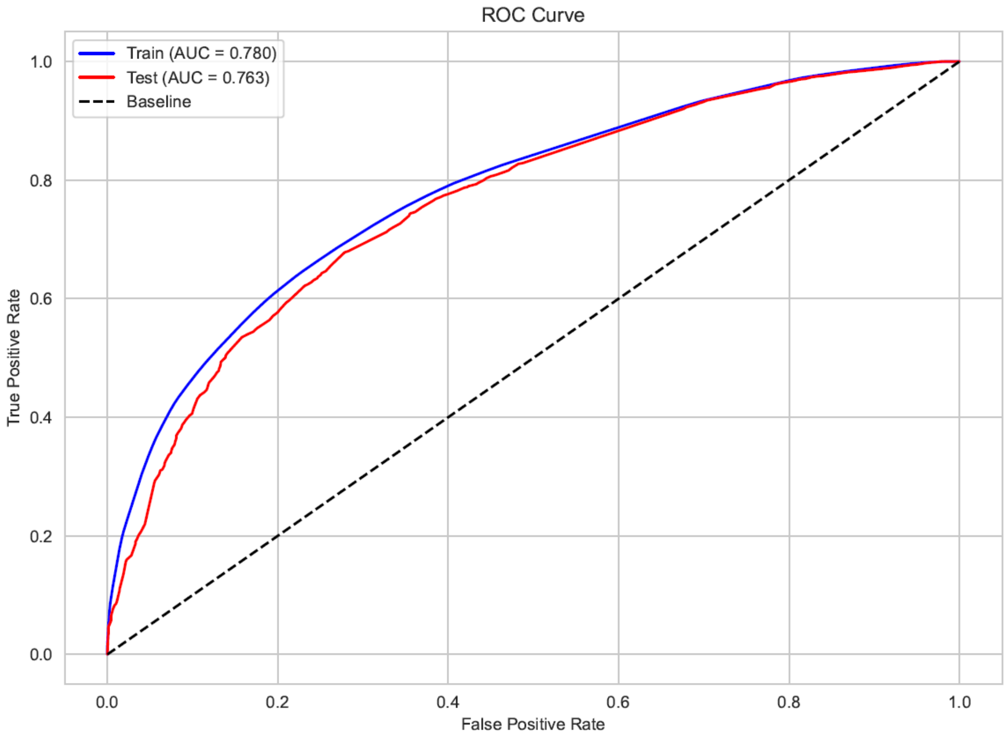

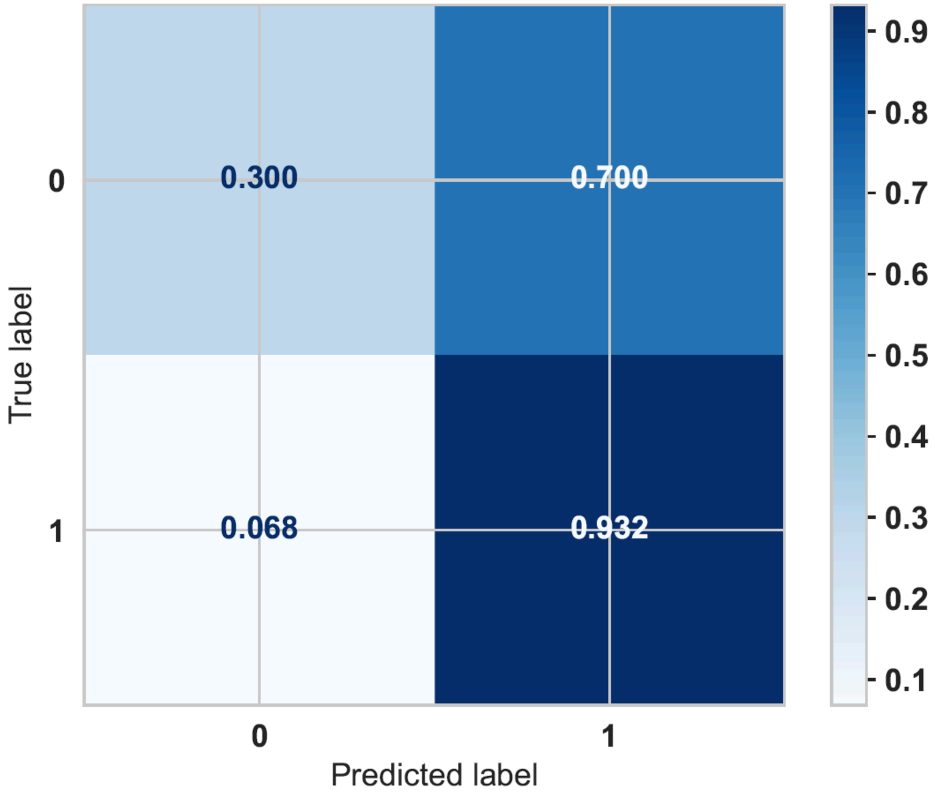

1.2.1.5. ExtraTreesClassifier

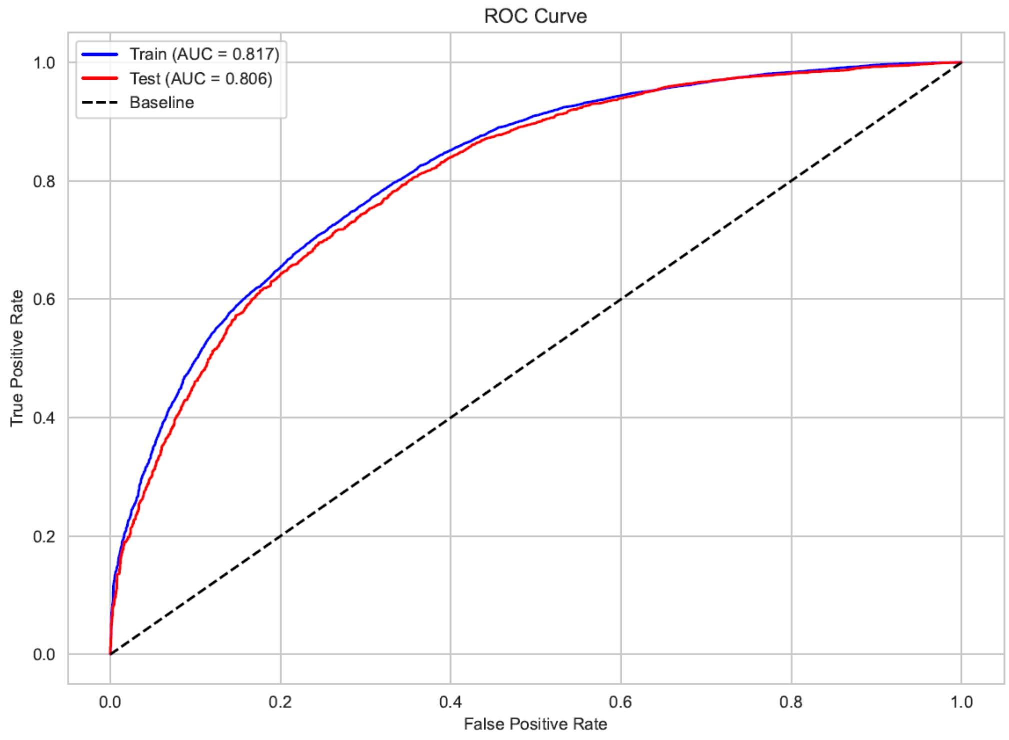

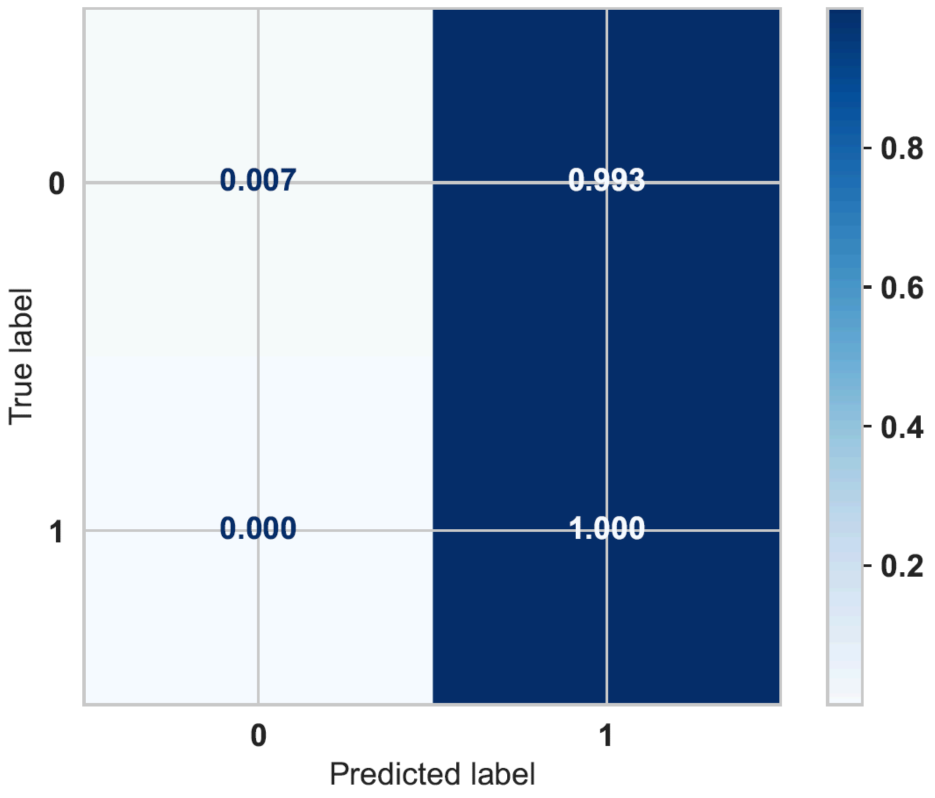

1.2.1.6. GradientBoosting

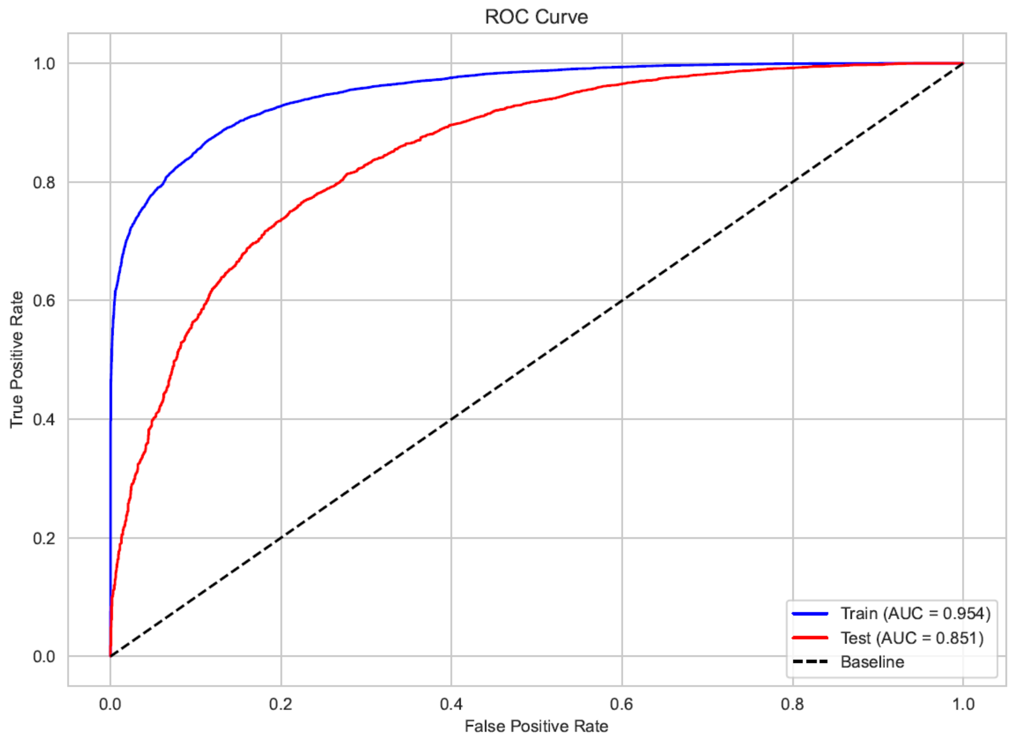
ROC Curve
True Positive Rate
False Positive Rate
Train (AUC = 0.954)
Test (AUC = 0.851)
Baseline

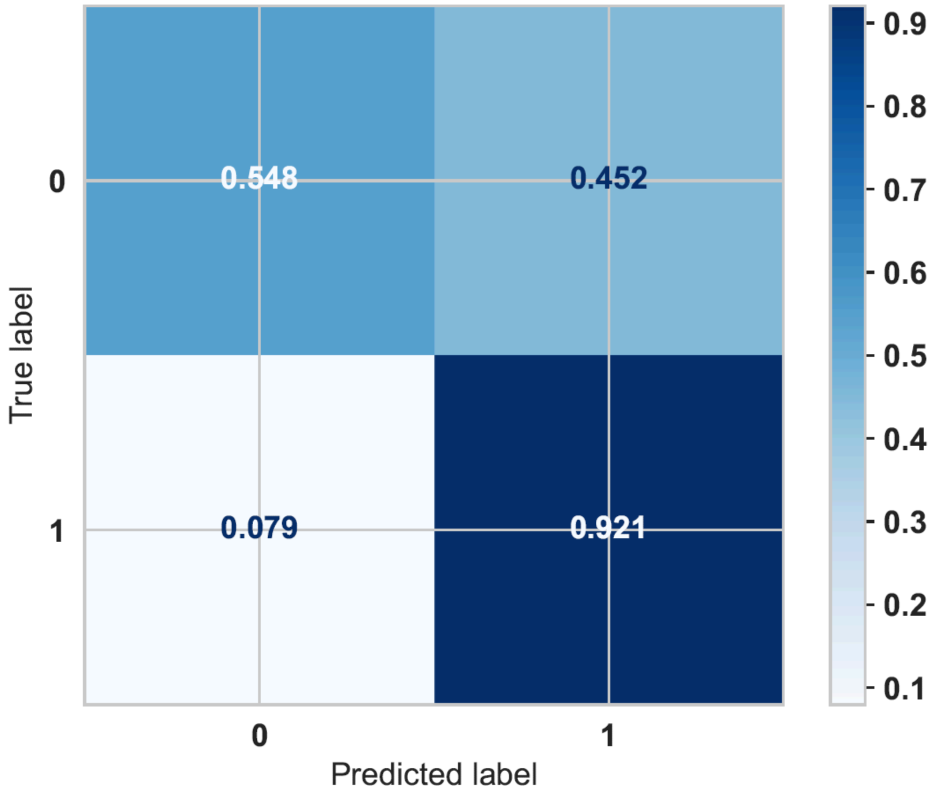
0.548
0.452
0.079
0.921
True label
Predicted label

1.2.1.7. KNN

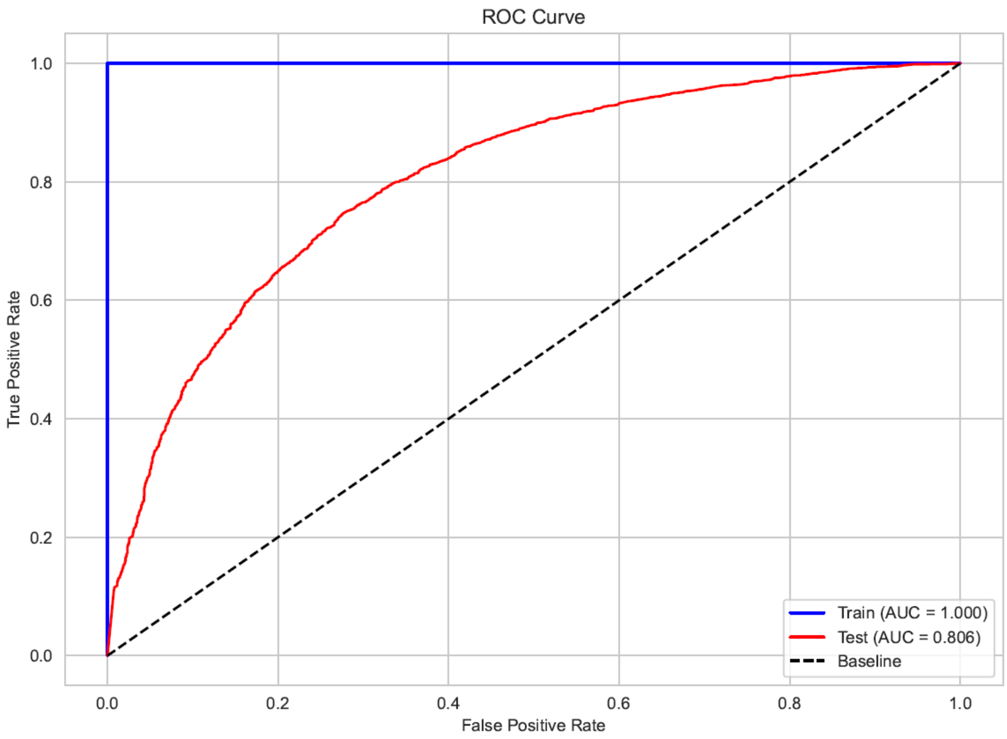

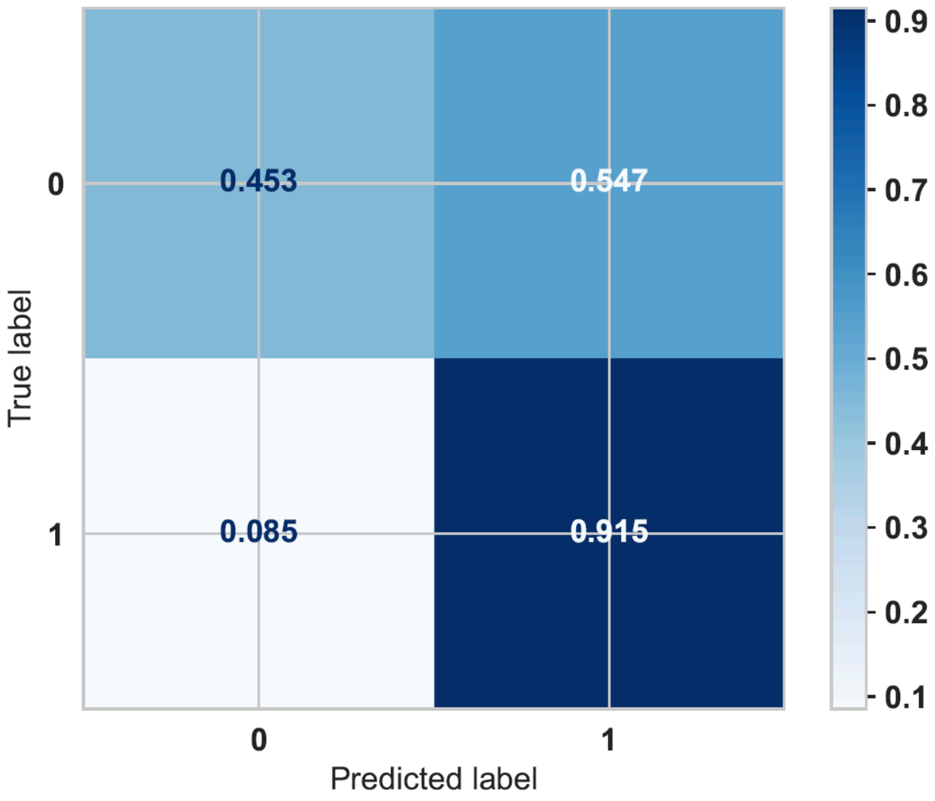

1.2.1.8. lightgbm

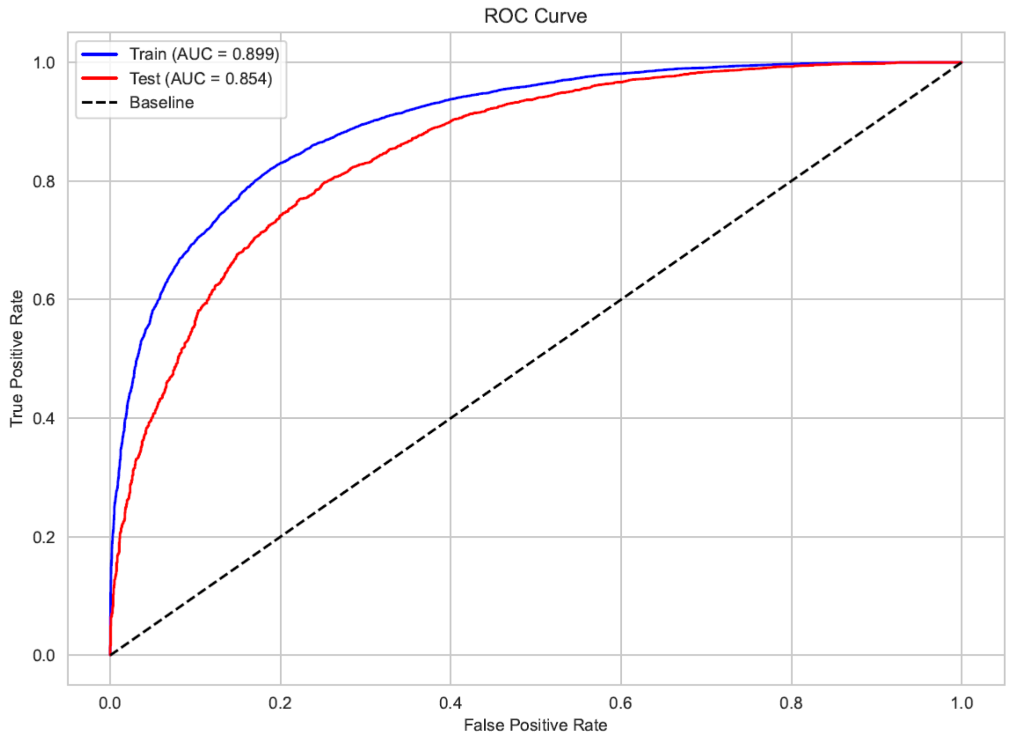
ROC Curve
Train (AUC = 0.899)
Test (AUC = 0.854)
Baseline
True Positive Rate
False Positive Rate
0.0
0.2
0.4
0.6
0.8
1.0

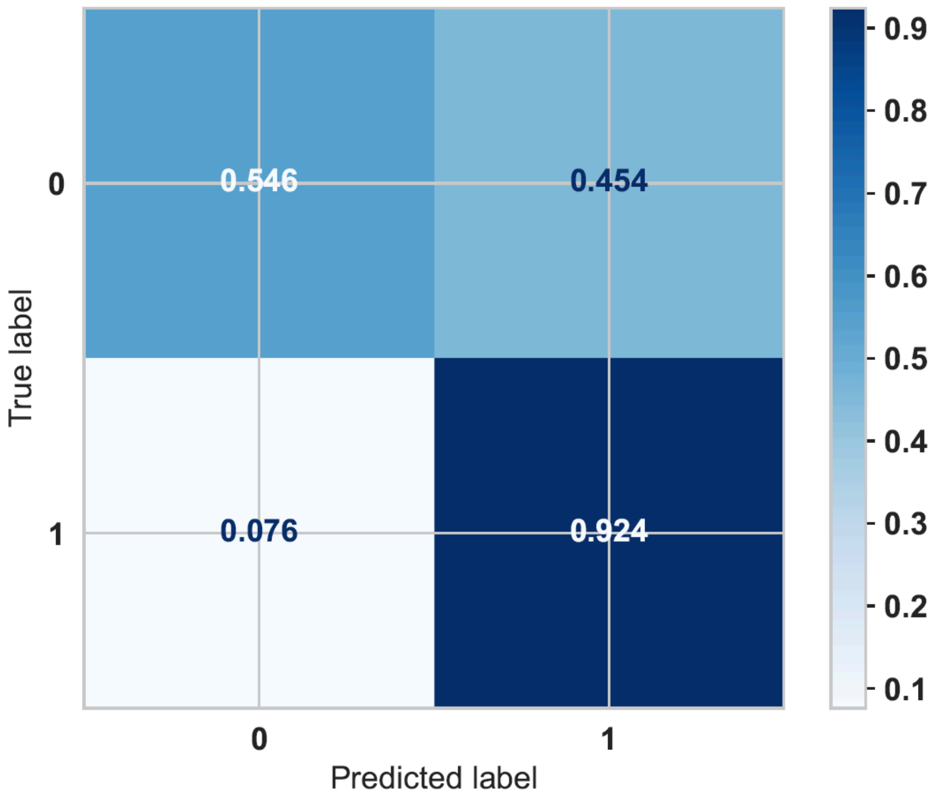
0.546
0.454
0.076
0.924
True label
Predicted label
0
1
0.9
0.8
0.7
0.6
0.5
0.4
0.3
0.2
0.1

1.2.2. Alive 3 year

1.2.2.1. Random Forest

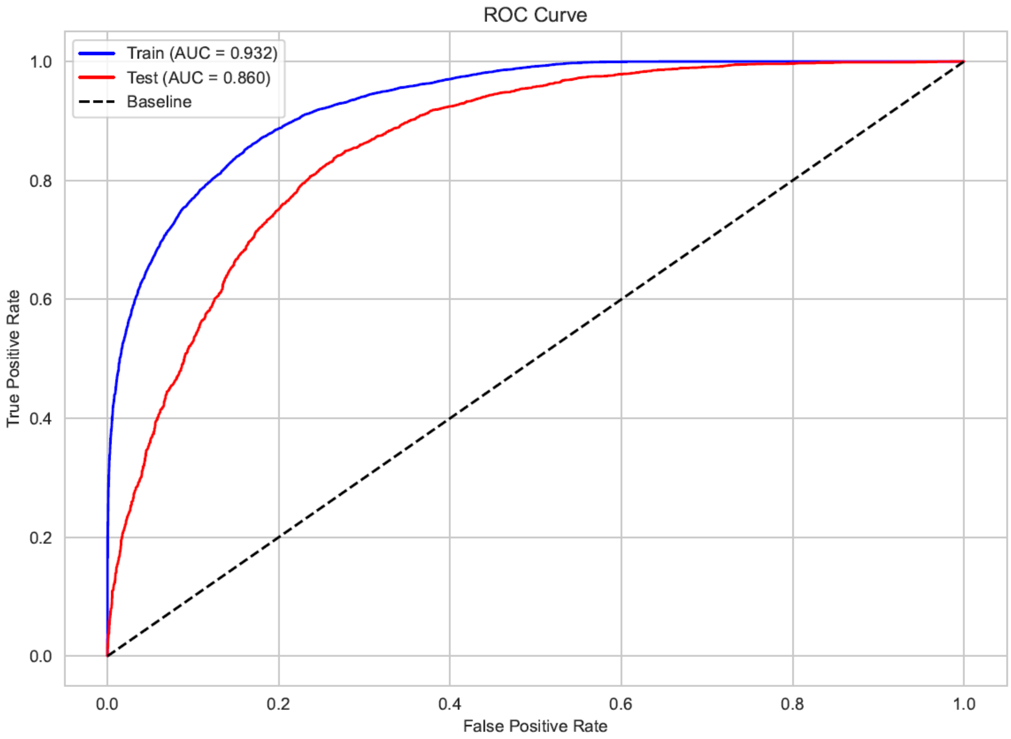

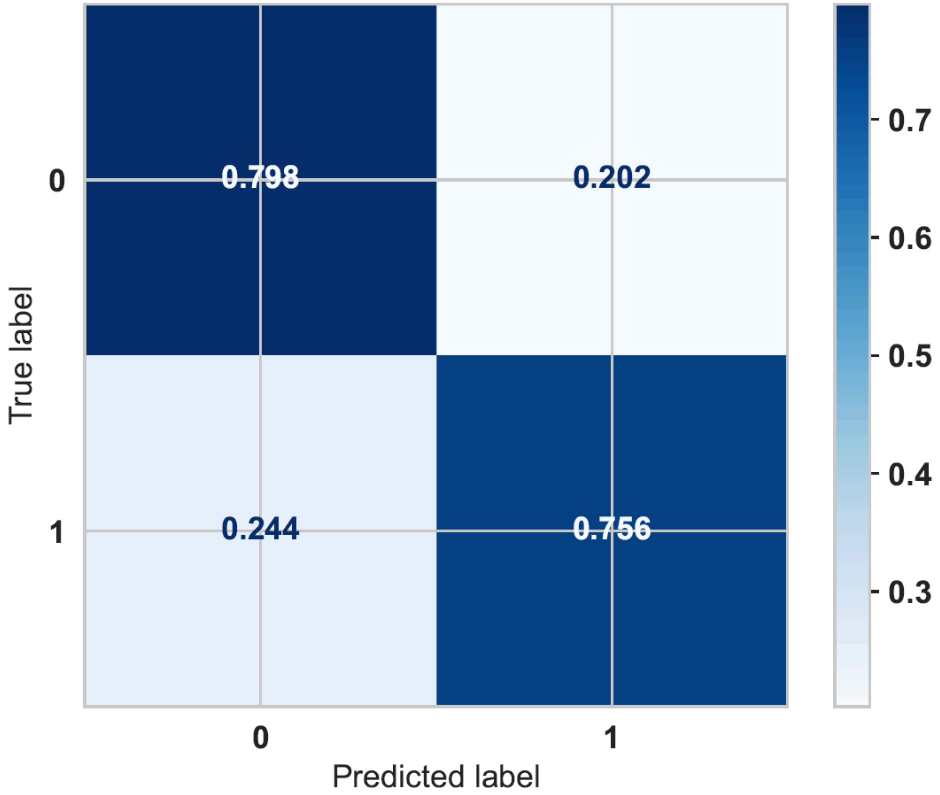

1.2.2.2. XGBoost

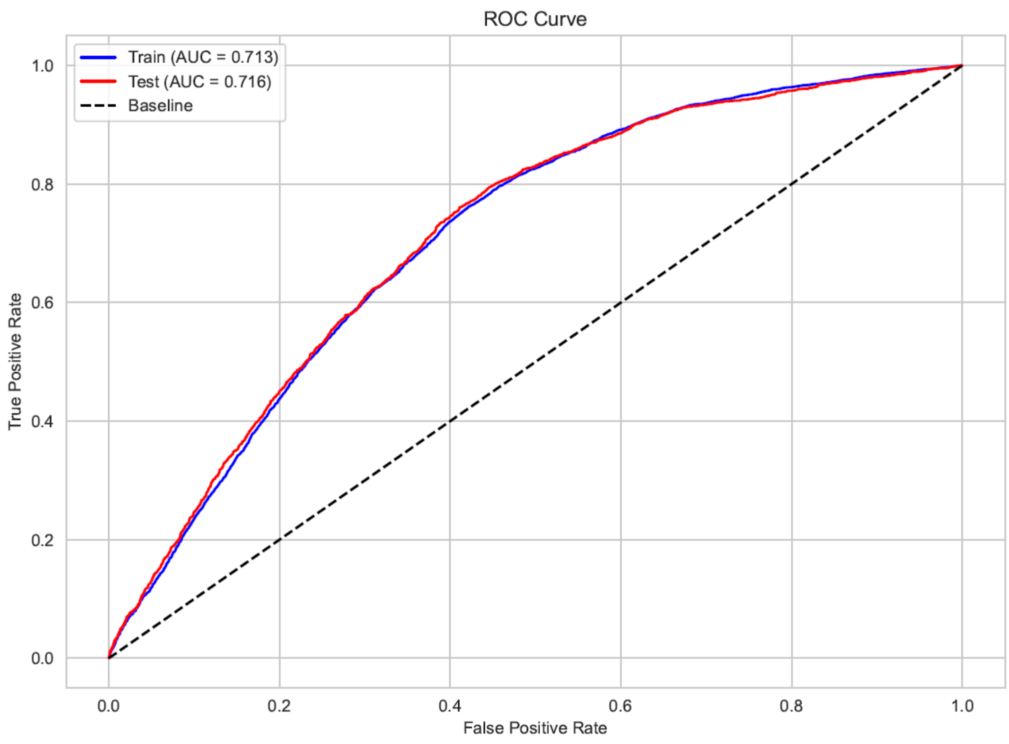

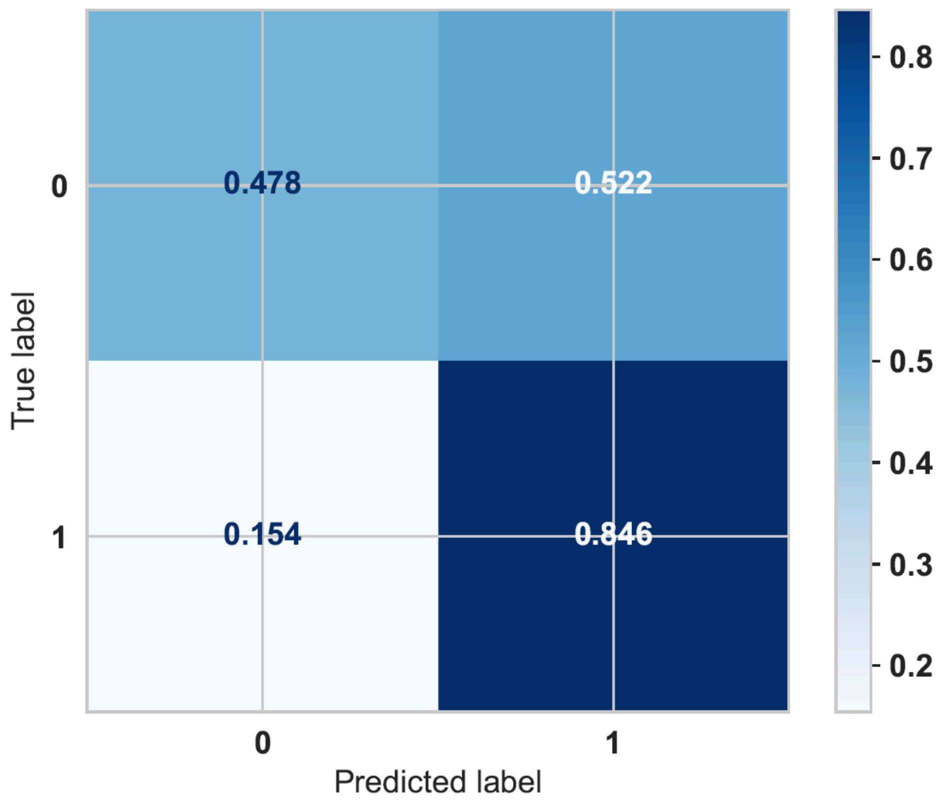

1.2.2.3. CatBoost

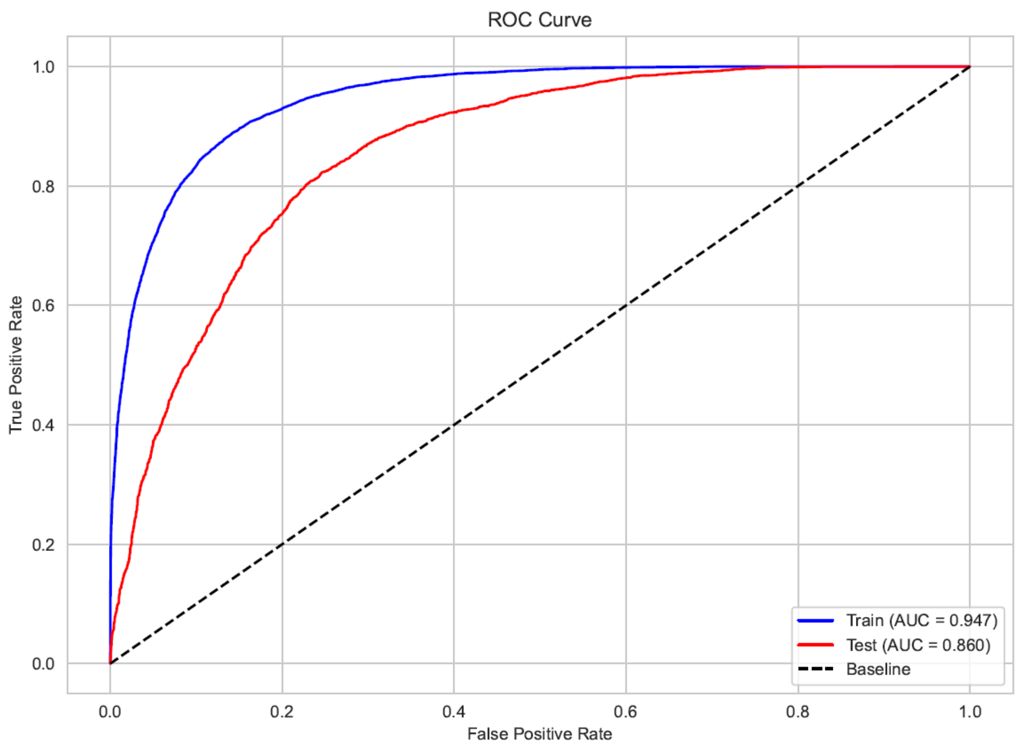
ROC Curve
True Positive Rate
False Positive Rate
0.0
0.2
0.4
0.6
0.8
1.0
Train (AUC = 0.947)
Test (AUC = 0.860)
Baseline

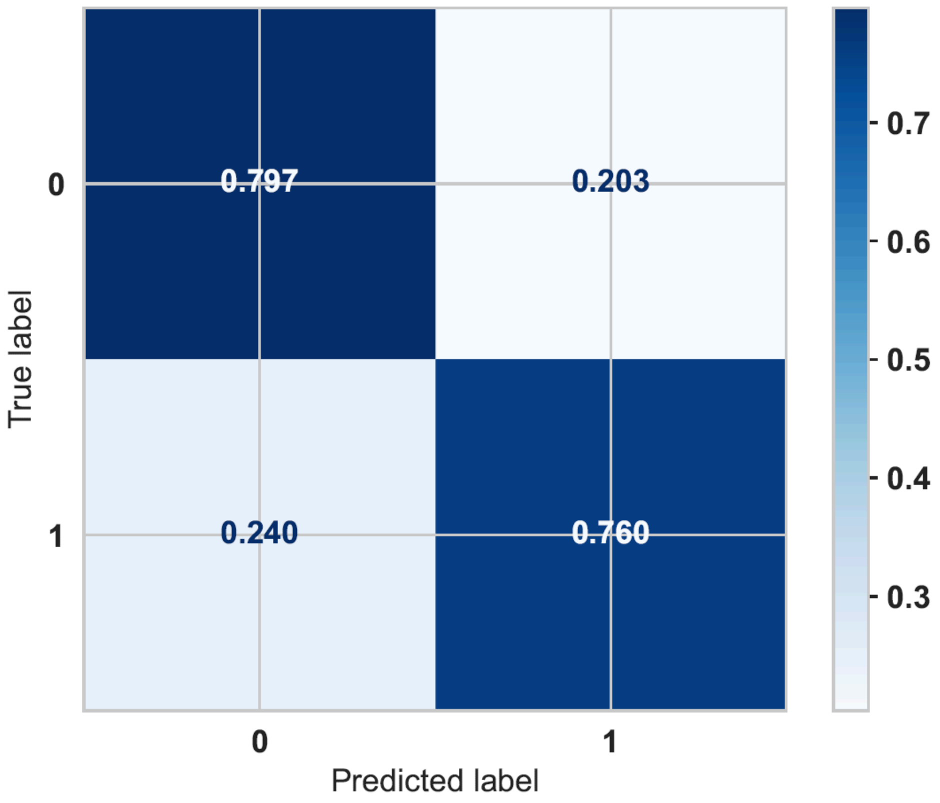
0.797
0.203
0.240
0.760
0
1
True label
Predicted label
0.7
0.6
0.5
0.4
0.3

1.2.2.4. DecisionTreeClassifier

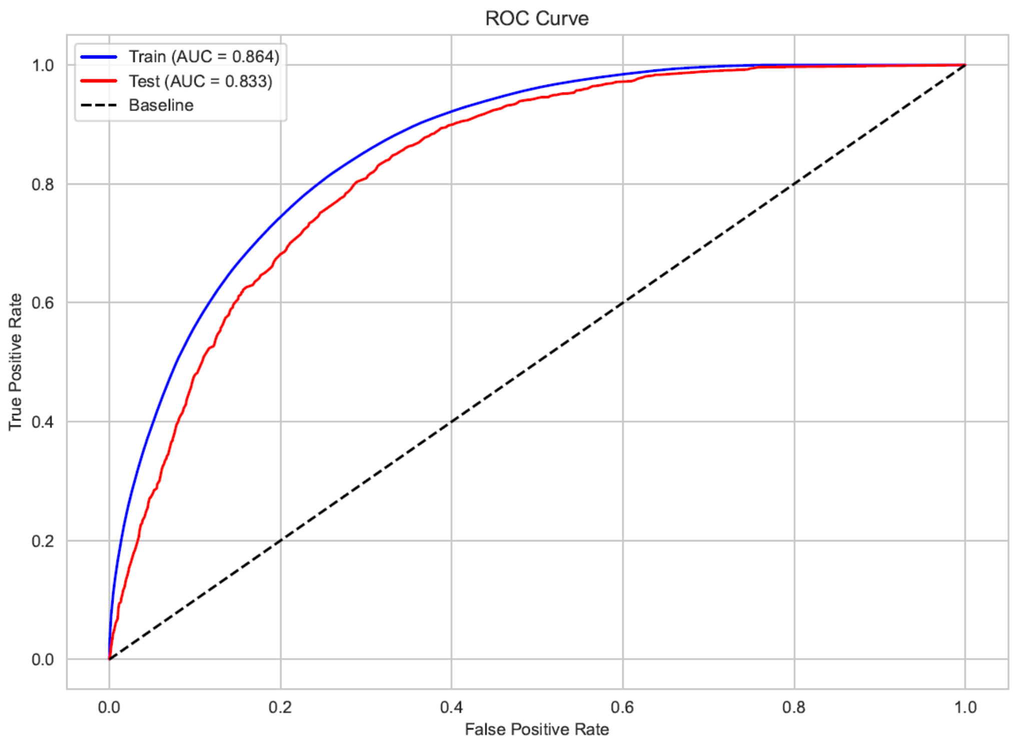

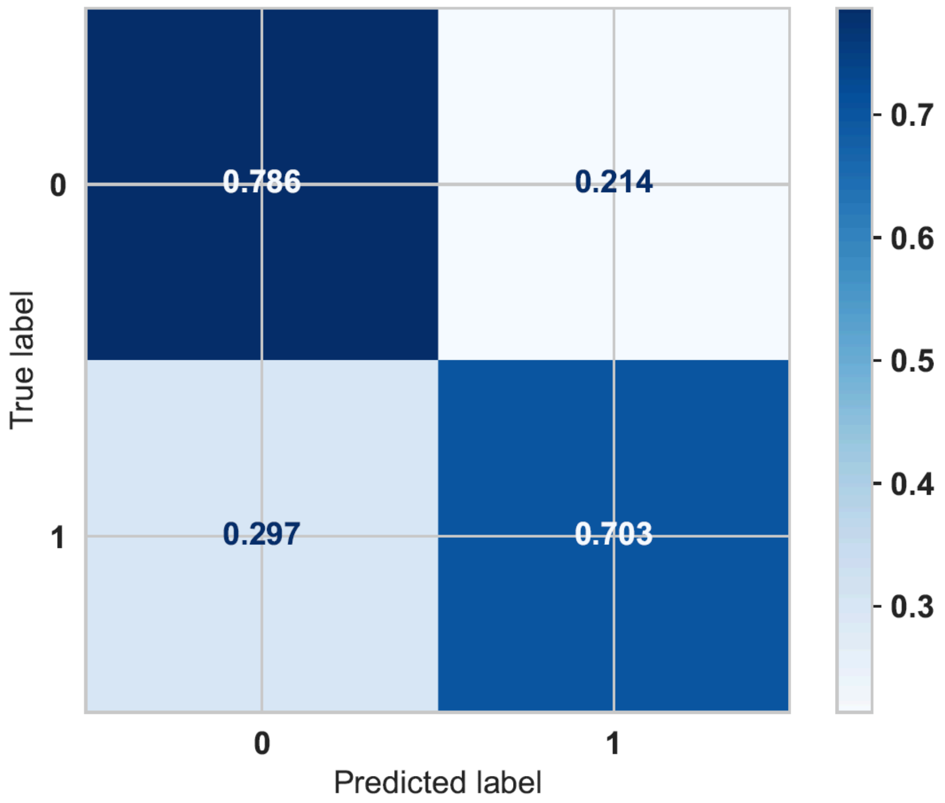

1.2.2.5. ExtraTreesClassifier

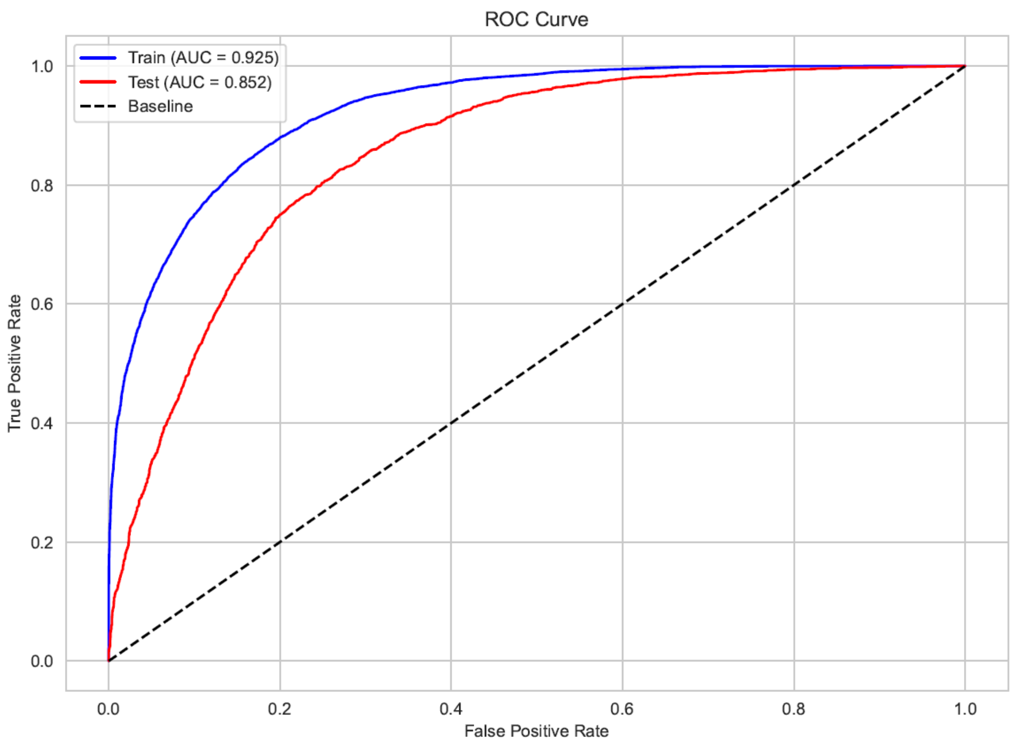

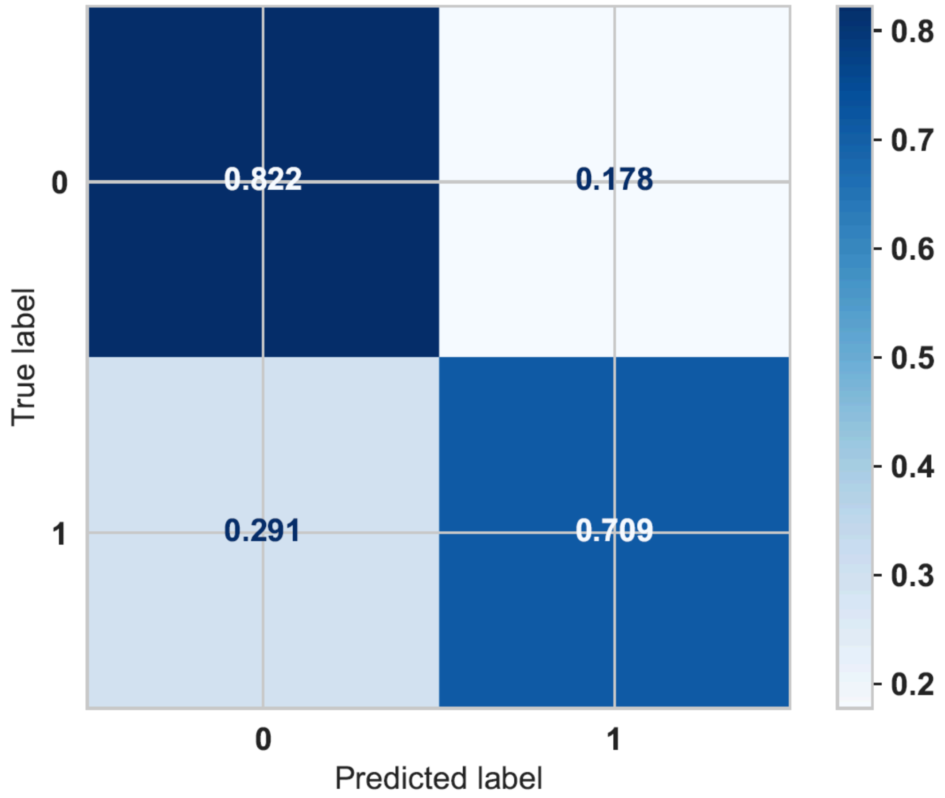

1.2.2.6. GradientBoosting

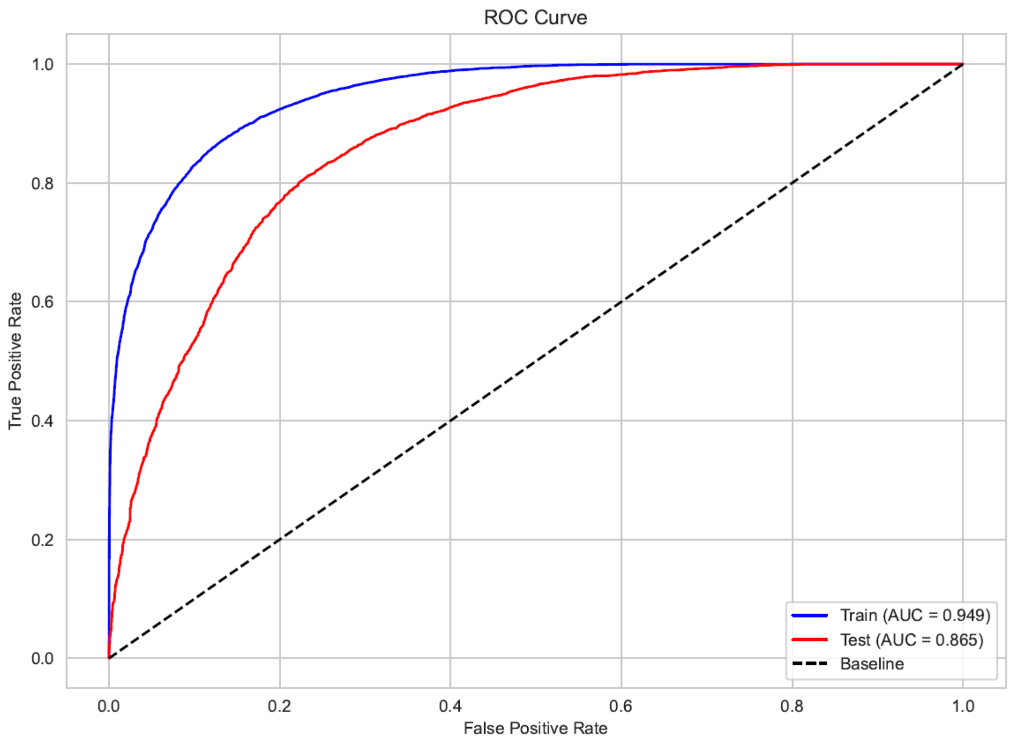

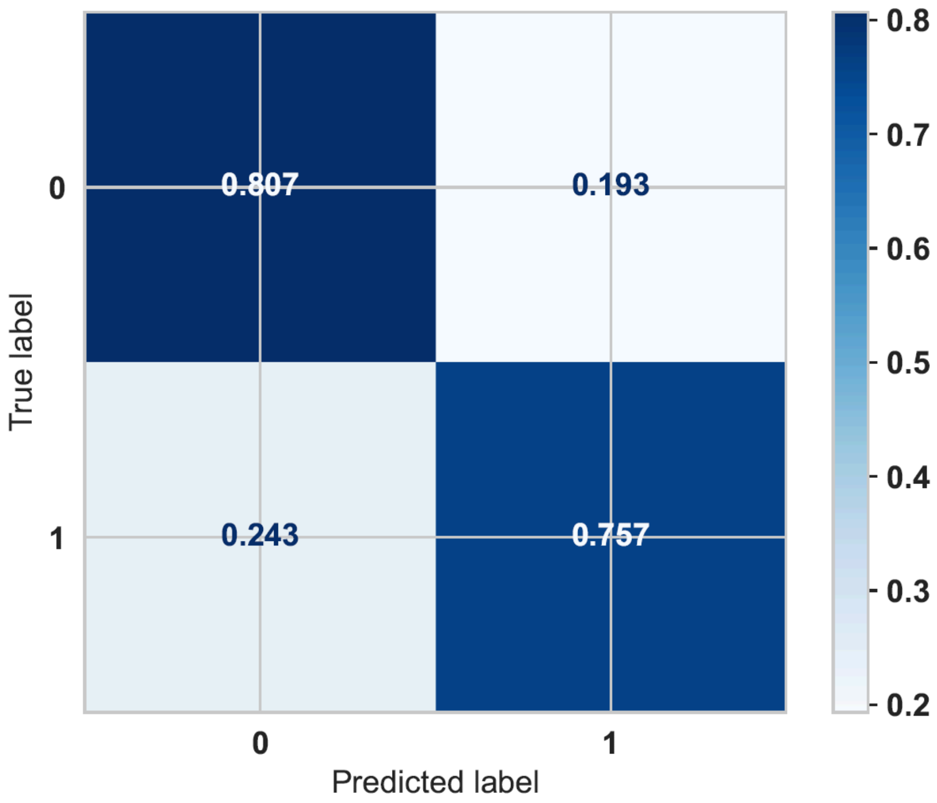

1.2.2.7. KNN

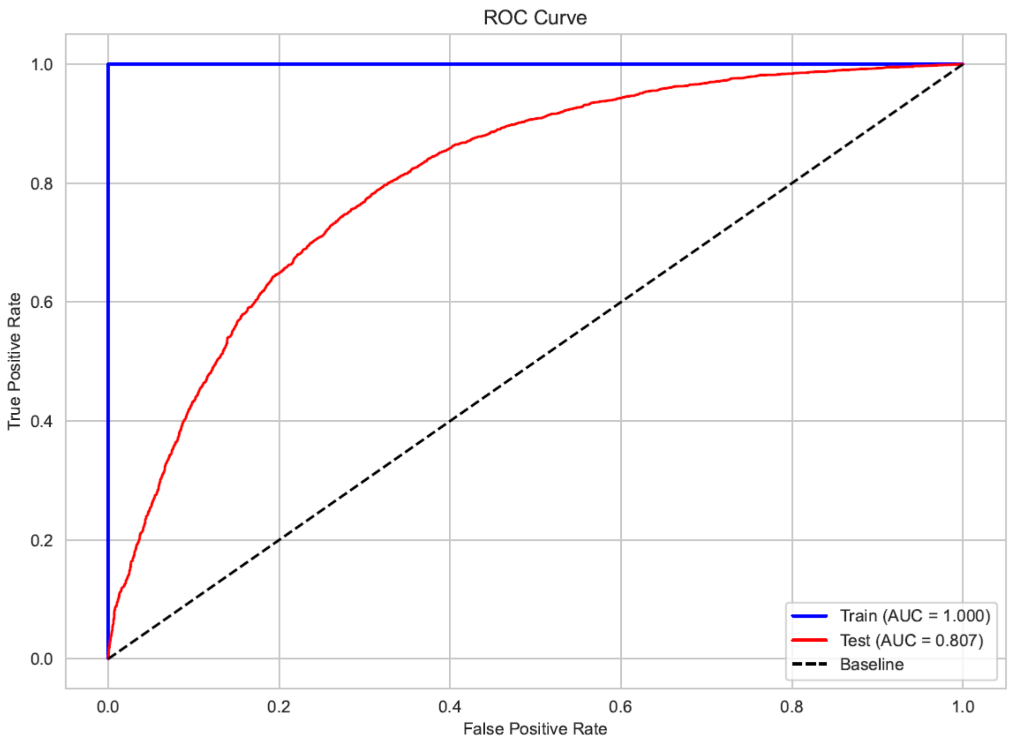

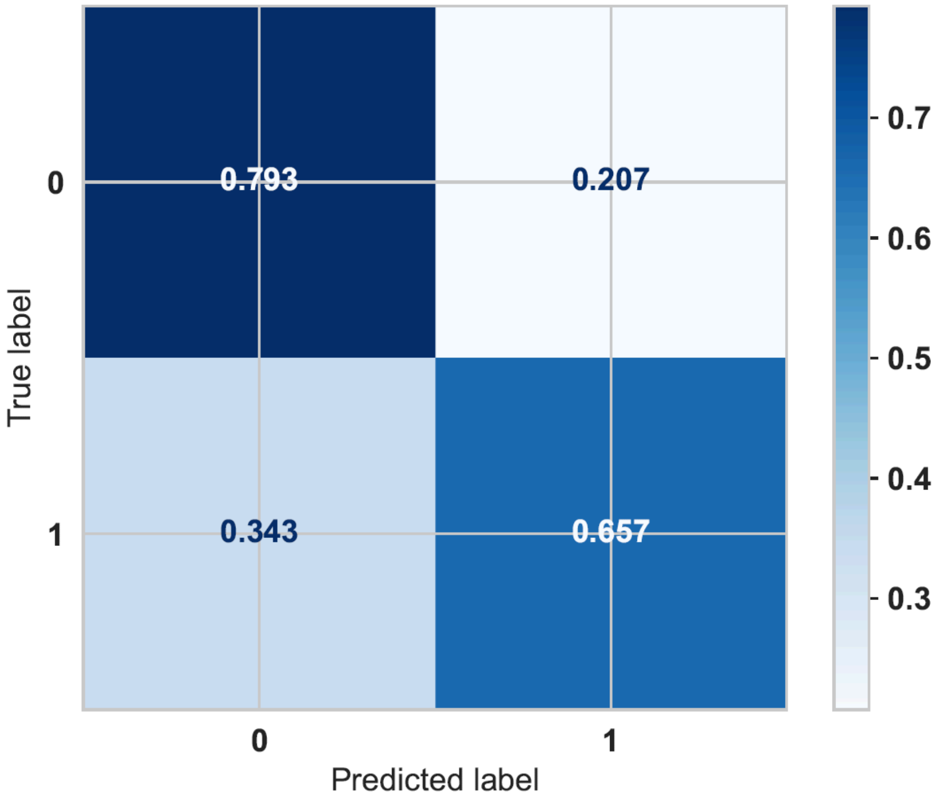

1.2.2.8. lightgbm

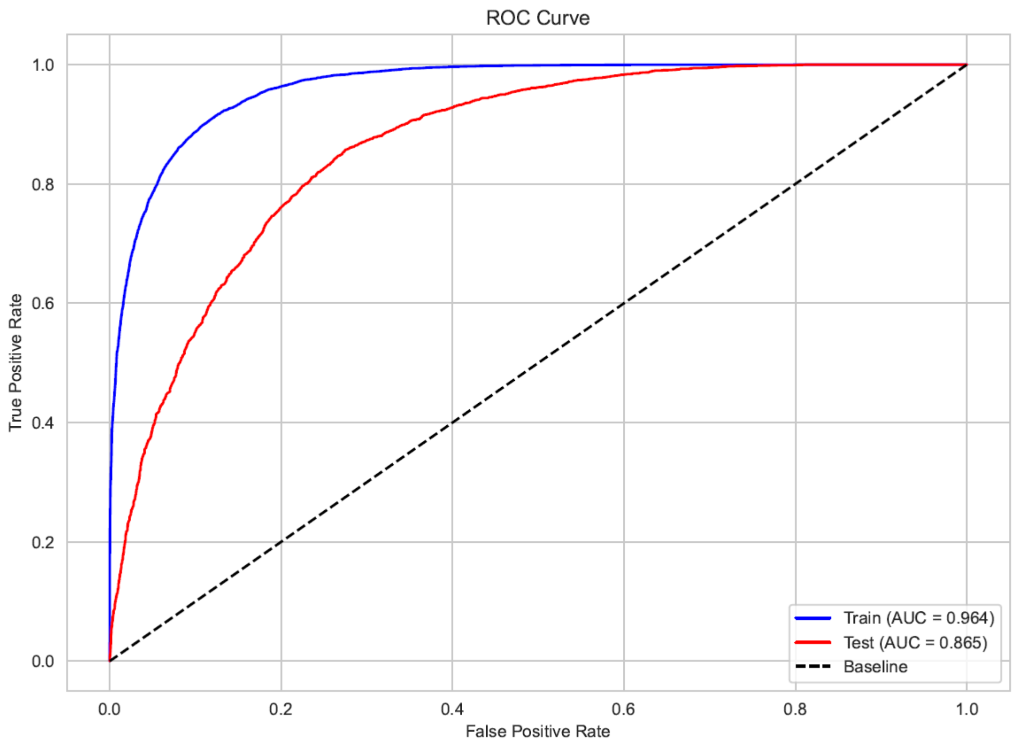

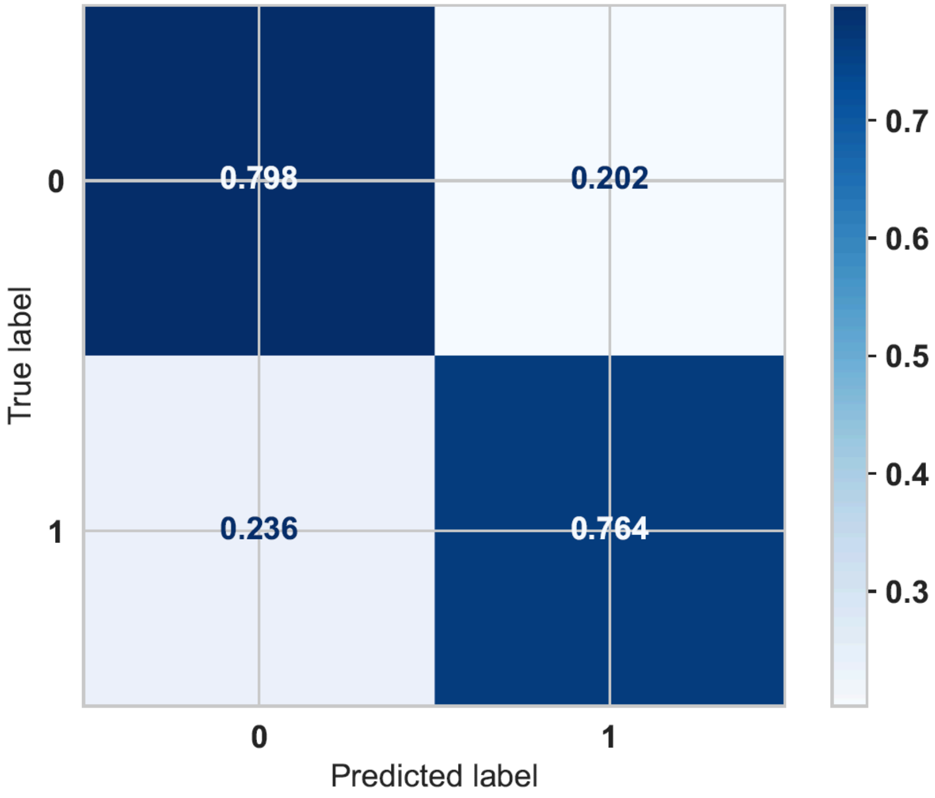

1.2.3. Alive 5 year

1.2.3.1. Random Forest

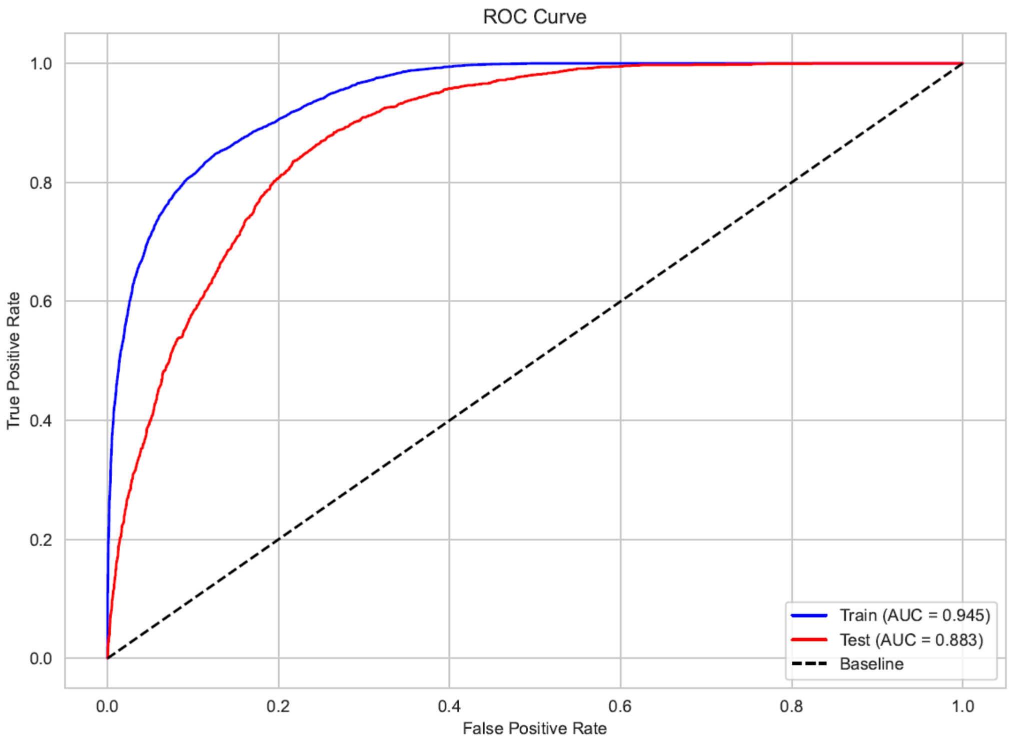

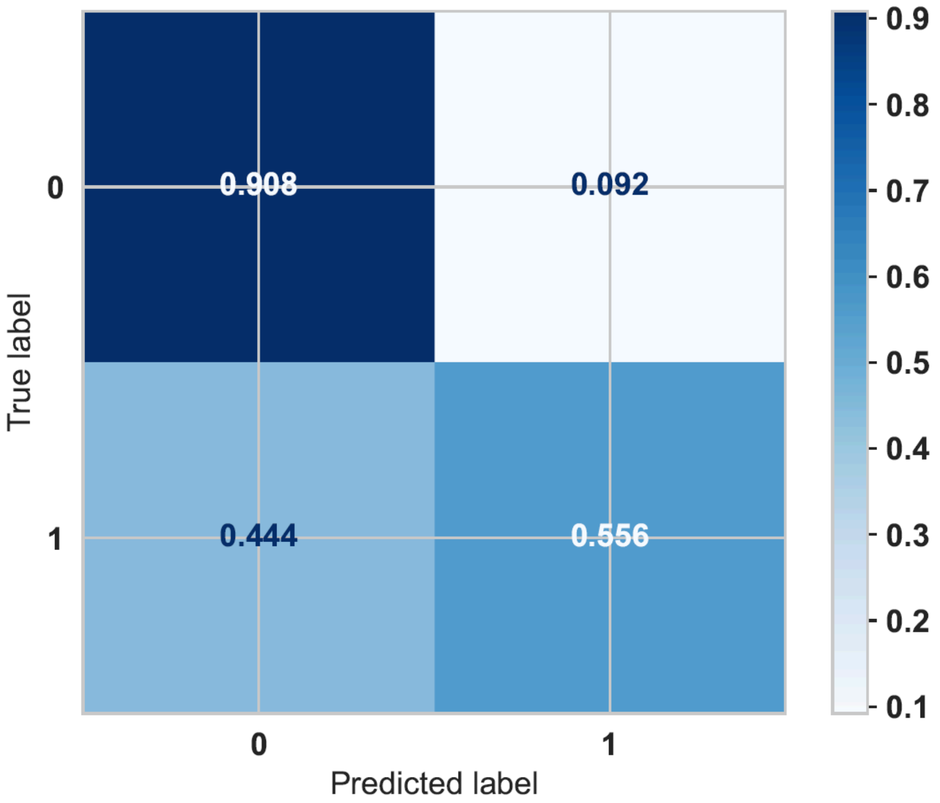

1.2.3.2. XGBoost

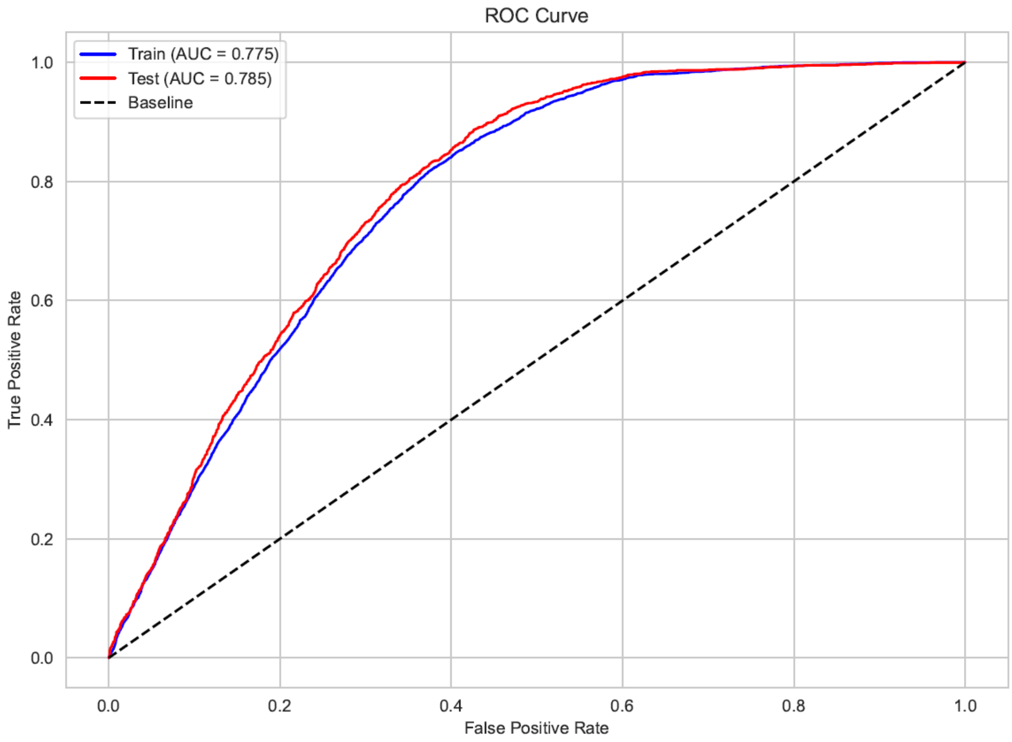

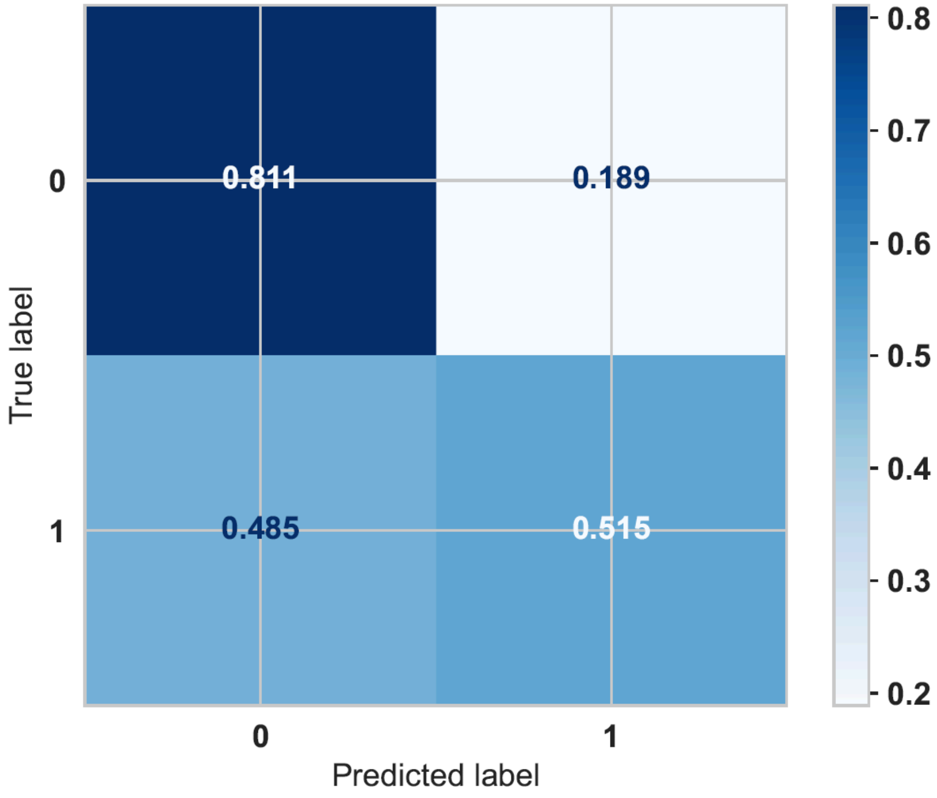

1.2.3.3. CatBoost

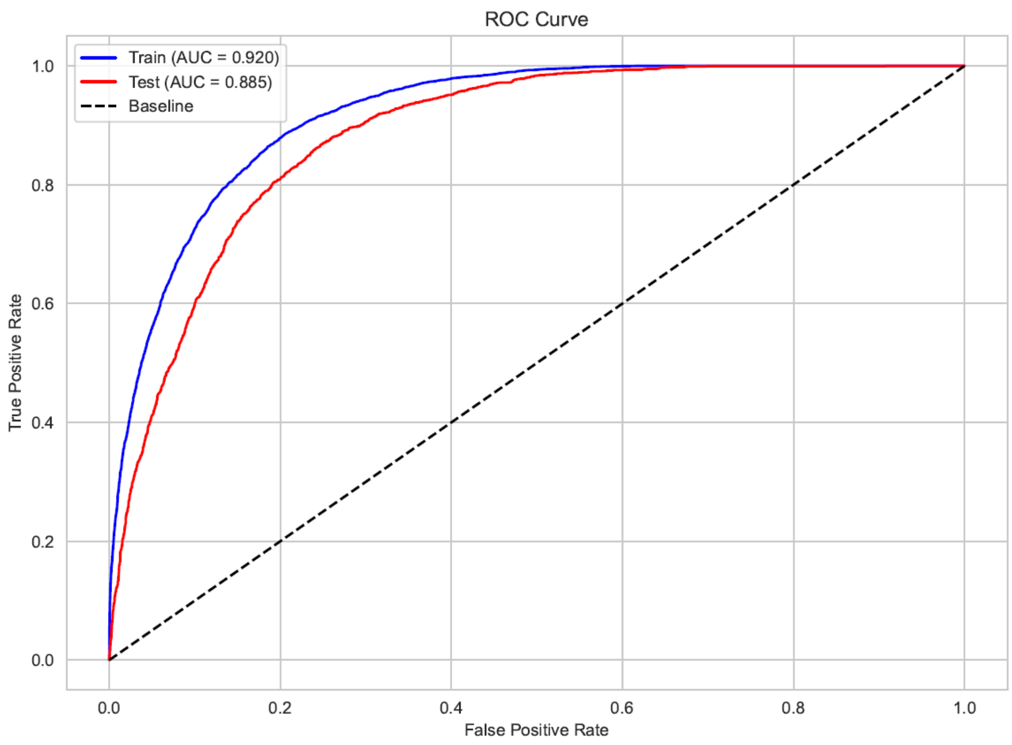

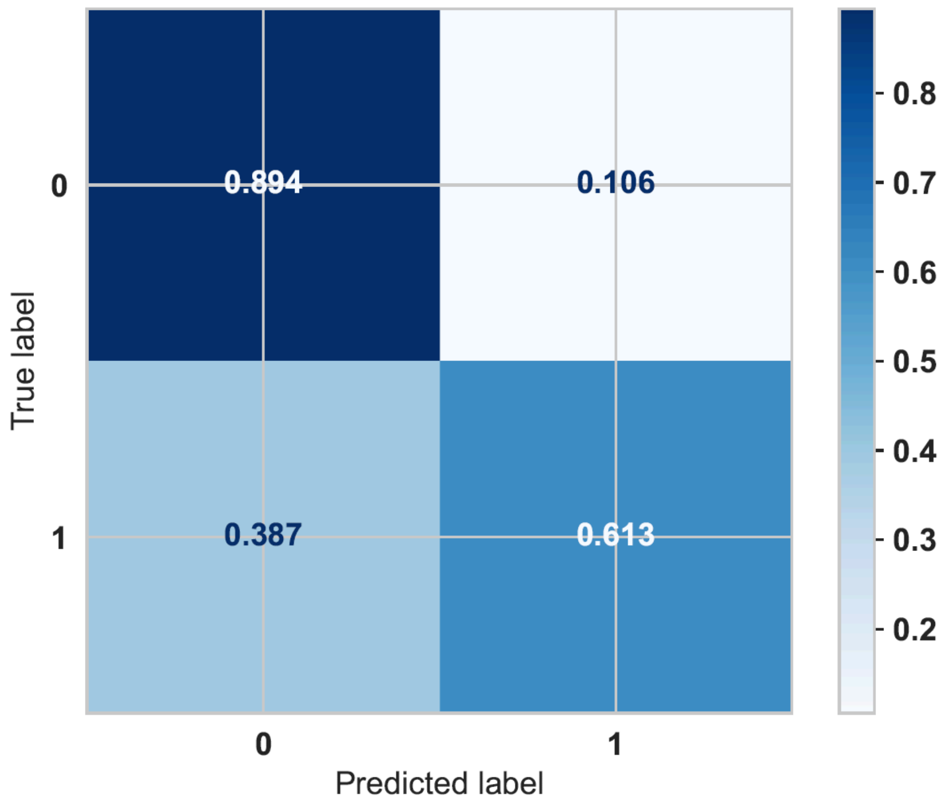

1.2.3.4. DecisionTreeClassifier

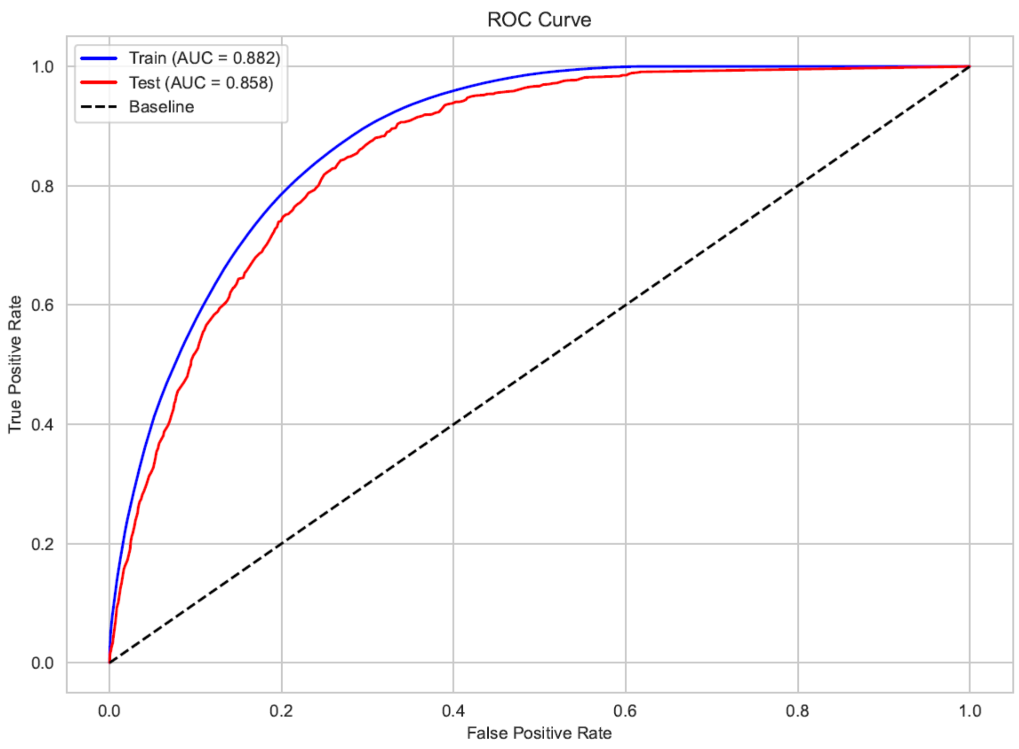

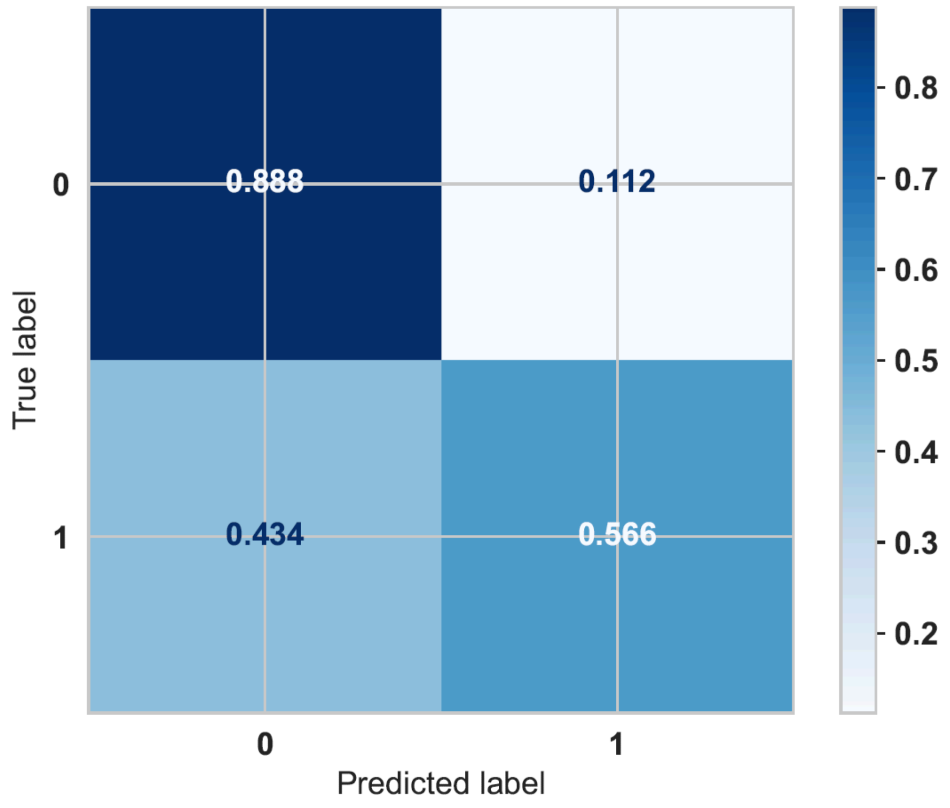

1.2.3.5. ExtraTreesClassifier

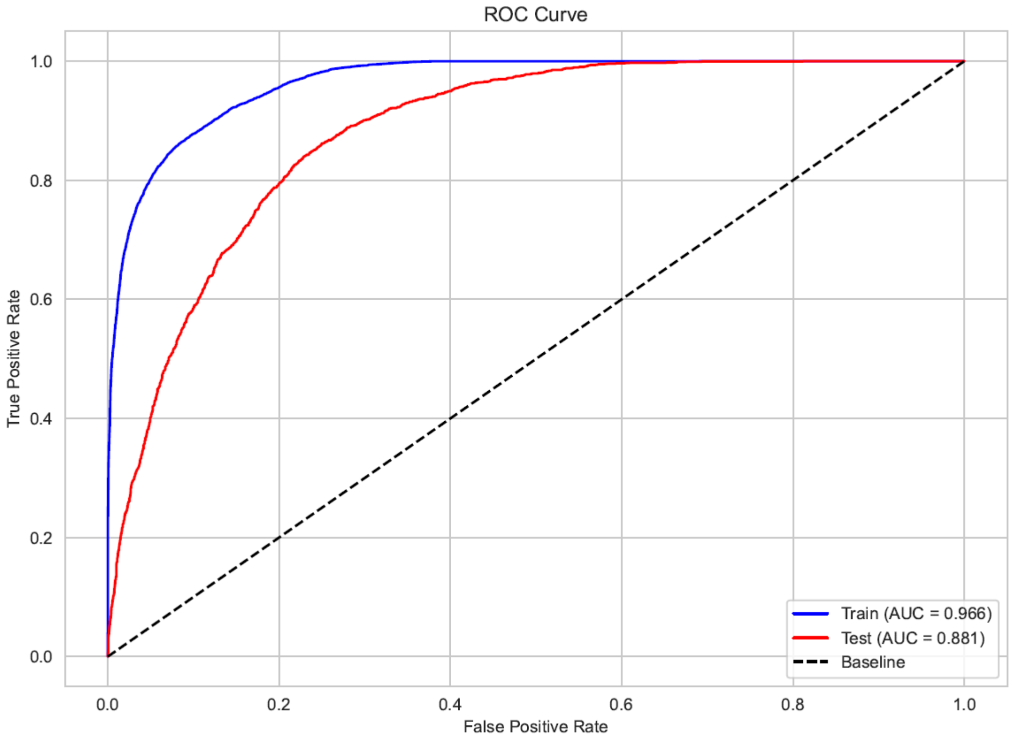

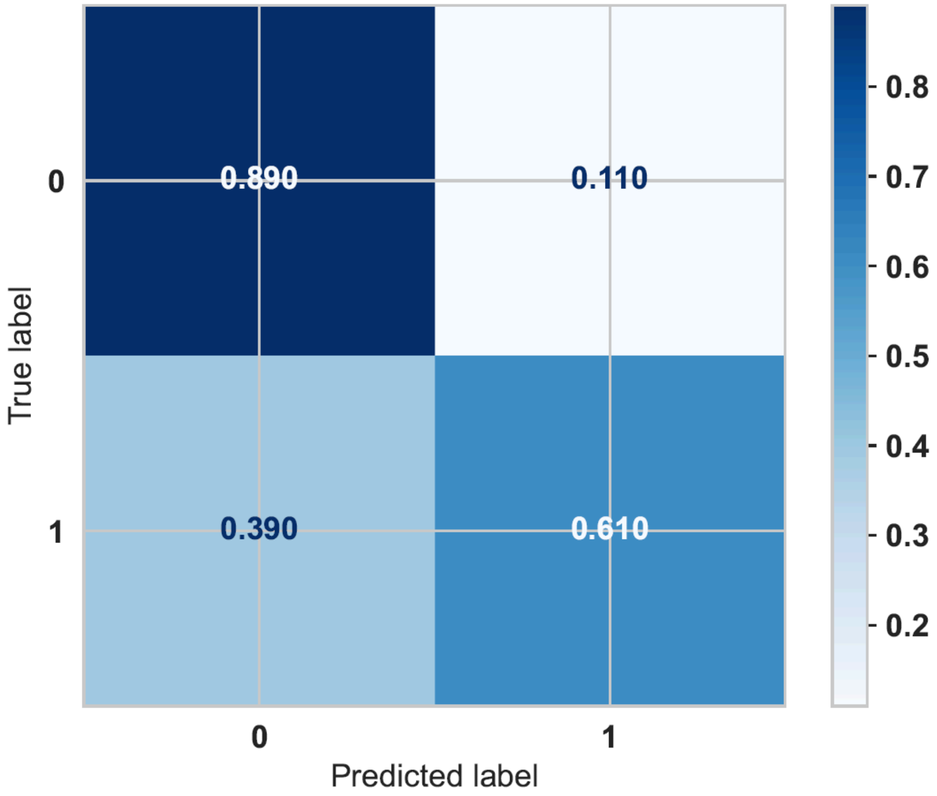

1.2.3.6. GradientBoosting

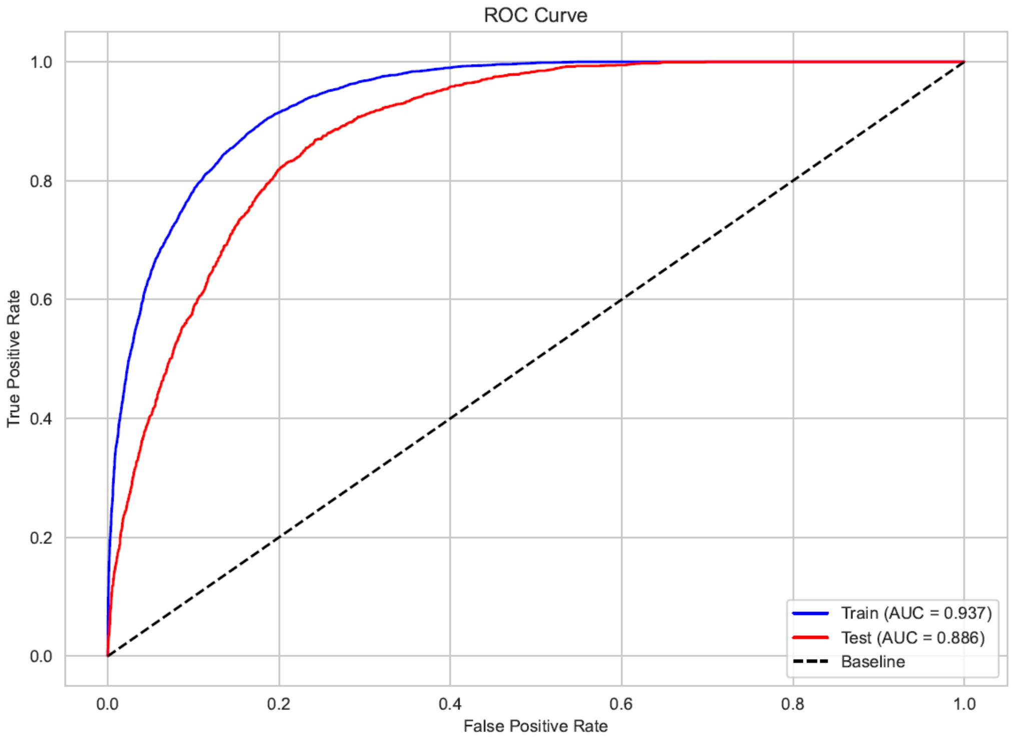
ROC Curve
True Positive Rate
False Positive Rate
0.0
0.2
0.4
0.6
0.8
1.0
Train (AUC = 0.937)
Test (AUC = 0.886)
Baseline

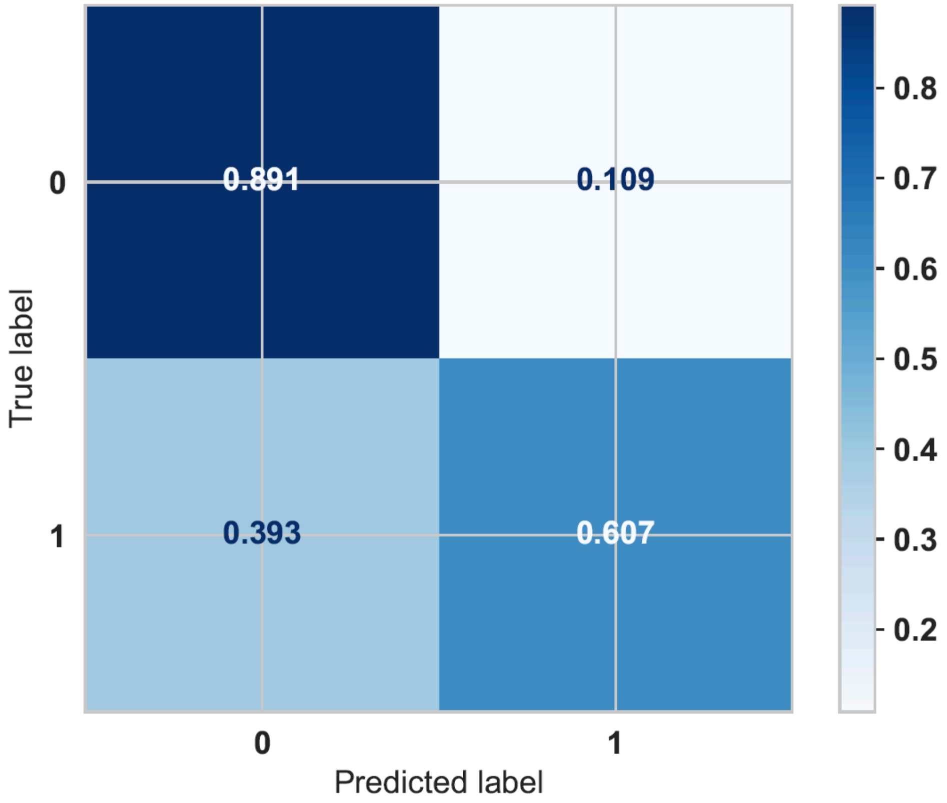
0.891
0.109
0.393
0.607
True label
Predicted label
0
1
0.8
0.7
0.6
0.5
0.4
0.3
0.2

1.2.3.7. KNN

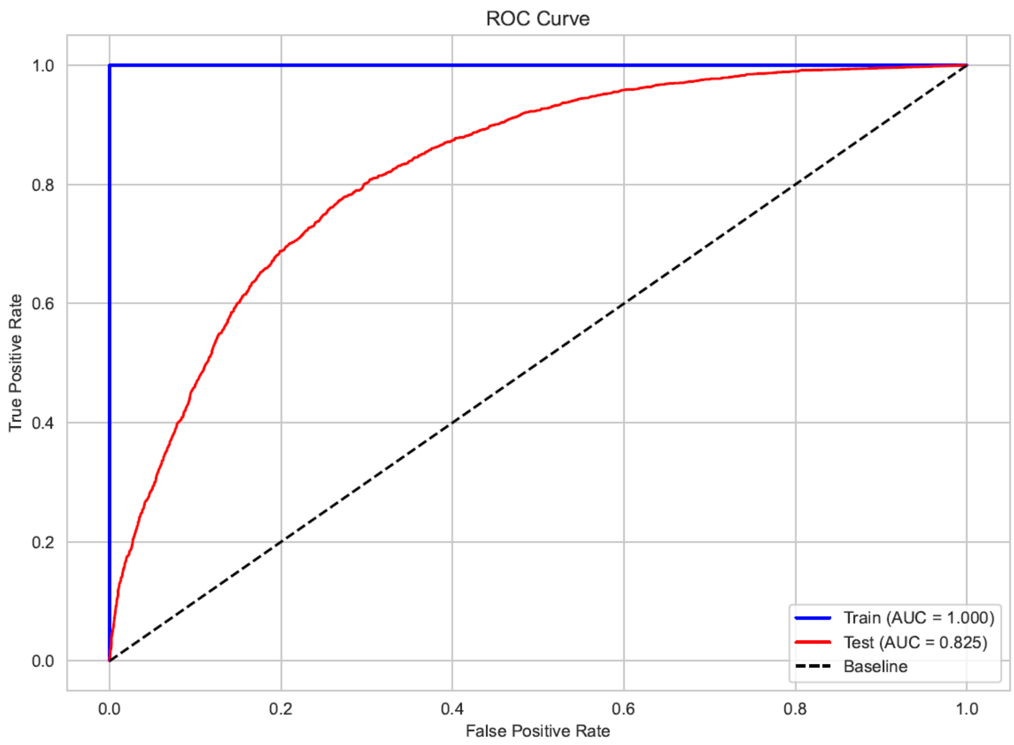

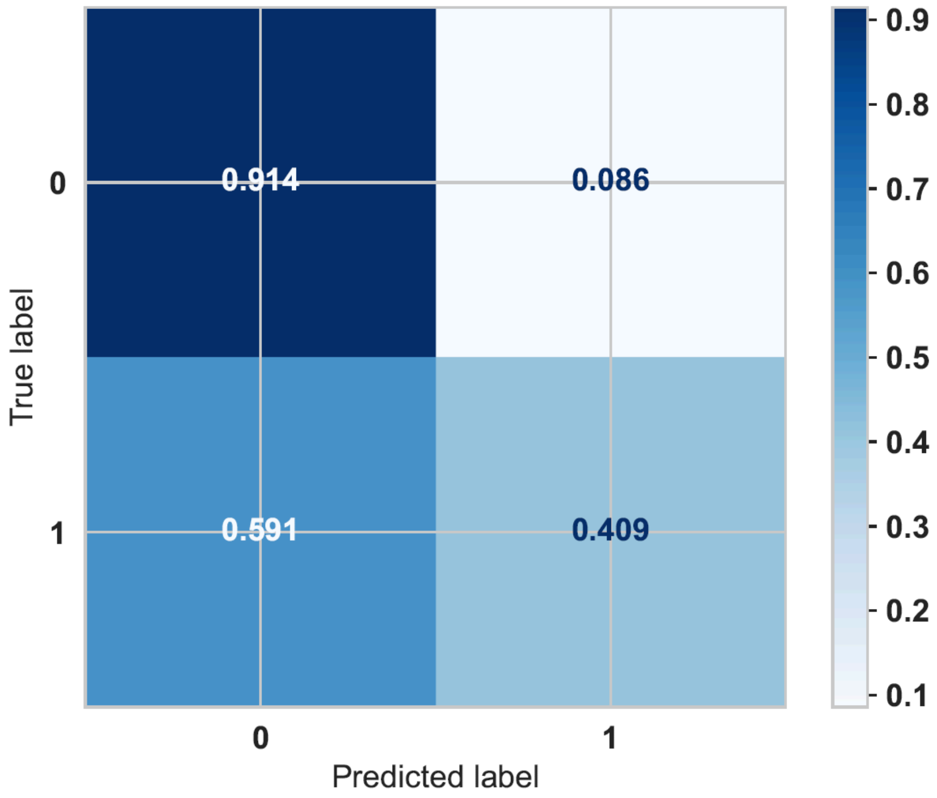

1.2.3.8. lightgbm

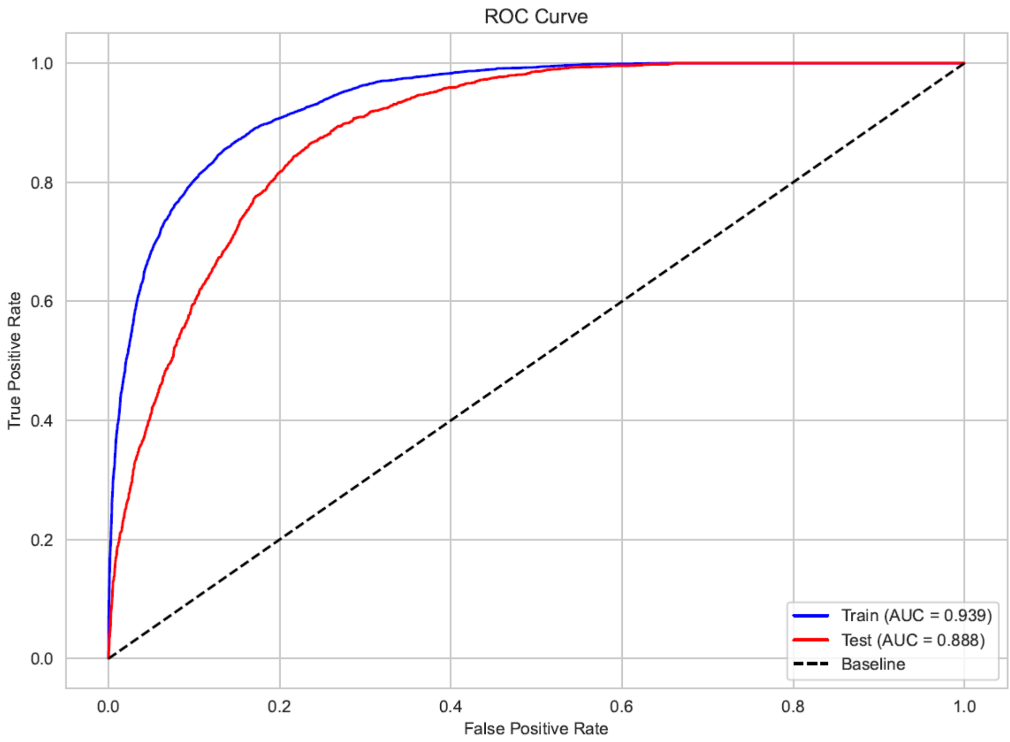

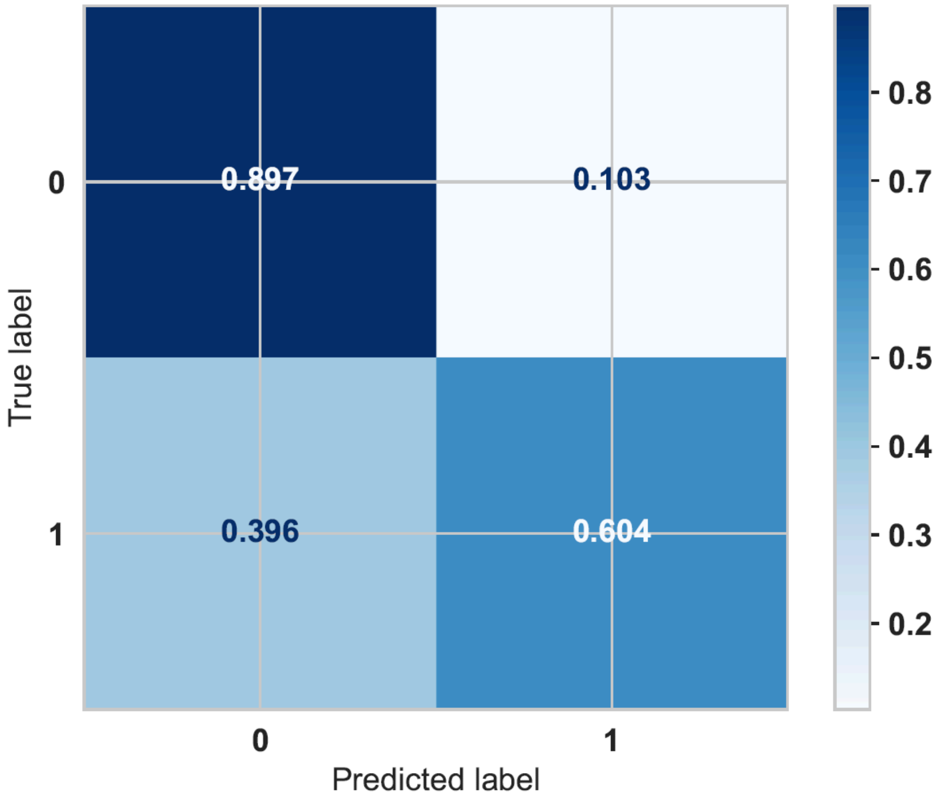

1.2.4. Cancer death

1.2.4.1. Random Forest

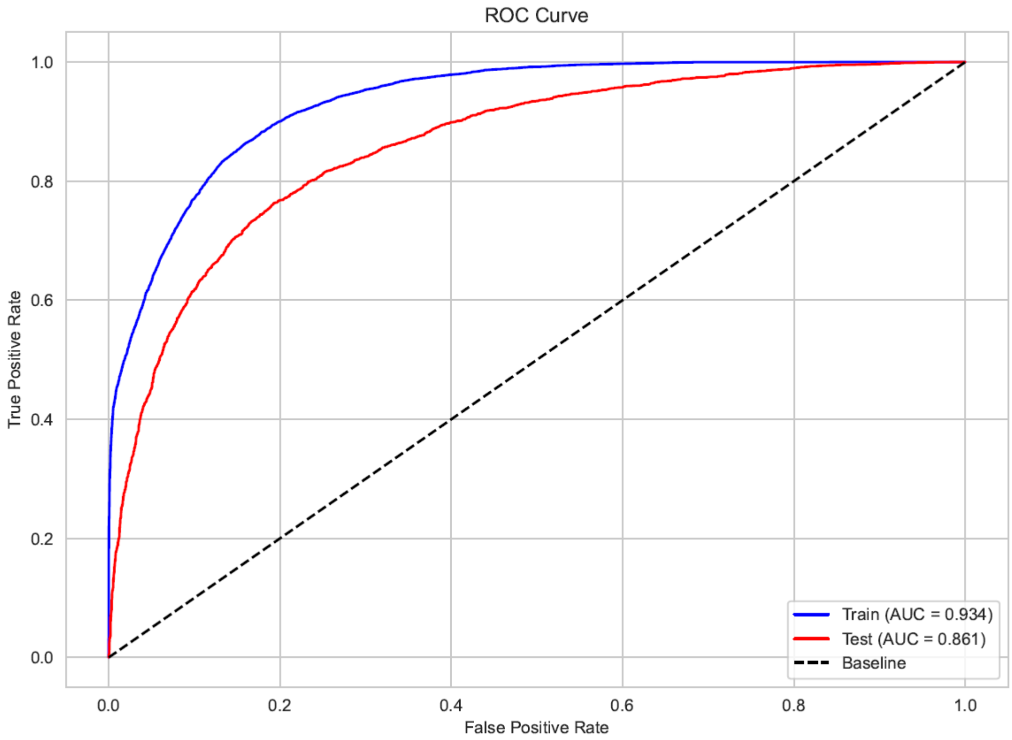

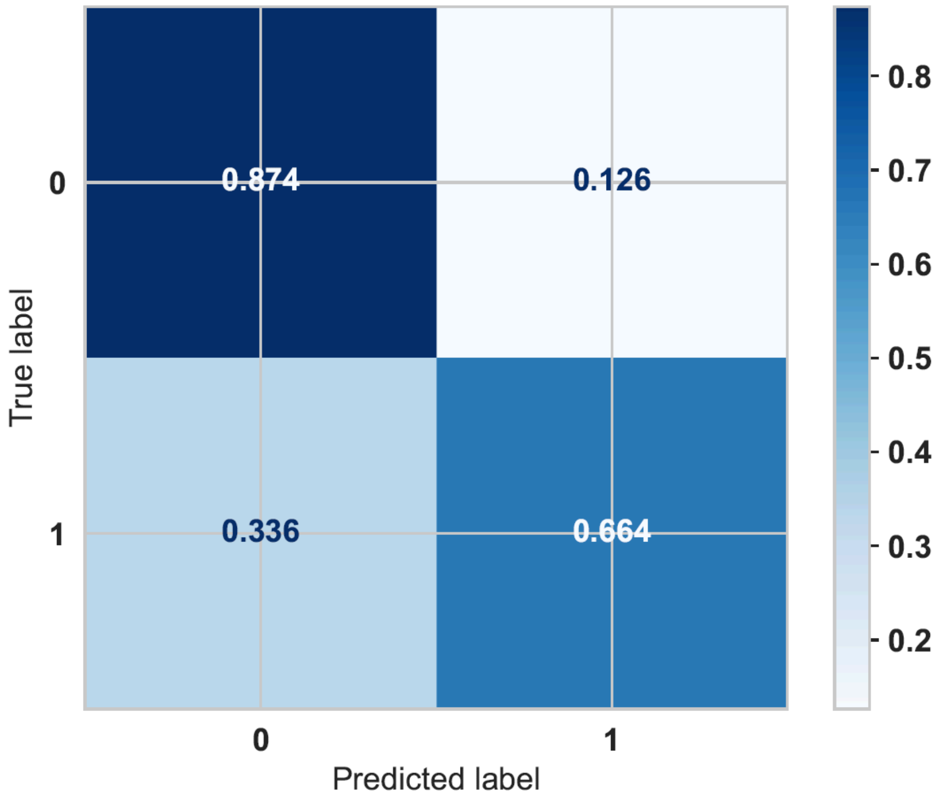

1.2.4.2. XGBoost

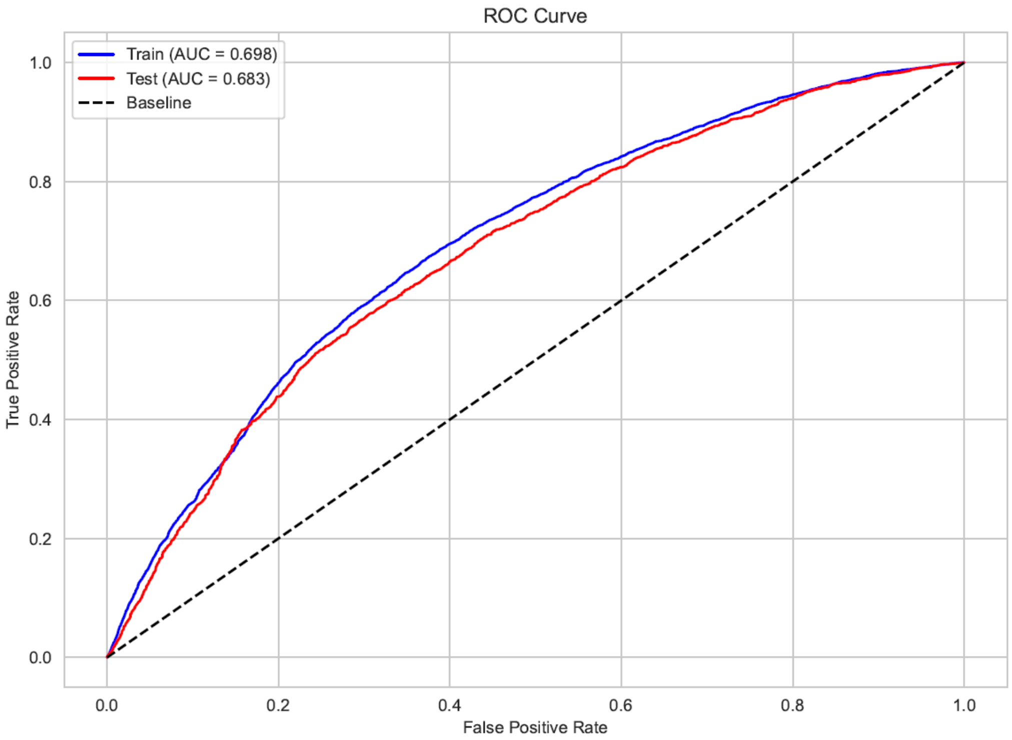

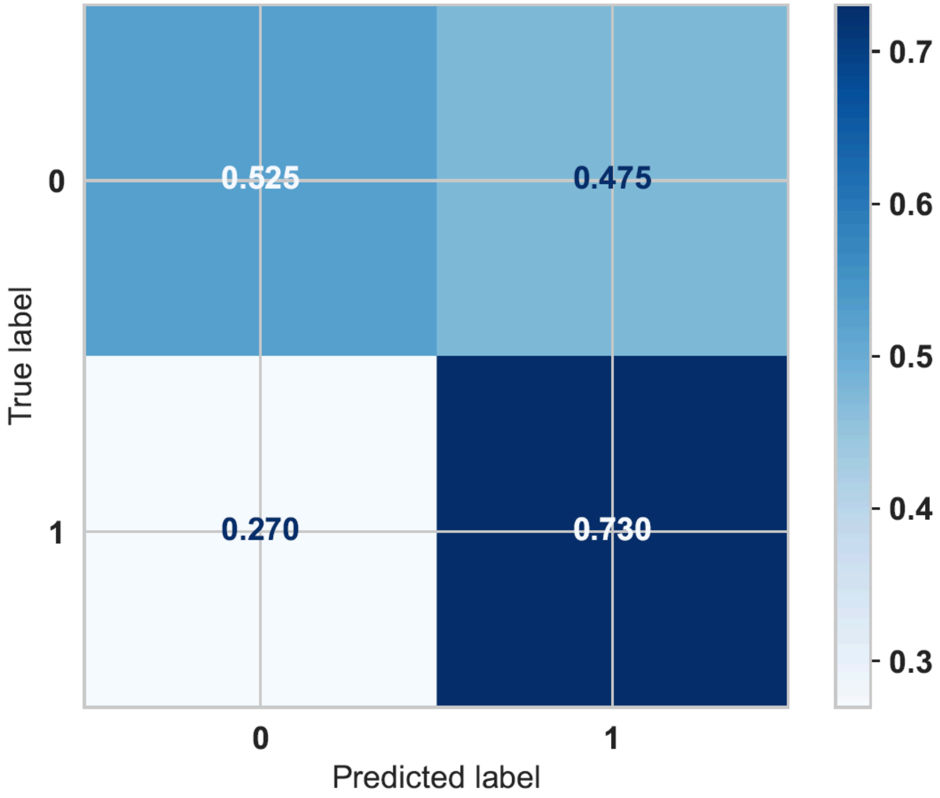

1.2.4.3. CatBoost

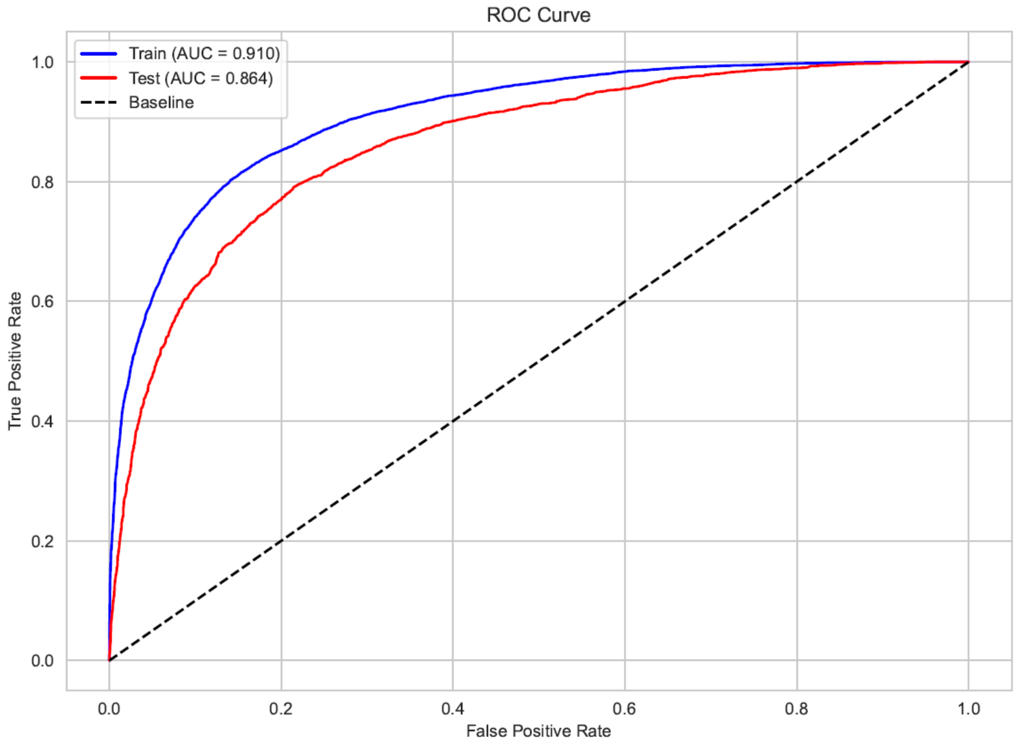

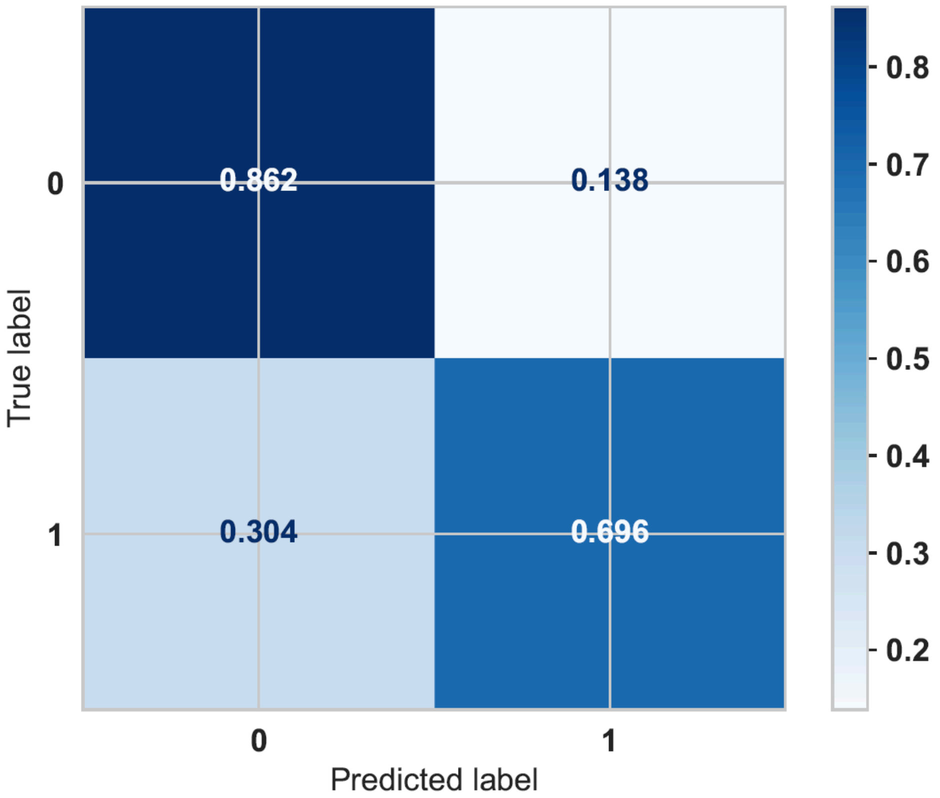

1.2.4.4. DecisionTreeClassifier

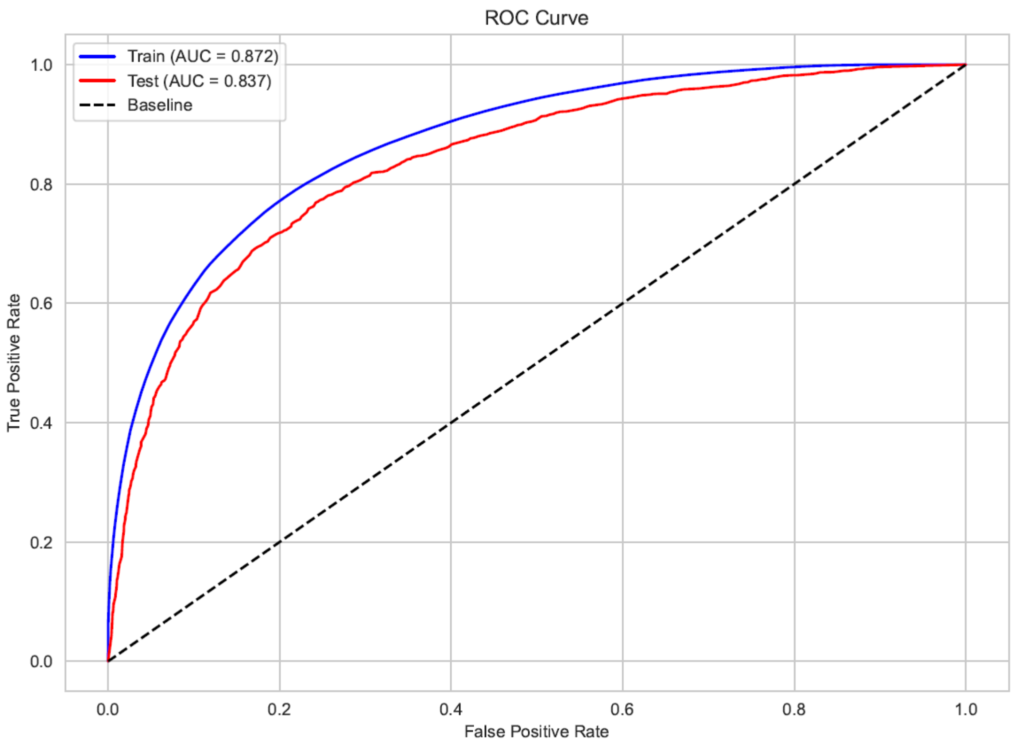

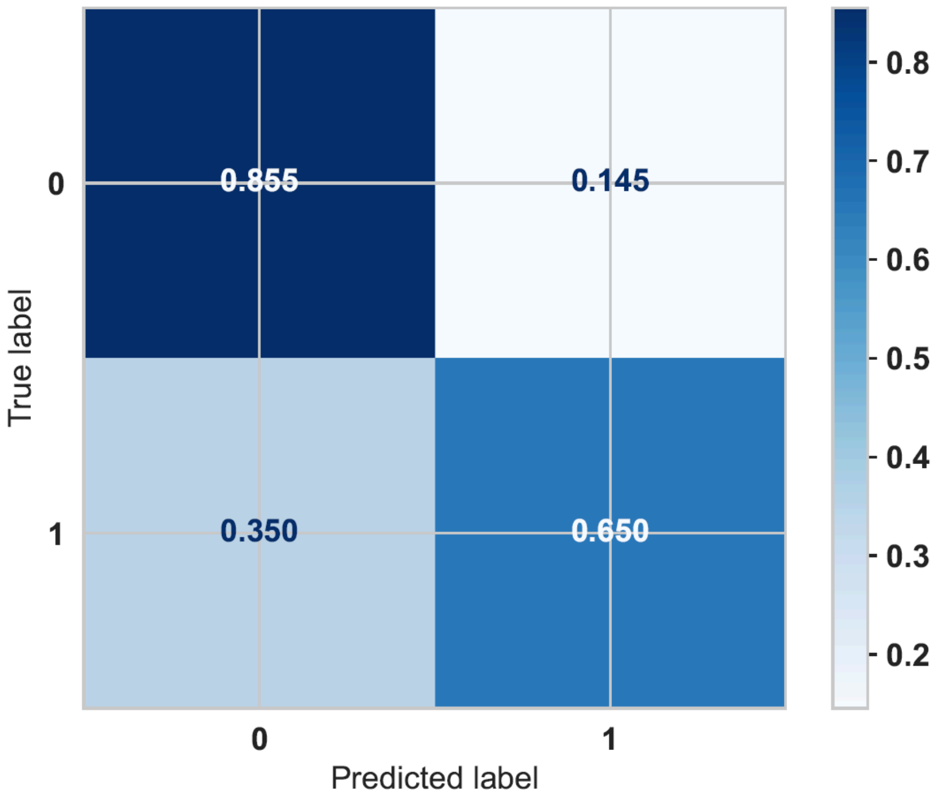

1.2.4.5. ExtraTreesClassifier

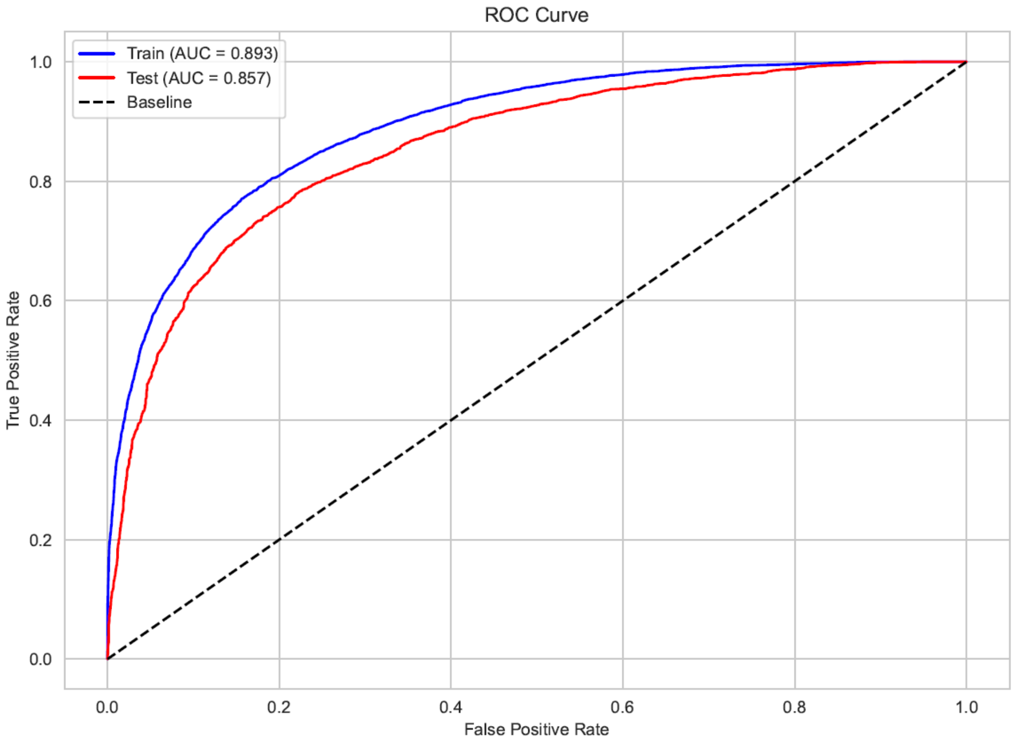

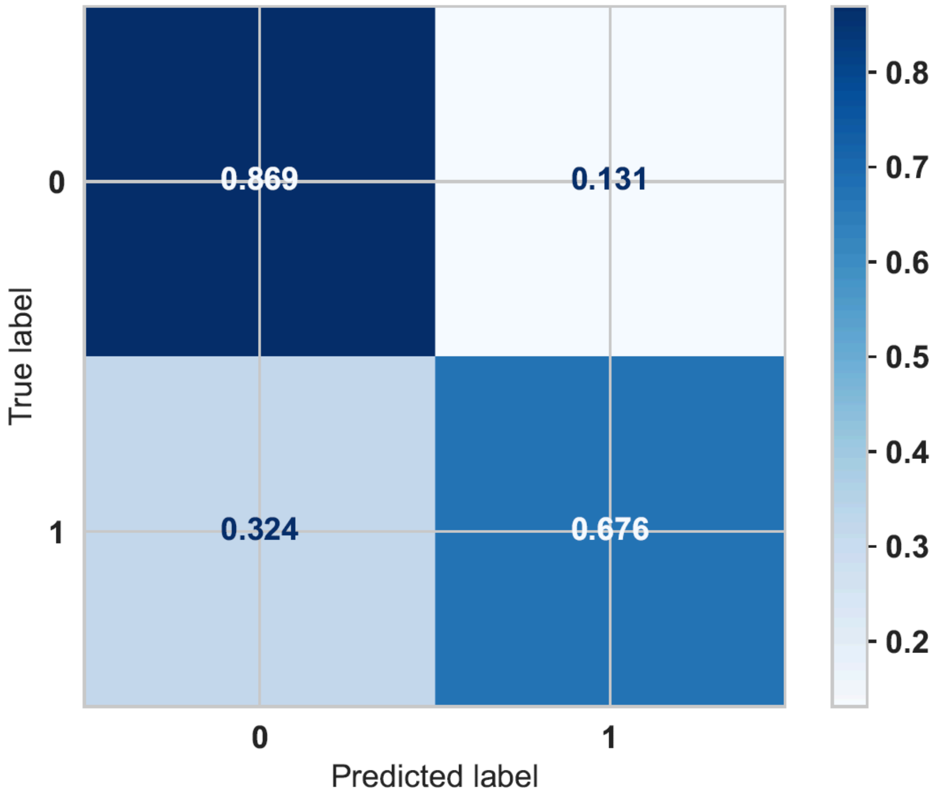

1.2.4.6. GradientBoosting

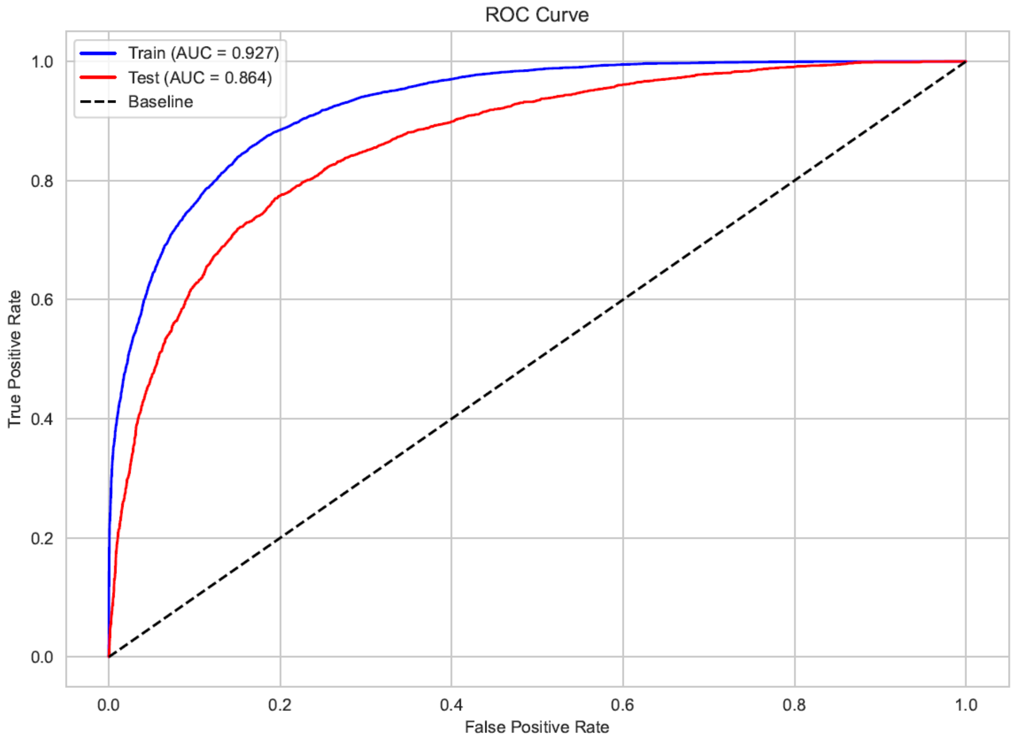
ROC Curve
Train (AUC = 0.927)
Test (AUC = 0.864)
Baseline
True Positive Rate
False Positive Rate
0.0
0.2
0.4
0.6
0.8
1.0

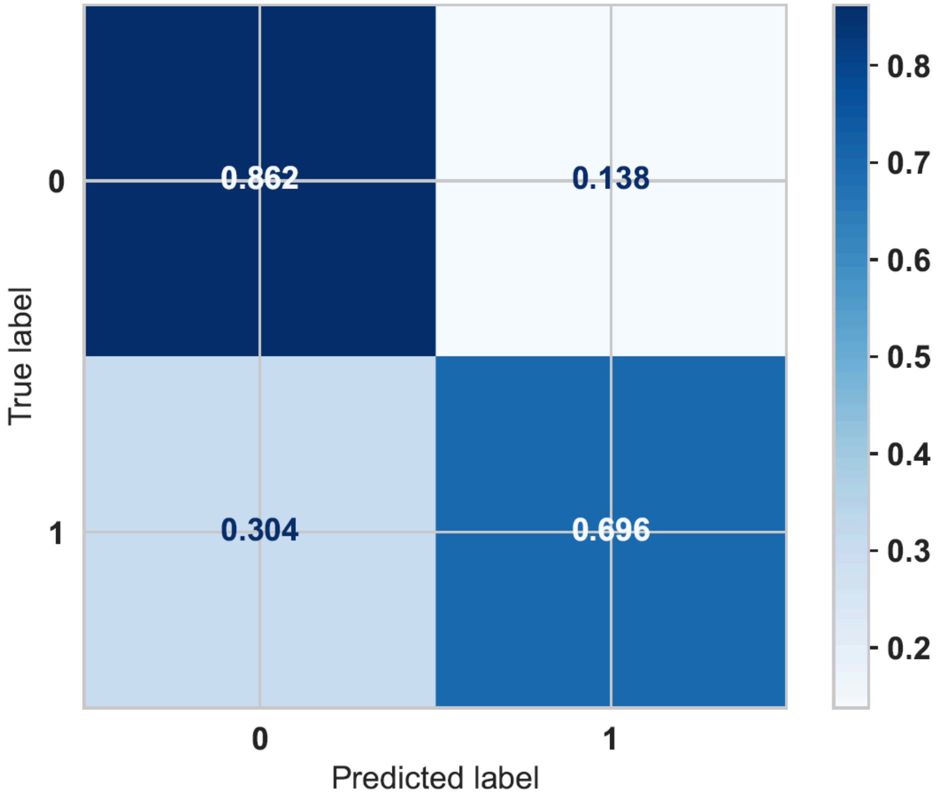
0.862
0.138
0.304
0.696
True label
Predicted label
0
1
0.8
0.7
0.6
0.5
0.4
0.3
0.2

1.2.4.7. KNN

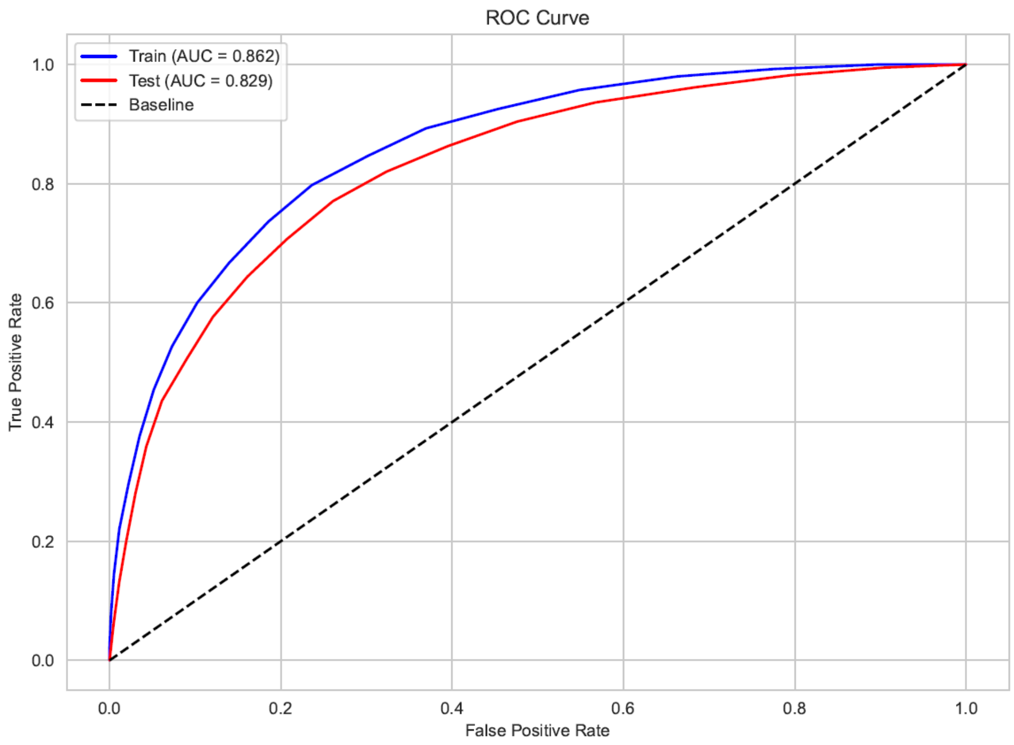

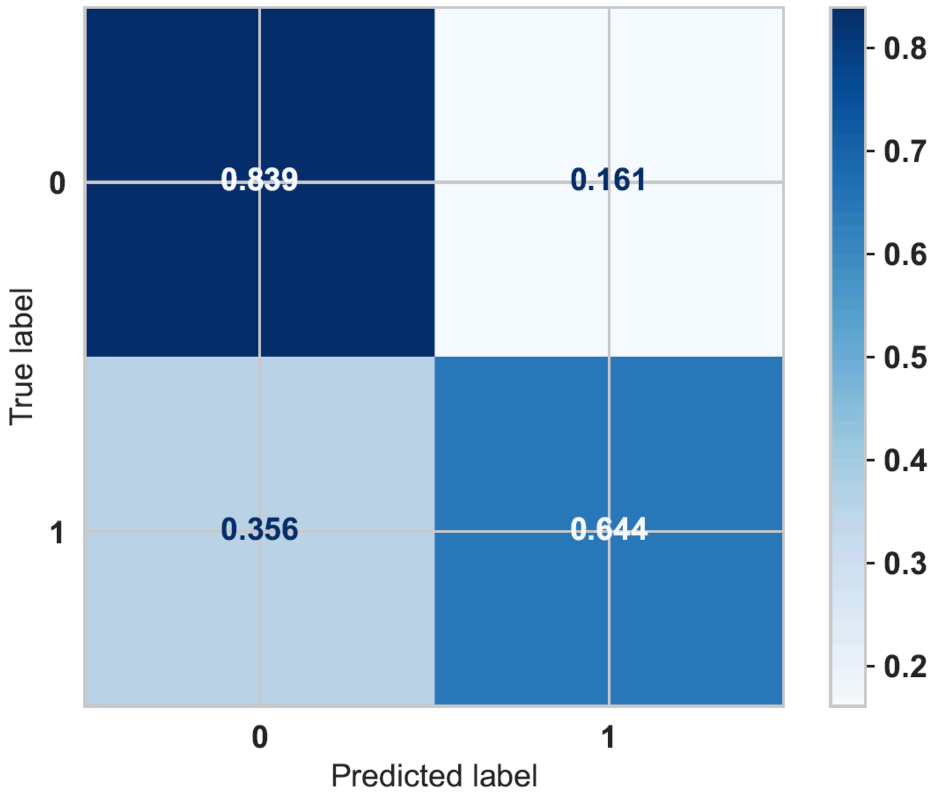

1.2.4.8. lightgbm

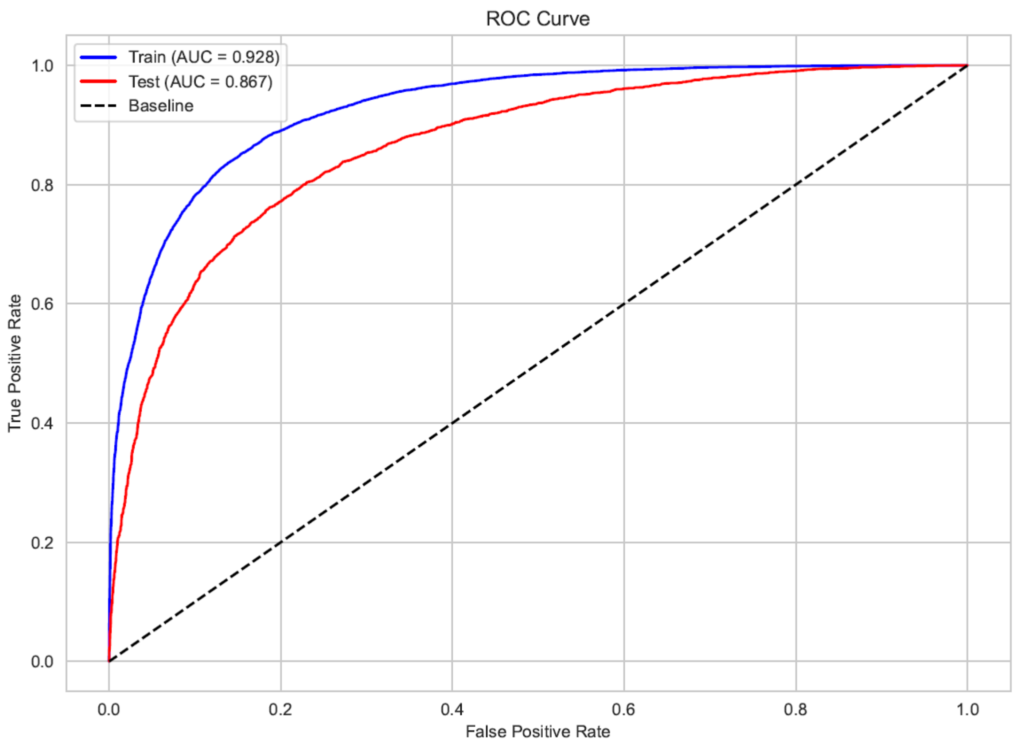

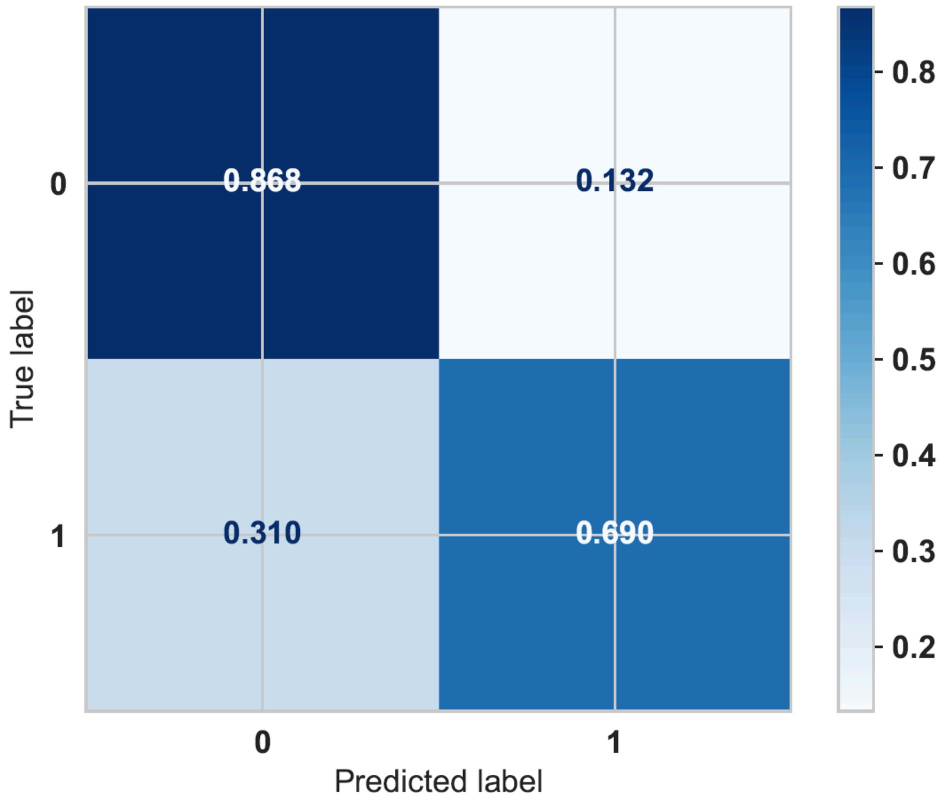

1.2.5. Overall death

1.2.5.1. Random Forest

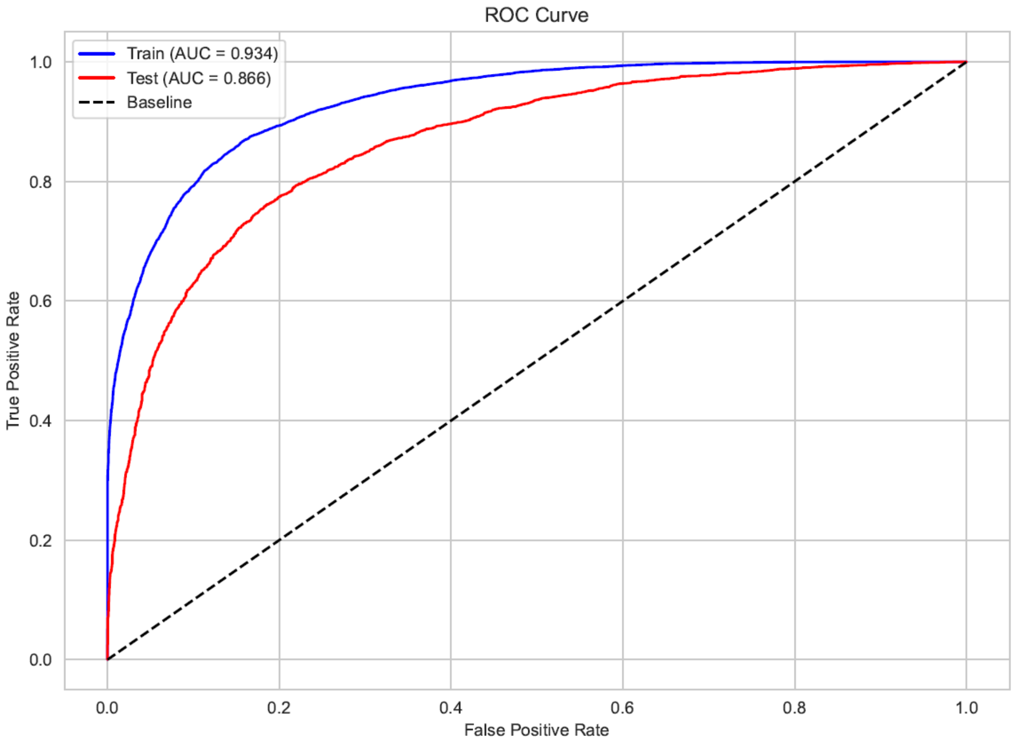

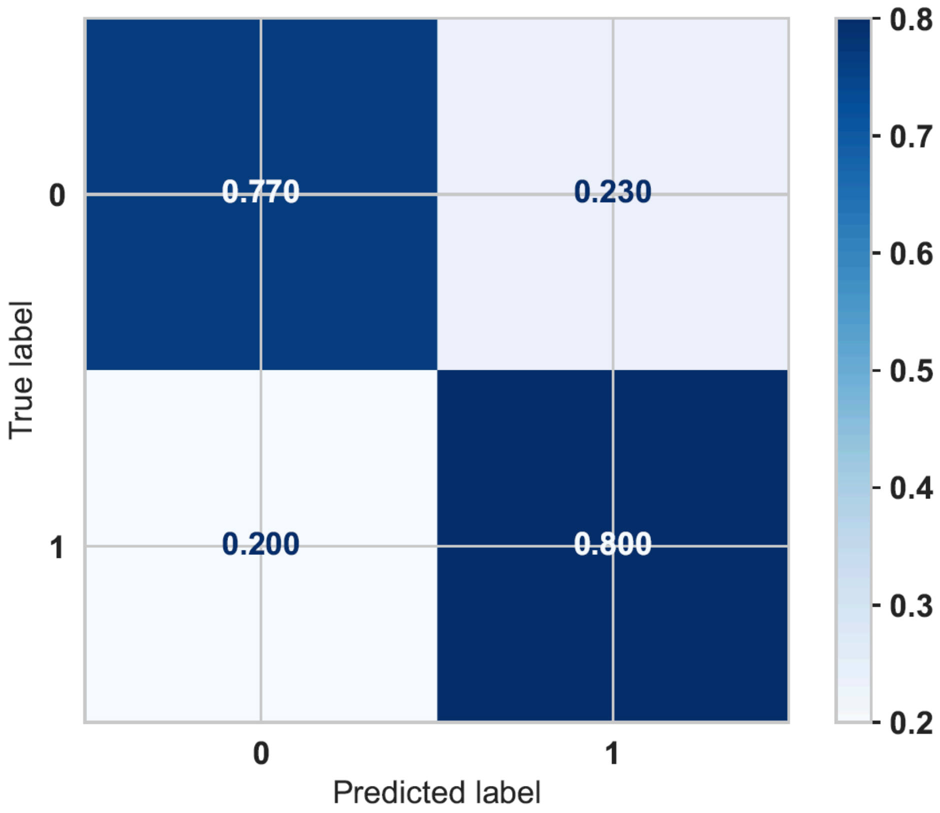

1.2.5.2. XGBoost

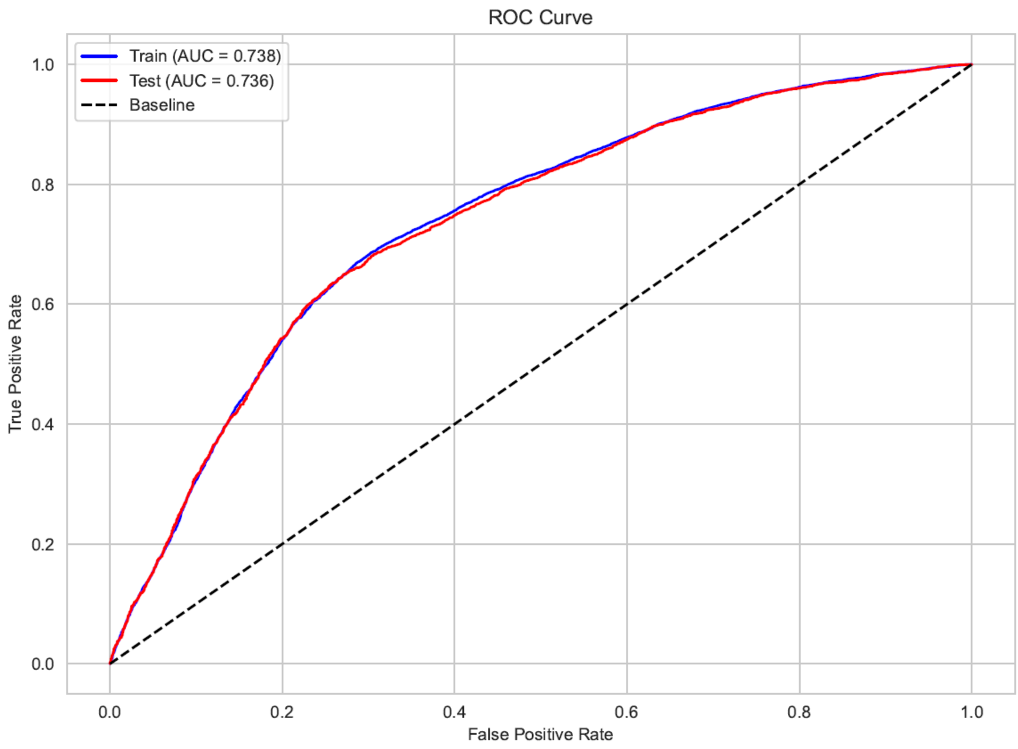

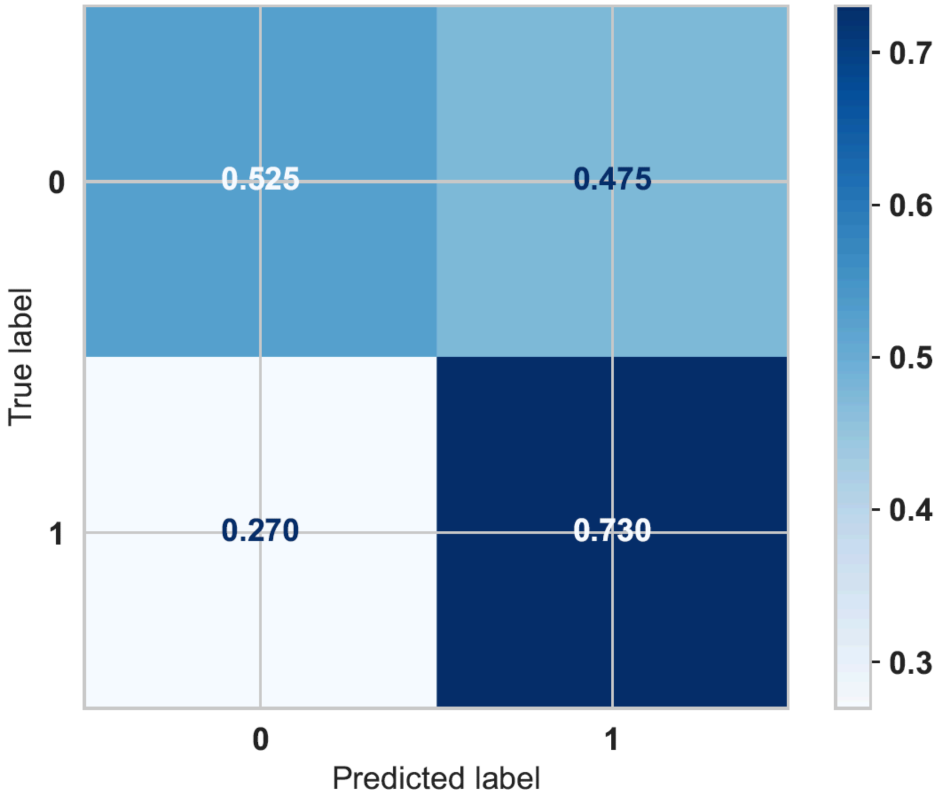

1.2.5.3. CatBoost

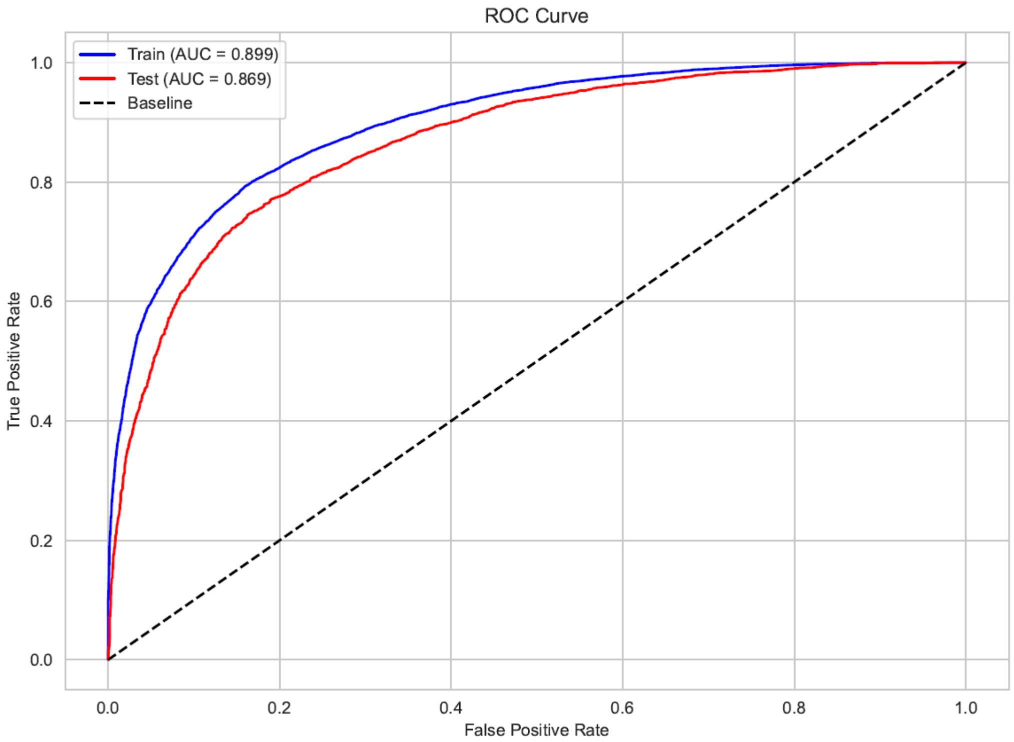

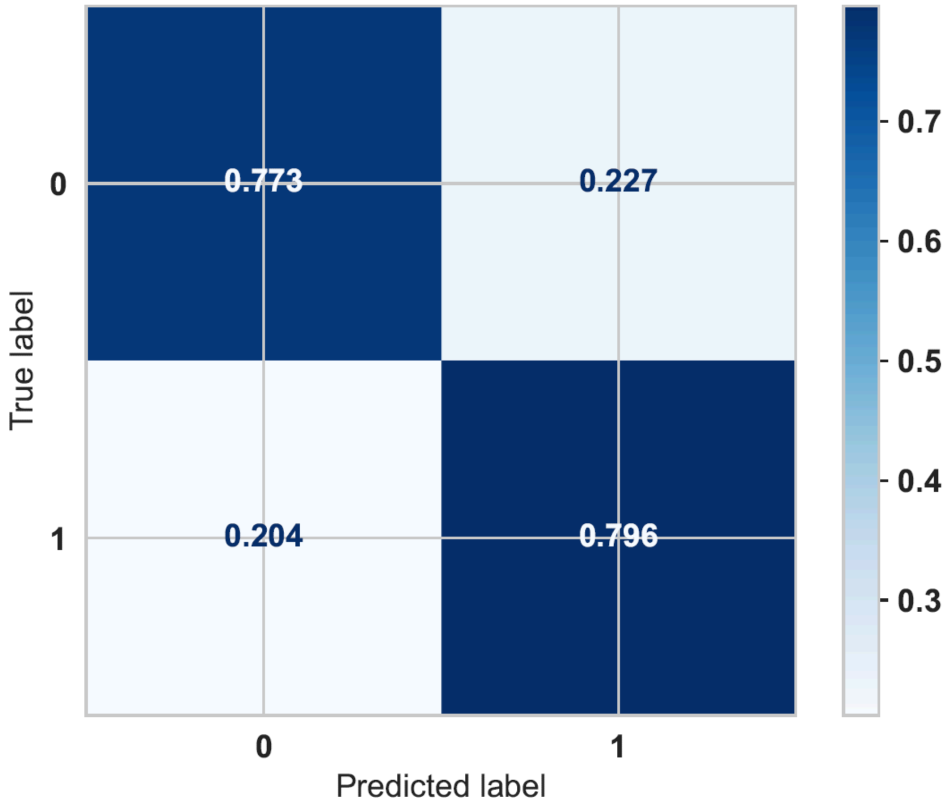

1.2.5.4. DecisionTreeClassifier

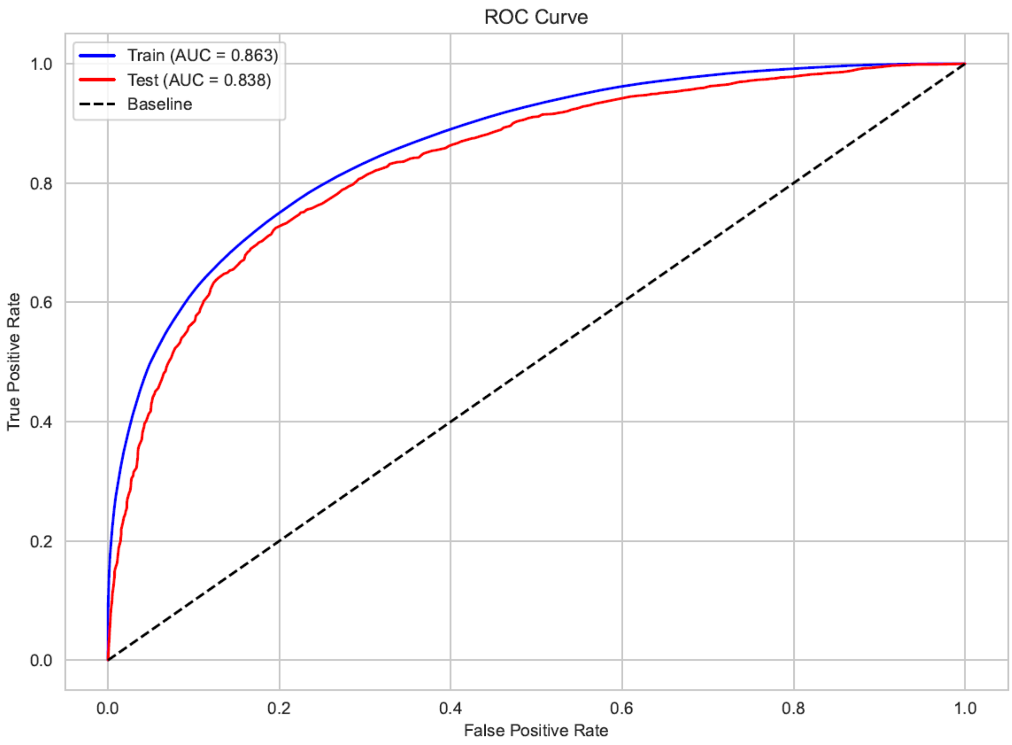

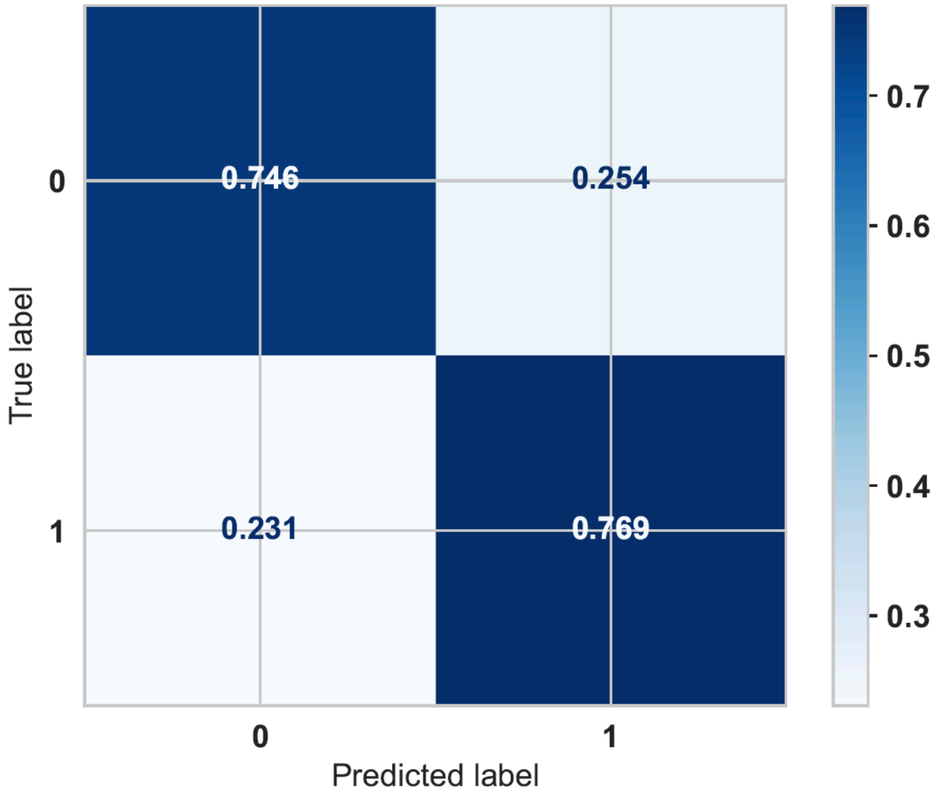

1.2.5.5. ExtraTreesClassifier

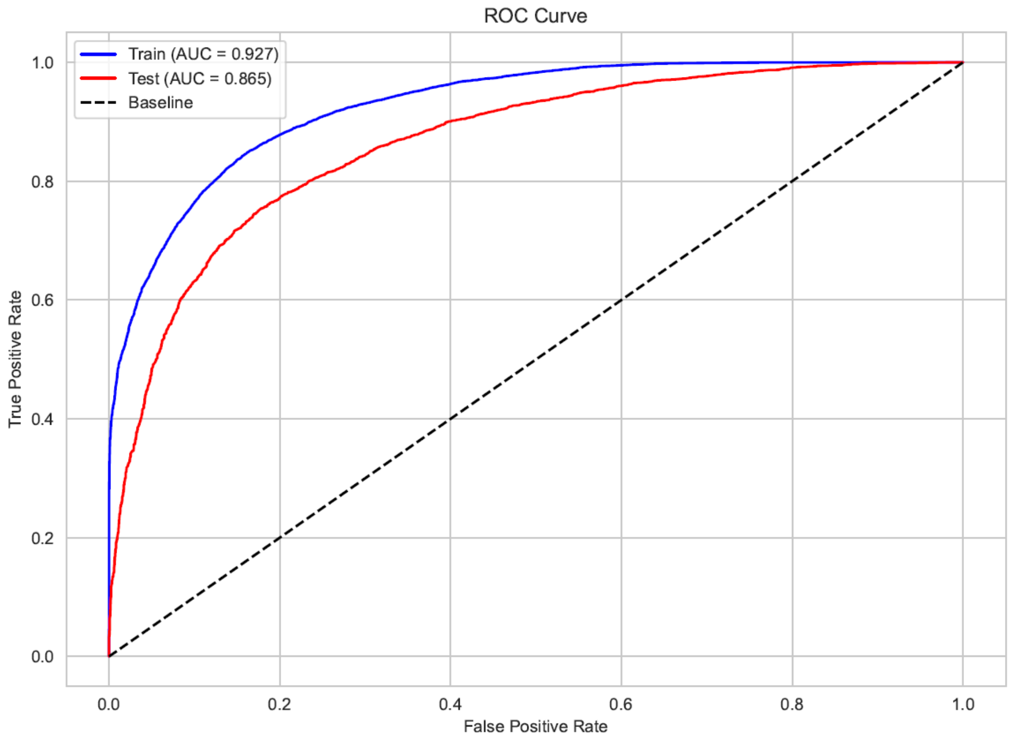

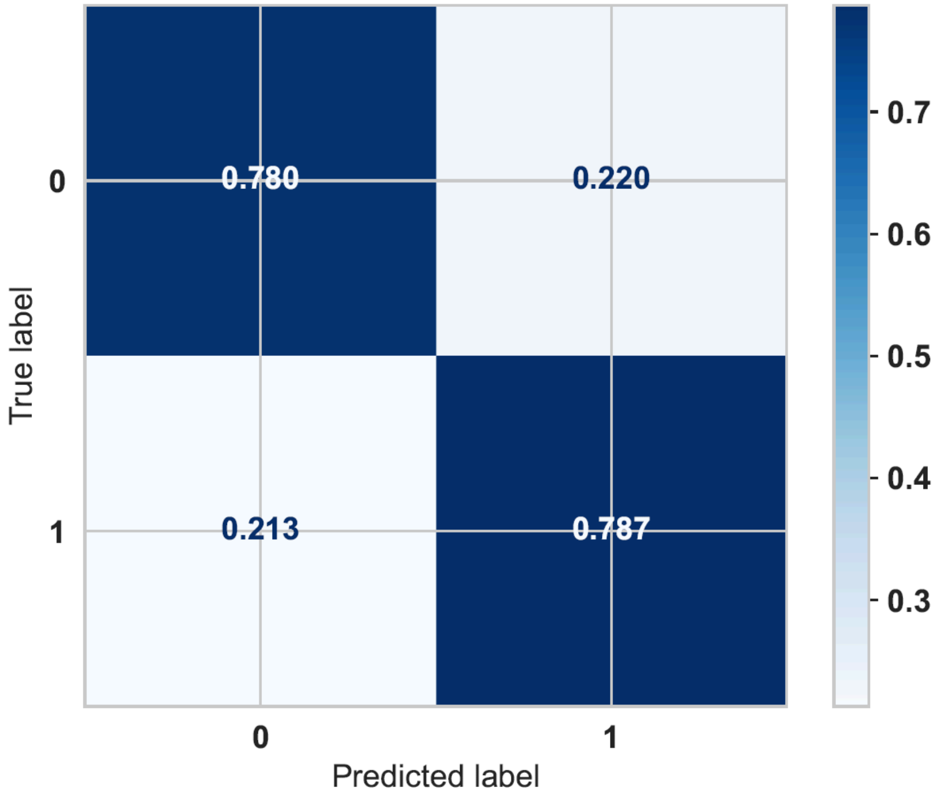

1.2.5.6. GradientBoosting

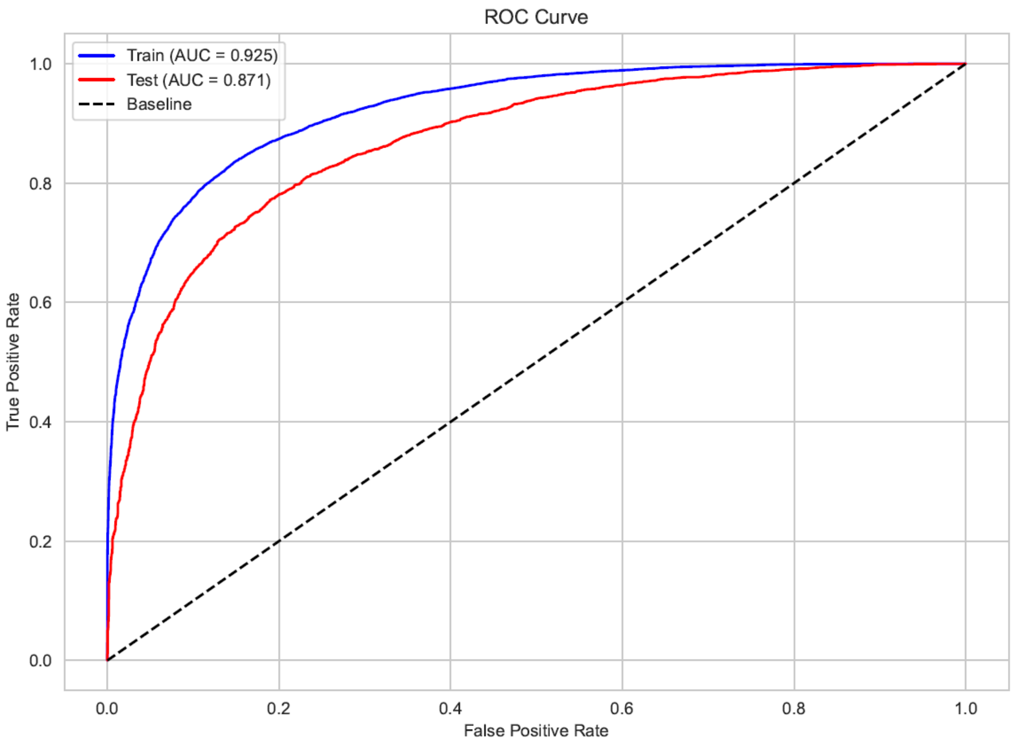
ROC Curve
Train (AUC = 0.925)
Test (AUC = 0.871)
Baseline
True Positive Rate
False Positive Rate
0.0
0.2
0.4
0.6
0.8
1.0

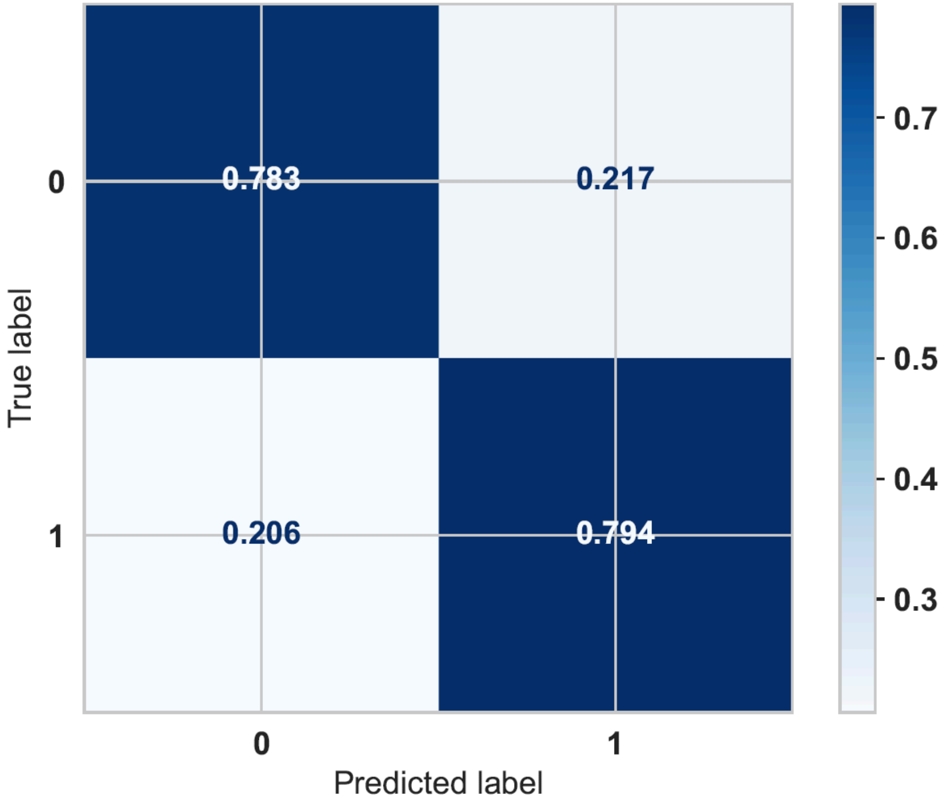
0.783
0.217
0.206
0.794
True label
Predicted label
0
1
0.7
0.6
0.5
0.4
0.3

1.2.5.7. KNN

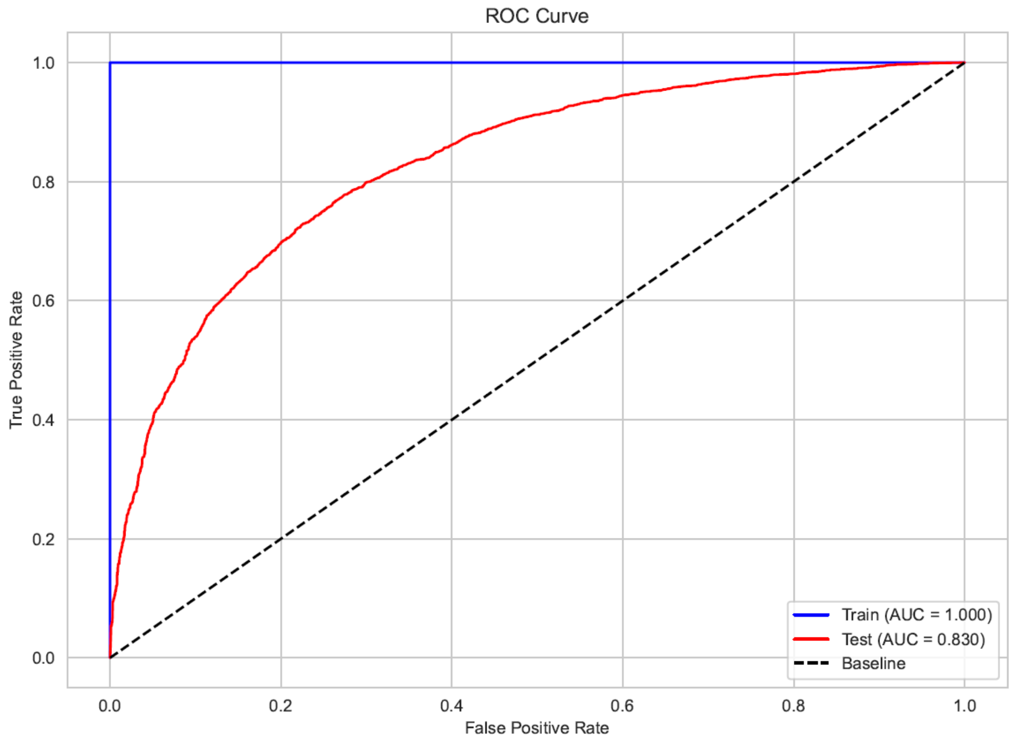

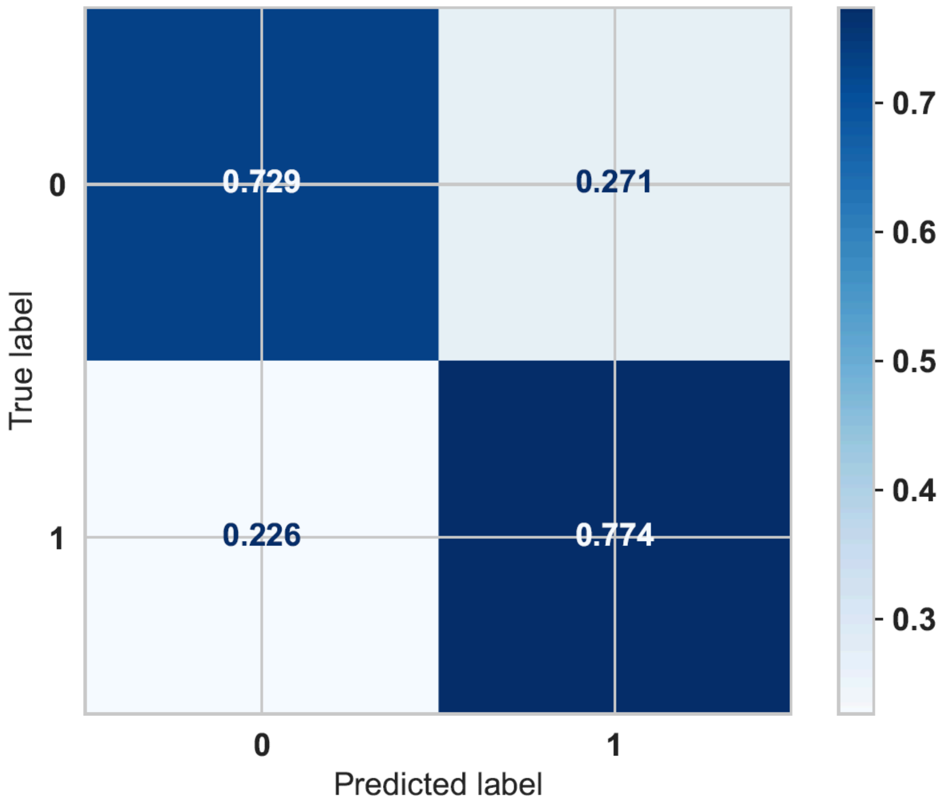

1.2.5.8. lightgbm

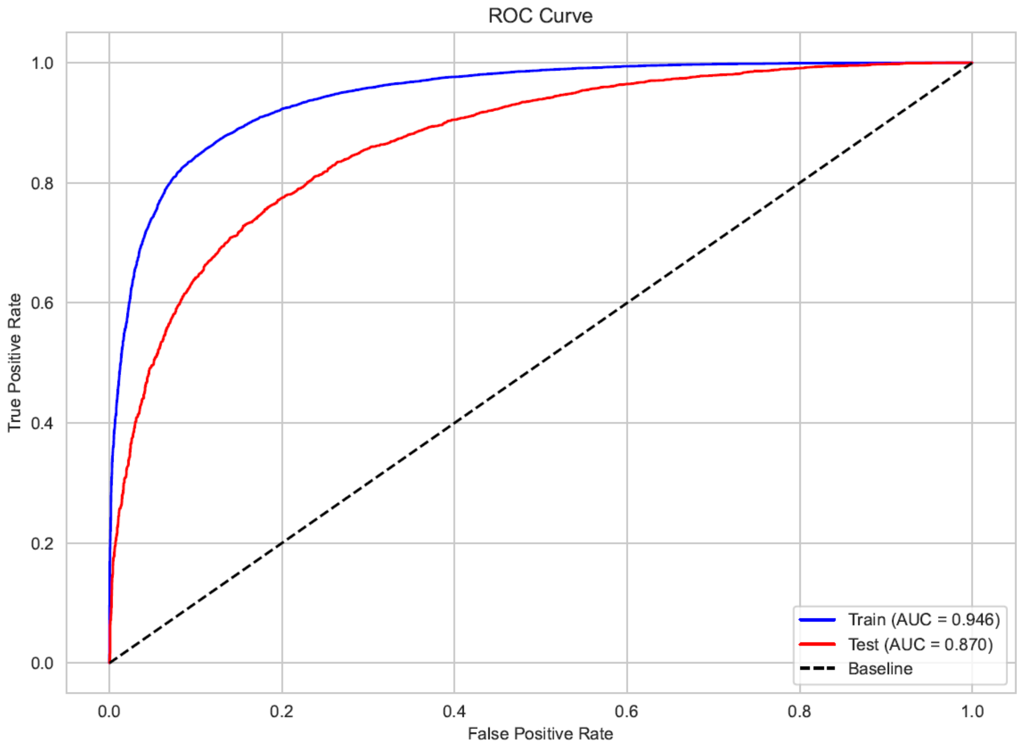
ROC Curve
True Positive Rate
False Positive Rate
0.0
0.2
0.4
0.6
0.8
1.0
Train (AUC = 0.946)
Test (AUC = 0.870)
Baseline

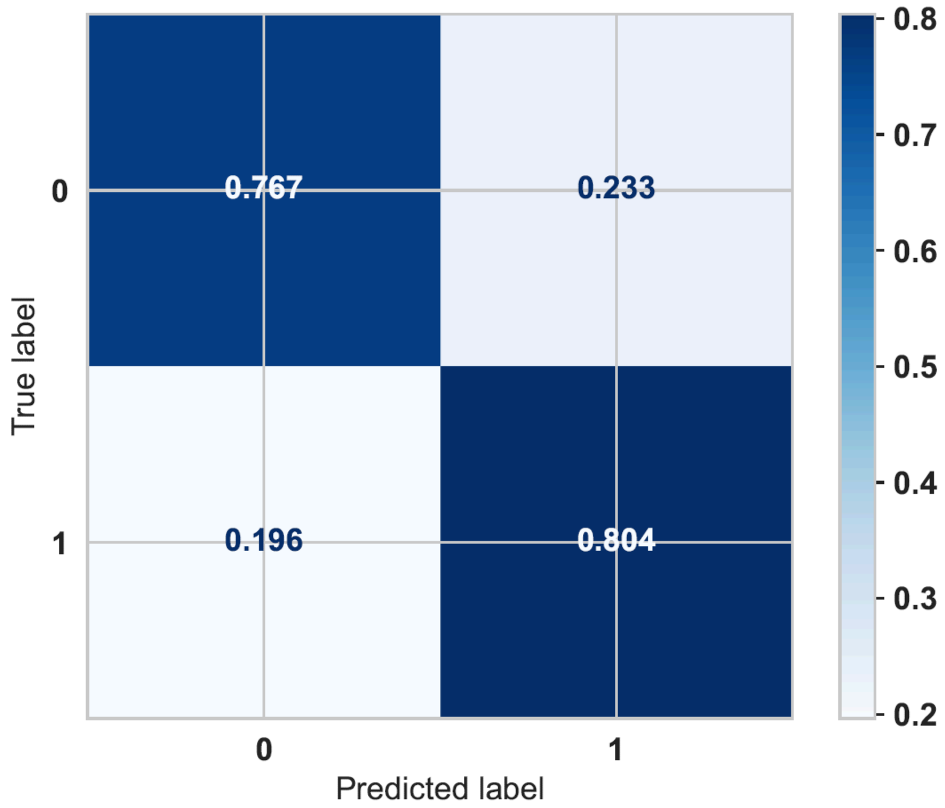
True label
Predicted label
0
1
0.767
0.233
0.196
0.804
0.8
0.7
0.6
0.5
0.4
0.3
0.2

1.3. RayTune

1.3.1. Alive 1 year

1.3.1.1. Random Forest

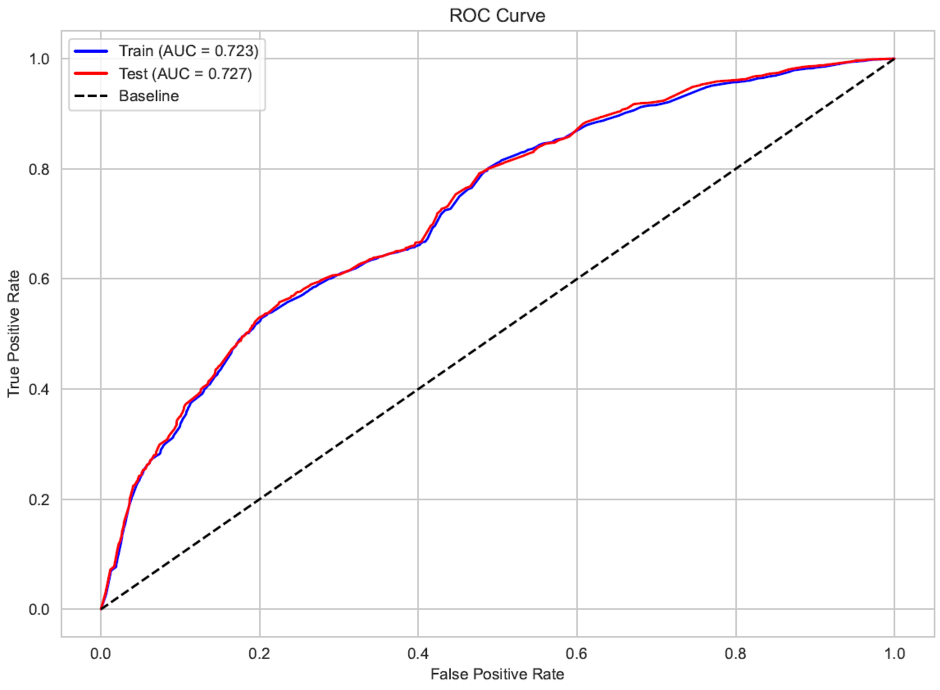

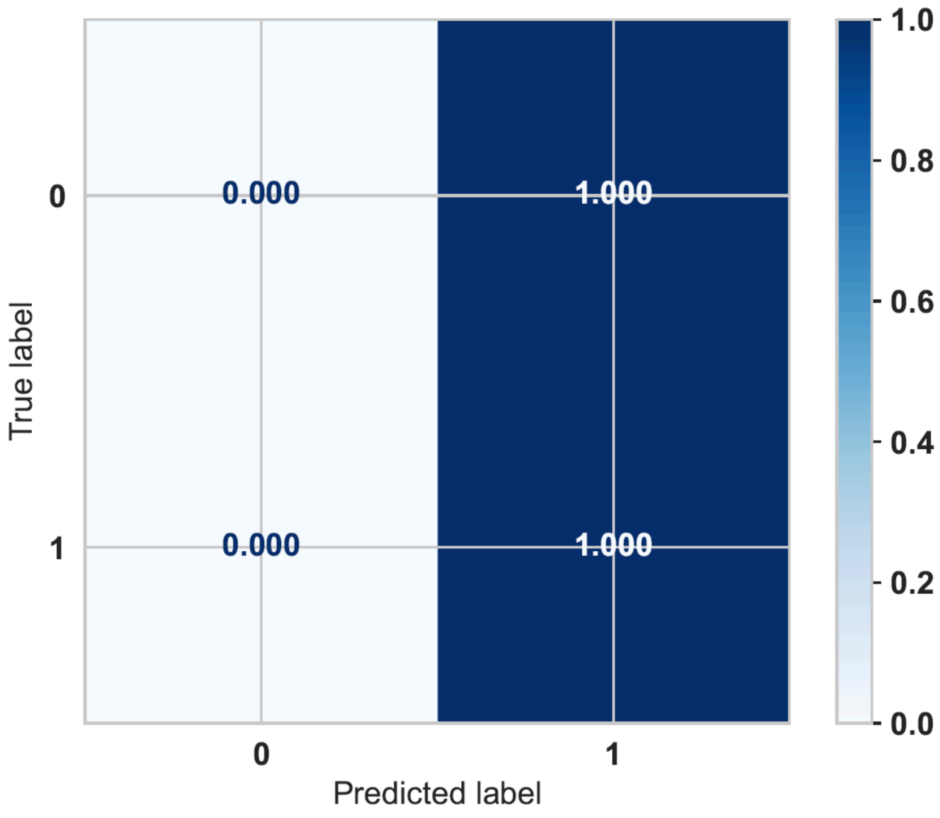

1.3.1.2. XGBoost

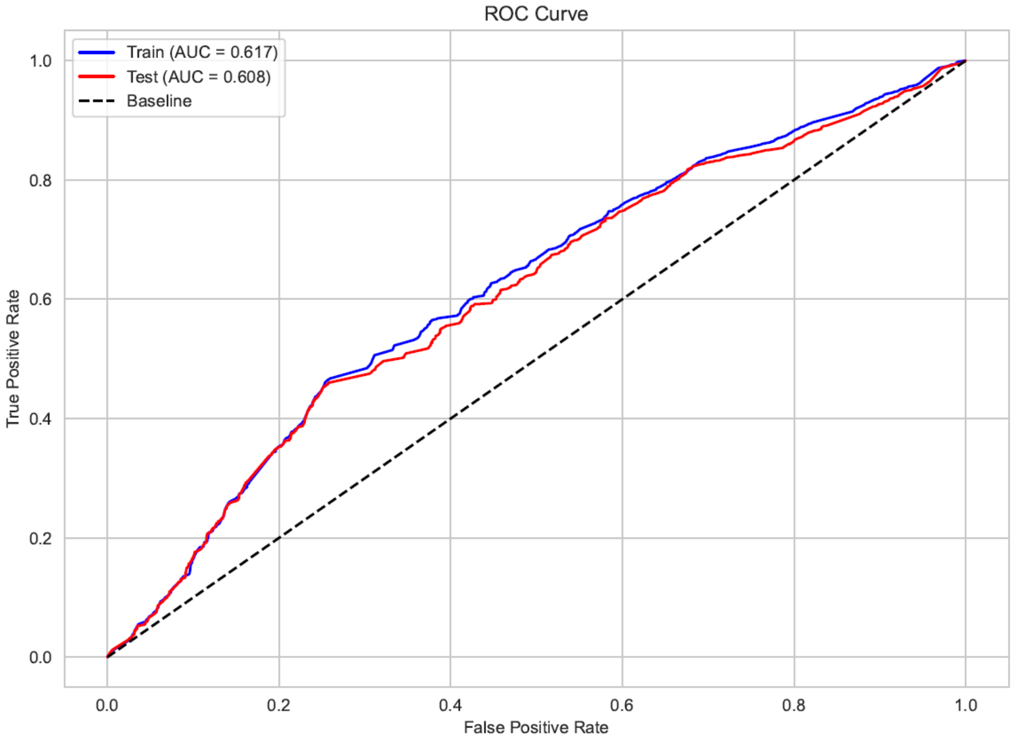

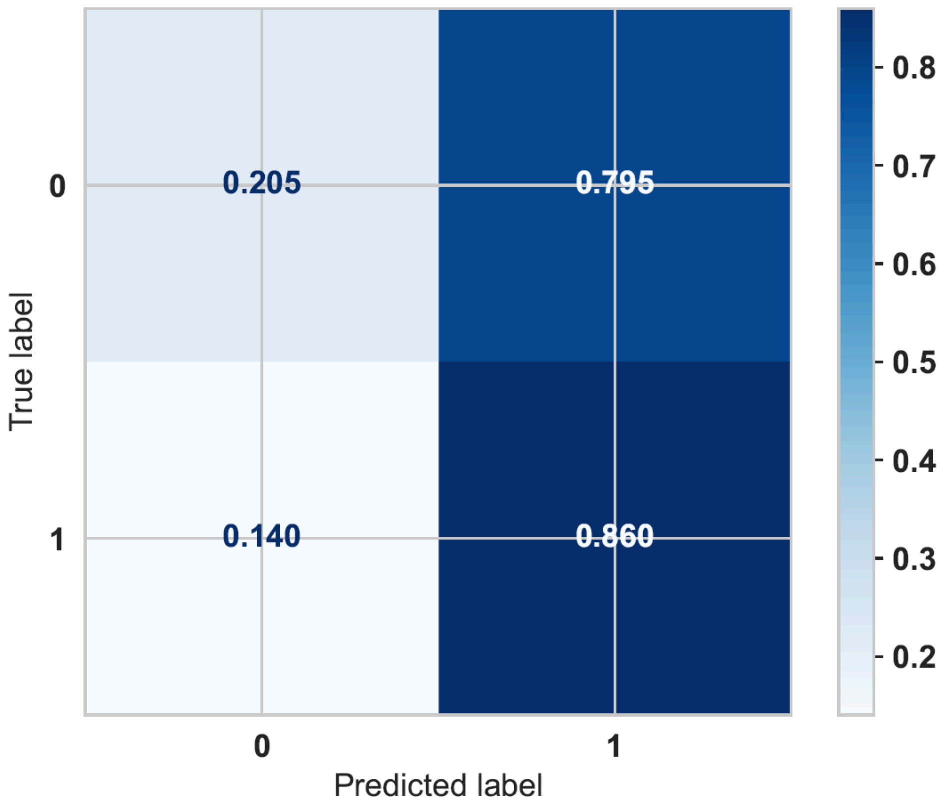

1.3.1.3. CatBoost

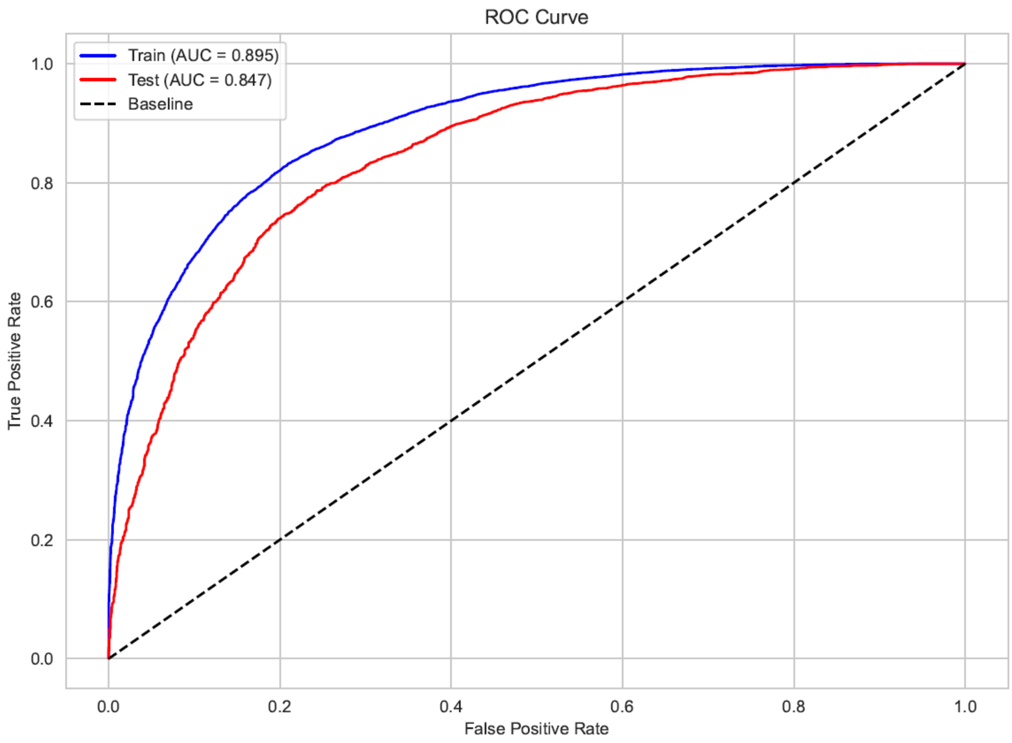

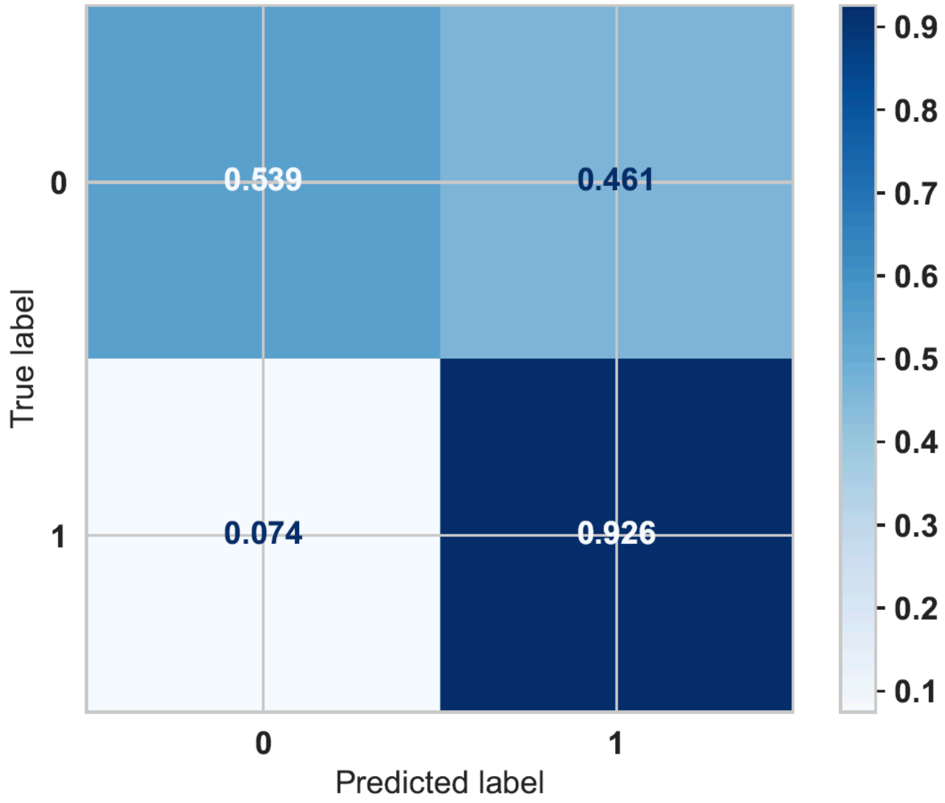

1.3.1.4. DecisionTreeClassifier

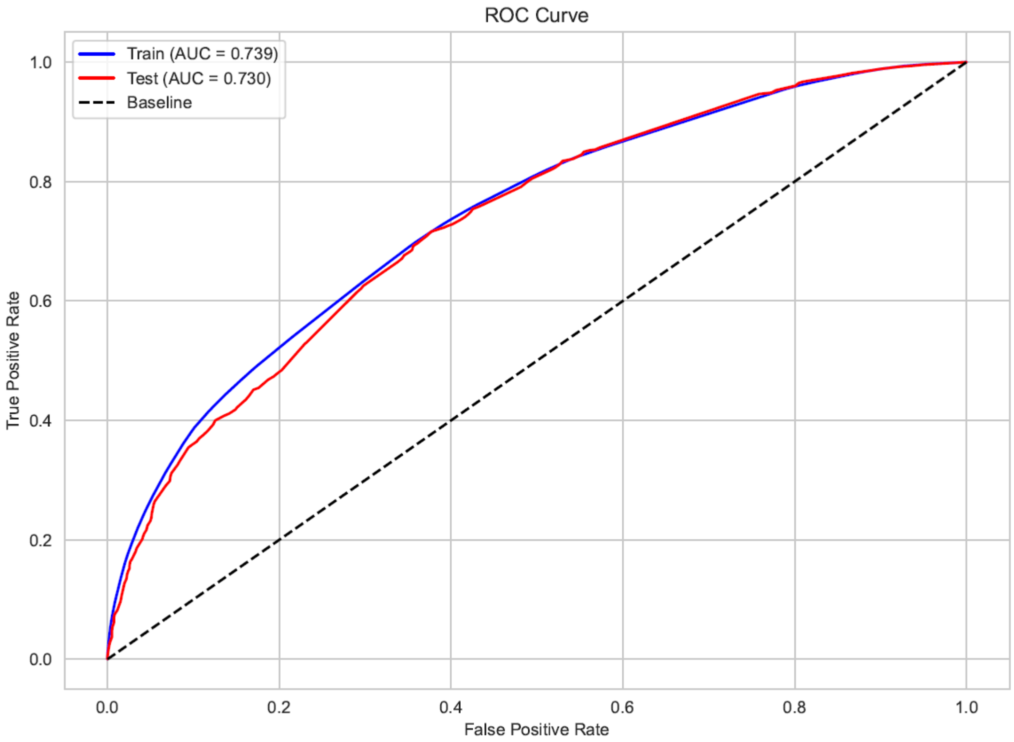

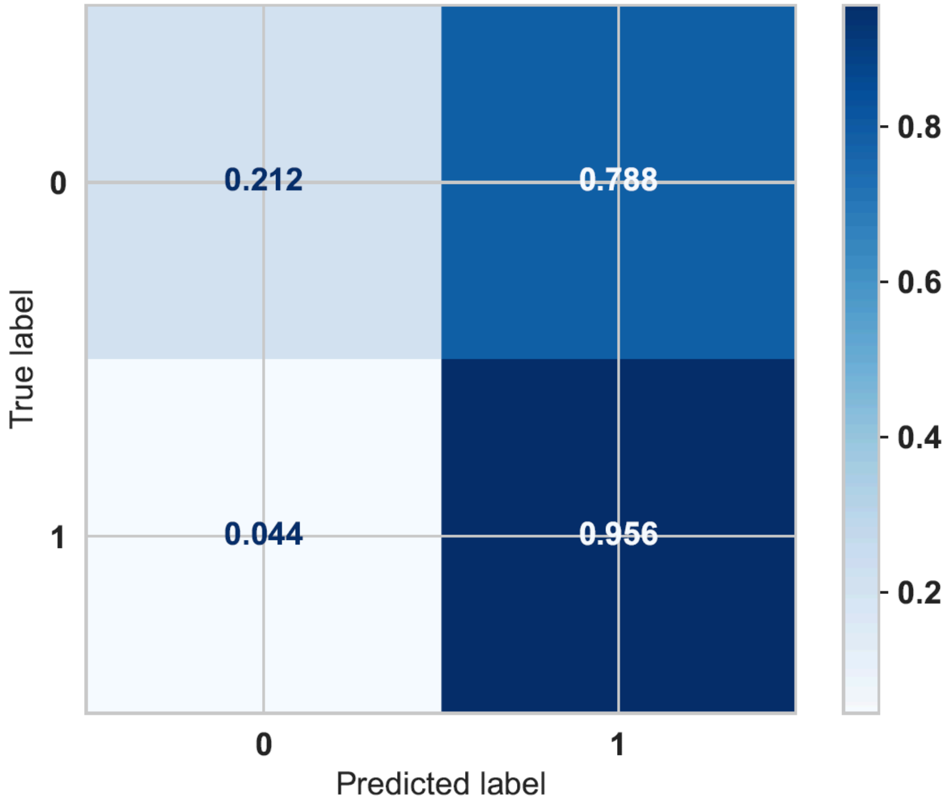

1.3.1.5. ExtraTreesClassifier

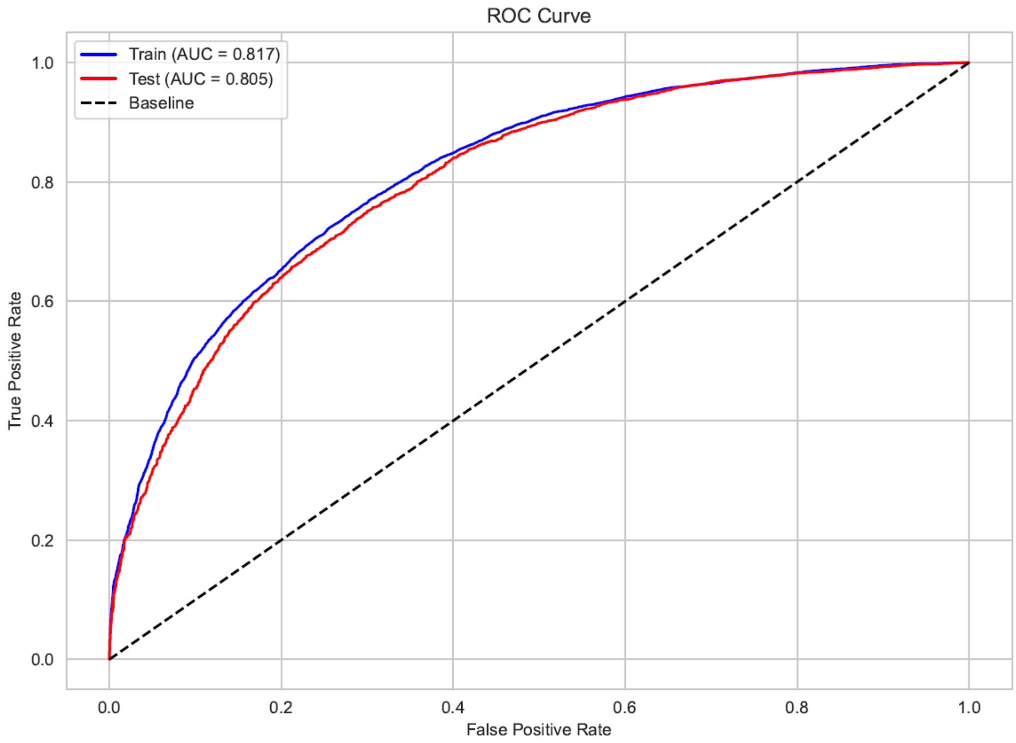

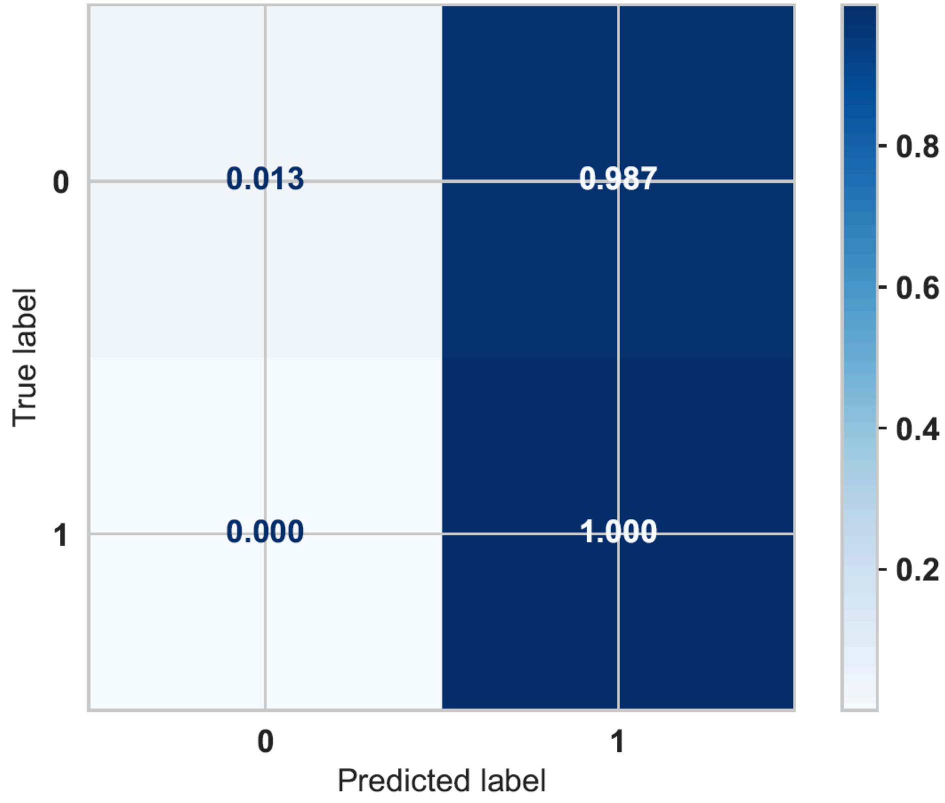

1.3.1.6. GradientBoosting

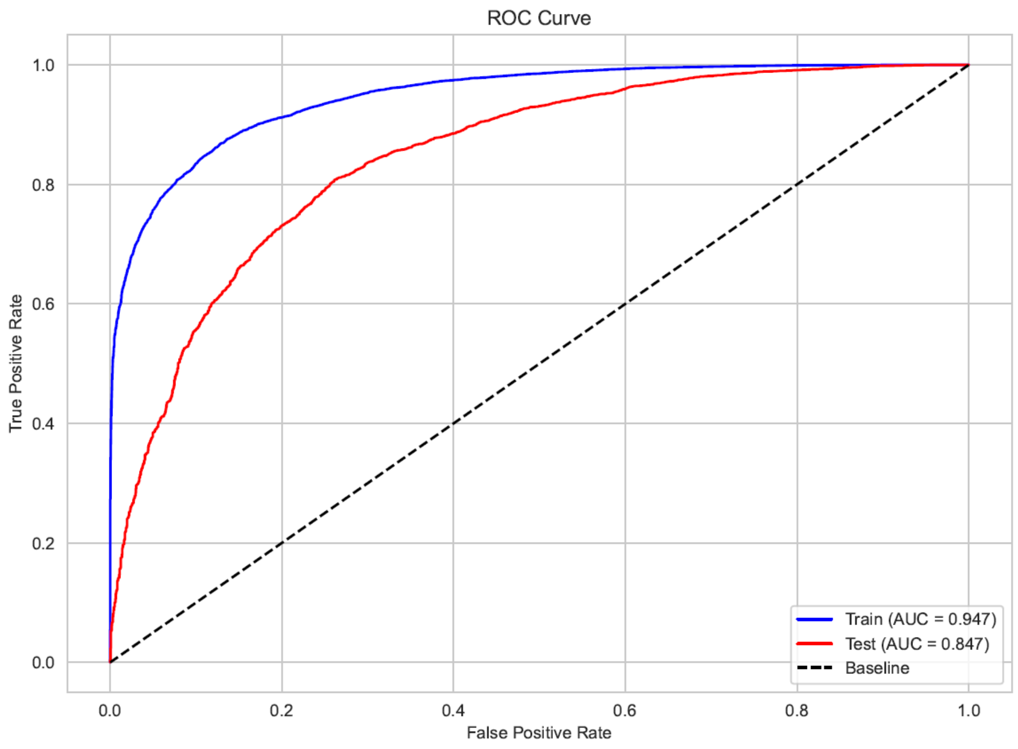
ROC Curve
True Positive Rate
False Positive Rate
0.0
0.2
0.4
0.6
0.8
1.0
Train (AUC = 0.947)
Test (AUC = 0.847)
Baseline

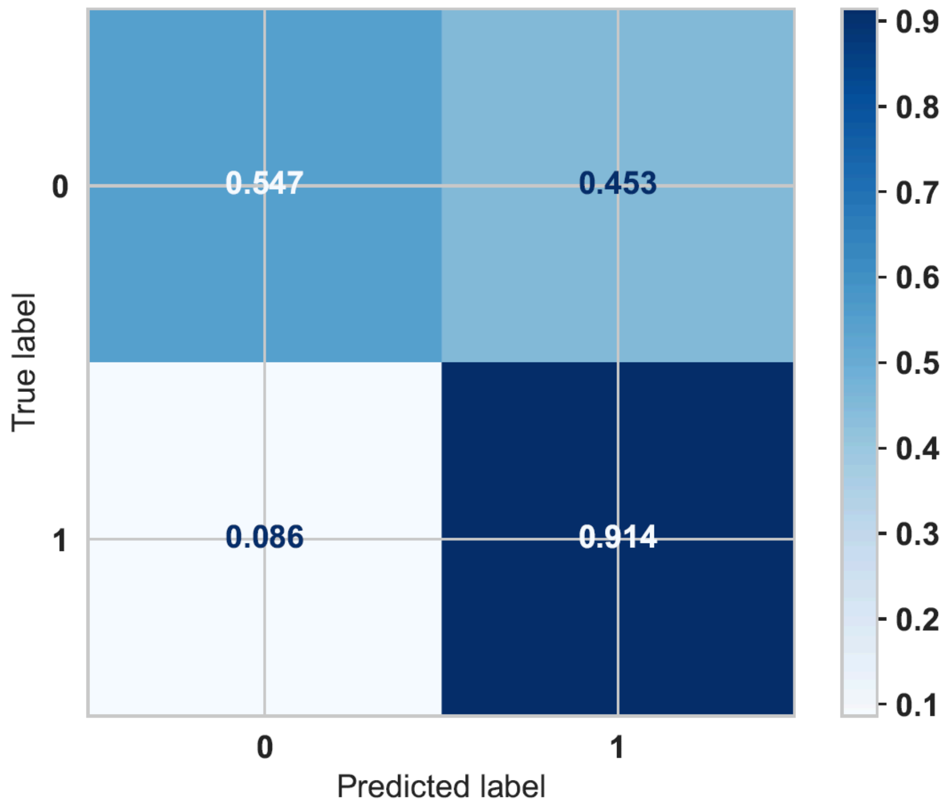
0.547
0.453
0.086
0.914
True label
Predicted label
0
1
0.1
0.2
0.3
0.4
0.5
0.6
0.7
0.8
0.9

1.3.1.7. KNN

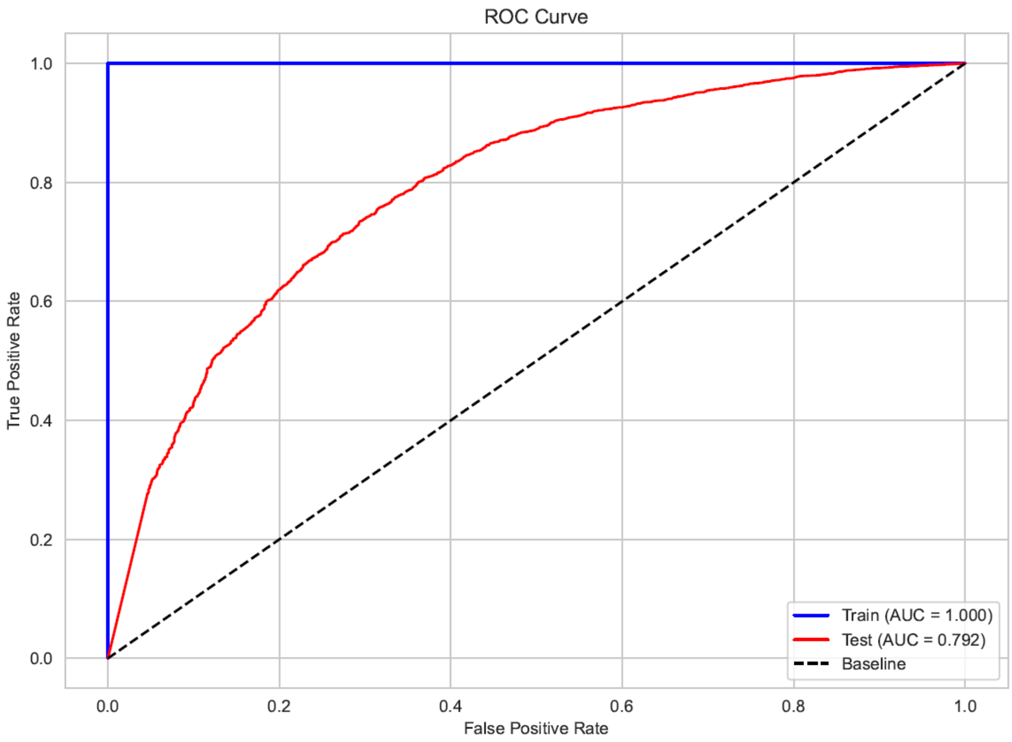

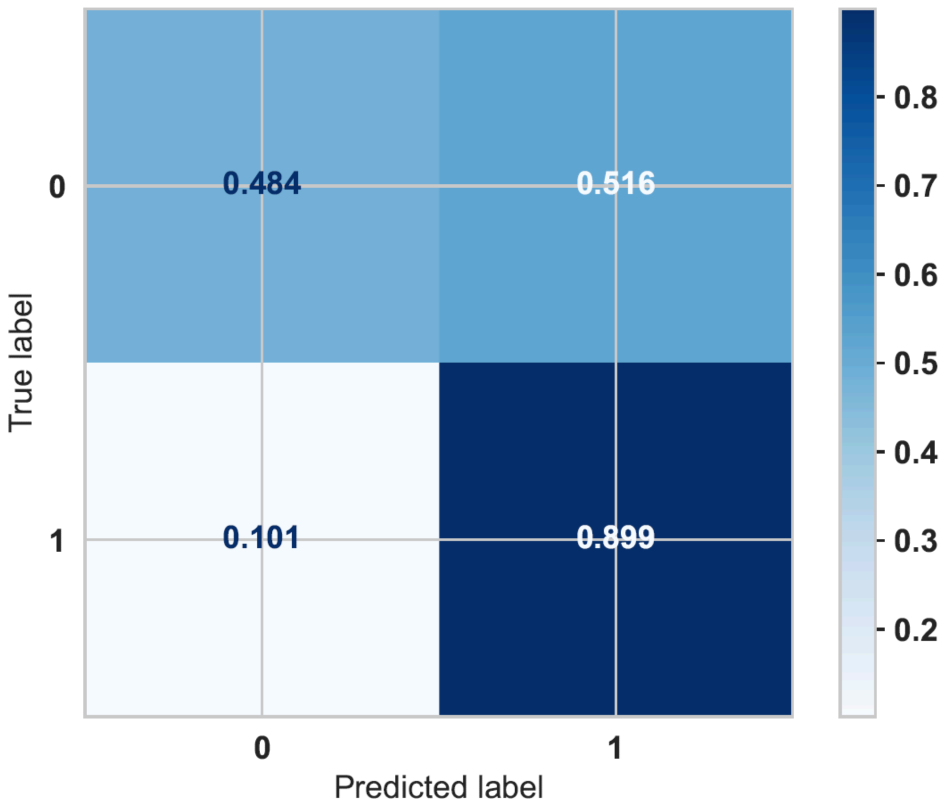

1.3.1.8. lightgbm

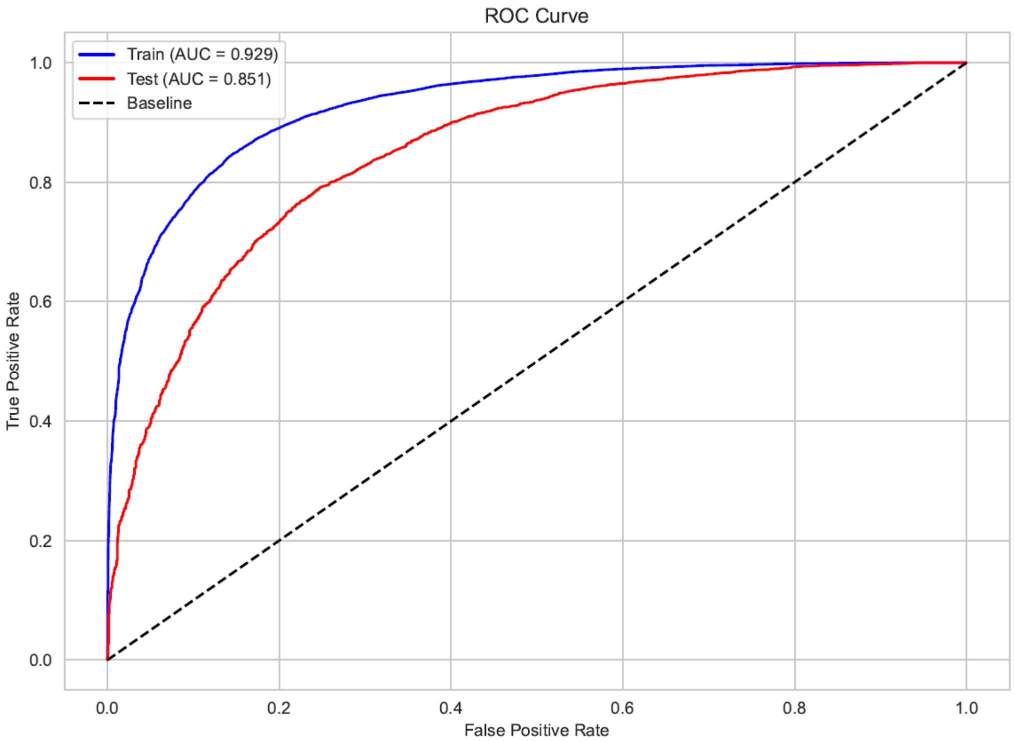

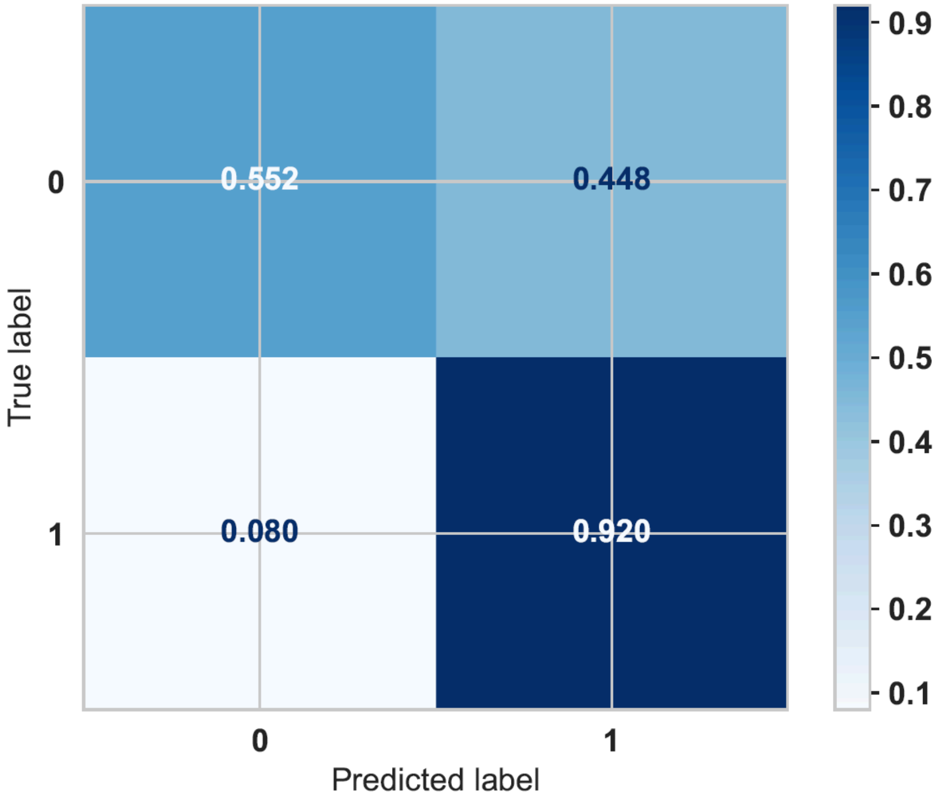

1.3.2. Alive 3 year

1.3.2.1. Random Forest

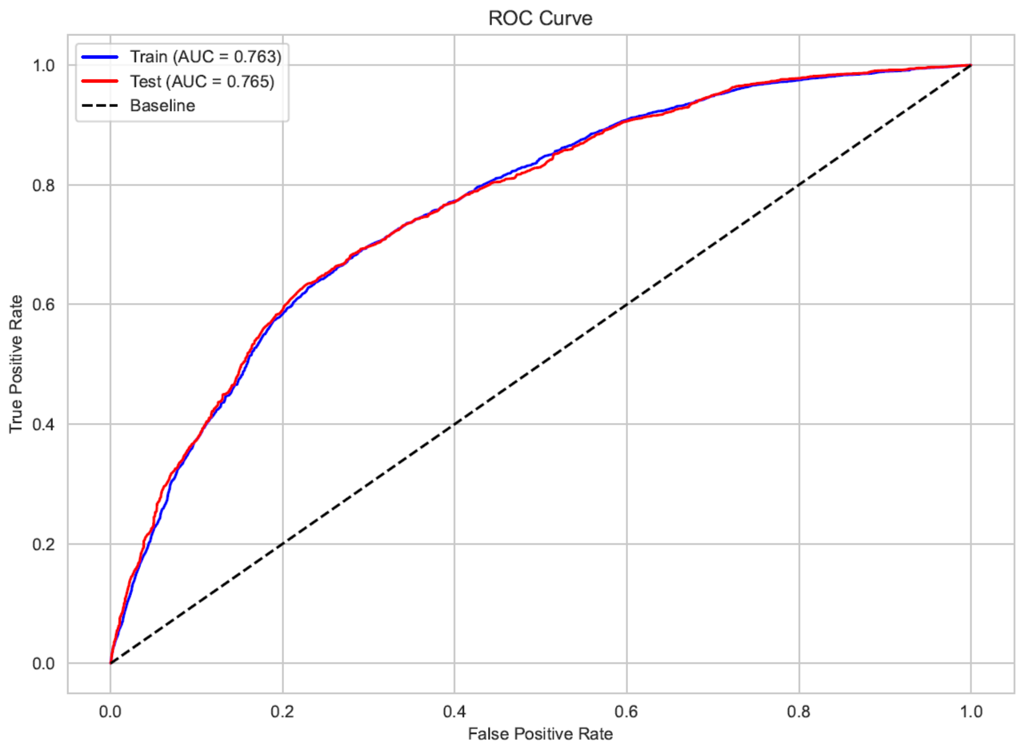

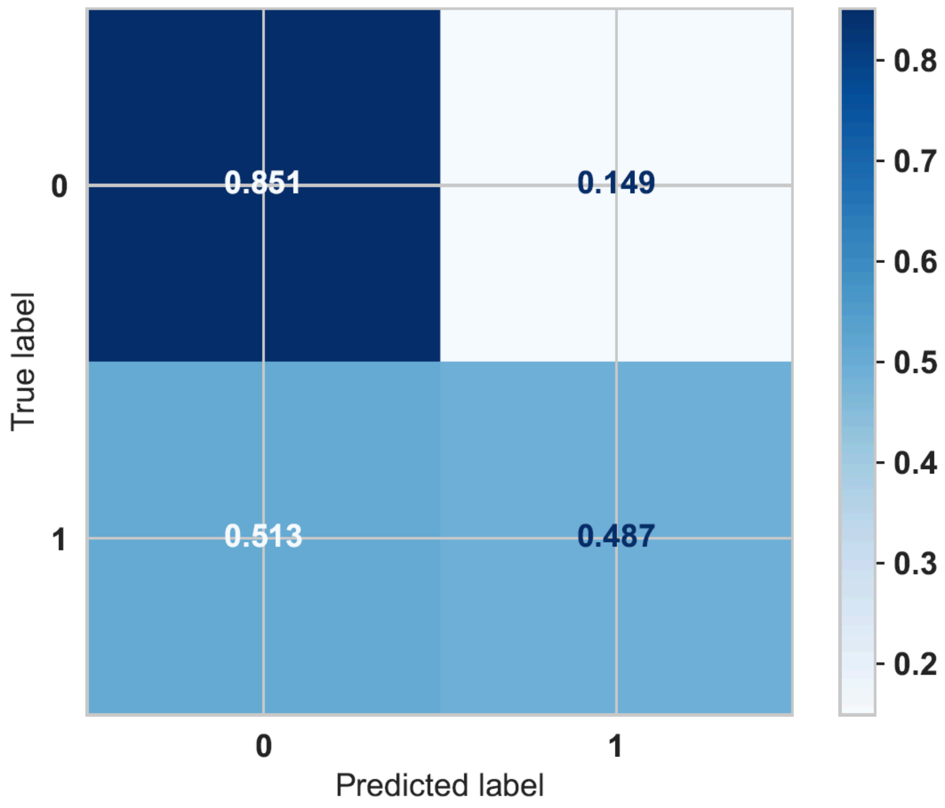

1.3.2.2. XGBoost

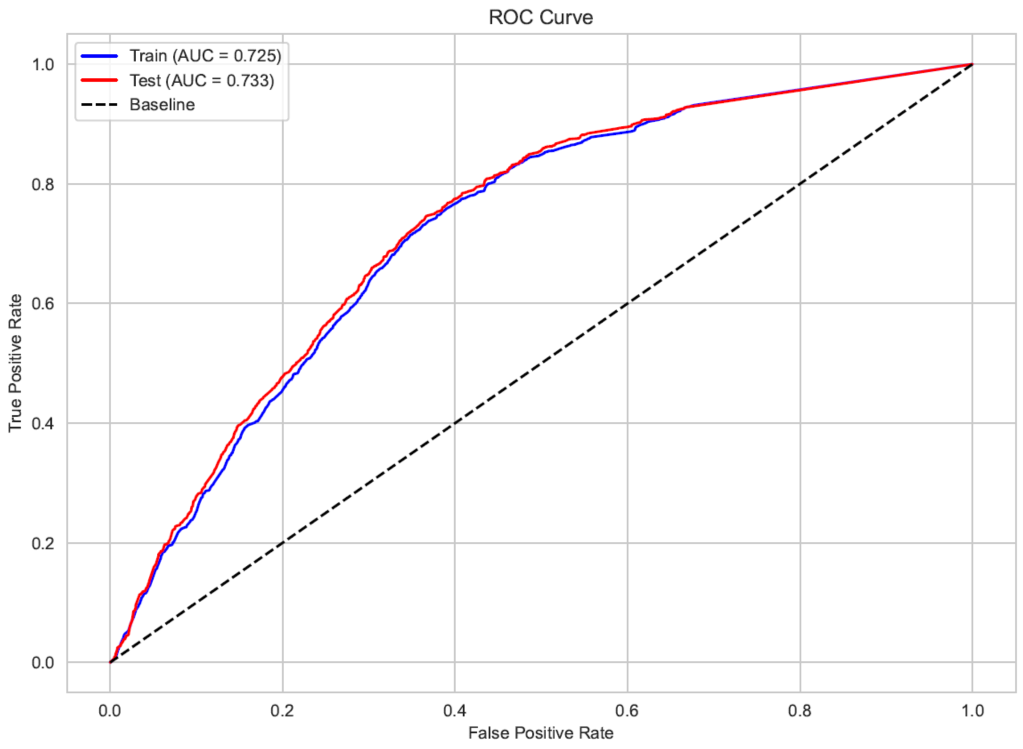

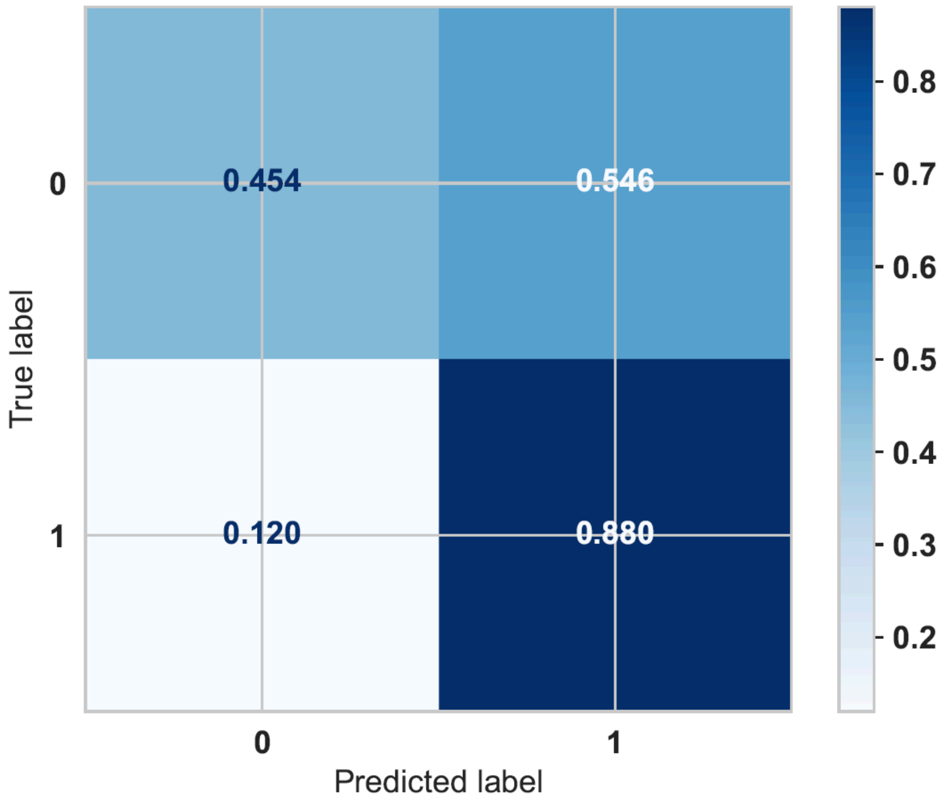

1.3.2.3. CatBoost

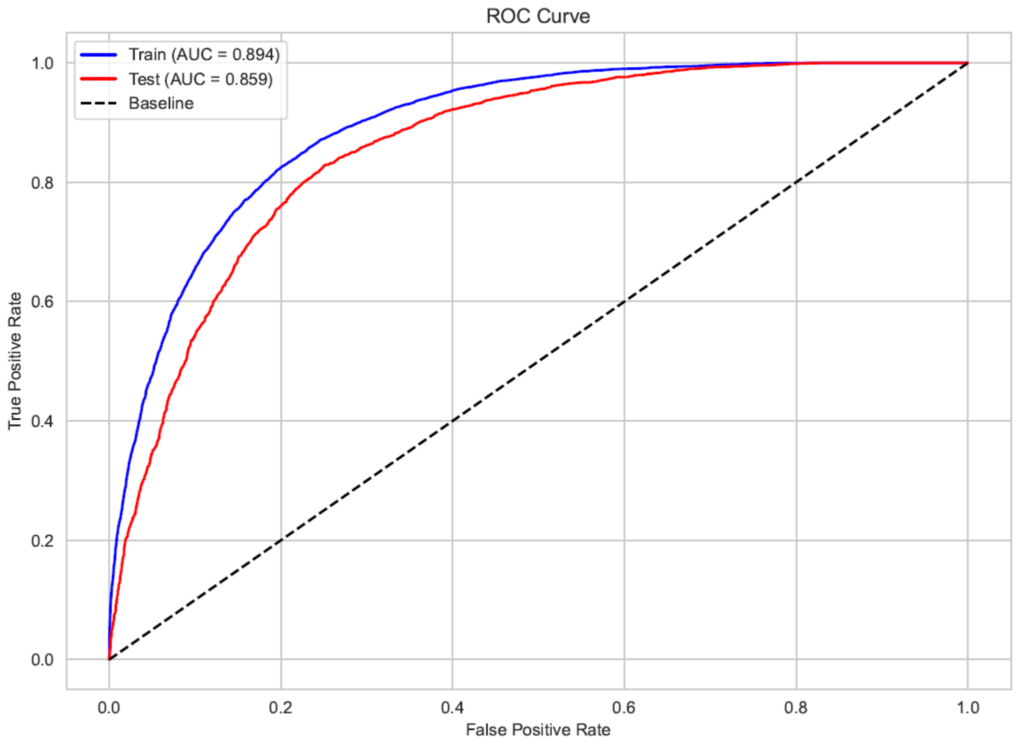

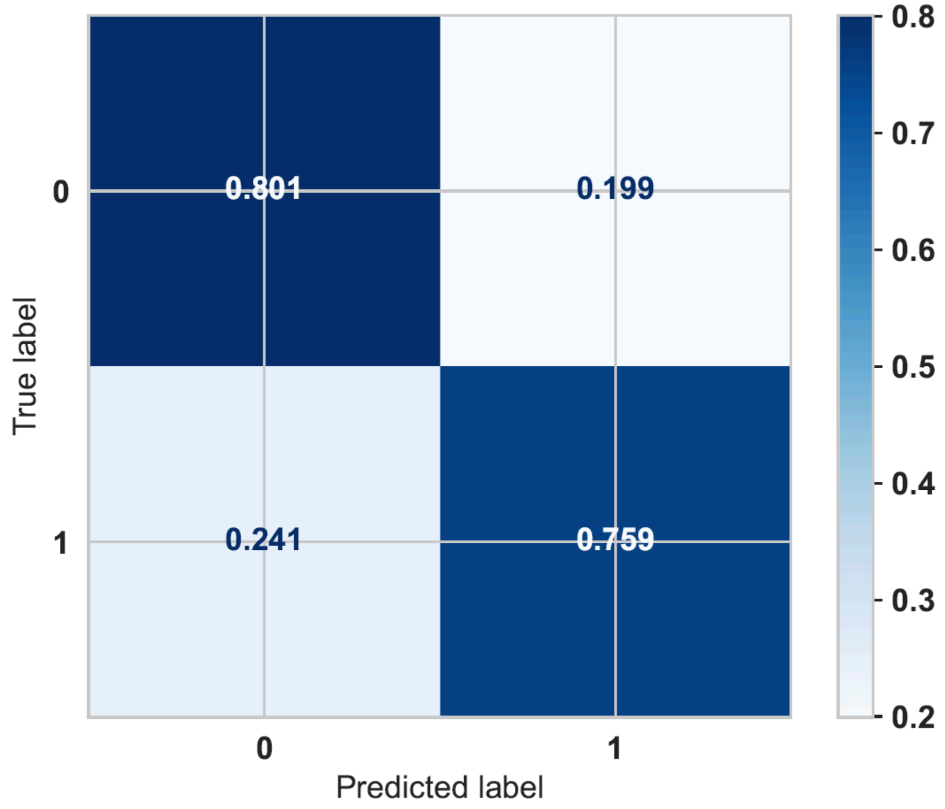

1.3.2.4. DecisionTreeClassifier

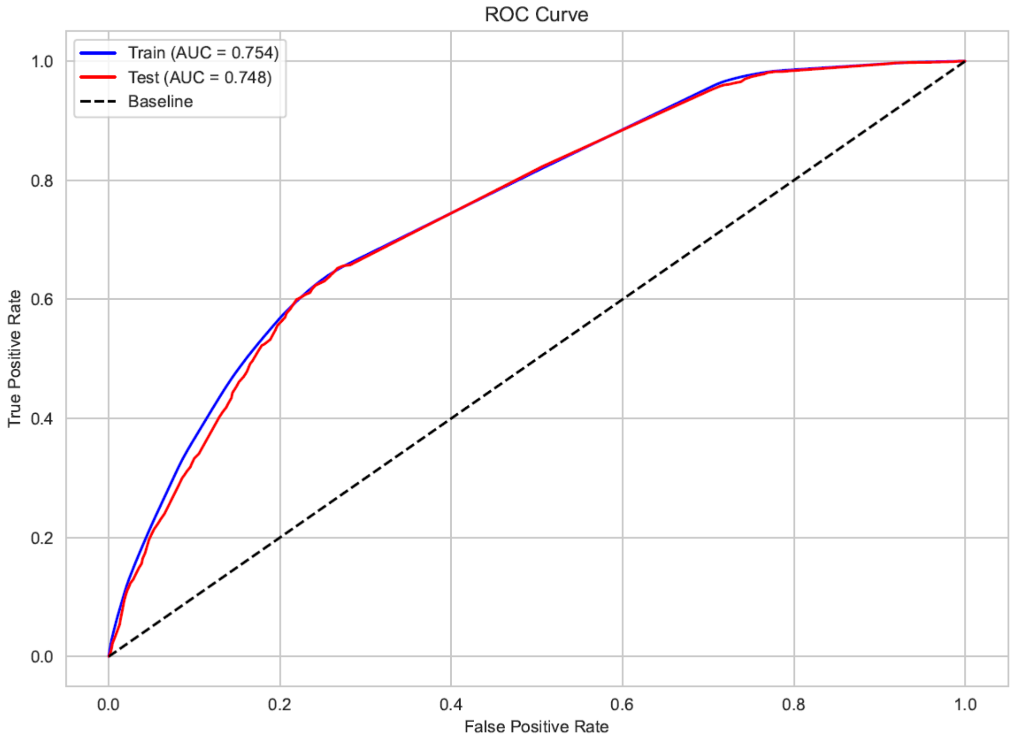

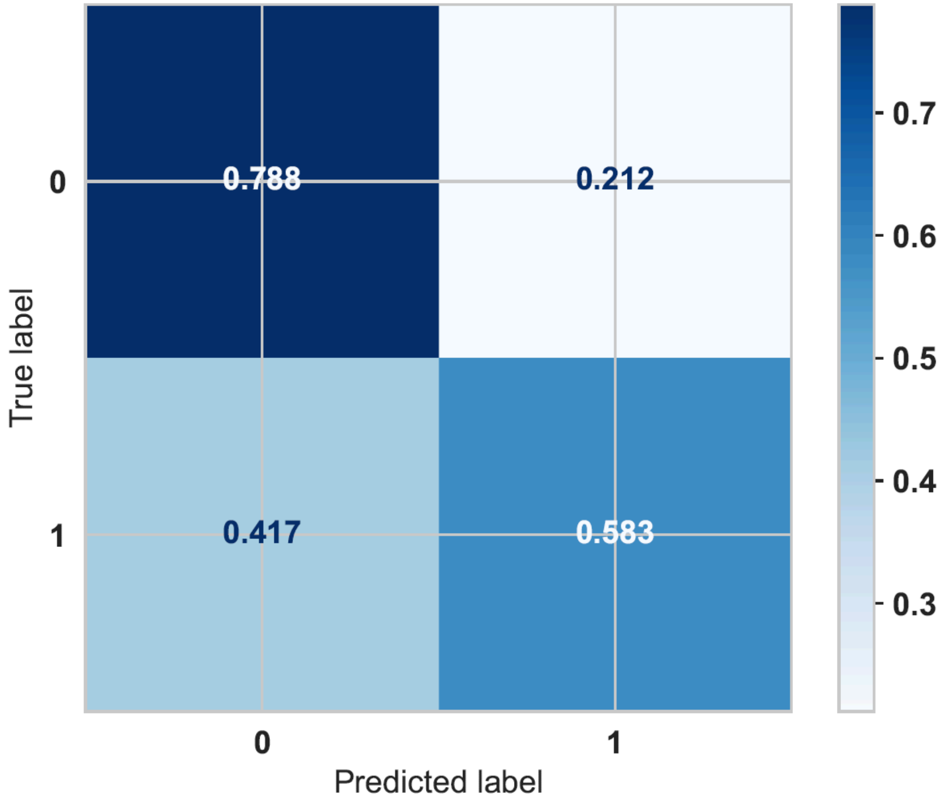

1.3.2.5. ExtraTreesClassifier

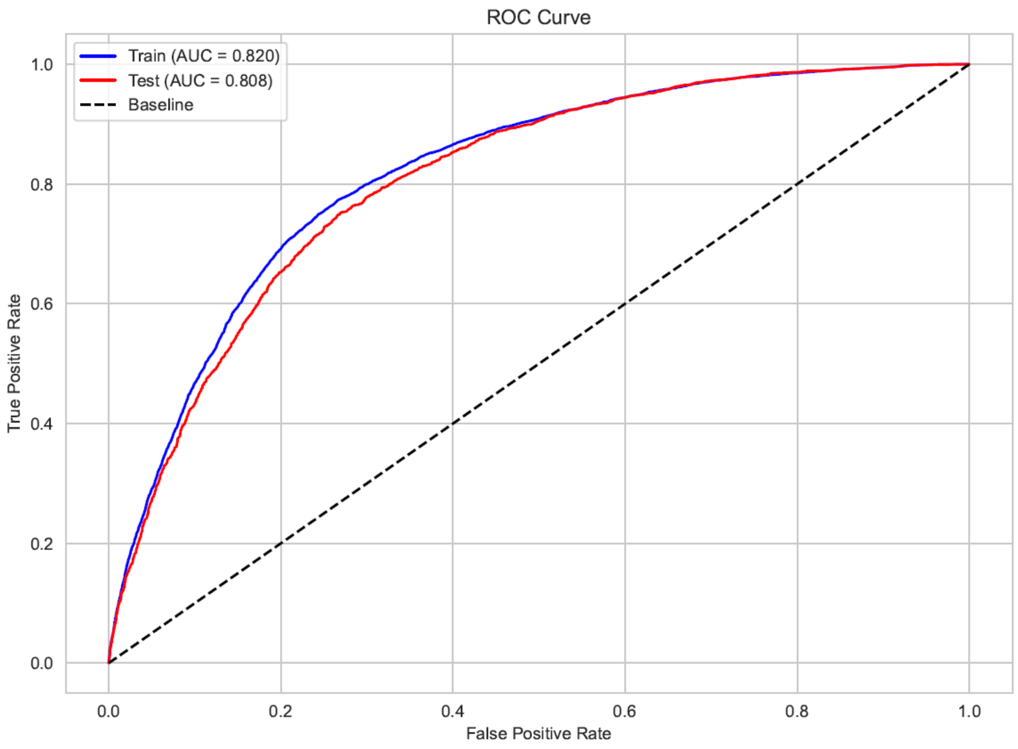

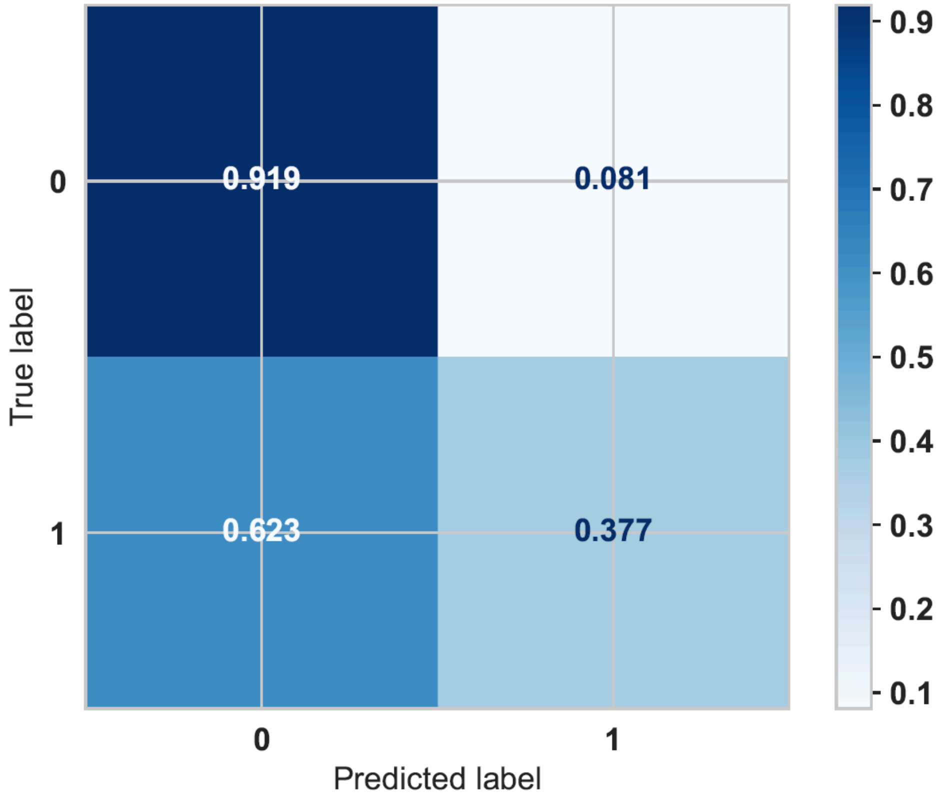

1.3.2.6. GradientBoosting

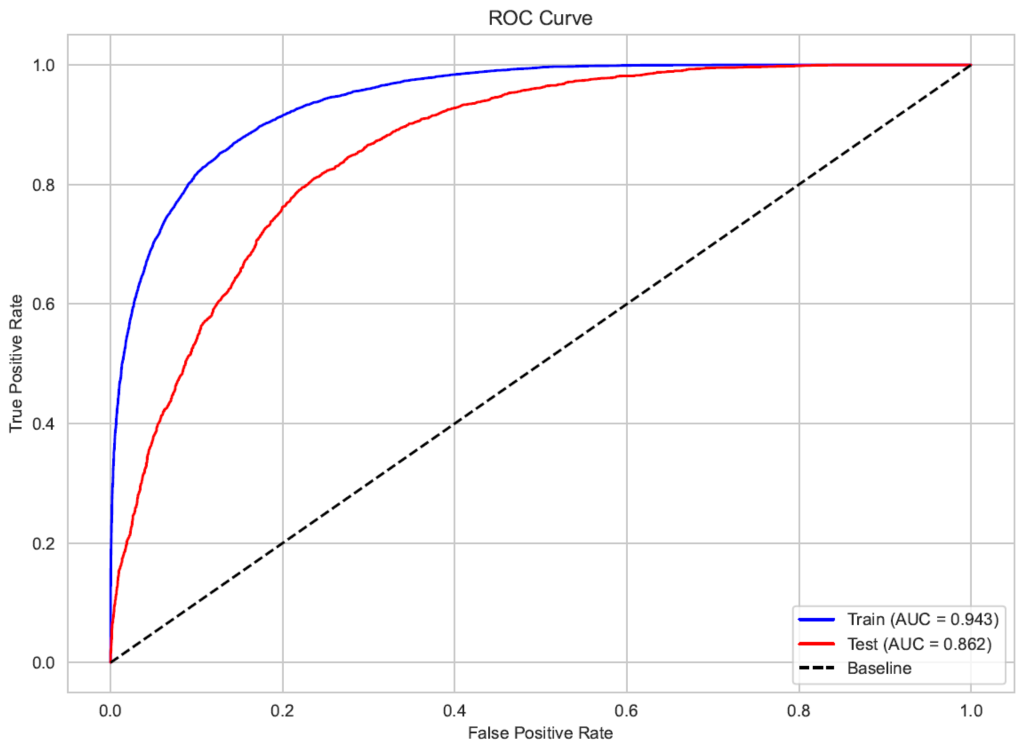

ROC Curve
1.0
0.8
0.6
0.4
0.2
0.0
True Positive Rate
0.0
0.2
0.4
0.6
0.8
1.0
False Positive Rate
Train (AUC = 0.943)
Test (AUC = 0.862)
Baseline

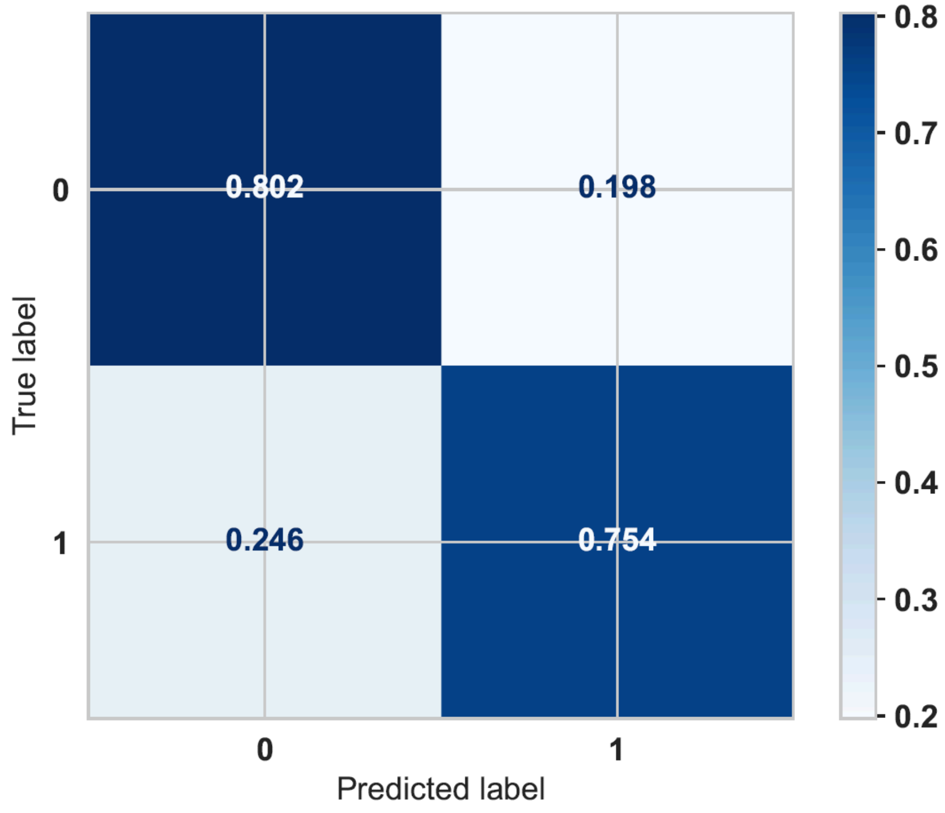

0.802
0.198
0.246
0.754
0
1
True label
0
1
Predicted label
0.8
0.7
0.6
0.5
0.4
0.3
0.2

1.3.2.7. KNN

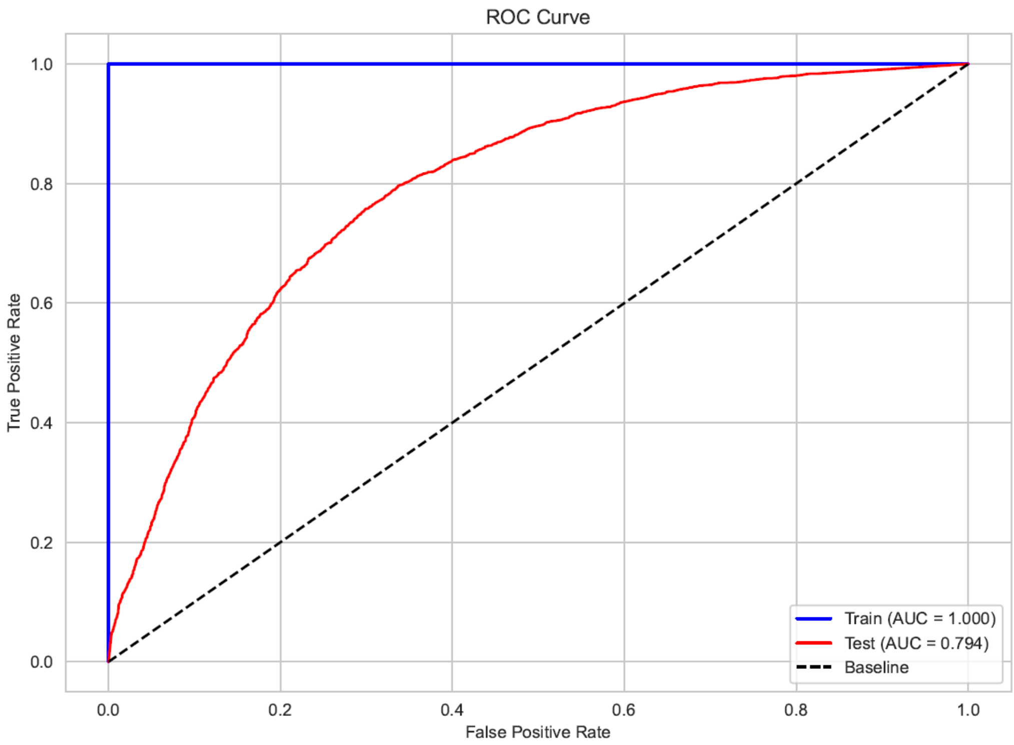

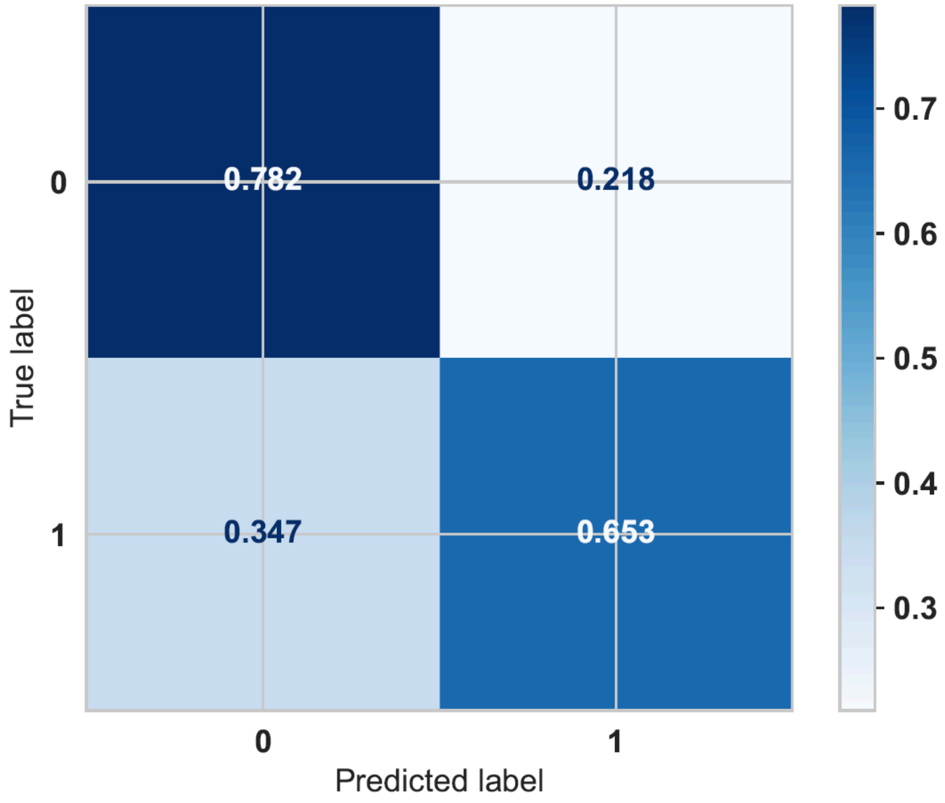

1.3.2.8. lightgbm

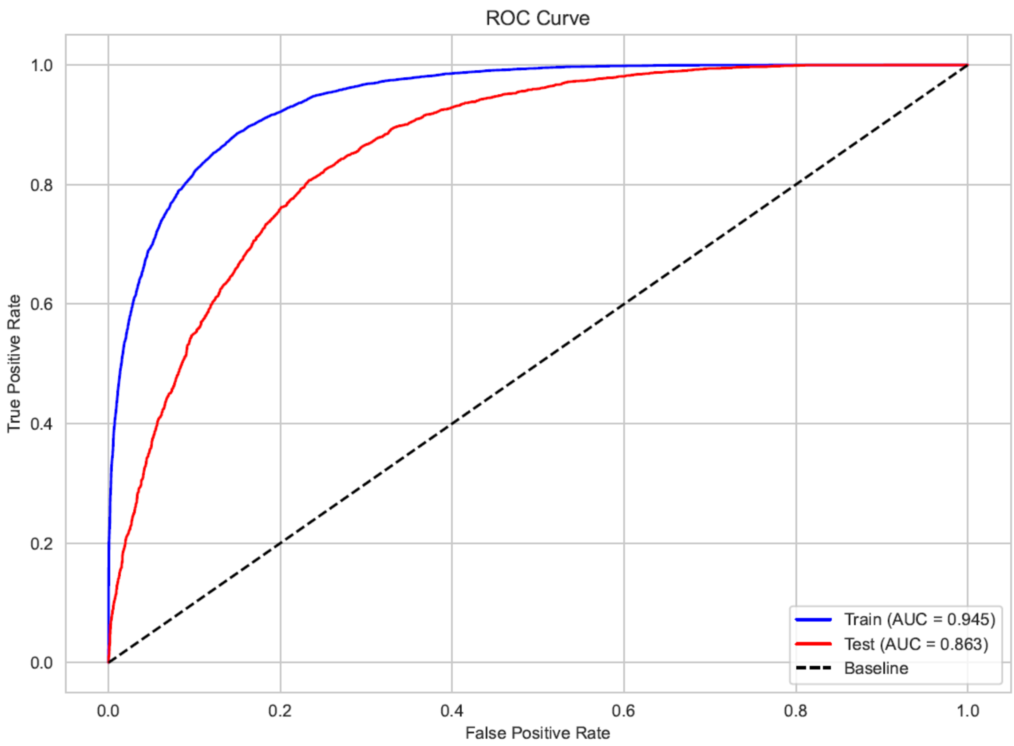

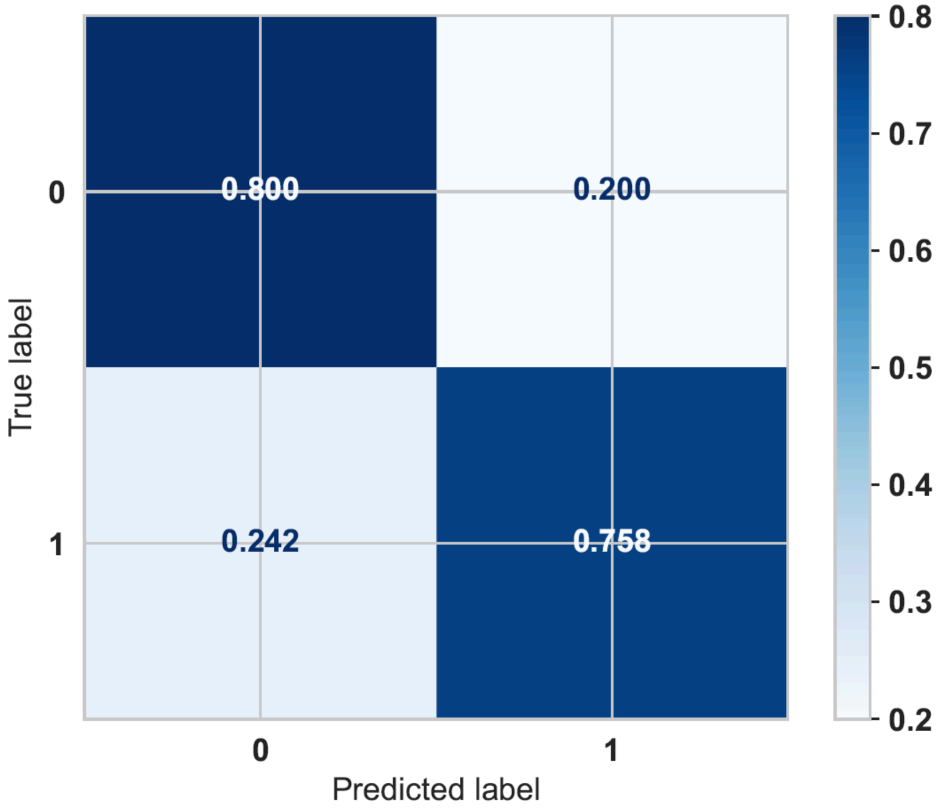

1.3.3. Alive 5 year

1.3.3.1. Random Forest

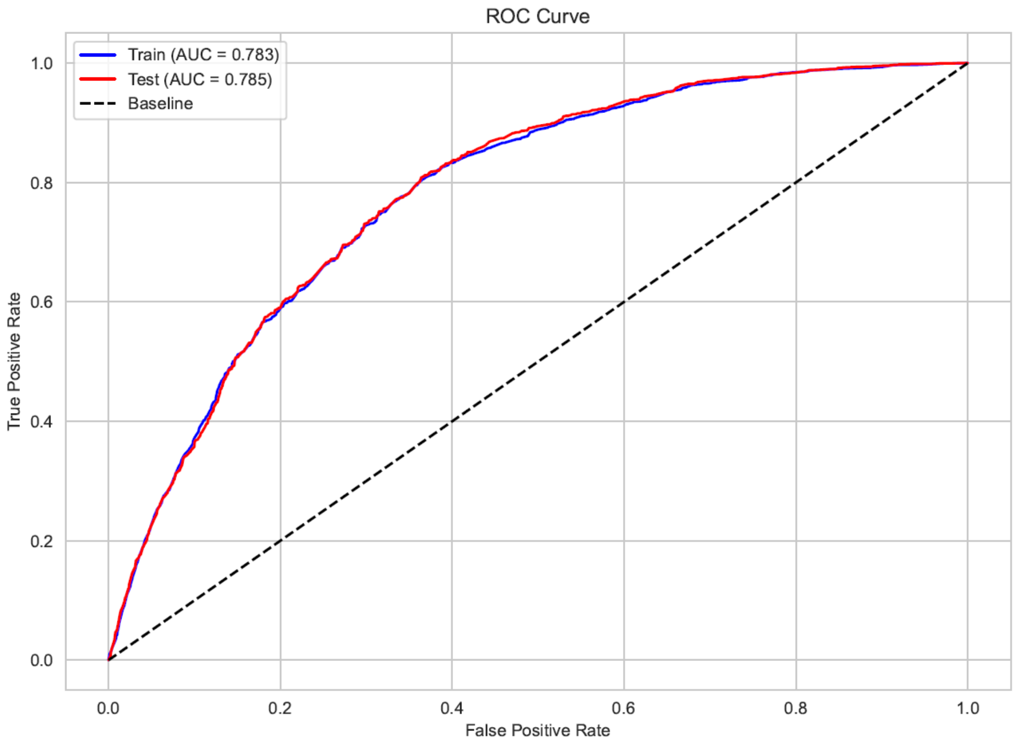

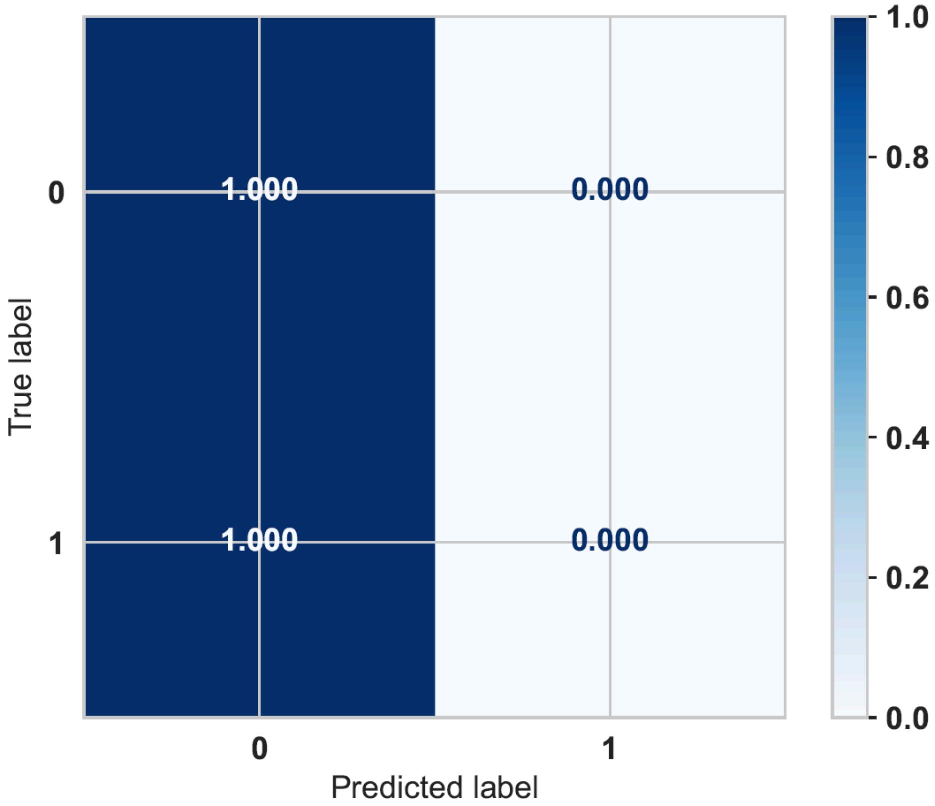

1.3.3.2. XGBoost

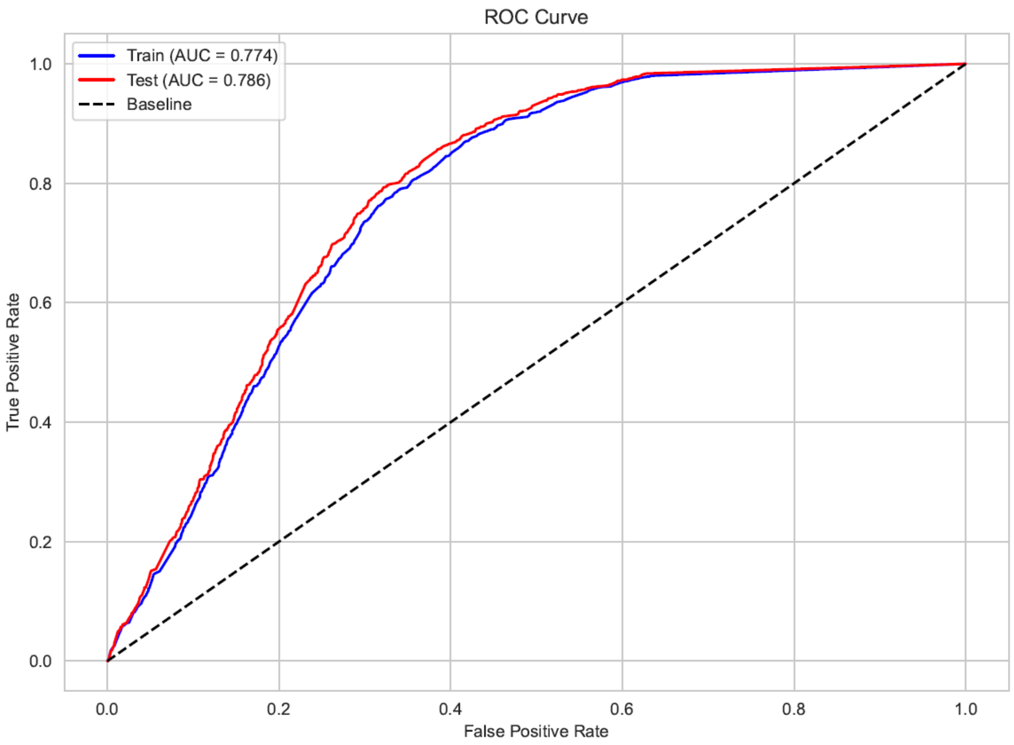

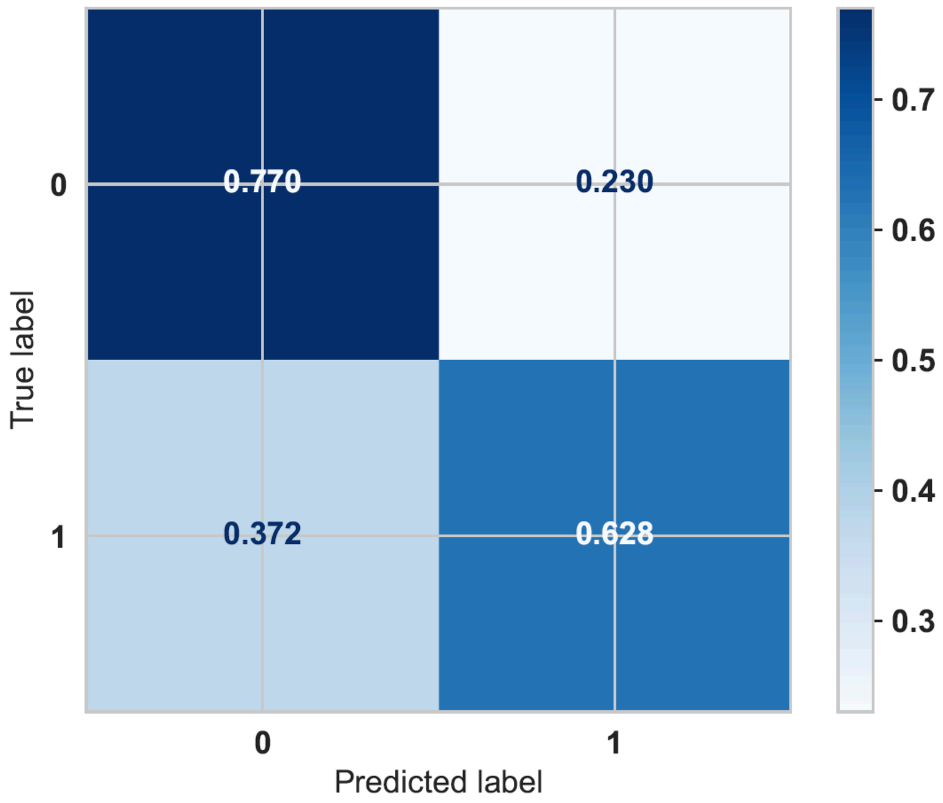

1.3.3.3. CatBoost

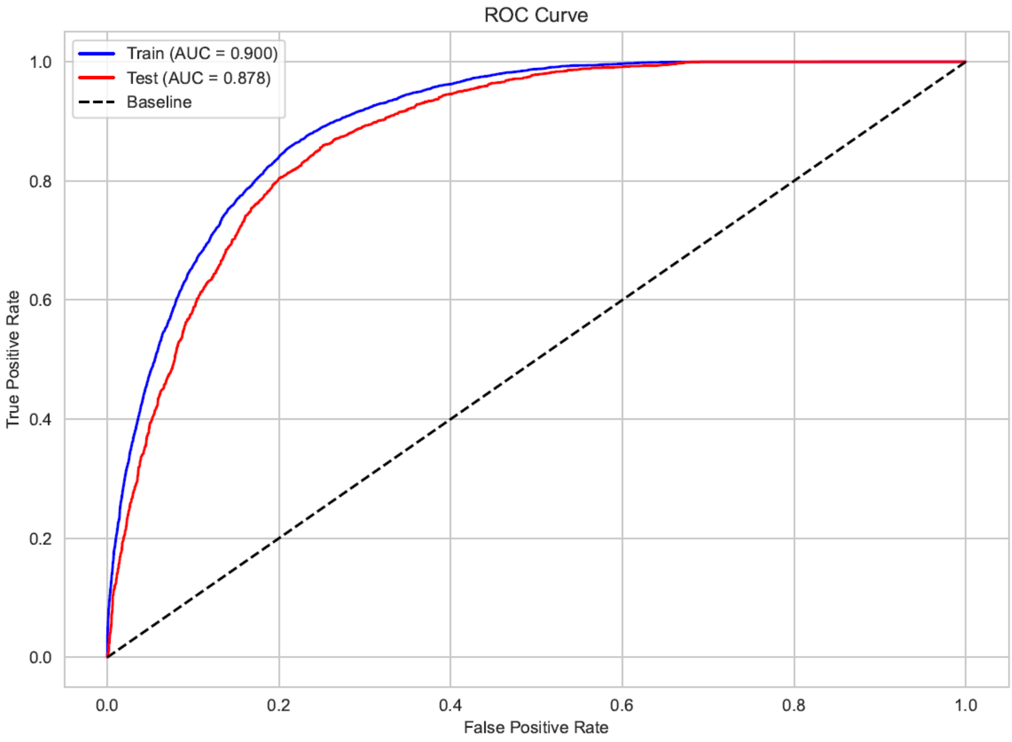

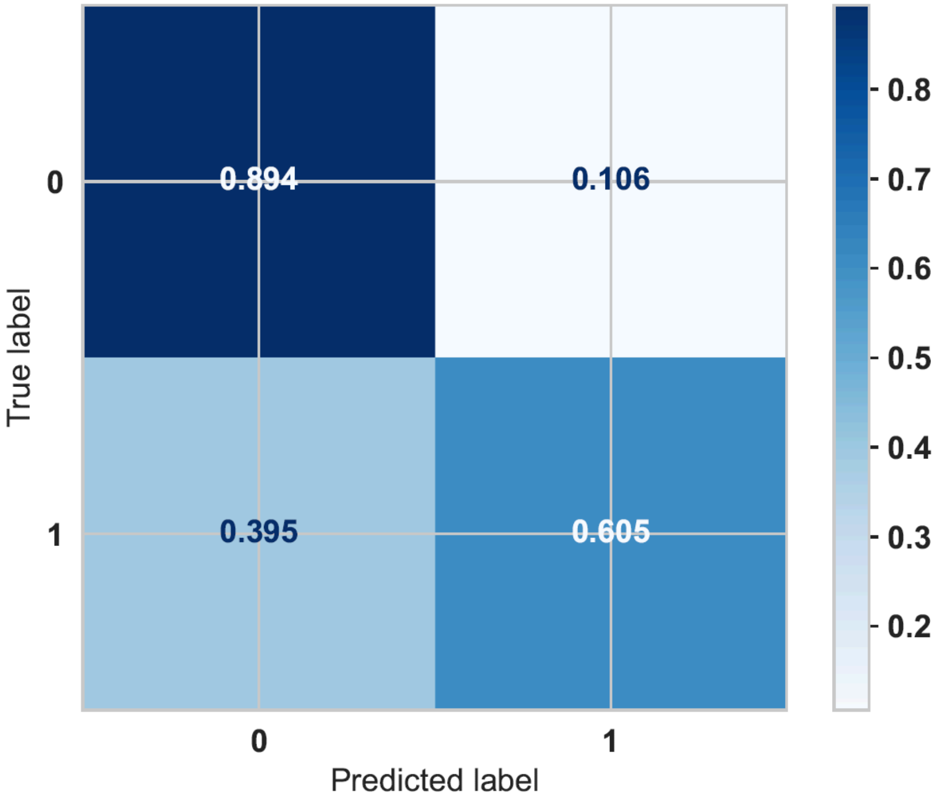

1.3.3.4. DecisionTreeClassifier

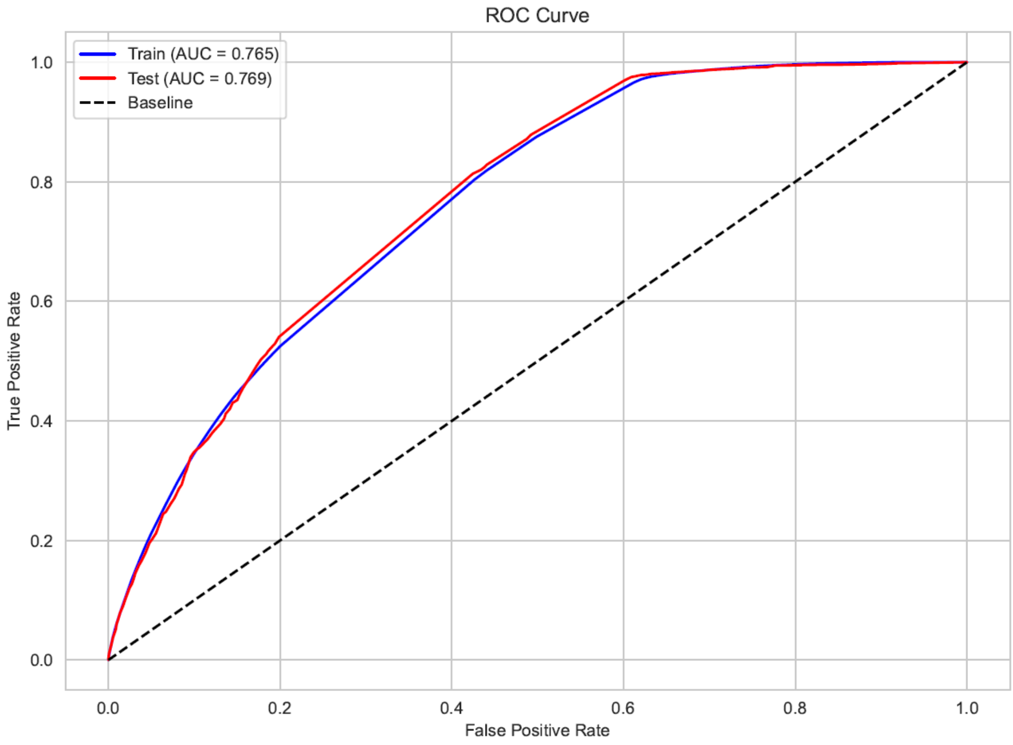

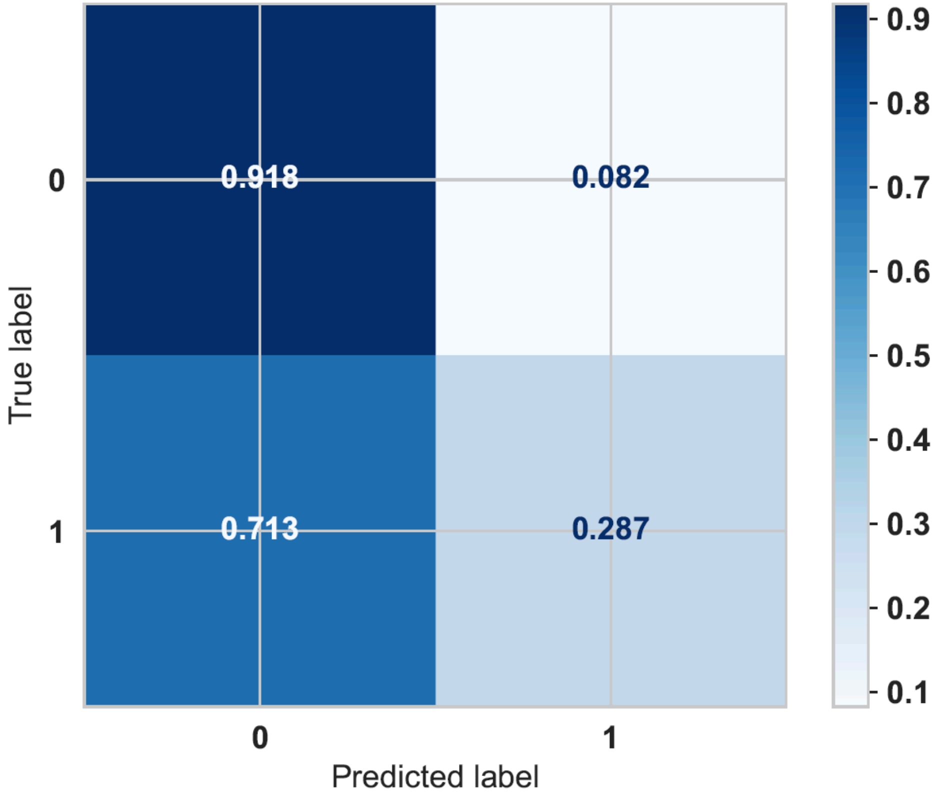

1.3.3.5. ExtraTreesClassifier

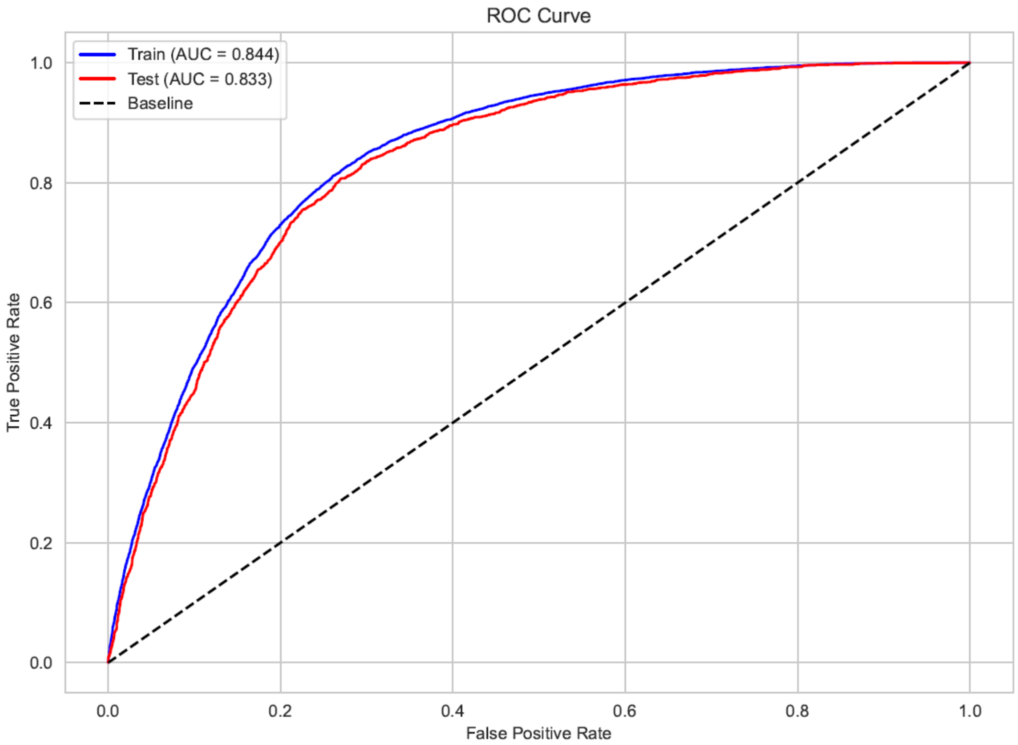
ROC Curve
Train (AUC = 0.844)
Test (AUC = 0.833)
Baseline
True Positive Rate
False Positive Rate
0.0
0.2
0.4
0.6
0.8
1.0

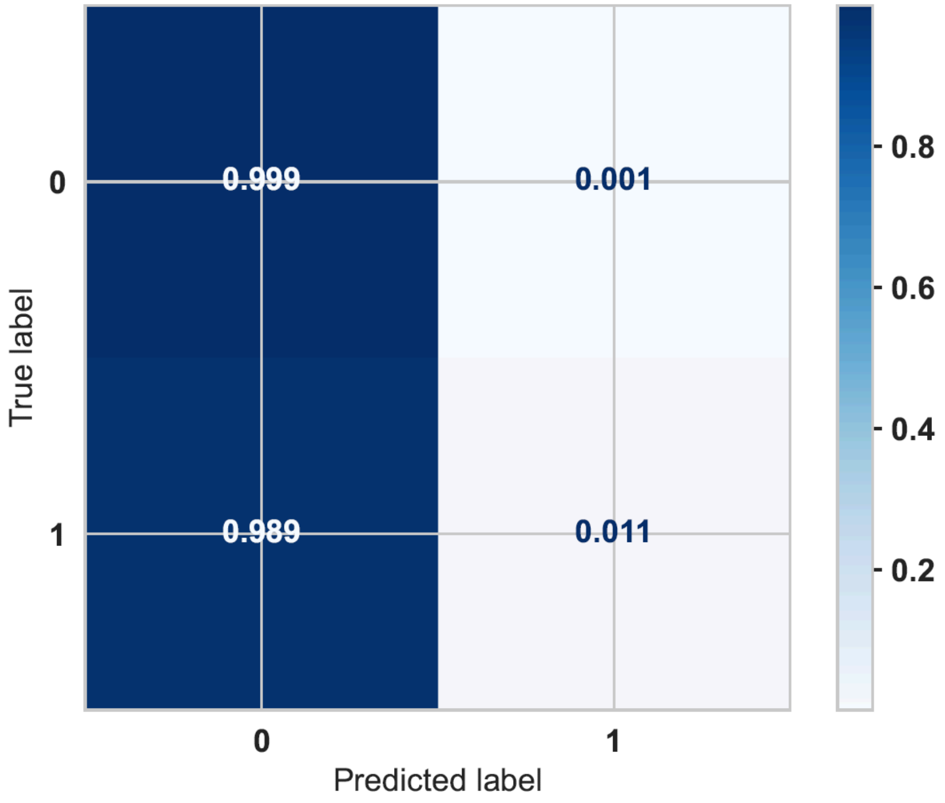
0.999
0.001
0.989
0.011
True label
Predicted label
0
1
0.8
0.6
0.4
0.2

1.3.3.6. GradientBoosting

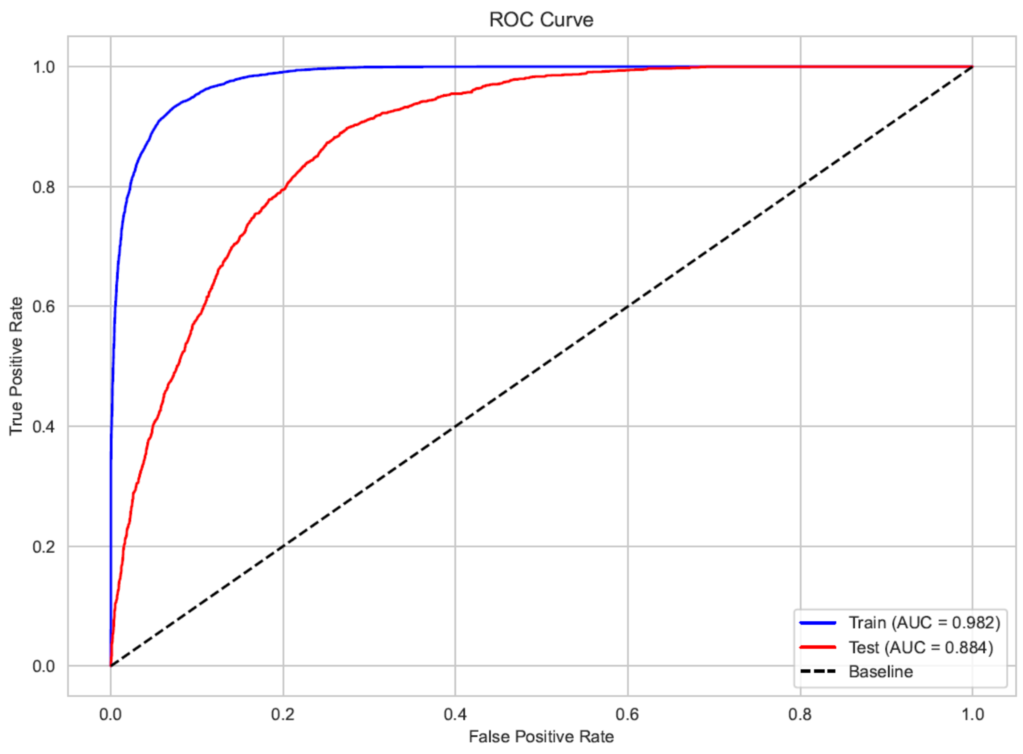

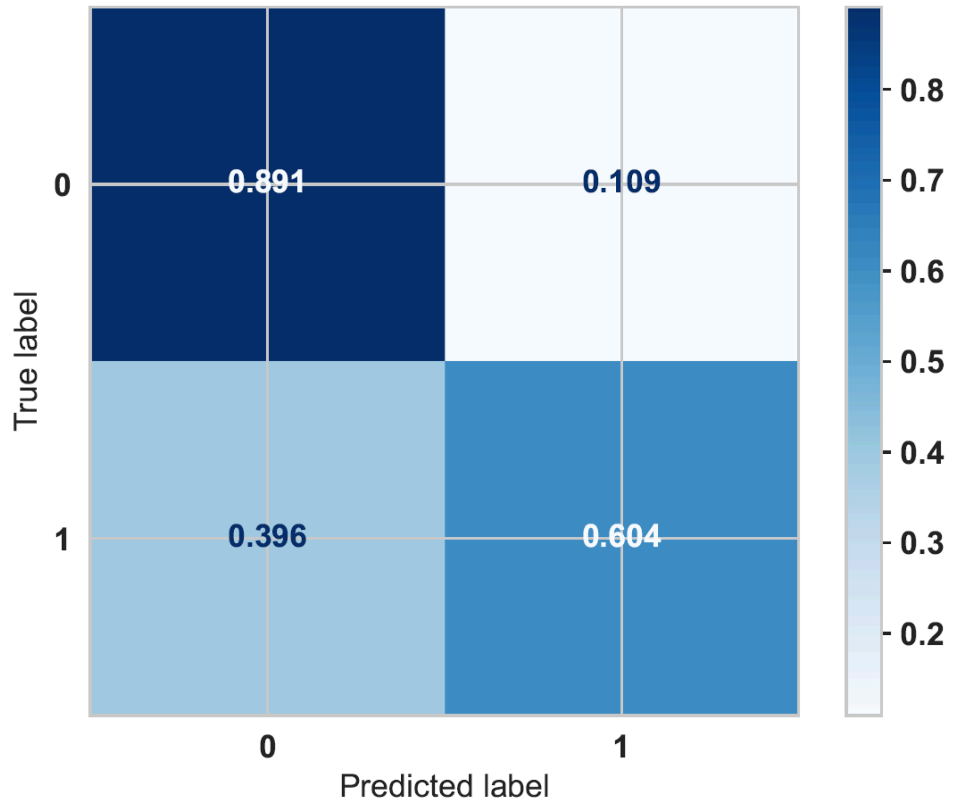

1.3.3.7. KNN

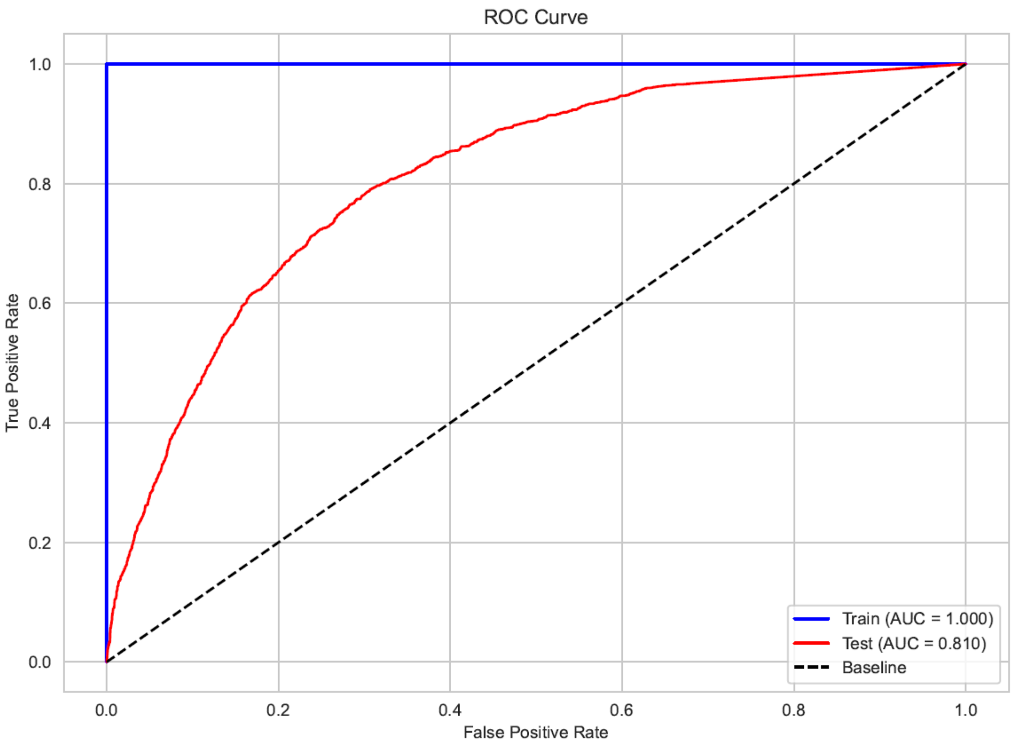

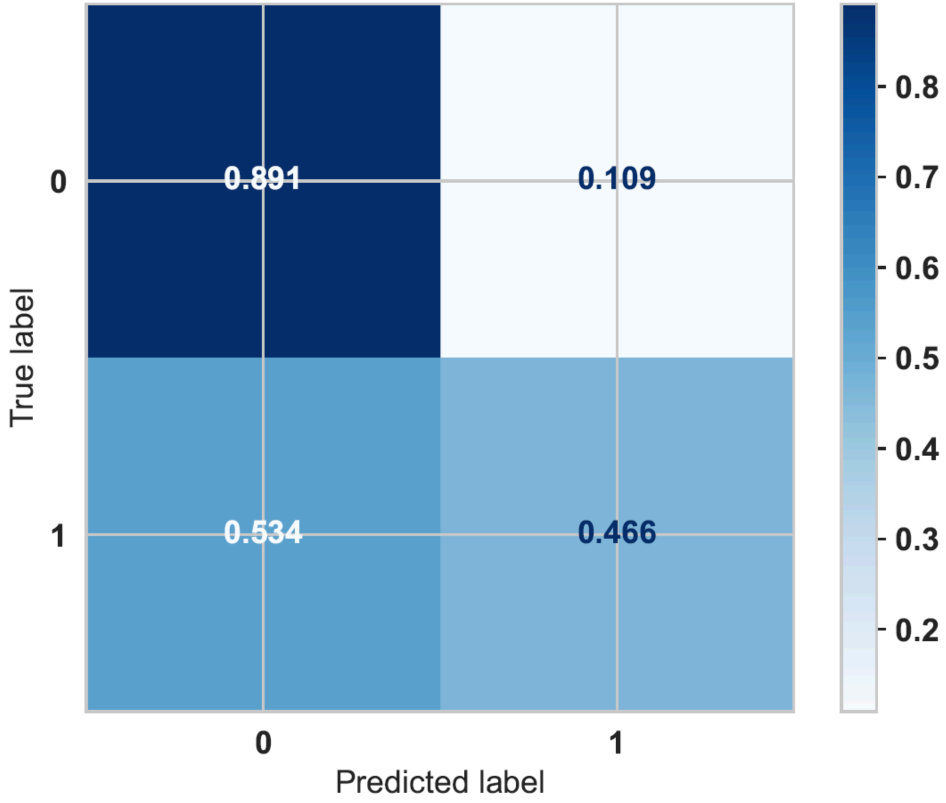

1.3.3.8. lightgbm

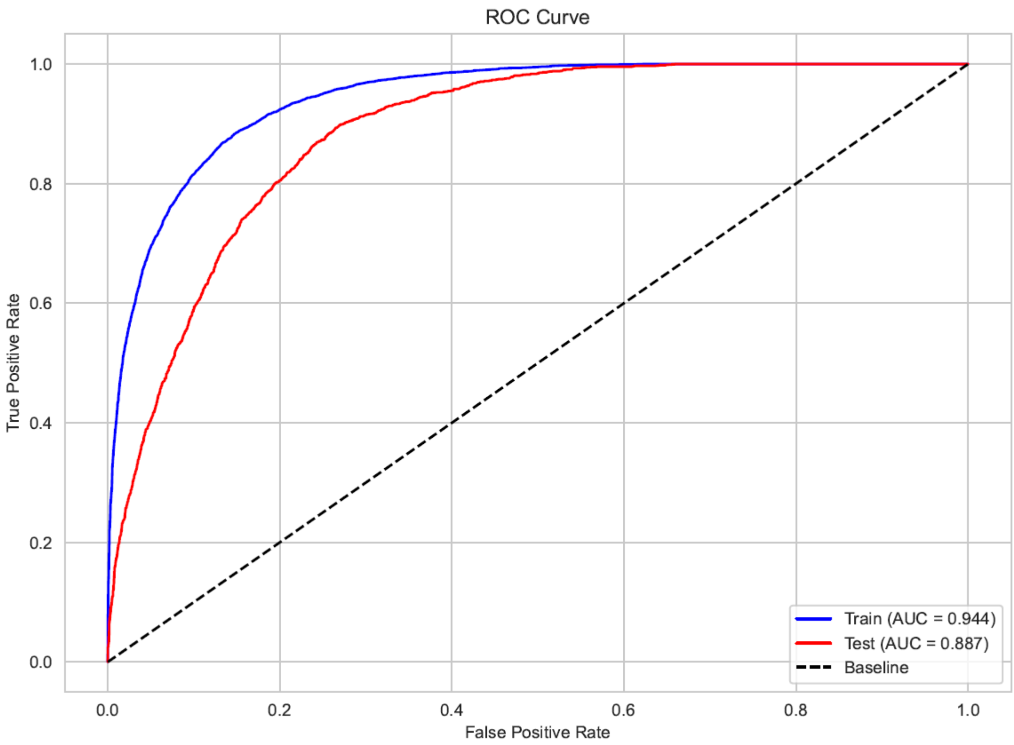

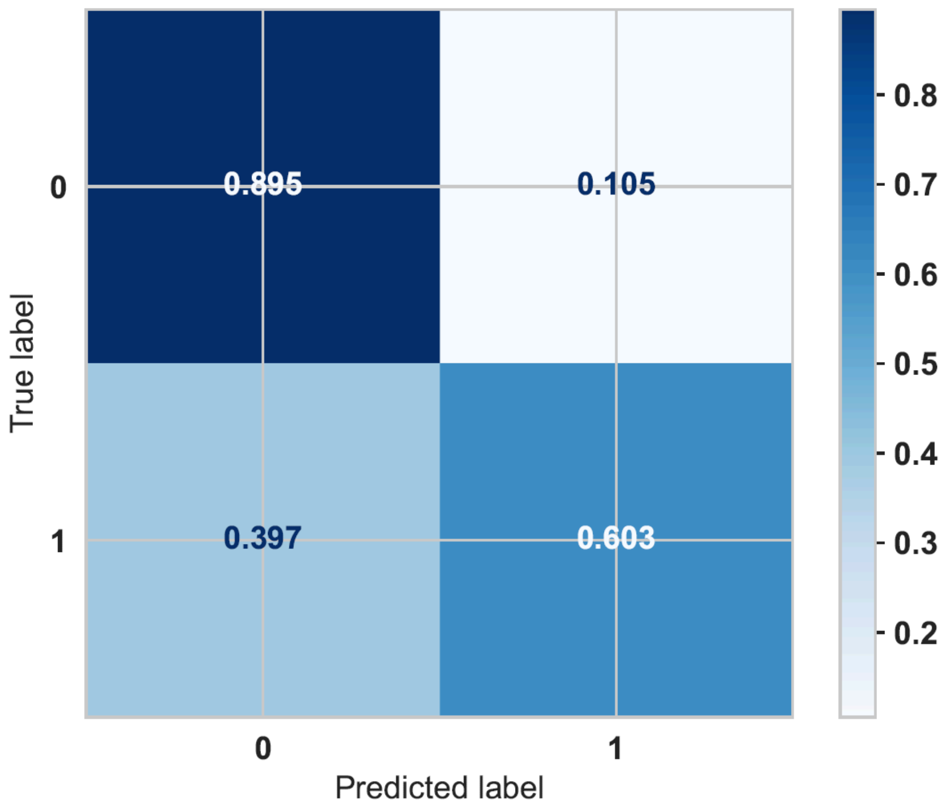

1.3.4. Cancer death

1.3.4.1. Random Forest

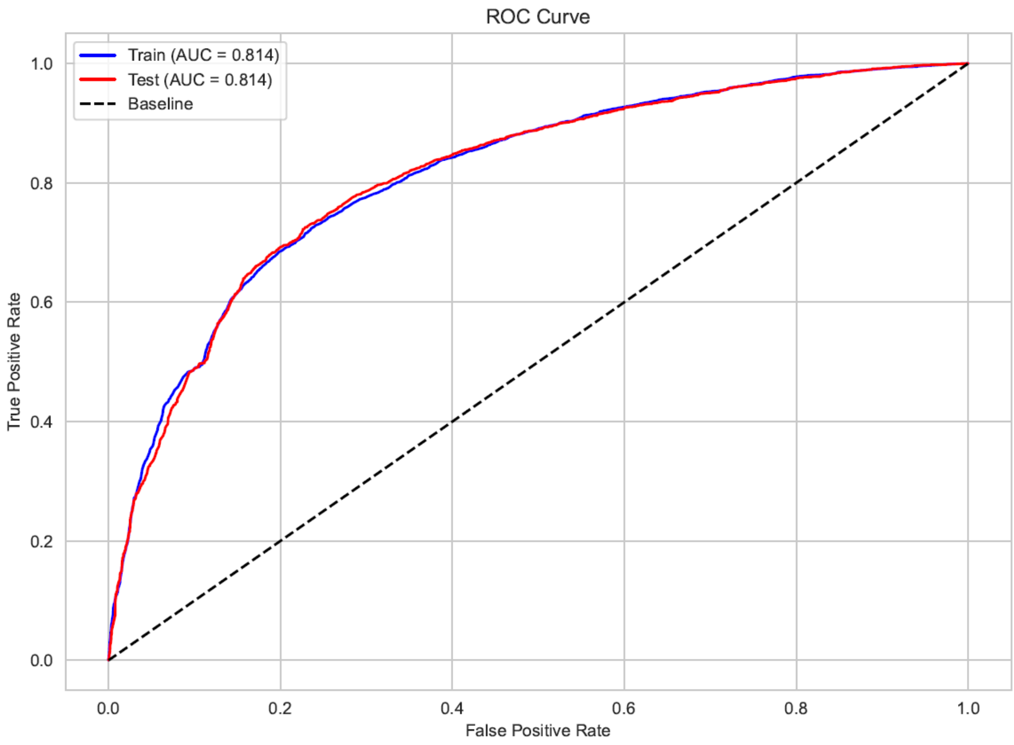

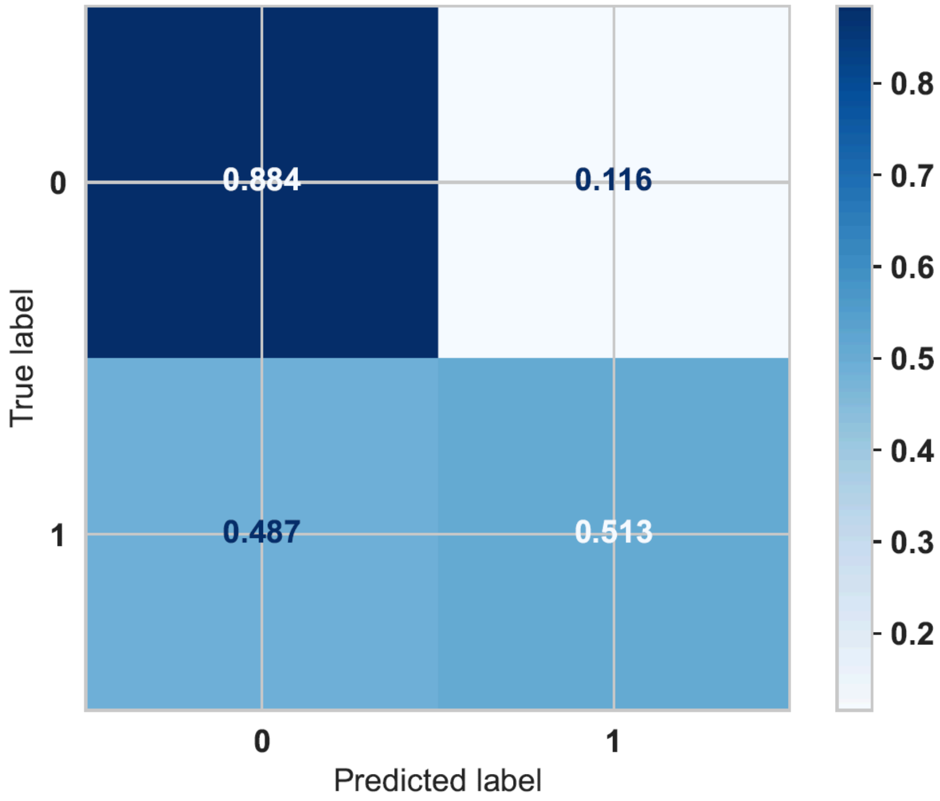

1.3.4.2. XGBoost

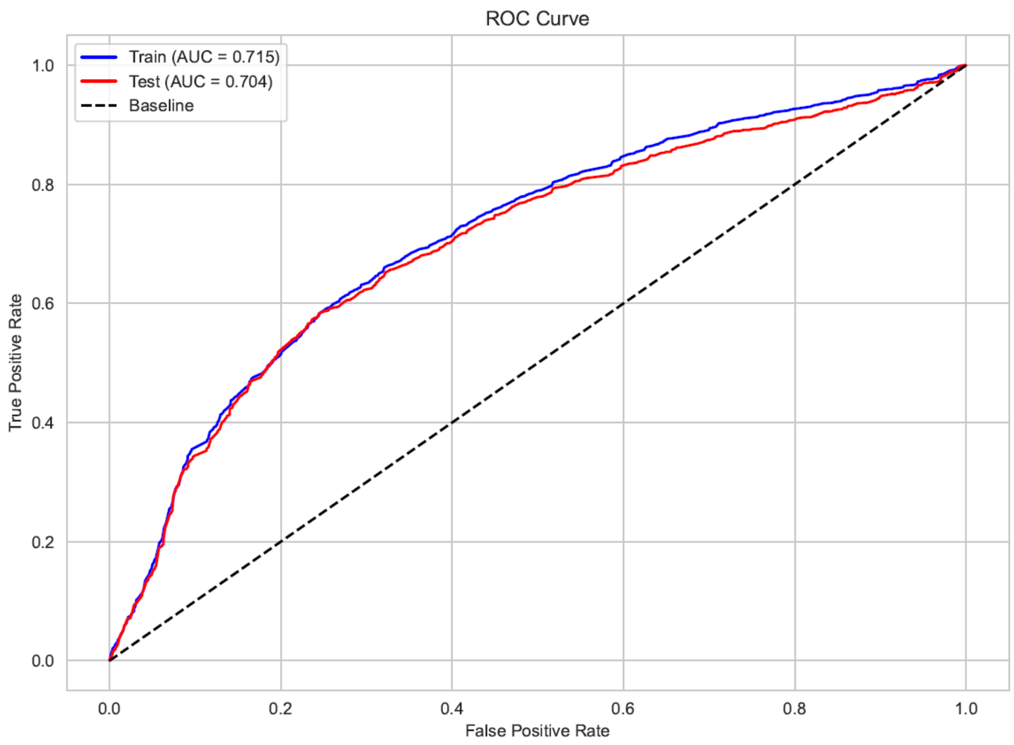

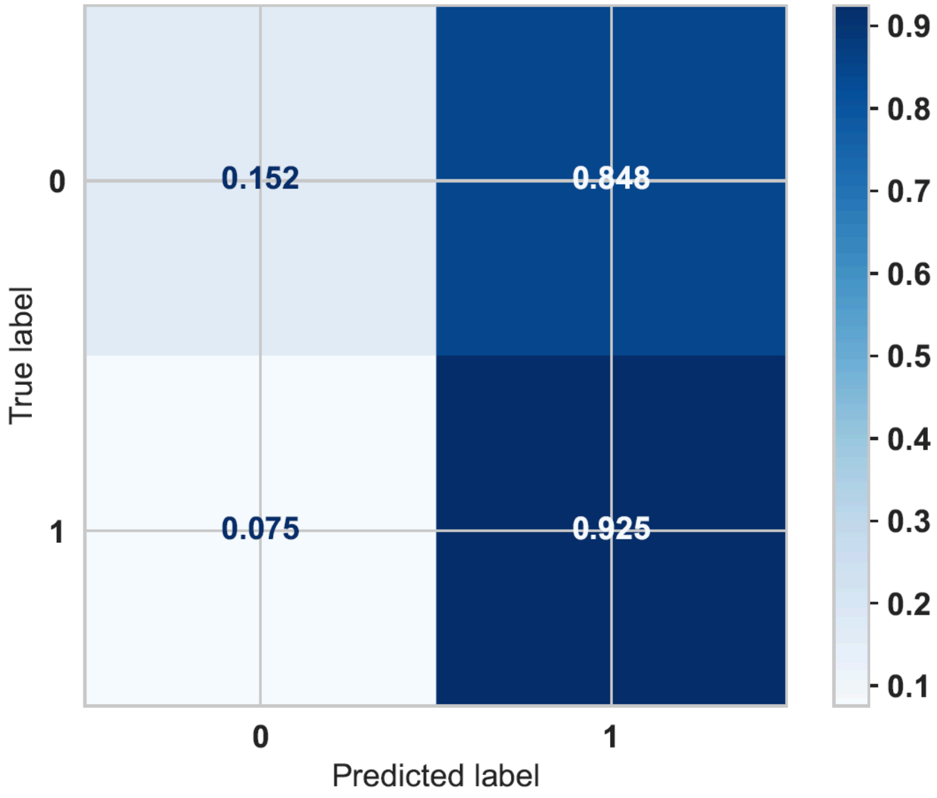

1.3.4.3. CatBoost

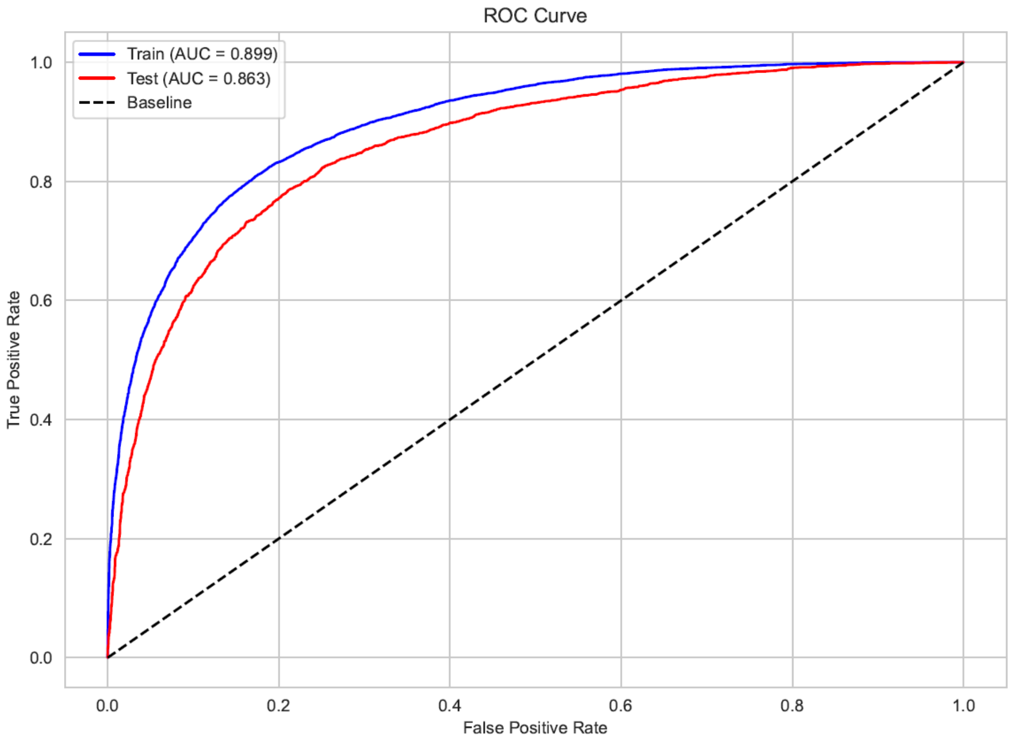

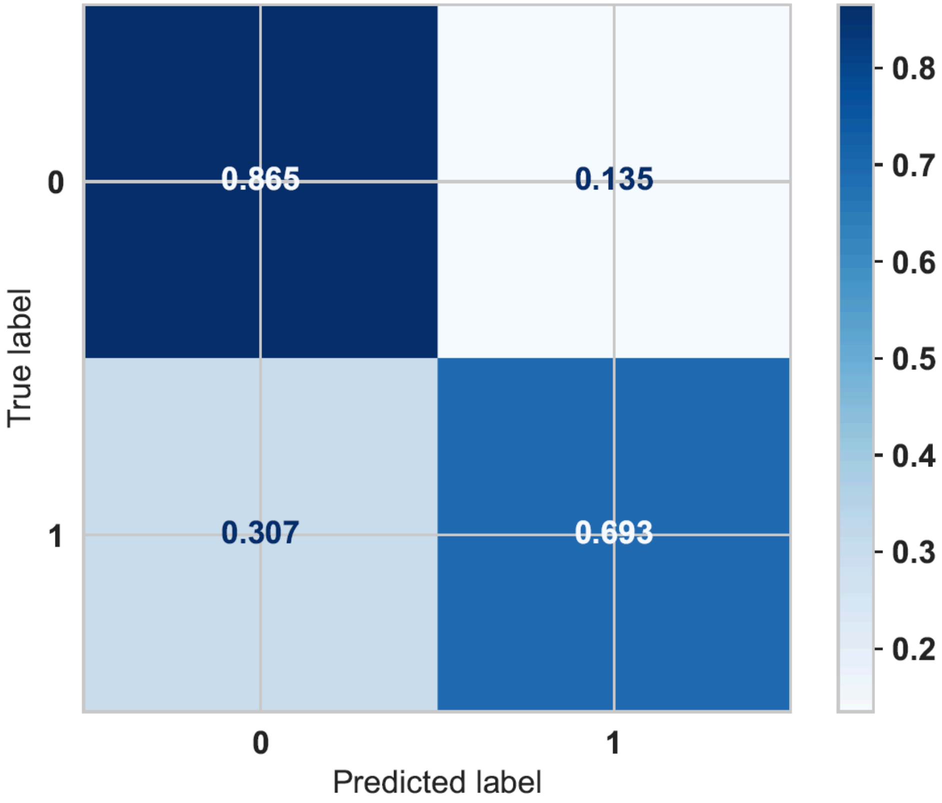

1.3.4.4. DecisionTreeClassifier

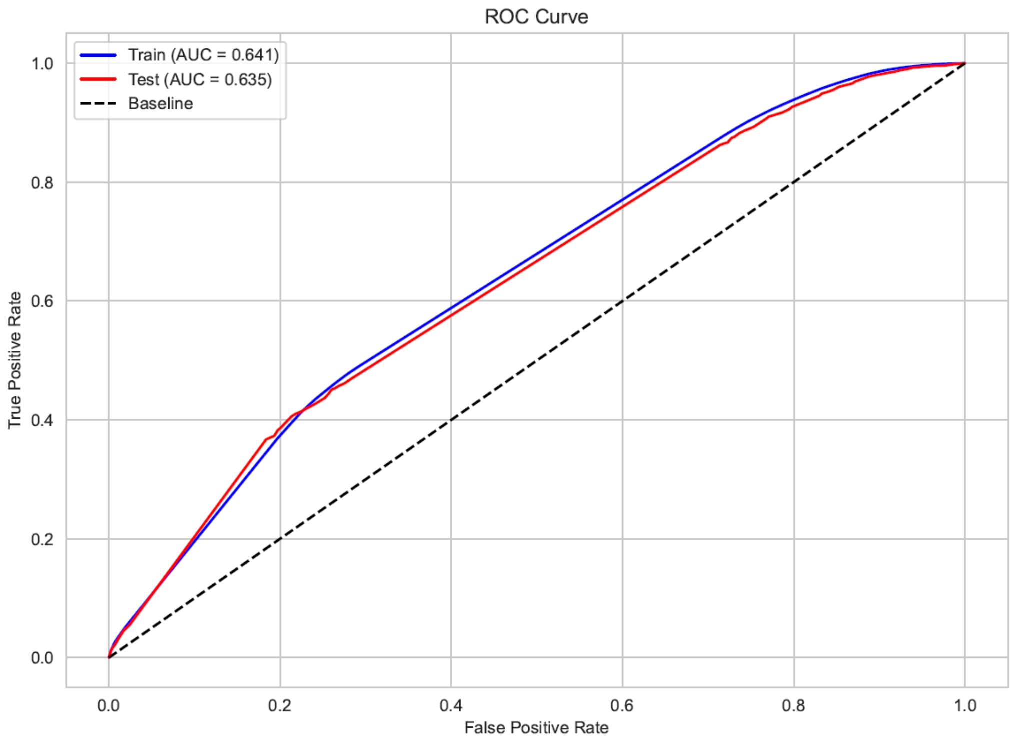

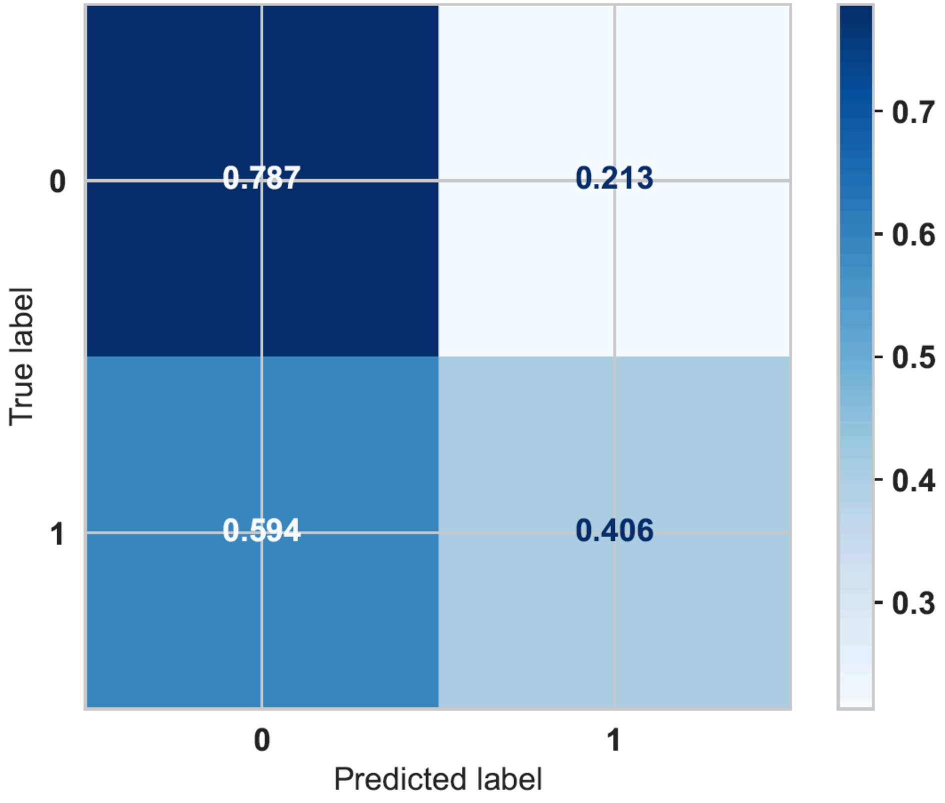

1.3.4.5. ExtraTreesClassifier

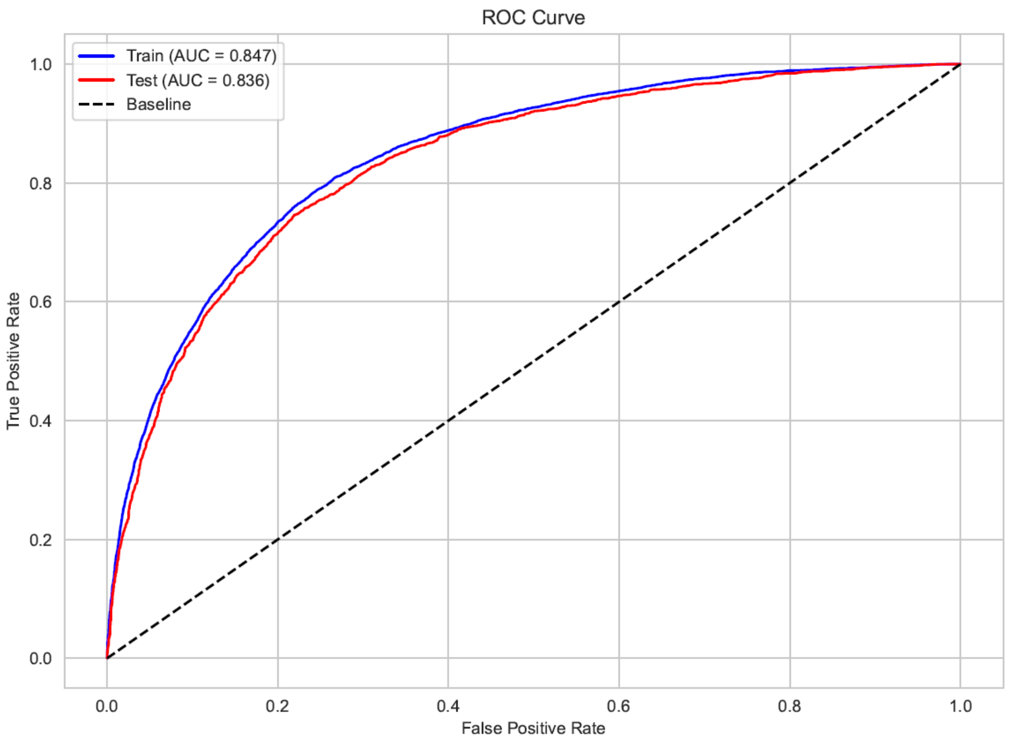

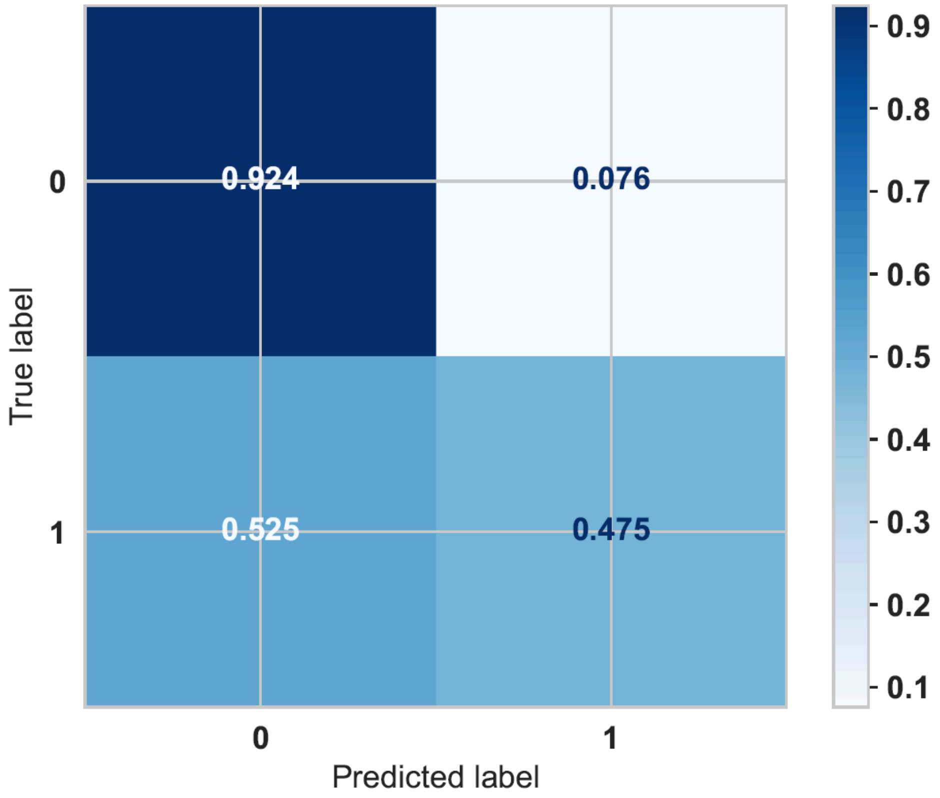

1.3.4.6. GradientBoosting

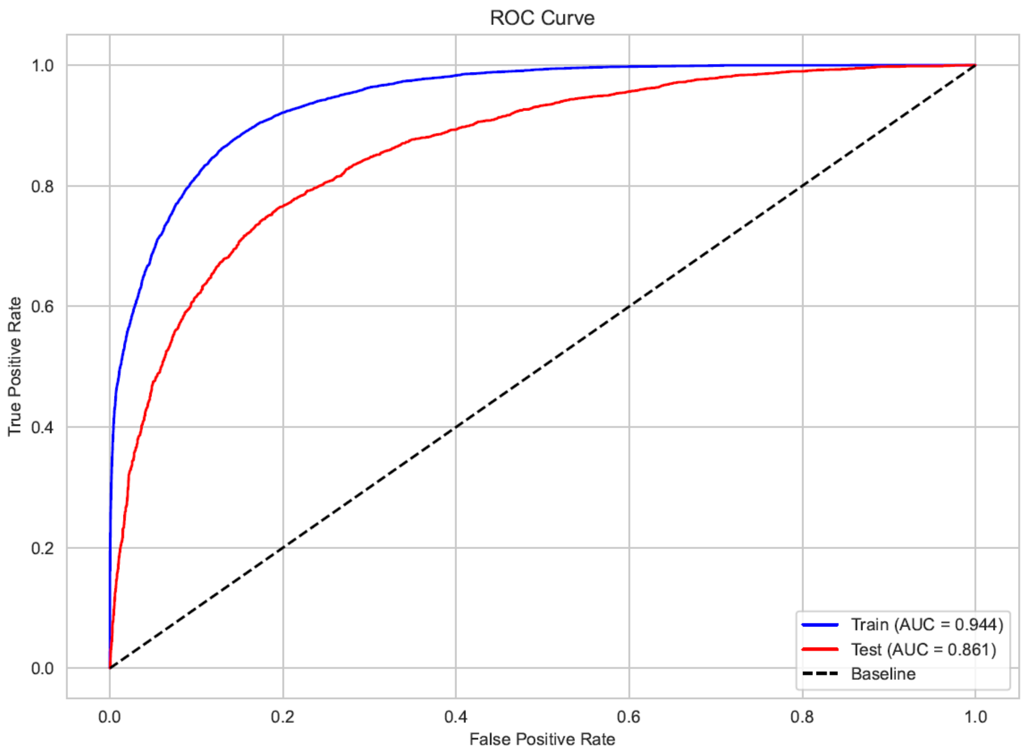
ROC Curve
True Positive Rate
False Positive Rate
0.0
0.2
0.4
0.6
0.8
1.0
Train (AUC = 0.944)
Test (AUC = 0.861)
Baseline

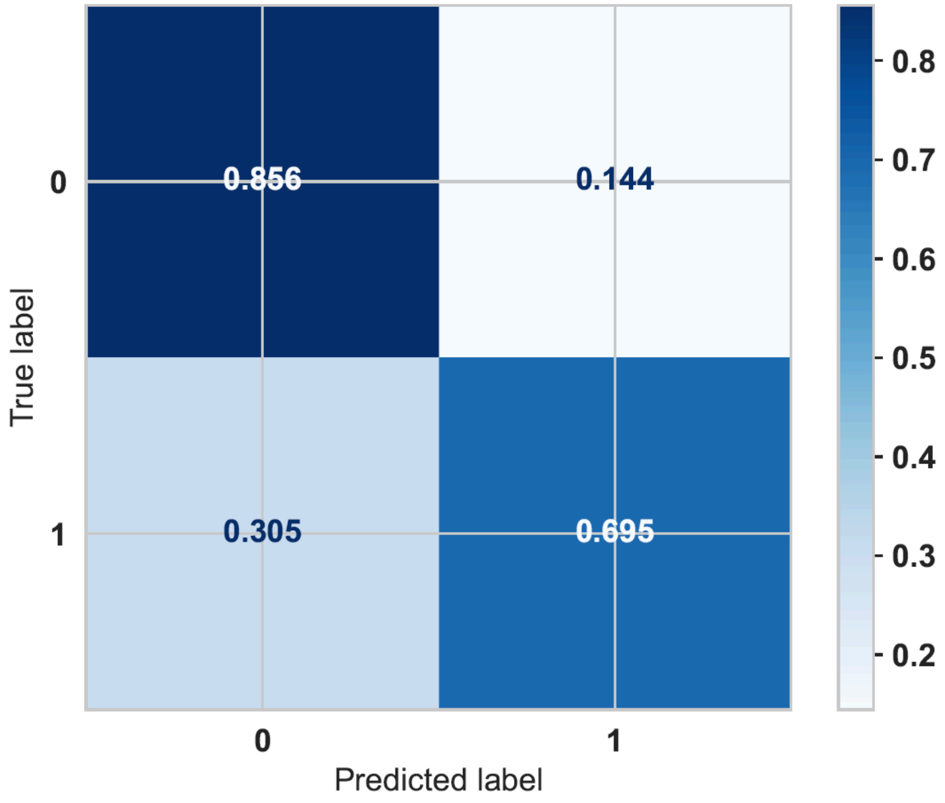
0.856
0.144
0.305
0.695
True label
Predicted label
0
1
0.8
0.7
0.6
0.5
0.4
0.3
0.2

1.3.4.7. KNN

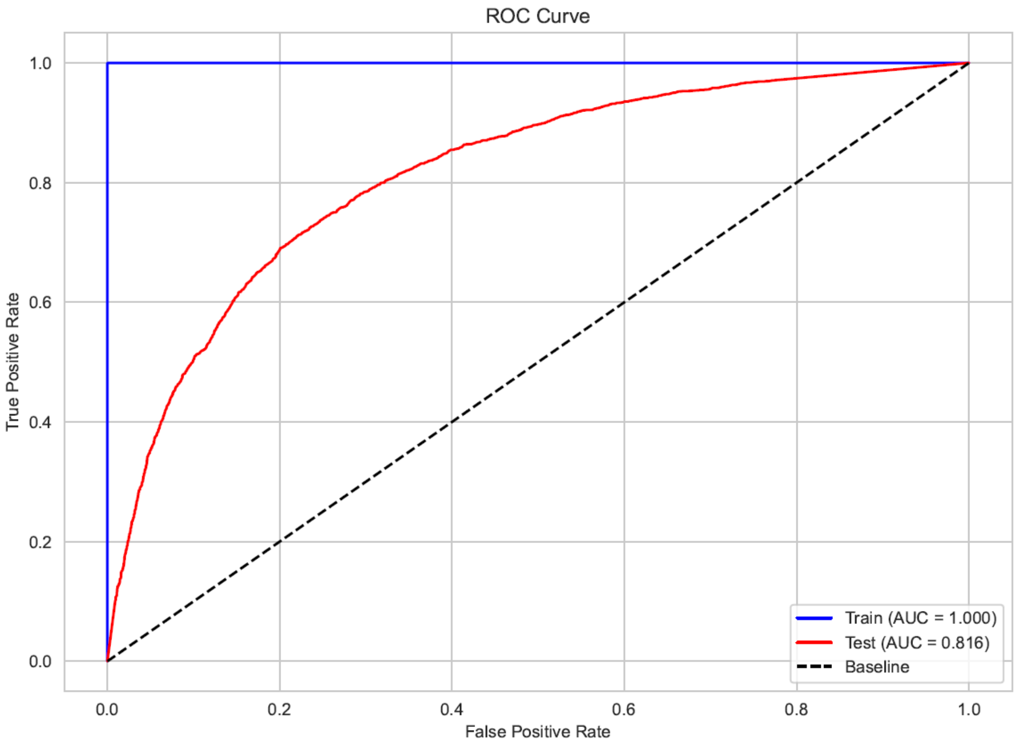

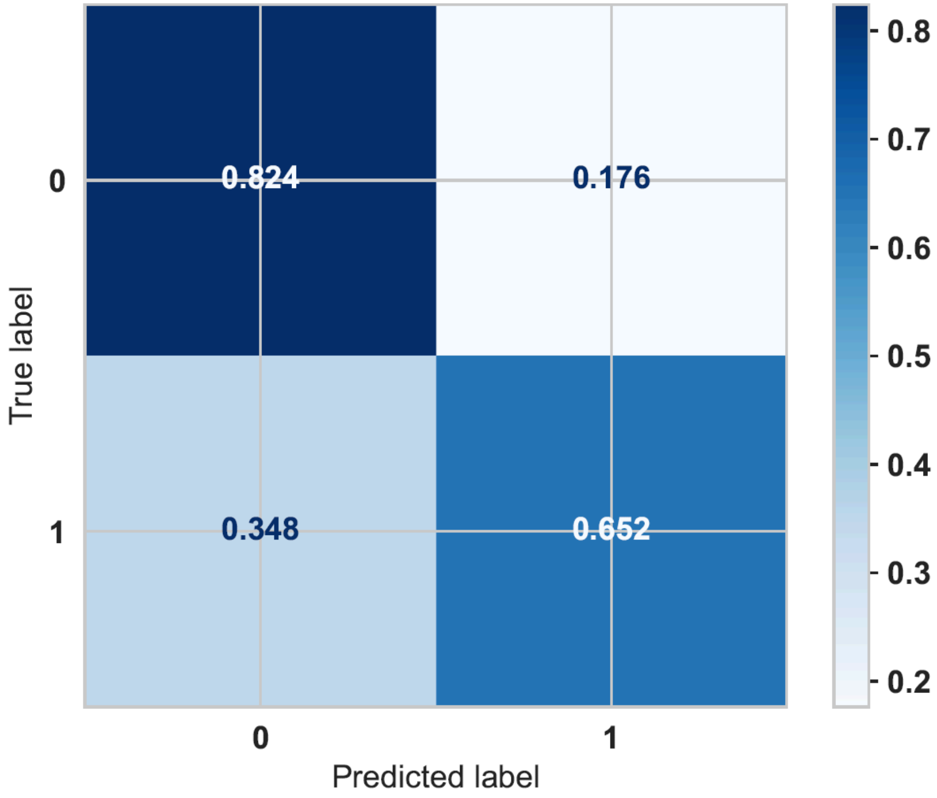

1.3.4.8. lightgbm

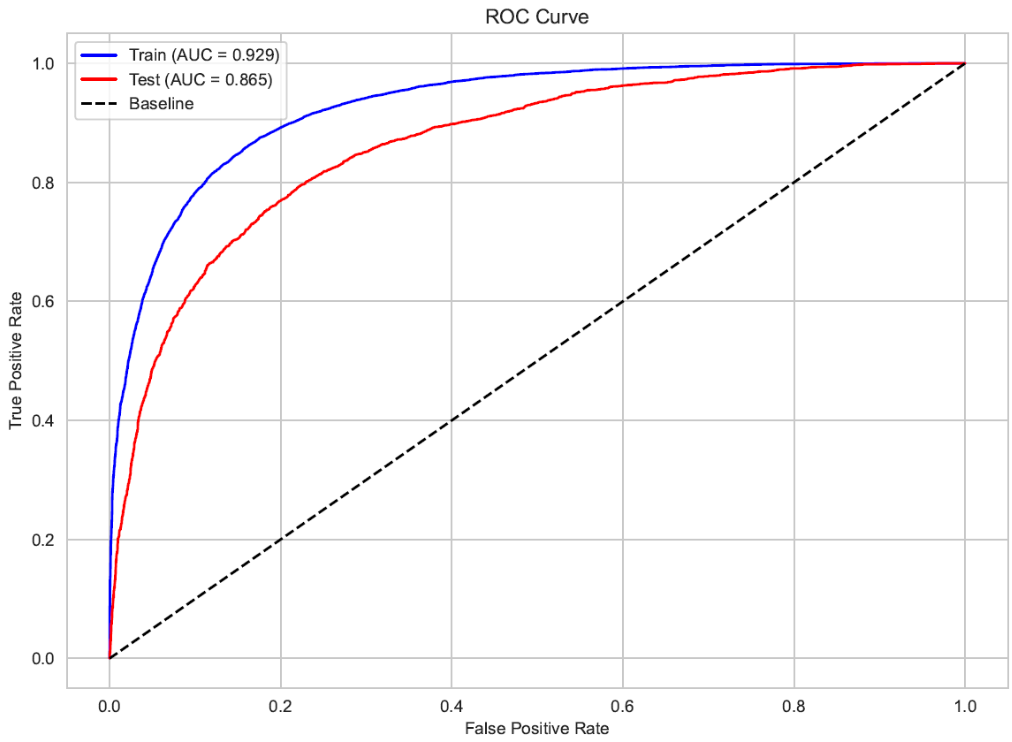

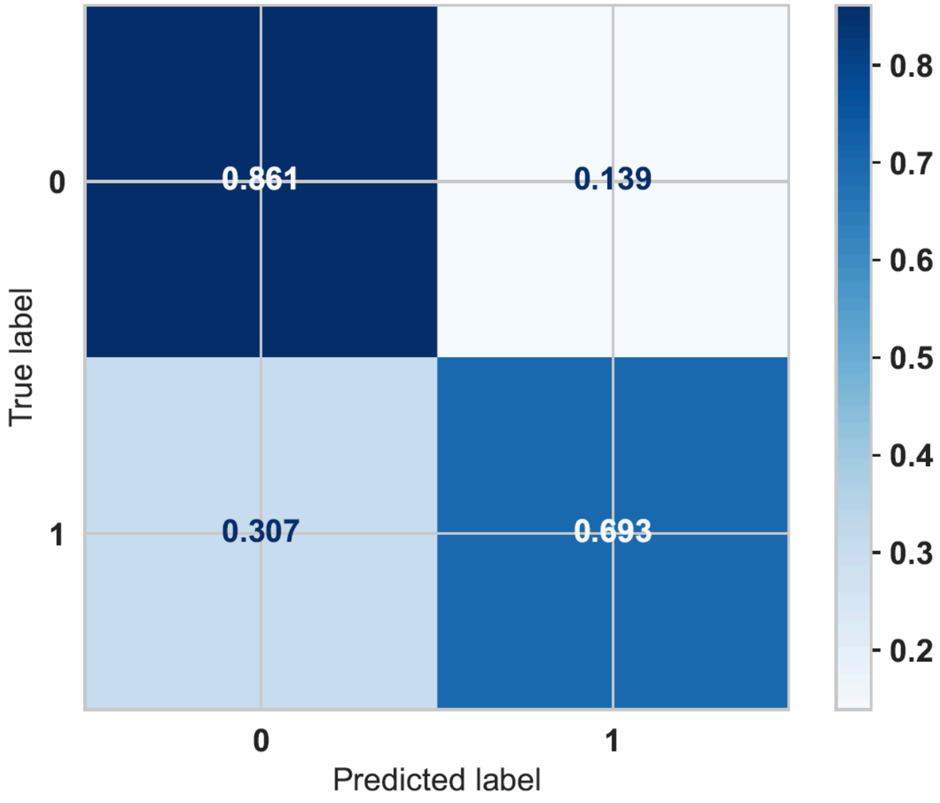

1.3.5. Overall death

1.3.5.1. Random Forest

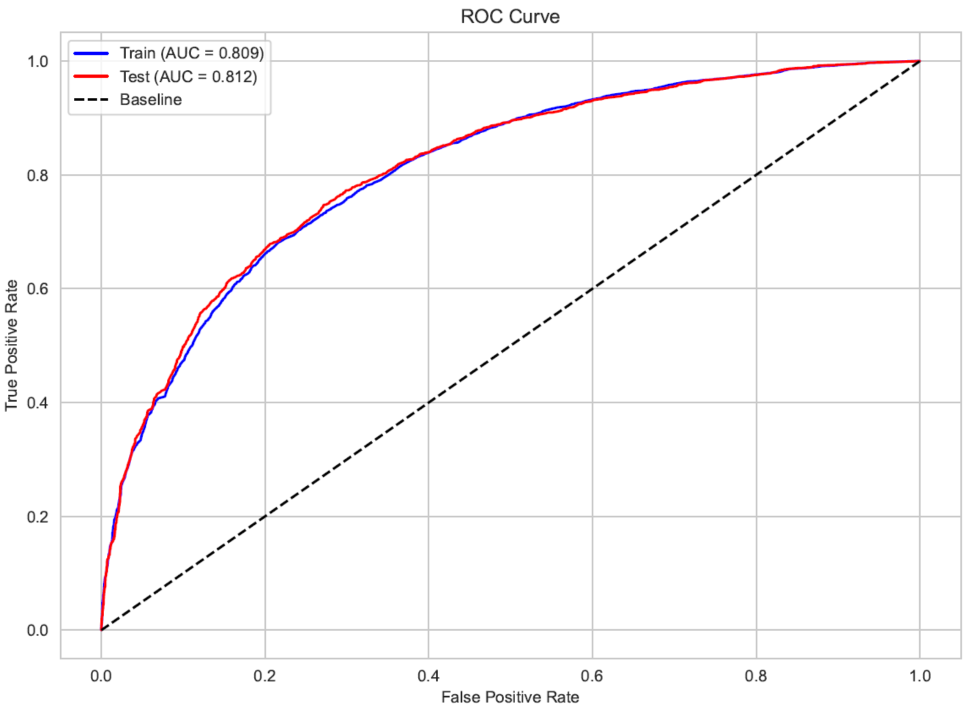

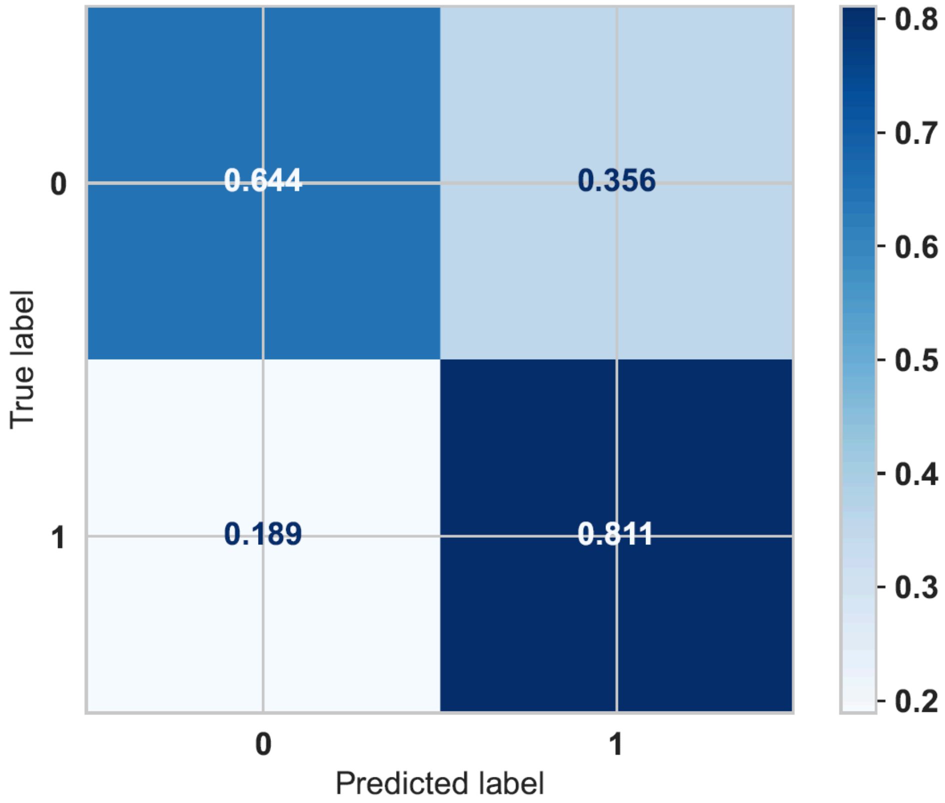

1.3.5.2. XGBoost

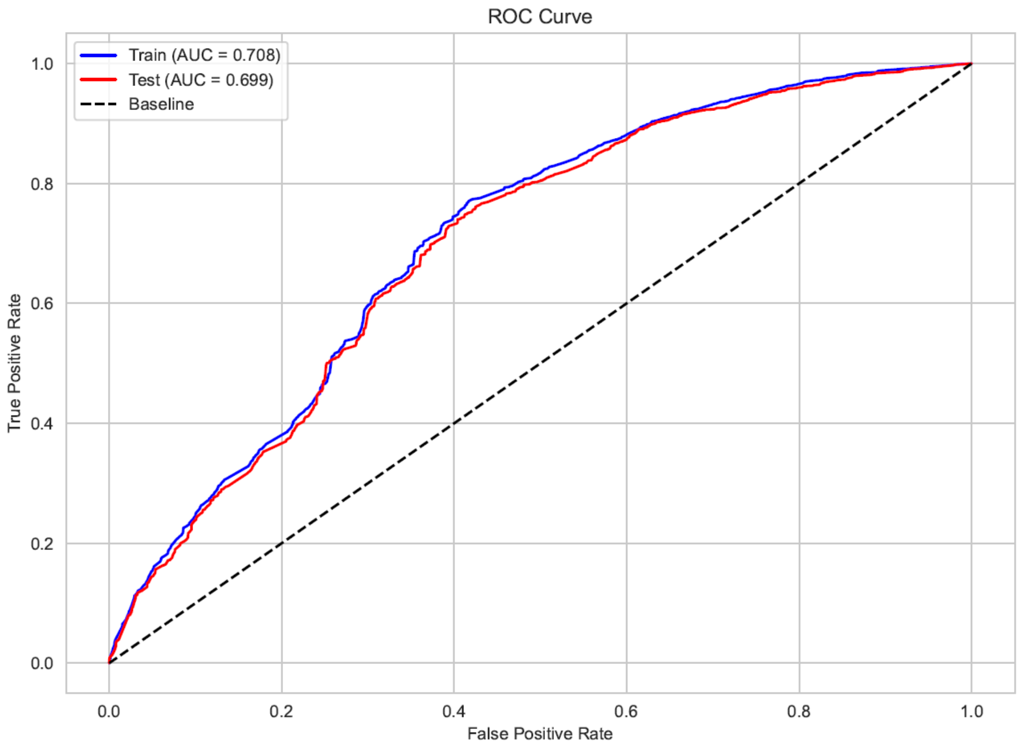

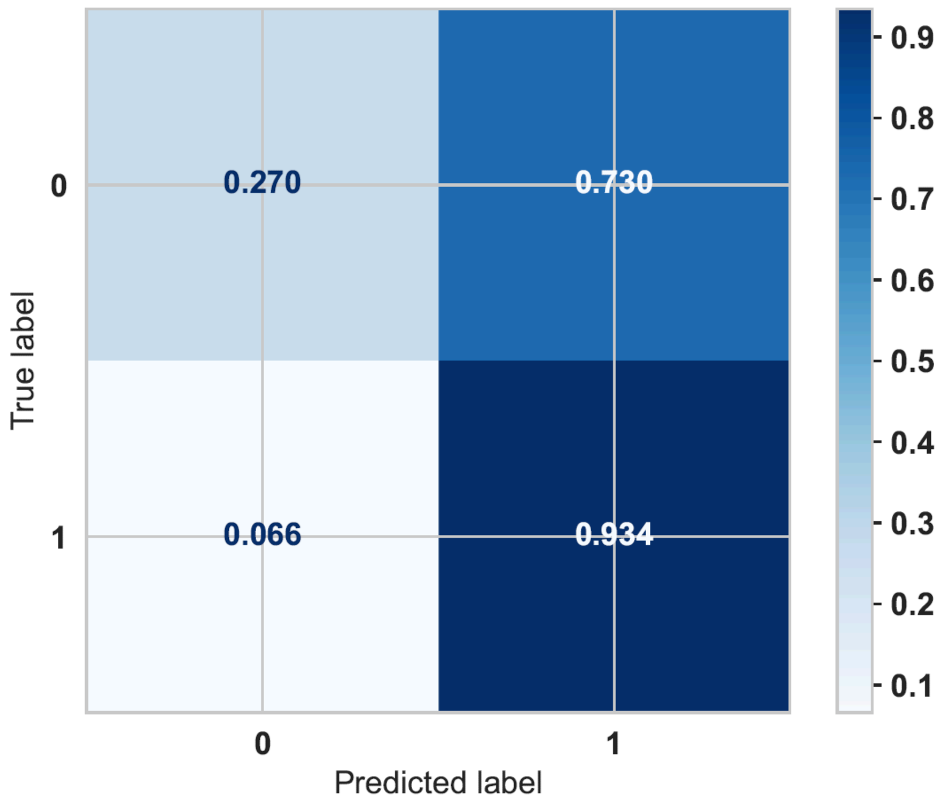

1.3.5.3. CatBoost

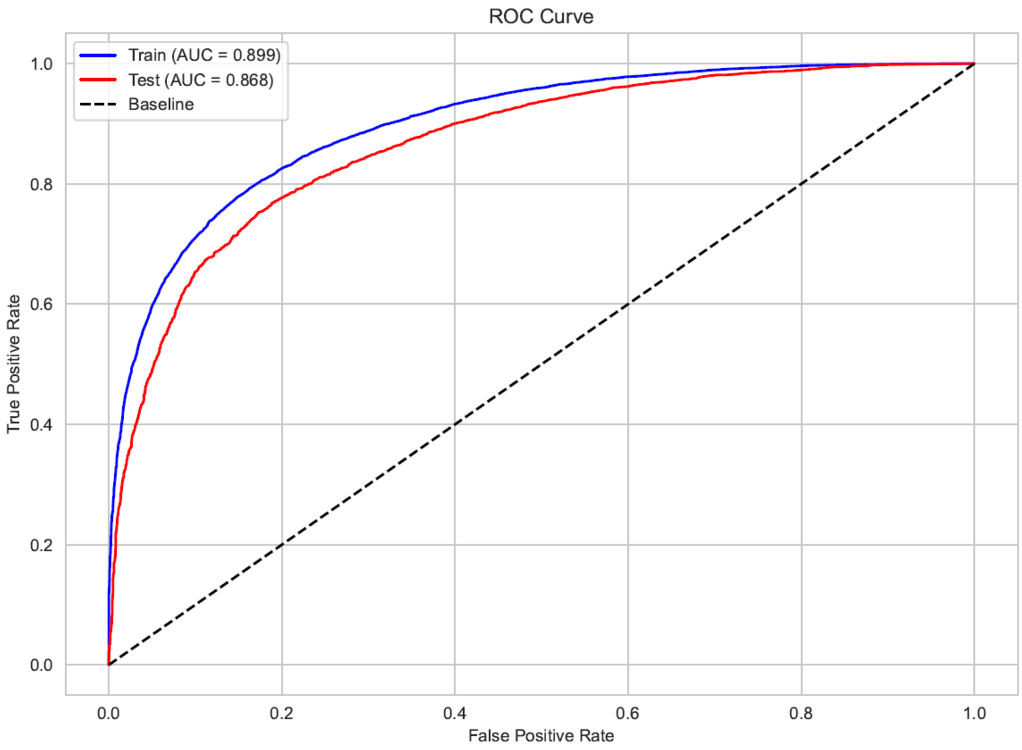

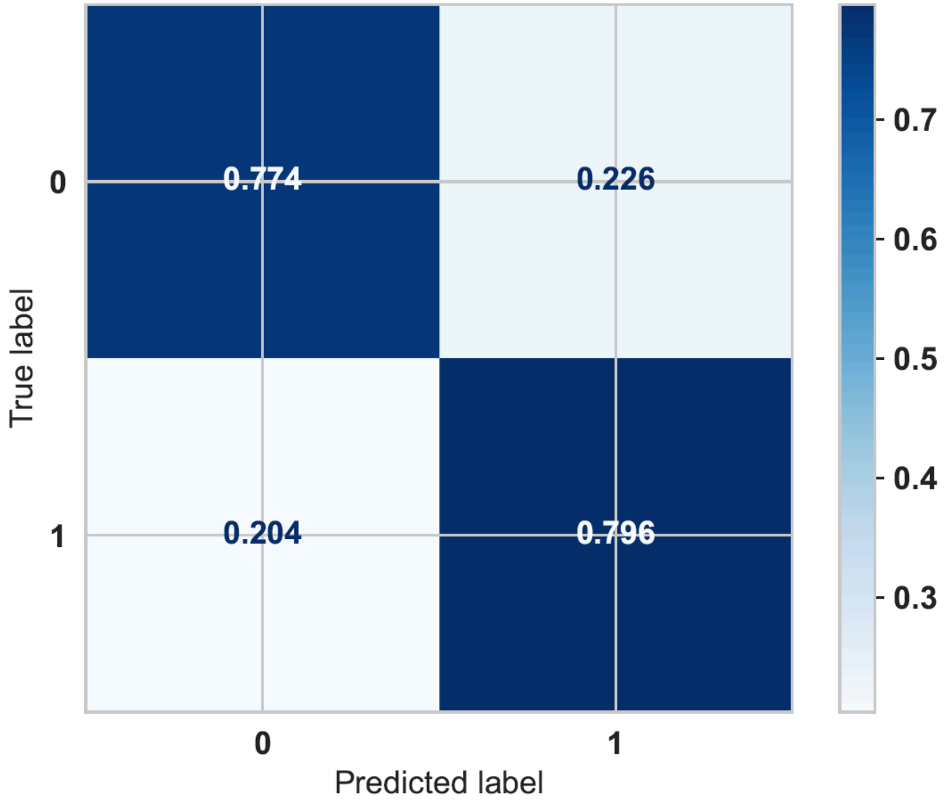

1.3.5.4. DecisionTreeClassifier

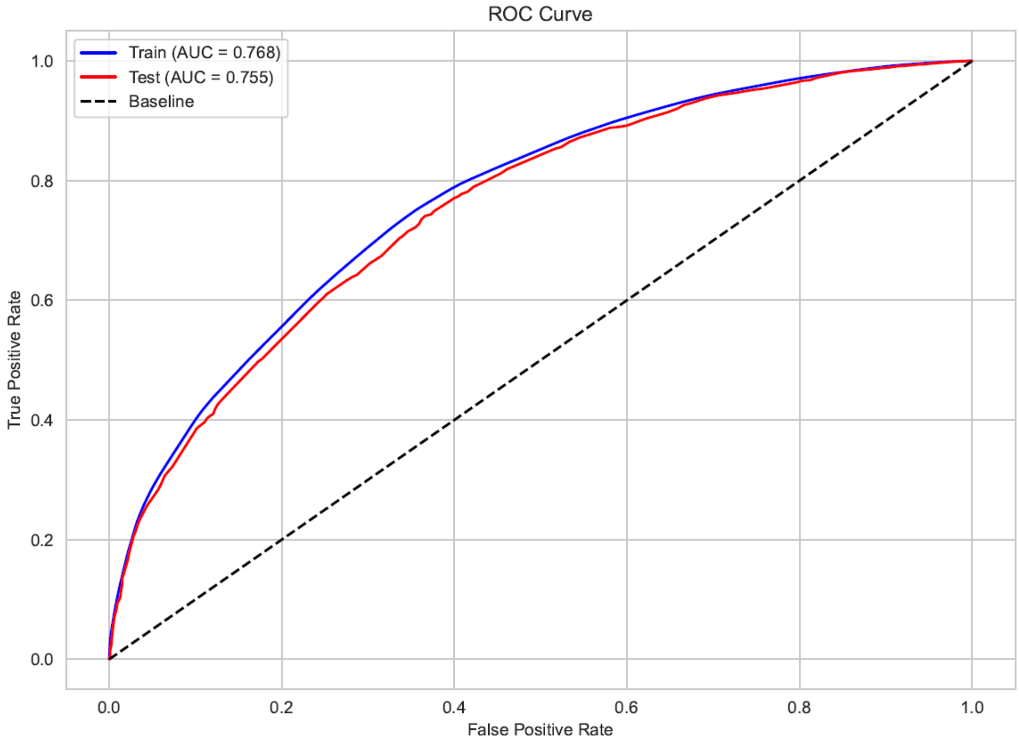

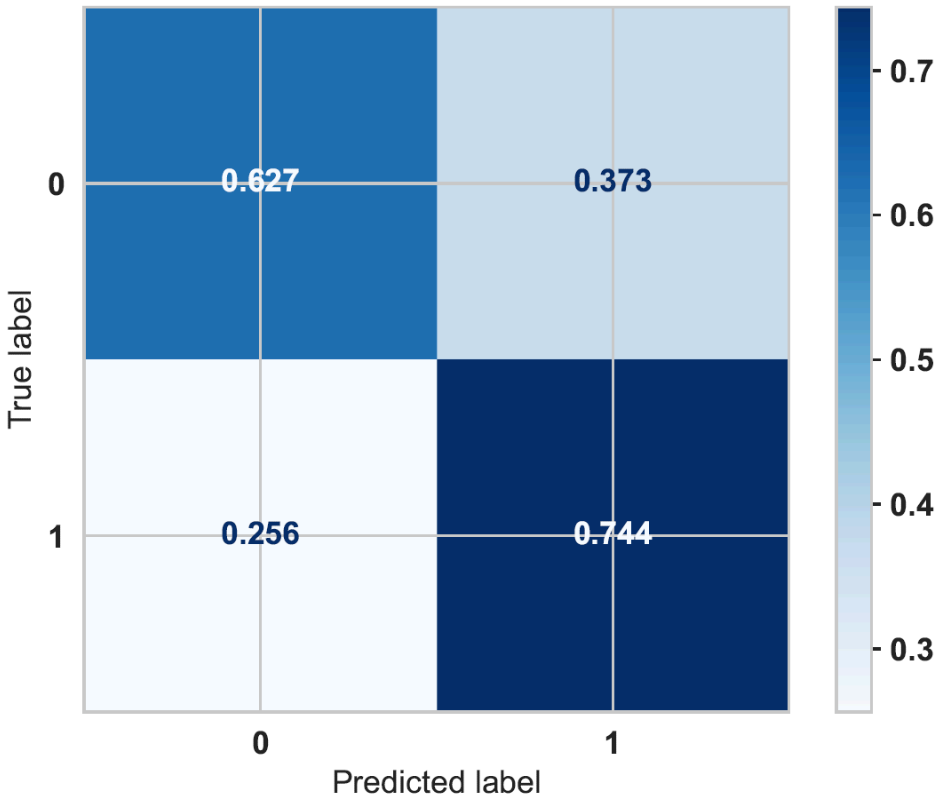

1.3.5.5. ExtraTreesClassifier

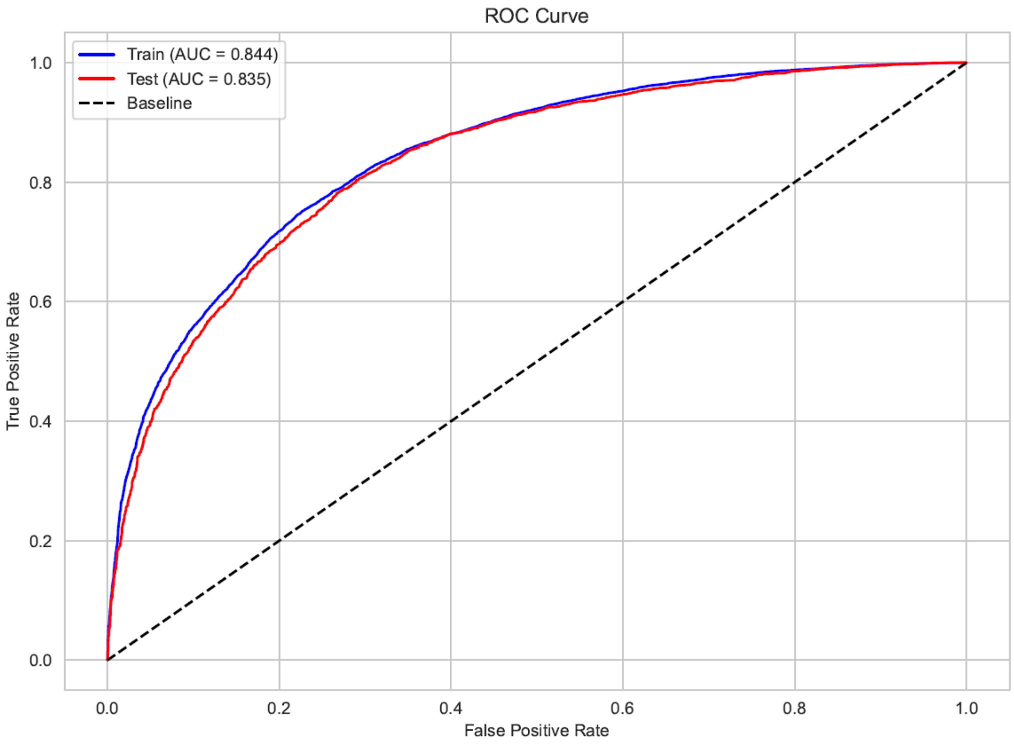

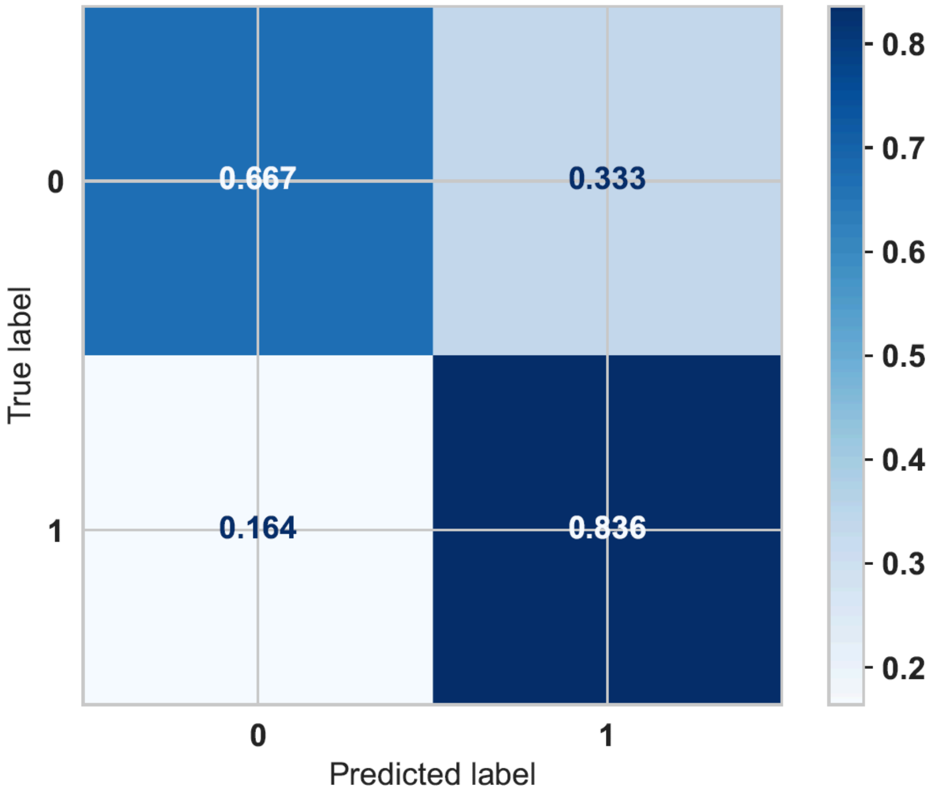

1.3.5.6. GradientBoosting

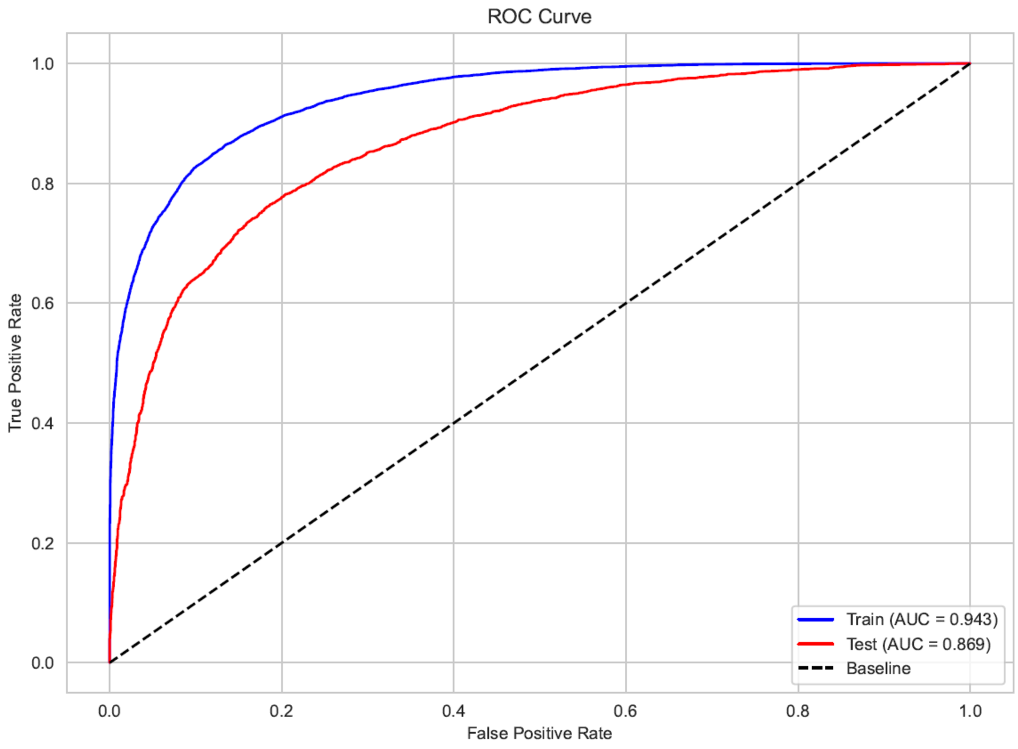

ROC Curve
True Positive Rate
False Positive Rate
0.0
0.2
0.4
0.6
0.8
1.0
Train (AUC = 0.943)
Test (AUC = 0.869)
Baseline

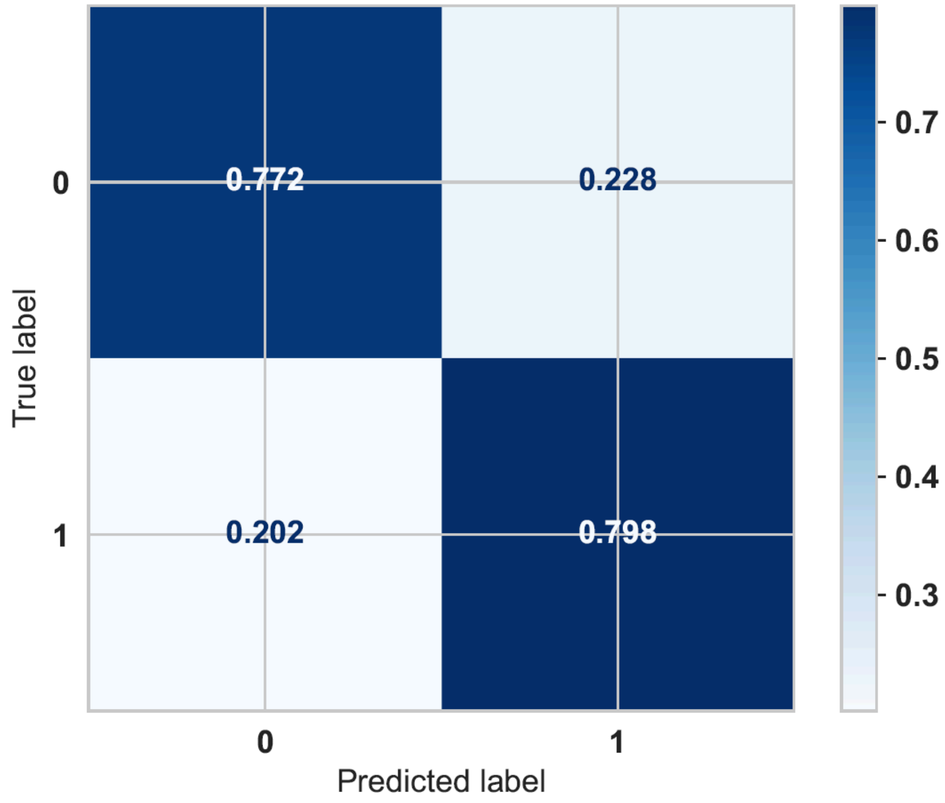

0.772
0.228
0.202
0.798
True label
Predicted label
0
1
0.7
0.6
0.5
0.4
0.3

1.3.5.7. KNN

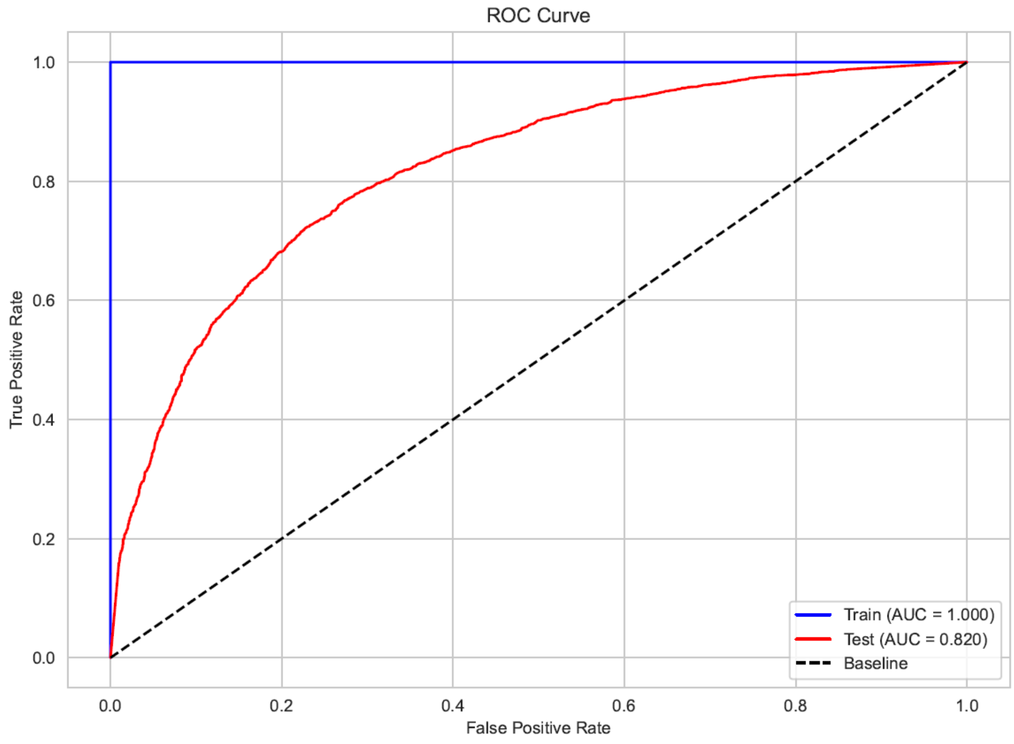

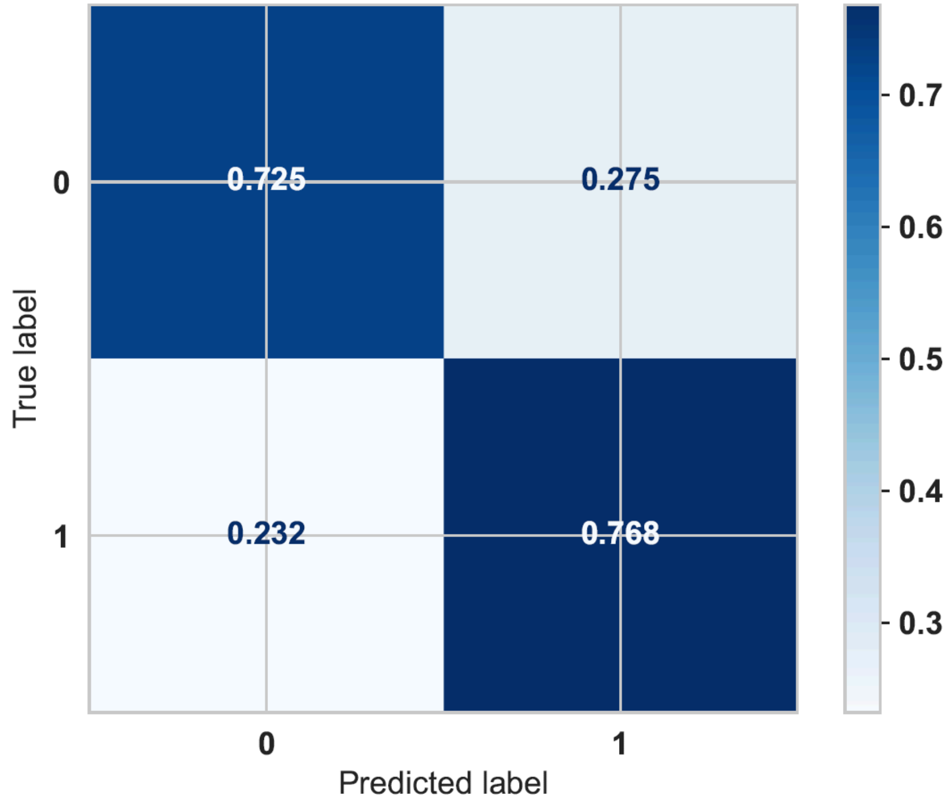

1.3.5.8. lightgbm

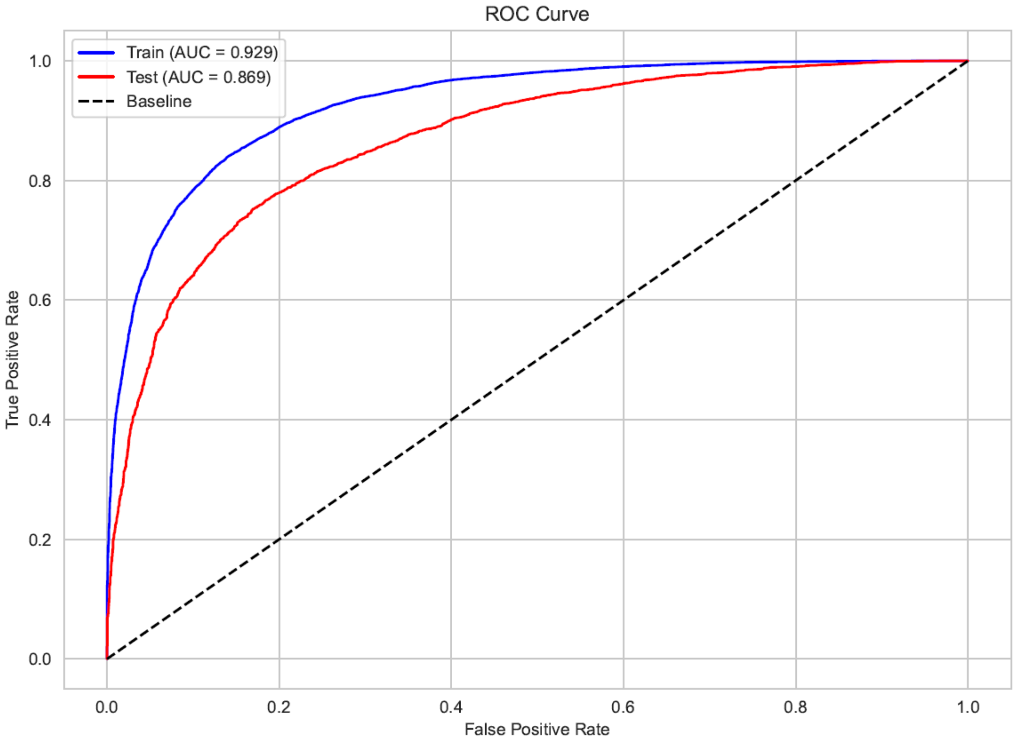
ROC Curve
Train (AUC = 0.929)
Test (AUC = 0.869)
Baseline
True Positive Rate
False Positive Rate
0.0
0.2
0.4
0.6
0.8
1.0

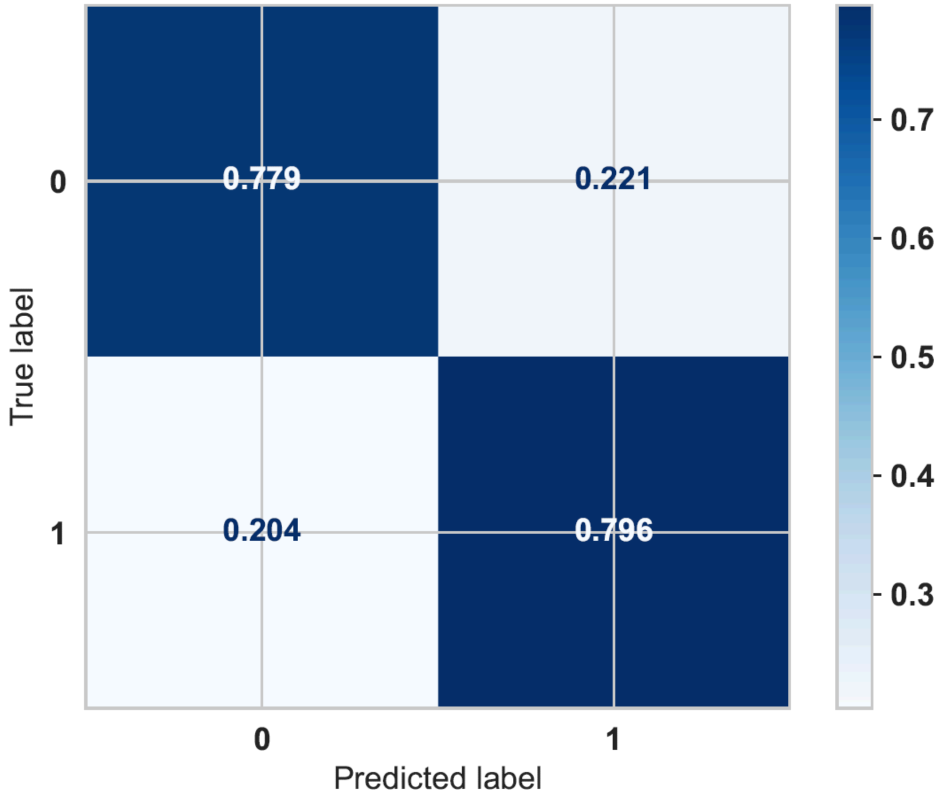
0.779
0.221
0.204
0.796
True label
Predicted label
0
1
0.7
0.6
0.5
0.4
0.3
